# GoIFISH: a system for the quantification of single cell heterogeneity from IFISH images

**DOI:** 10.1186/s13059-014-0442-y

**Published:** 2014-08-26

**Authors:** Anne Trinh, Inga H Rye, Vanessa Almendro, Åslaug Helland, Hege G Russnes, Florian Markowetz

**Affiliations:** University of Cambridge, Cancer Research UK Cambridge Institute, Robinson Way, CB2 0RE Cambridge UK; Department of Genetics, Institute for Cancer Research, Postboks 4950 Nydalen, 0424 Oslo Norway; K. G. Jebsen Centre for Breast Cancer Research, University of Oslo, Postboks 4950 Nydalen, 0424 Oslo Norway; Department of Medical Oncology, Dana-Farber Cancer Institute, Boston, US; Harvard Medical School, Boston, US; Department of Cancer treatment, Oslo University Hospital, Postboks 4950 Nydalen0424 Oslo, Norway; Department of Pathology, Oslo University Hospital, Postboks 4950 Nydalen, 0424 Oslo, Norway

## Abstract

Molecular analysis has revealed extensive intra-tumor heterogeneity in human cancer samples, but cannot identify cell-to-cell variations within the tissue microenvironment. In contrast, *in situ* analysis can identify genetic aberrations in phenotypically defined cell subpopulations while preserving tissue-context specificity. GoIFISH is a widely applicable, user-friendly system tailored for the objective and semi-automated visualization, detection and quantification of genomic alterations and protein expression obtained from fluorescence *in situ* analysis. In a sample set of HER2-positive breast cancers GoIFISH is highly robust in visual analysis and its accuracy compares favorably to other leading image analysis methods. GoIFISH is freely available at www.sourceforge.net/projects/goifish/.

**Electronic supplementary material:**

The online version of this article (doi:10.1186/s13059-014-0442-y) contains supplementary material, which is available to authorized users.

## Quantifying cell-to-cell heterogeneity in the tissue context

Intra-tumor heterogeneity is currently accepted as a hallmark of cancer, being present in virtually all tumor traits [1]. Sensitive molecular techniques developed in the last few years have allowed a detailed genetic and phenotypic deconvolution of intra-tumor heterogeneity. These include genome-wide analysis of bulk tumor samples to describe evolutionary trajectories in relapsed tumors and genomic divergence between primary tumors and metastases [2-4], as well as single-cell genomic profiling [2,5]. However, despite methodological improvements in the molecular characterization of single cells, the accurate interpretation of intra-tumor heterogeneity requires the inference of cell-to-cell variability within a particular tissue context, which can only be directly assessed by *in situ* analysis. Microenvironmental constraints within spatially restricted areas of a tumor can exert differential selective pressures, leading to the manifestation and the selection of different phenotypes and particular genotypes. For instance, different oxygen levels, the presence of inflammatory cells, or the physical interaction with extracellular components in different parts of a tumor [6-8] can influence cellular phenotypes and contribute to different trajectories in the evolution of a tumor [2]. Therefore, the accurate interpretation of cellular phenotypic and genomic heterogeneity requires tissue-context specificity [9-11].

**IFISH: Immunofluorescence ***in situ***hybridization***In situ* fluorescence-based detection of proteins, DNA, and RNA enables the simultaneous detection of multiple markers in single cells by epifluorescence, confocal, or multispectral imaging technology. Combining both immunofluorescence and fluorescence *in situ* hybridization (IFISH) allows multiplexing to detect both genomic and phenotypic traits at the single cell level [10]. This approach captures cell-to-cell variations missed in cell population analyses while preserving specific microenvironmental contexts. As an *in situ* analysis, IFISH allows the spatial mapping of individual cells to measure topological heterogeneity. Visualizing topological heterogeneity can have important implications in predicting treatment response, as well as tailoring treatment to suit the diverse cell populations observed within a tumor [10]. However, these *in situ* studies require the analysis of multiple markers in thousands of cells, are very time-consuming, and their reproducibility could be influenced by variability between users [12]. Therefore, there is an urgent need for the development of objective analytical tools that minimize scoring subjectivity and facilitate the quantification of multiple traits in single cells while preserving context specificity. The implementation of these tools in both basic and translational research will advance our understanding of tumor biology and will facilitate biomarker discovery and validation.

**GoIFISH**:**quantifying tumor heterogeneity in IFISH images** For application to IFISH, accurate segmentation at the nuclear, membrane and spot level are critical for subsequent analysis, which often interrogates clonal populations or evaluates relationships between protein and genomic expression. Objective integration of protein expression and copy number requires not only accurate segmentation, but also the separation of normal cells from tumor cells, and appropriate background subtraction associated with auto-fluorescence. Very few existing softwares allow manual alterations of small inaccuracies in cell segmentation and often incorrect cell classification results cannot be changed. Visual scoring by a trained observer (e.g. pathologist) is the gold standard for detecting cells and automated image analysis systems developed to complement pathologist scoring require user validation [12].

To address these challenges, we have developed a semi-automated system which provides users with an automated starting point in segmentation and can readily accept user input to improve the segmentation result. GoIFISH is able to segment nuclei, locate and estimate spots, detect membranes, measure morphological and intensity properties and classify cells. GoIFISH is a versatile method that allows researchers to determine and quantify, for instance, the amplification status of single locus within cells, together with the detection of phenotypic markers present in different subcellular locations. It preserves the tissue context specificity and provides coordinates for the topological mapping of each cell. Simple topology maps can be displayed to illustrate spatial variations within an image with respect to two given stains. GoIFISH allows users to analyze FISH, IF or IFISH images containing a maximum of 5 markers, of which one must be the nuclear marker DAPI. We validated our software in a pilot HER2+ breast cancer cohort of 10 samples and compared its performance with existing softwares.

**Related approaches** Several general image processing methods including CellProfiler [13], Icy [14], OMERO [15], ImageJ [16], CellTracker [17] and ImageM [18] have been developed for the quantitative analysis of images, and the capabilities of each software are described in Table 1.

**Table 1:** **Comparison between softwares available for cell segmentation**

**Property**	**GoIFISH**	**Columbus**	**CellProfiler**	**ImageJ**	**ImageM**	**CellTracker**
Cost	Open-Source	Proprietary	Open-Source	Open-Source	Open-Source on request	Open-Source
Real-Time Update	Yes	Yes	No	No	No	Yes
Background Intensity Subtraction	Yes	Tuning but no subtraction	Correct Illumination Calculate	Flatten Illumination	No	No
A-Priori knowledge required (eg. cell size or segmentation methods)	No	No	Yes	No	No	No
Nuclear Segmentation	Yes	Yes	Yes	Yes	Manual	Yes
Membrane Segmentation	Yes	Yes	Yes	Yes	Manual	Yes
Spot Detection	Yes	Yes	Yes	Yes	Yes	No
Batch Processing	CmdLine Only	Yes	Yes	BatchCommand Plugin	No	No
Visualise Batch Segmentation Results	Yes	No	Yes	No	No	No
Manual Editing	Yes	No	No	No	Yes	Yes
Cell Specific Information	Yes	Yes	Yes	Yes	Yes	Yes
Summary Report	Yes	Yes	Yes	Yes	Yes	Yes
Topology or summary Maps	Yes	No	Yes	Topit Plugin	No	No

OMERO is a platform for the storage and annotation of microscopy images [15], and Icy and ImageJ have been developed as general platforms for image analysis [14,16]. All three are dependent on the development of plug-ins for specific applications from its user-base. OMERO currently does not have automated algorithms for image segmentation, and is dependent on user input for the delineation of cell boundaries or other regions of interest. ImageJ and Icy have a series of plugins for nuclear segmentation, membrane segmentation and spot detection, however these three processes are often disjoint and will require user effort to collate these results. We have used MATLAB as the platform for developing GoIFISH due to its wide user-base, strong image analysis capabilities and comprehensive data analysis features.

Softwares with specific cell segmentation capabilities include Columbus, CellProfiler [13], ImageM [18] and CellTracker [17]. CellProfiler and Columbus are programs which specialize in the segmentation of cells from the cytoplasmic and nuclear level, down to the subcellular or genomic level. These have been developed primarily for high-throughput analysis of cells in culture, and often are based on assumptions about the regularity of size and morphology within the cell population of interest. These softwares are optimized to have minimal segmentation errors in cell-culture images, however, may not be directly applicable to real tissue.

ImageM [18] is a software developed for detection and counting of RNA signals using a semi-automated approach. Users can refine results, however, to extract features on a per nucleus or cell basis, manual delineation of regions of interest is required. CellTracker [17] has been developed primarily for the live-tracking of cells, and a semi-automated approach to nuclear and cytoplasmic segmentation is also applied.

**Figure 1:**
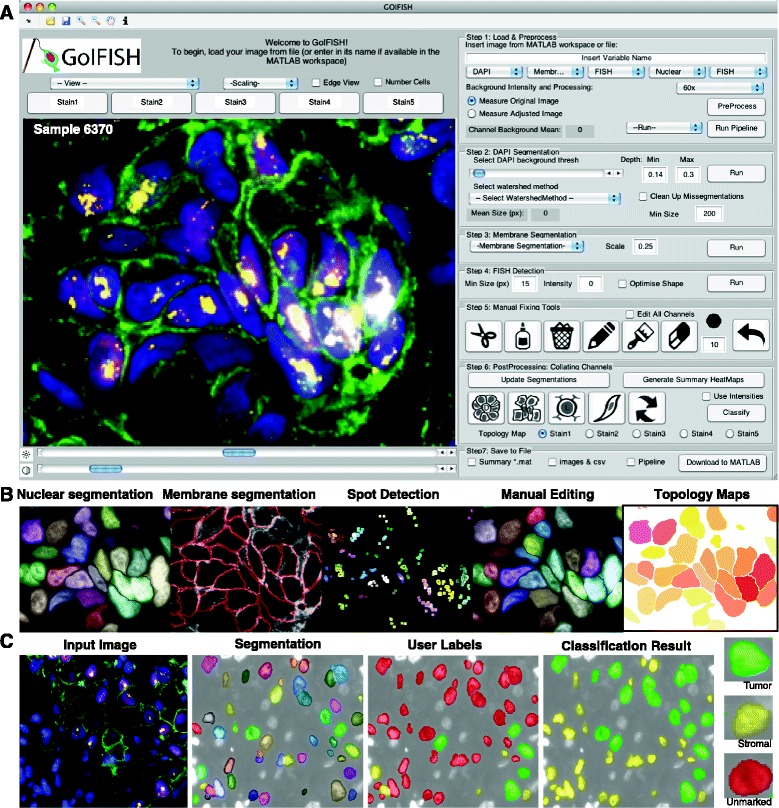
**Overview of** **GoIFISH**, **including computational capabilities.** **(A)** The GoIFISH graphical user interface **(B)** Outputs from GoIFISH including nuclear and membrane segmentation, spot detection, manually edited segmentation, and topology maps **(C)** Cell classification. After cell segmentation, cells are labelled as tumor (green) or stromal (yellow) by the user. Cells are automatically classified using the marked cells as training data.

Currently available segmentation softwares are not tailored for the segmentation of IFISH images, due to the heterogeneous nature of tumor populations and complex tissue structure. This has motivated the development of GoIFISH to perform accurate nuclear, membrane and spot detection, while allowing the user the freedom to manually edit segmentation outputs (Figure 1). This is critical as a starting point for the analysis of tumor subpopulations, and the extraction of biologically relevant features from the images.

## Availability of GoIFISH

GoIFISH is freely available at www.sourceforge.net/projects/goifish/ under the GNU General Public License version 2. GoIFISH is written in MATLAB and all source code is provided to allow analysis on both command line and through the Graphical User Interface (GUI) (Additional file 1). It is dependent on OMERO Bio-formats for the conversion of images to the correct file format for loading [19]. All images used in this study are available at the given link. The GUI has been created into a stand-alone program operable on Windows and Mac OS systems following the installation of the appropriate version of MATLAB Compiler Runtime (v7.14), provided at the given link.

The user-guide for the software is included as Additional file 2. The types of images for optimal use in GoIFISH are described in Table 2. Note that due to memory constraints, there are size limitations of 12 megapixels for comfortable use in the GUI however, this limitation can be overcome using the GoIFISHWrapper in the MATLAB environment.

**Table 2:** **Optimal properties of images for analysis in** **GoIFISH**

	**GUI**	**GoIFISHWrapper (Command Line)**
File Format	.mat or.tiff	.tiff.zvi Use bio-formats to convert other formats
Image Size	12 megapixels for comfortable use	Theoretically unlimited
Number of stains	Up to 5. Must include DAPI	Unlimited but must include DAPI
Number of Cells	Under 1000 for comfortable use	Theoretically unlimited
Cell Size (px)	Optimally 60x magnification (2500-10000 px), 20x and 40x magnification	also available (250-3000 px)

## The GoIFISH workflow

The following paragraphs give a detailed overview of the GoIFISH workflow. An overview of the capabilities of GoIFISH, including the user interface is shown in Figure 1.

**Step 1: Loading and preprocessing data**Images can be loaded as a.mat file containing a cell array or a.tiff file into the GUI. After successful loading, the first image in the series will be presented (Figure 1A). These are automatically adjusted to ensure 1% of the image is saturated at lower and higher intensities, which is more suited for nuclear or cytoplasmic images but may saturate spots. The ‘RangeScale’ option is recommended in these scenarios. Brightness and contrast of each image can be adjusted for auto-fluorescence, to ensure an optimal dynamic range and to prevent saturation of the image. This is critical for good segmentation results. Note that the image adjustment will improve the user experience and segmentation results, however, the intensities can be measured from the raw image for comparative quantitation.

GoIFISH allows background intensity adjustment, both using a single global intensity value, or on a cell specific level for nuclei. The user draws ‘background’ regions using the paintbrush tool, from which the mean background intensity is calculated. This is subtracted from either raw intensities, which is recommended for comparisons between samples, or from an adjusted image. A per cell nuclear background adjustment is also available, whereby a background intensity is calculated at a margin of 2-6 pixels from the edge of each segmented nucleus. This will be computed automatically for all nuclear stains, and will account for local variation in background intensity.

To finalize the preprocessing stage the user will need to indicate the stain type in each channel, of which one must be DAPI, and the magnification of the image. 60x, 40x and 20x magnifications are permitted, however higher resolutions are recommended for the accurate detection of spots. Following this, a quick preprocessing step is applied to assign the stain type to each image. After this, segmentations on either the DAPI channel alone or all channels using default parameters can be performed (Figure 1B).

**Step 2: Nuclear segmentation** The foreground or cellular portion of the image is automatically detected by Otsu thresholding [20] on a combined image of entropy and intensity, which is used as a mask in cell segmentation. Nuclei are segmented using an iterative H-minima transformed watershed [21], where the local intensity depth of pixels under a given threshold is suppressed, and a watershed is applied. Fragments attained from each step are classified as either optimal, undersegmented, or oversegmented. Cells with optimal properties are selected, and the remaining image is subjected to segmentation at a lower threshold. We have developed an approach to mitigate oversegmentation by joining neighboring fragments according to their morphological features (see Material and methods). There is also an option of performing a seeded-watershed [22] for images with a small number of cells or poor contrast at cell boundaries, whereby the user indicates the cell locations with a series of spots. A H-minima watershed is then applied using this information.

**Step 3: Membrane segmentation** Following segmentation, the user has the option of narrowing down the population of interest using a minimum size threshold, or edit the borders manually (see Step 5). It is recommended that the user checks the output of the nuclear segmentation before proceeding as membrane segmentation and spot detection are dependent on the nuclear map generated.

Membrane segmentation is performed by combining a Voronoi segmentation of nuclei with the intensity information of the image. High intensity edges within the image are set as local maxima to ensure segmentation occurs along these edges. Fragments are then merged based on their location with respect to the nuclear segmentation. This result can be further refined using active contours, such as Chan-Vese Segmentation [23] or Localized Segmentation [24].

**Step 4: FISH detection** For single spot detection, a Laplacian of Gaussian filter is applied to the image to determine candidate spots. Spots which are also local maxima in the gradient image are selected. The user has an option of entering an expected size threshold (Default minimum spot size of 15 pixels for 60x images), and a preferred intensity threshold. An optional morphology classifier (see Material and methods) can be applied to differentiate between spots and artefacts in images with low contrast signal, such as signals from centromere 17.

**Step 5: Manually editing segmentations**GoIFISH provides a toolbox to manually edit segmentations if needed. Manual editing of the segmentation output can be easily achieved by drawing a border between cells with the ‘scissors’ tool, oversegmented cells can be ‘glued’ together, and artefacts can be ‘trashed’. Regions can be ‘painted’ or ‘erased’. All operations are terminated by right clicking, and pressing the ‘escape’ exits a particular editing mode.

**Step 6: Post segmentation processing**Segments from each channel need to be mapped to the DAPI channel in order to construct a matrix of features, using the ‘Update’ function. An error will appear if there are inconsistencies in the segmentation, such as two nuclei mapping to one membrane. Following successful mapping, the user can generate heatmaps and topology maps to visualize staining variations within the image (Figure 1B) and perform cell classification (Figure 1C).

Cells in the image are classified by support vector machine into 4 possible cell types. The user labels candidate cells for each class (for example, to separate fibroblasts from tumor cells), and applies the classification. This classification is based on morphological parameters but can also include intensity information. The classification result can be manually corrected if inconsistencies appear.

**Step 7: Output from GoIFISH**The output from GoIFISH can be saved as a series of images and a.csv file with cell specific measurements. These include intensity measurements, including raw and background adjusted intensities, morphologial parameters such as area, perimeters, axis lengths, the location of the centroid of each nuclei, the cell label if classification is performed and copy numbers in the case of spot detection. Data can be downloaded into the MATLAB environment, or saved as a progress.mat file where processing can be resumed in another session.

**GoIFISHWrapper: Combining Steps 1-4**GoIFISH provides a wrapper for batch analysis, which is implemented via command line. The user simply provides the filepath of interest and edits a Parameter File which contains information such as the stains used, the magnification of the image and the segmentation parameters. The results are automatically saved as progress.mat files which can be loaded into the GUI for segmentation editing, background selection and cell labelling. Other benefits of running the wrapper include unlimited number of stains and the analysis of larger images. However, it is recommended that each image does not exceed a resolution of 12 megapixels if the user wishes to edit cells in the GUI. In this circumstance, it is recommended that the image is sectioned into a number of smaller constituent images which are analyzed independently.

## GoIFISH performance and benchmarking

The performance of GoIFISH was compared to two state-of-the-art image analysis systems, the proprietary Columbus software from Perkin-Elmer, and the open-source CellProfiler from the Broad Institute [13]. All three image analysis softwares can detect nuclei, FISH signals, cytoplasm staining, and report morphological properties including size, and intensities. Details of all statistical analyses including code to reproduce plots are contained in the Additional file 3.

### Comparing **GoIFISH** to existing automated methods

Columbus has an intuitive interface, real-time feedback, automatic detection of approximate cell sizes, with very little image processing knowledge required to operate the system (Table 1). On the other hand, CellProfiler has the benefits of wider functionality as its open-source nature allows its user base to develop and maintain specialized functions. However, it requires a-priori knowledge about the images and different segmentation methods, which may take the user a long time to develop an optimal segmentation pipeline. The parameters used to segment images in these two programs are described in Material and methods.

GoIFISH was tested in two scenarios. In order to perform fair benchmarking, images from 10 samples were run in GoIFISH on the default settings (see Material and methods). In addition, we tested its capabilities for improvement with user input.

**Figure 2:**
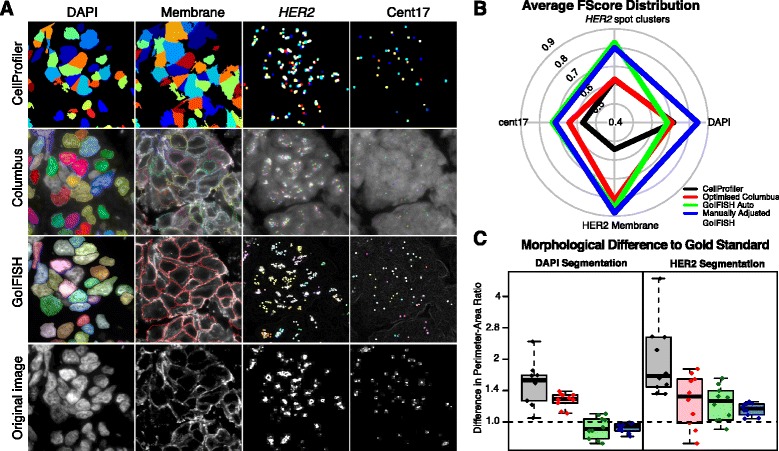
**Performance benchmarking against Columbus and CellProfiler.** **(A)** Example of nuclear, membrane and spot segmentation using CellProfiler, Columbus, and GoIFISH manually corrected segmentations. **(B)** Average F-Scores for nuclear segmentation, membrane detection, spot detection (including clusters) in 10 sample images. **(C)** Differences in perimeter-area ratio of segmented nuclei and membranes compared to the gold standard. Values closer to 1 indicate similar morphology to the gold standard.

An example of the segmentation output from all three softwares is shown in Figure 2A. GoIFISH, using its default segmentation parameters, demonstrated precision and recall in nuclear and spot detection which are comparable to both Columbus and CellProfiler (Figure 2B, Additional file 4: Figure S1B, Mean F-Score: 0.68,*N*=10). From visual inspection of the segmentation output (Figure 2A), Columbus does not perform nuclear segmentation when the borders are not well defined. CellProfiler applies edges to ensure segments are within the defined range of cell diameters, resulting in segmentation inaccuracies. GoIFISH with default parameters outperformed existing softwares in membrane detection (Figure 2B, Mean F-Score 0.86, N=10) with results very similar to the manually edited GoIFISH result. All methods demonstrated high precision as membranes are detected around nuclei, but varying degrees of recall (Additional file 4: Figure S1B). We observed that Columbus treats membranes with high intensity as cytoplasmic regions (Figure 2A).

In Cent17 and *HER2* detection GoIFISH with default parameters surpassed the two existing methods (Mean F-Scores: 0.69, 0.83 for cent17 and *HER2* respectively). Columbus has high recall but poor precision, reflecting a higher false positive rate. It should be noted that the manually curated samples had variable F-score, which may be a subsequent propagation error from nuclear segmentation.

While precision and recall assess the presence of an object, they provide no information on how accurate the morphology of the segmentation is. For instance, encroachment errors with slight misplacement of cell boundaries will have no effect on the F-Score. Therefore, the perimeter-area ratio was measured for each cell and compared to the gold standard as an assessment of whether the correct shape was detected. Each individual spot in Figure 2C represents the average difference in perimeter-area ratio for one particular image. Points centred around 1 indicate very little variation in shape compared to the gold standard. In both nuclear and membrane segmentation, GoIFISH values were closer to 1 compared to Columbus and CellProfiler, indicating that the morphology was better represented.

Time-benchmarking was performed between GoIFISH and the newest version of CellProfiler (v2.1.0) (Table 3). Columbus operates on a server and thus a direct comparison was not applicable. Timings were performed on a 2x2.4 GHz Quad-Core Intel Xeon processor with 6GB RAM, on 5 candidate samples with an increasing number of cells. GoIFISH performs comparably to CellProfiler when the image contains well defined cells, but the processing time increased with greater image complexity. For example, processing image 6370 took the longest as it has a high number of cells with invasive phenotype and poorly defined cell boundaries. All segmentations can be conducted within 1.2 minutes, which despite being longer than CellProfiler, is still sufficiently short for mainstream use.

**Table 3:** **Timing comparisons between** GoIFISH **and CellProfiler**

**Sample**	**7461**	**6361**	**7435**	**7619**	**6370**
Approximate Number of Cells	20	60	80	100	120
CellProfiler Time (s)	26	25	29	30	32
GoIFISH Time (s)	26	27	40	42	73

### Correlating **GoIFISH** output with visual interpretation

While precision-recall testing allows reliable assessment of segmentation accuracy, the obtained data must be reflective of the biology to draw valid conclusions. Automated scoring of protein intensities and spot areas were compared with visual pathologist scoring on a single-cell level, with a total of 355 cells scored for ER staining, membrane HER2 intensity and cent17 and *HER2* copy number.

**Figure 3:**
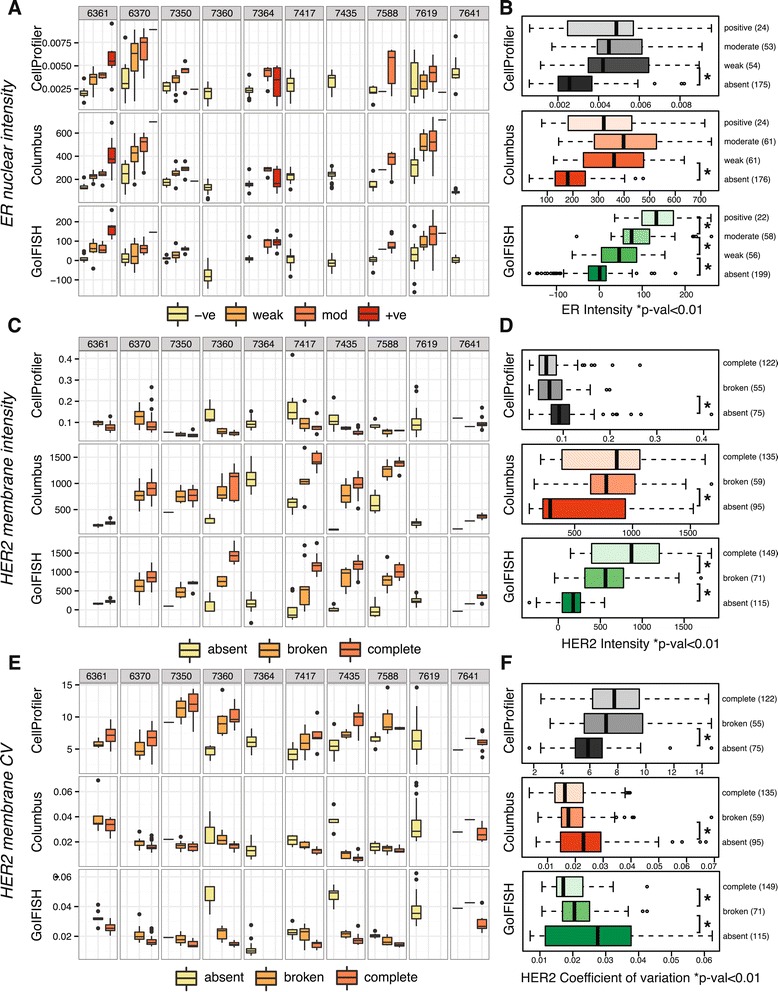
**Correlation between measured intensities and classification by a pathologist.** Comparison of semi-quantitative scoring by a pathologist with CellProfiler, Columbus and GoIFISH quantitation in **(A)** ER staining, **(C)** HER2 membrane completeness using mean intensities **(E)** HER2 membrane intensity using coefficient of variance. Combined distributions of quantitations across all 10 samples for **(B)** ER positivity, **(D)** HER2 membrane completeness using mean intensities and **(F)** HER2 membrane intensity using coefficient of variance. Note that GoIFISH nuclear adjusted intensities were used in **(A, B)**, GoIFISH background adjusted intensities were used in **(C, D)** and GoIFISH raw intensities used in **(E, F)**.

In a first analysis, we assessed immunofluorescence of ER and HER2 to determine (1) whether the distribution of cell intensities within an image is reflective of the semi-quantitative scoring by a trained observer and (2) whether two cells with the same scoring in two different images are directly comparable. All methods detecting the nuclear ER stain showed a correlation between the semi-quantitative scoring and intensity measurements, however, samples were not shown to be directly comparable to each other. As an example, a ‘positive’ cell in sample 6361 was on similar intensity to a ‘moderately’ stained cell in sample 6370 (Figure 3A).

ANOVA in conjunction with Tukey’s range test was performed on samples with ‘negative’ expression to determine whether the baseline means are directly comparable to each other. Out of the 45 possible pairwise comparisons, Columbus had 26 pairwise comparisons, CellProfiler had 13 and GoIFISH had only 10 pairwise comparisons which showed a significant difference in baseline mean (*p*<0.05). Sample 7360 had a negative intensity after background correction using GoIFISH, but in practice would be assigned a value of 0 which would further lower the number of significant differences. Using a per nucleus specific background subtraction method in GoIFISH, ‘positive’ samples become comparable to each other across all images (Figures 3A, Additional file 5: Figure S2A). Figure 3B illustrates the right ordering and statistical difference between each class, demonstrating that the method can reproduce quantitatively the visual scoring (T-test, *p*<0.01 between all categories).

The same analysis was applied to HER2 membrane staining to determine whether the intensity could recapitulate the membrane completeness in cells. HER2 protein assessment in a clinical setting often uses patterns of staining to guide subsequent treatment [25]. Cells are classified as having ‘negative’ membrane staining, ‘complete’ positive staining or ‘incomplete’ positive staining. Both GoIFISH after background subtraction and Columbus demonstrated a step-wise increase in intensity with highest intensity observed in complete membranes (Figure 3C,D). Combining all cells, a statistical difference at the correct order of classes was observed in only the GoIFISH background adjusted and manually edited samples (Figure 3D, Additional file 5: Figure S2D).

The coefficient of variation was also computed as a second metric of differentiating between ‘complete’ and ‘incomplete’ membranes. It is expected that ‘complete’ membranes have a lower variation than ‘incomplete’ membranes. In both GoIFISH and Columbus, an increased coefficient was observed in the samples with broken membranes compared to the samples with complete membranes, this was however only statistically significant in GoIFISH but not in Columbus (Figure 3E,F, Additional file 5: Figure S2C,D).

**Figure 4:**
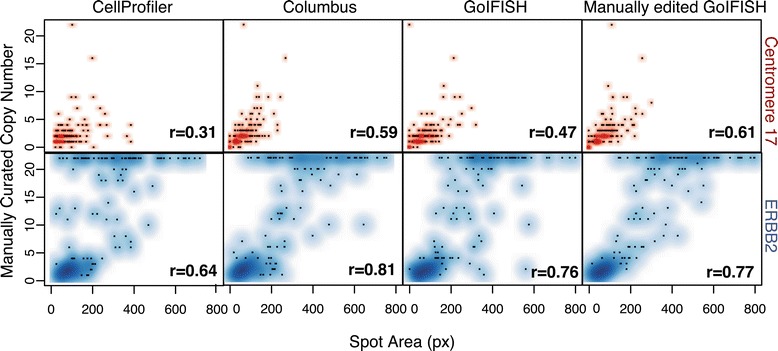
**Correlating copy number with spot area at a single cell level.** Correlation between manually counted spots and automated detection of spot area using Columbus, CellProfiler and GoIFISH. A linear relationship between copy number and area is observed until 21 spots per cell, after which individual spots are no longer discernable by eye. Darker regions indicate a higher population of cells with similar properties.

In a second analysis, we assessed how accurately an automated system can estimate the number of spots within each cell based on the area measured. In 9 samples (Sample 7619 was excluded due to overexposure of the channel and thus high false positive rate), we assessed the correlation between manually counted copy number per cell with the automatically detected spot area (Figure 4). In samples where the distinct number of copies cannot be counted due to amplification, a value of 22 was assigned (which was one more than the upper observable limit of 21 spots). Most relationships appeared to be linear for centromere 17, with the exception of measurements by CellProfiler. The estimates of *HER2* spots by GoIFISH and Columbus showed a strong linear correlation with manually curated copy numbers, which plateaued at a value of approximately 400 pixels. Similar gradients were shown for centromeric and *HER2* spots, and in both cases a linear regression will approximate an area of 20 pixels per spot.

### Effects of user variability on **GoIFISH** outputs

User input may influence background intensity correction, cell segmentation results and cell classification. GoIFISH has been developed with a number of strategies to minimize the effects of inter-user variation.

**Figure 5:**
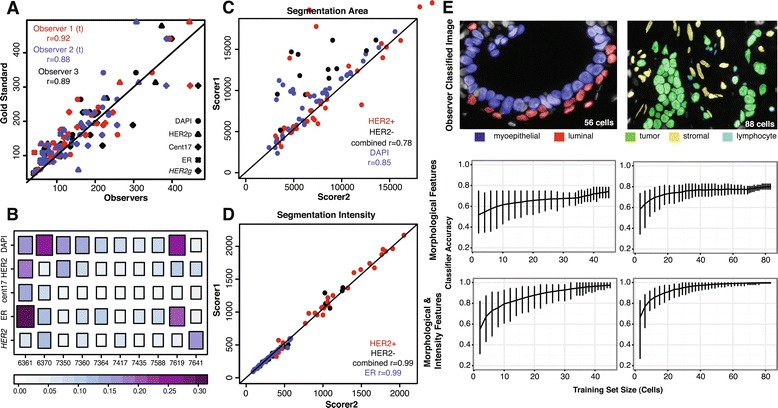
**Robustness of** GoIFISH **performance with user input.** **(A)** Inter-user variation in background intensity selection from three observers (two trained) when compared to the Gold Standard. **(B)** Heatmap of coefficient of variation of background intensity in all sample images. Larger shape indicates a higher mean. **(C)** Inter-user variation in area of segmented cells (nuclear and membrane) and **(D)** corresponding intensity of HER2 membranes and ER. **(E)** Permutation testing of accuracy of classifier using either only morphological information or morphological and intensity information for myopepithelial-luminal discrimination (left) and lymphocyte-stromal-tumor discrimination (right).

To determine the variation in background selection between users, measurements were made by two trained observers and one untrained observer who was given background selection-guidelines (see User Guide in Additional file 2). All three observers demonstrated 88% or greater correlation with the gold standard (Figure 5A), demonstrating that our guidelines are sufficient for an ‘untrained observer’ to attain a similar scoring as a ‘trained observer’.

To address the issue of background heterogeneity within an image, a trained observer selected four different background regions within each image to compare intensities. These values are reported as coefficients of variation (Figure 5B), where the size of each box proportional to the mean reported intensity. In most images, a low variation of 10% or less was observed, with the exception of 6361 and 7916 which had high auto-fluorescence and overexposure respectively. The greatest variation was observed in the DAPI channel, which is a general DNA marker used to assess the quality of the sample. In practice, DAPI intensities are rarely measured for quantitative analysis. The other stains are more selective and specific for a particular protein or locus of interest, and have demonstrated greater stability in background intensities.

Manual editing of segmentation results is also prone to user subjectivity. To reduce both this effect and manual labor, GoIFISH was designed with a toolbox which minimizes the amount of clicks or mouse-drawing performed by the user. For example, the merging of cells requires two clicks of the mouse, and the segmentation of overlapping cells requires one line to be drawn. These features ensure that the morphology and boundary of cells are consistent in each image irrespective of the user.

To determine the effectiveness of these tools, two independent scorers manually edited 50 missegmented cells across the 10 test images. The nuclear and cytoplasmic areas were measured, alongside nuclear ER intensity and HER2 membrane intensity. Nuclear segmentation was consistent between the two scorers (*r*=0.86, Figure 5C), however a number of cells were considered to be larger by Scorer 1 than Scorer 2. The discrepant cells were determined to be mitotic, phenotypically characterised by the appearance of two nuclei in the DAPI channel yet sharing the same membrane in the HER2 channel, and considered as one cell by Scorer 1 but as two cells by Scorer 2. The cyotplasmic areas had lower correlation between the two observers (*r*=0.79), which can be attributed to the HER2 status of the cell. In the absence of a well defined HER2 membrane, the shape is open to interpretation, accounting for the greater variation in the HER2- cells than the HER2+ cells (HER2+ only: *r*=0.83).

Despite the differences in morphology of the segmented cells, 99% correlation was observed in the raw recorded intensities (*r*=0.99 for both ER and HER2, Figure 5D), demonstrating that intensity measurements are robust to differences in cell segmentation between users.

Finally, we tested how the performance of the cell classifier depends on the size of the training set and cellular features in two different cell classification scenarios: (1) to differentiate myoepithelial from luminal cells and (2) to differentiate lymphocytes and stroma from tumor (Figure 5E). Two images representing these two scenarios were labelled by a trained observer. Training sets of increasing size (starting from 2 cells) were created by randomly sampling the number of required cells, and where possible an equal number of cells from each class were selected. To determine the 95% confidence interval for classifier accuracy, 500 permutations of the training set for each size were used to predict the labels within an image. Our results demonstrate that using morphological parameters alone, the accuracy approaches 70% for myoepithalial-luminal discrimination, and 80% for lymphocyte-stromal-tumor discrimination. With the addition of stain information, the accuracy approached 95% and 100% accuracy respectively. The average accuracy of the classifier increases with a larger number of labelled cells, however, if well-chosen, high classification accuracy can still be attained with a training set of under 10 cells.

### Visualizing the cellular diversity within an image

**Figure 6:**
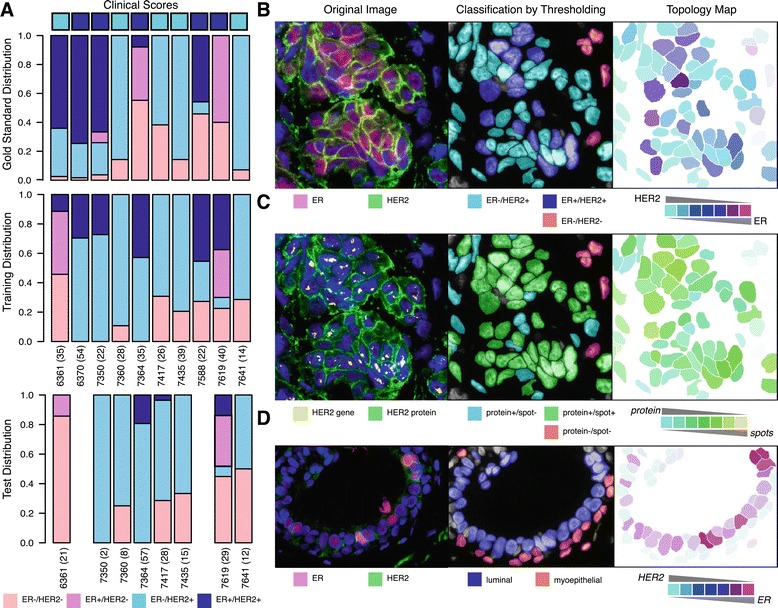
**Visualizing cellular heterogeneity.** **(A)** Comparison of cellular diversity in IFISH images by applying a global intensity threshold of 300 for HER2 and 50 for ER on the gold standard cell distribution, the training distribution and test set. **(B)** Classification of cells in Sample 6370 based on HER2 and ER intensity and the corresponding topology map. The color indicates the relative ratio between the two stains. **(C)** Classification and topology map applied to HER2 protein expression (cyan) and *HER2* spot area (yellow). **(D)** Classification of cells in Sample 6361 into luminal or myoepithelial cells based on morphology, and topology map based on HER2 and ER staining.

Finally, we visualized the cellular diversity within the images, first by looking at IF staining to compare HER2 and ER intensities and also by comparing genomic expression with protein expression of HER2 (Figure 6). A global intensity cut-off of 50 for ER after nuclear adjustment, and 300 for background adjusted HER2 staining was implemented to define cells as positive or negative. These values were chosen from global assessment of the distribution of staining across all 10 samples (Additional file 5: Figure S2B,D). These cutoffs yield 4 classes as shown in Figure 6A. This procedure was applied to both the training set of cells, which were scored by a pathologist, and the remaining cells within the same image, which formed the test set.

Both the training and test set showed resemblance, however samples 6361 and 7619 differed from the gold standard distribution. These differences may be due to the arbitrary setting of one intensity threshold, and the subjectivity in visual scoring where background intensities between samples are seldom taken into consideration.

Figure 6B illustrates the spatial distribution of the classified cells in sample 6370 and the corresponding topology map which displays the relative ratio between HER2 and ER. Most of the cells which were considered as double negative are identified morphologically as stroma, and display low intensity in both proteins. Cells which were classified as double positive demonstrated subtle cell-to-cell variations which would not otherwise be observable with a strict threshold. The same analysis was applied to HER2 protein and *HER2* spots, with a cutoff placed at *HER2* spot area of 60 pixels which is roughly equivalent to 3 spots. Most cells exhibited a HER2 protein intensity increase with a spot area increase (shown in green), however, a subset with high expression of HER2 but a relatively lower spot area was also present.

The scoring of cells is also dependent on the tumor region selected for analysis (Figure 6D). Sample 6361 was considered to be clinically HER2+, however a ductal carcinoma *in situ* rather than an invasive component of the tumor was selected for this analysis. As a result, weak HER2 and ER intensities were attained, as shown in the topology map, compared to sample 6370.

## Conclusions

**Segmentation of complex tissue components improves with manual correction**There are many challenges with *in situ* analysis of molecular features in tissue sections. Tissues display complex compositions of organic structures such as epithelial elements, vasculature, lymphatic components, nerves and supportive tissue including different types of fibers. There is morphological diversity between cell types and their organization, and in cancer this is even more pronounced. For automatized image analysis, accurate segmentation of cellular components is crucial to avoid misleading estimates of markers. Overlapping or closely spaced nuclei can easily be interpreted as one, and cells with major sectioning artefacts need to be discarded.

Many automatized approaches have been developed to address these issues, and Columbus and CellProfiler are two state of the art softwares used to benchmark the performance of GoIFISH. These have been developed for general applicability to a number of biological scenarios, of which cell-based culture is their main strength. As a result, the application to tissue-sections with the heterogeneous morphology may have resulted in the poorer results observed. In particular, CellProfiler did not perform at a similar level as Columbus and GoIFISH: this may be attributed to the need to optimize parameters in a segmentation pipeline before batch processing, whereas both Columbus and GoIFISH automatically calculates parameters for each image. In addition, GoIFISH provides user-friendly options to manually correct inaccurate segmentations and remove artefacts. As shown in Figures 2A and Additional file 4: Figure S1B, this correction step is crucial for improving precision and recall.

Inter-user subjectivity is a large issue in the field of pathology and there is potential of introducing user bias in the background selection and segmentation editing steps. To mitigate this, our editing toolbox is designed to minimize the amount of manual cell-outlining required, and guidelines have been included in the user-manual for background selection. We have demonstrated that these measures are effective in minimizing inter-user variation, with similar intensity measurements for both background and cell staining reported by different scorers (Figure 5).

**Image analysis is dependent on image quality**The quality of the segmentation and marker recognition is highly dependent on the quality of the samples attained. For formalin-fixated paraffin-embedded tissue sections, there are variables including fixation type, fixation duration and tissue processing that differ from patient to patient and between laboratories [26]. For fluorescence analysis in general, some tissue composites induce more autofluorescence than others making “true" staining difficult to quantify. Tissue sections from patient samples have preprocessing steps which cannot be controlled for at the same level of precision as fixation of cell lines can. This explains the variation in sample-to-sample fluorescence intensities as illustrated in Figure 3, 5B, 6B, despite imaging all sections under similar conditions.

The images used in this study were of high quality but still exhibited artefacts that confounded segmentation results, which is reflective of the challenges faced in image acquisition and analysis. Low intensity of a spot marker compared to the background was observed in sample 6361, resulting in poor detection using all three methods. Overexposure of a channel will increase the false positive rate, as seen in image 7619, and the presence of background artefacts in the DAPI channel will affect segmentation accuracy, as seen in sample 7350. Background adjustment, manual editing and the application of classifiers are strategies GoIFISH uses to address these issues. GoIFISH allows users to select background regions to ensure baseline intensities are comparable across samples, and performs per nucleus background adjustment to remove local variations in auto-fluorescence. This is necessary for comparability of cells across samples. To assist in accurate segmentation, a morphological classifier was applied to centromere 17 detection to remove confounding effects. In addition, manual user input in GoIFISH rectified most difficulties encountered during segmentation.

**Contribution of the software on measuring intra-tumor heterogeneity**Accurate segmentation on the nuclear, membrane and spot level are essential for the extraction of biologically meaningful features from cells. GoIFISH has demonstrated comparable segmentation to CellProfiler and Columbus in nuclear segmentation, and has outperformed them in membrane and spot detection. GoIFISH is capable of segmenting membranes when weakly positive or incomplete, allowing for subsequent objective analysis of intensity-based features.

To address the complexity of tissue composition and its impact on prognosis [27] we have also included a cell-type classifier based on morphology and intensity. By marking a few segmented cells, all cells with a similar morphology are identified with high accuracy, particularly if intensity information is also included. To illustrate the importance of quantitative analysis in the correct cellular context, we have included a pre-invasive part of a tumor in our analysis (Sample 6361). As shown in Figure 6, the clinically reported HER2 positivity was not detected. These cells are luminal epithelial and myoepithelial cells, rather than invasive neoplastic cells. In downstream analyses, the added categorical knowledge will ensure these would not be directly compared to invasive cells. The extraction of features from a sample can be multi-dimensional, making visualization of heterogeneity a difficult task. We have included simple topology maps that overlap two stains of interest, allowing visualization of both heterogeneity across cells and their spatial relationships.

**Summary**GoIFISH has been developed to segment high magnification images with combined genomic and phenotypic traits, combining the analysis of nuclei, membranes and spots into a single easy-to-operate system. Thus, GoIFISH allows the objective quantification of the morphological, genomic and phenotypic heterogeneity often observed in tumor IFISH images. Application of quantitative approaches like GoIFISH on large sample collections will lead to profound insights into the impact tumor heterogeneity has on disease progression, and may uncover evolutionary pathways explaining the development of resistance.

## Material and methods

### A sample set of HER2 positive breast cancers

This study was conducted in compliance with the Declaration of Helsinki and was approved by the regional ethics committee (REK S-06495b).

Human tissue samples were collected following protocols approved by the institutional review board of Oslo University Hospital Radiumhospitalet (IRB 2006-53). We used 10 formalin-fixed paraffin-embedded (FFPE) primary tumors from HER2+ breast cancer patients. In this work, we performed IFISH by combining the immunodetection of HER2 protein (expressed in the cell membrane) and Estrogen Receptor *α* (ER *α*, located in the nuclei) with the detection of *HER2* and centromere 17 (cent17) copy number, following a protocol previously described [10]. FFPE samples were dewaxed and hydrated in series of ethanol. Heat-induced antigen retrieval was performed in citrate buffer (pH 6) followed by pepsin digestion. After the immunostaining of HER2 and ER at room temperature in a humidifier, tissue slides were hybridized with *HER2* and centromere 17 probes at 37C°. overnight. Post wash was carried out in SSC (saline-sodium citrate) buffers with different stringency, before air drying and mounting media with DAPI was added. Image acquisition was carried out in an epifluorescence microscope. One randomly selected area per tumor was photographed in a Zeiss Axioplan 2 microscope equipped with an Axio Cam MRM CCD camera and Axio Vision software. The experimental methods are explained in greater detail in the Additional file 3.

### Analysis pipelines for CellProfiler, Columbus and**GoIFISH**

Columbus provides 4 nuclear, 4 cytoplasmic and 3 spot detection methods. These were first tested visually to determine the best candidate methods, which were then quantitatively compared with CellProfiler and GoIFISH in terms of precision and recall. The best results from each image were then used for direct comparison (see Additional file 3).

A CellProfiler analysis pipeline was constructed with the following parameters: Nuclei Segmentation was performed using two class Otsu Global Thresholding, and diameter of objects restricted to 20-120 pixels. Clumped cells were separated using the propagation method. Membrane detection was performed based on the propagation method, using the combination of the distance to the nuclei and intensity gradient to select the membrane. The spot signals were enhanced and masked to nuclear regions. Spots were detected using ‘RobustBackgroundPerObject’, limited to a diameter between 5 and 40 pixels, with clumped objects separated based on intensity.

The default GoIFISH pipeline performed shape optimised nuclear segmentation with intensity suppression between 10 to 30% and fragments of size less than 500 pixels discarded. The output nuclear map was applied to HER2 membrane detection and spot detection. Detected Centromere 17 spots were run through a morphological classifier to minimise effects of autofluorescence.

### Metrics for performance evaluation

We compared computational approaches to a manually segmented ‘gold standard’. For each of the 10 images, nuclei, membranes and spots were outlined manually in the maximum projected image. Spots were counted through 15-21 z-stacks. In total, 355 individual cells were scored for membrane completeness, nuclear positivity and copy number. We then benchmarked the computational outputs of GoIFISH with the gold standard using the following panel of quality criteria. We define *N*_*t*_ as the number of correctly segmented cells, *N*_*under*_ and *N*_*over*_ as the number of under and over segmented cells, *N*_*FP*_ as the number of false positives which do not appear in the gold standard image, and *N*_*FN*_ as the number of false negatives (See Additional file 4: Figure S1A).

- Precision is defined as *P*=*N*_*t*_/(*N*_*t*_+*N*_*FP*_+*N*_*over*_)
- Recall: *R*=*N*_*t*_/(*N*_*t*_+*N*_*FN*_+*N*_*under*_)
- the F-Score is the harmonic mean of precision and recall: *F*=2*P*·*R*/(*P*+*R*), which is a measure of how well a cell can be detected, and whether the number of cells present closely resemble the true value.

### Mitigating oversegmentation

We have developed and implemented a technique to mitigate oversegmentation. For a fragment *f* classified as oversegmented, we list all neighboring fragments **g**={*g*_1_,…,*g*_*N*_} within a radius of *r* pixels. Each union *c*^*j*^=*f*∪**g**^*j*^ of *f* with a subset of neighboring fragments **g**^*j*^⊆**g** is a potential extension of *f* into a full cell. The index *j* ranges over all members of the powerset of **g**, i.e., the set of all its subsets. To select the best extension *c*^∗^, we first score all possible extensions *c*^*j*^ by a function *S*_*F*_(*c*^*j*^) that compares a feature *F* of the combined fragments to the average of that feature in the individual fragments:

(1) $$ S_{F}\left(c^{j}\right) = F\left(c^{j}\right) - \frac{1}{n_{j}} \sum\limits_{i=1}^{n_{j}} F\left({c^{j}_{i}}\right), \; \text{where } n_{j} = |c^{j}|,  $$

and then sum all feature-wise scores into a final score: $S\left (c^{j}\right) = \sum S_{F}\left (c^{j}\right)$, which we use to select the optimal extension *c*^∗^ as

(2) $$ c^{*} = \mathop{\text{argmax}}\limits_{j} \ S\left(c^{j}\right).  $$

Currently we use two morphological features for scoring fragments: Solidity and deviation from theoretical area. If **g**=*∅* or if there is no positive maximum of the score, then *c*^∗^=*f*.

### Classifiers

Classifiers have been implemented in a number of segmentation steps, including nuclei detection and spot detection, to minimise errors. GoIFISH only uses linear classifiers to reduce overfitting to data.

**Nuclear detection**Nuclei detection performs linear discriminant analysis based on a training set of 153 fragments from 5 images attained from nuclear segmentation performed at different depths. A total of 10 morphological properties including solidity, area, perimeter, axis lengths, axis ratios, circularity, area-perimeter ratio, and deviation from theoretical area and perimeter were measured. Each fragment was scored for oversegmentation, undersegmentation or optimal shape.

**Spot detection**A spot classifier was constructed using segmentation output from 2 training images containing 67 spot candidates and placed into a linear discriminant analysis. Features extracted for the classifier include solidity, area, perimeter, axis ratios, circularity, area-perimeter ratio deviation from theoretical area and perimeter, mean intensity and minimum intensity. Three classes were assigned to each ‘spot’: optimal, too small or too large.

**Cell classification**Classification of cells after segmentation is performed using a one vs all Support Vector Machine with linear kernel. Training data is generated on the spot from information supplied from the user. Features can either be morphological (area, perimeter, solidity, axis lengths and eccentricity) or contain extra information from the other channels, such as spot area or intensity.

## Additional files

Additional file 1
**GoIFISH** **software.** Source code for use directly in MATLAB. Binary files for use outside of MATLAB (For Mac and Windows OS) and the required MATLAB Compiler Runtime, are available for download at www.sourceforge.net/projects/goifish/ due to size constraints.

Additional file 2
**GoIFISH** **user manual.** A user guide for the operation of GoIFISH.

Additional file 3
**Supplementary information.** Supplementary information includes detailed experimental methods for the staining of images, and all code for the generation of plots and statistical analyses in R.

Additional file 4
**Figure S1.** Precision and recall in cell segmentation. (A) Illustration of the metrics used for Precision-Recall Testing (B) Precision Recall Plots for nuclear segmentation, HER membrane detection, centromere 17 spot detection and *HER2* cluster detection.

Additional file 5
**Figure S2.** Correlation between intensity and pathologist scoring. (A) ER staining intensity using GoIFISH raw, background adjusted and manually edited nuclear adjusted intensities compared to semi-quantitative pathologist scored ER intensity for each individual image. (B) Combined distribution of ER intensities across all samples using the methods described in (A) (C) Distribution of GoIFISH raw, GoIFISH manually edited mean HER2 intensities and GoIFISH manually edited coefficient of variation compared to pathologist scored membrane completeness for each individual image. (D) Distribution of HER2 intensities or coefficient of variation across all samples using the methods described in (C).

## References

[1] Almendro V, Marusyk A, Polyak K (2013). **Cellular heterogeneity and molecular evolution in cancer**. Annu Rev Pathol.

[2] Navin N, Kendall J, Troge J, Andrews P, Rodgers L, McIndoo J, Cook K, Stepansky A, Levy D, Esposito D, Muthuswamy L, Krasnitz A, McCombie WR, Hicks J, Wigler M (2011). **Tumour evolution inferred by single-cell sequencing**. Nature.

[3] Nik-Zainal S, Van Loo P, Wedge DC, Alexandrov LB, Greenman CD, Lau KW, Raine K, Jones D, Marshall J, Ramakrishna M, Shlien A, Cooke SL, Hinton J, Menzies A, Stebbings LA, Leroy C, Jia M, Rance R, Mudie LJ, Gamble SJ, Stephens PJ, McLaren S, Tarpey PS, Papaemmanuil E, Davies HR, Varela I, McBride DJ, Bignell GR, Leung K, Butler AP (2012). **The life history of 21 breast cancers**. Cell.

[4] Ding L, Ley TJ, Larson DE, Miller CA, Koboldt DC, Welch JS, Ritchey JK, Young MA, Lamprecht T, McLellan MD, McMichael JF, Wallis JW, Lu C, Shen D, Harris CC, Dooling DJ, Fulton RS, Fulton LL, Chen K, Schmidt H, Kalicki-Veizer J, Magrini VJ, Cook L, McGrath SD, Vickery TL, Wendl MC, Heath S, Watson MA, Link DC, Tomasson MH (2012). **Clonal evolution in relapsed acute myeloid leukaemia revealed by whole-genome sequencing**. Nature.

[5] Junker JP, van Oudenaarden A (2014). **Every cell is special: genome-wide studies add a new dimension to single-cell biology**. Cell.

[6] Widmer DS, Hoek KS, Cheng PF, Eichhoff OM, Biedermann T, Raaijmakers MIG, Hemmi S, Dummer R, Levesque MP (2013). **Hypoxia contributes to melanoma heterogeneity by triggering HIF1a-dependent phenotype switching**. J Invest Dermatol.

[7] Anderson ARA, Weaver AM, Cummings PT, Quaranta V (2006). **Tumor morphology and phenotypic evolution driven by selective pressure from the microenvironment**. Cell.

[8] Castaño Z, Marsh T, Tadipatri R, Kuznetsov HS, Al-Shahrour F, Paktinat M, Greene-Colozzi A, Nilsson B, Richardson AL, McAllister SS (2013). **Stromal EGF and igf-I together modulate plasticity of disseminated triple-negative breast tumors**. Cancer Discov.

[9] Merlo LMF, Pepper JW, Reid BJ, Maley CC (2006). **Cancer as an evolutionary and ecological process**. Nat Rev Cancer.

[10] Almendro V, Cheng YK, Randles A, Itzkovitz S, Marusyk A, Ametller E, Gonzalez-Farre X, Muñoz M, Russnes HG, Helland A, Rye IH, Borresen-Dale AL, Maruyama R, van Oudenaarden A, Dowsett M, Jones RL, Reis-Filho J, Gascon P, Gönen M, Michor F, Polyak K (2014). **Inference of tumor evolution during chemotherapy by computational modeling and in situ analysis of genetic and phenotypic cellular diversity**. Cell Rep.

[11] Almendro V, Kim HJ, Cheng YK, Gönen M, Itzkovitz S, Argani P, van Oudenaarden A, Sukumar S, Michor F, Polyak K (2014). **Genetic and phenotypic diversity in breast tumor metastases**. Cancer Res.

[12] Fuchs TJ, Buhmann JM (2011). **Computational pathology: challenges and promises for tissue analysis**. Comput Med Imaging Graph.

[13] Carpenter AE, Jones TR, Lamprecht MR, Clarke C, Kang IH, Friman O, Guertin DA, Chang JH, Lindquist RA, Moffat J, Golland P, Sabatini DM (2006). **CellProfiler: image analysis software for identifying and quantifying cell phenotypes**. Genome Biol.

[14] de Chaumont F, Dallongeville S, Chenouard N, Pop S, Provoost T, Meas-Yedid V, Pankajakshan P, Lecomte T, Le Montagner Y, Lagache T, Dufour A, Olivo-Marin JC, Hervé N (2012). **Icy: an open bioimage informatics platform for extended reproducible research**. Nat Methods.

[15] Johnston J, Nagaraja A, Hochheiser H, Goldberg I: **A flexible framework for Web interfaces to image databases: supporting user-defined ontologies and links to external databases**. *ISIB: IEEE*2006:1380–1383.

[16] Schneider CA, Rasband WS, Eliceiri KW (2012). **NIH Image to ImageJ: 25 years of image analysis**. Nat Methods.

[17] Wang Q, Niemi J, Tan CM, You L, West M (2010). **Image segmentation and dynamic lineage analysis in single-cell fluorescence microscopy**. Cytometry A.

[18] Lyubimova A, Itzkovitz S, Junker JP, Fan ZP, Wu X, van Oudenaarden A (2013). **Single-molecule mRNA detection and counting in mammalian tissue**. Nat Protoc.

[19] Linkert M, Rueden CT, Allan C, Burel JM, Moore W, Patterson A, Loranger B, Moore J, Neves C, Macdonald D, Tarkowska A, Sticco C, Hill E, Rossner M, Eliceiri KW, Swedlow JR (2010). **Metadata matters: access to image data in the real world**. J Cell Biol.

[20] Otsu N (1979). **A Threshold Selection Method from Gray-Level Histograms**. IEEE Trans Syst Man Cybernet.

[21] Soille P (1999). *Morphological image analysis: principles and applications*.

[22] Meyer F, Beucher S (1990). **Morphological segmentation**. J Vis Commun Image Represent.

[23] Chan TF, Vese LA (2001). **Active contours without edges**. IEEE Trans Image Process.

[24] Lankton S, Tannenbaum A (2008). **Localizing region-based active contours**. IEEE Trans Image Process.

[25] Wolff AC, Hammond MEH, Hicks DG, Dowsett M, McShane LM, Allison KH, Allred DC, Bartlett JMS, Bilous M, Fitzgibbons P, Hanna W, Jenkins RB, Mangu PB, Paik S, Perez EA, Press MF, Spears PA, Vance GH, Viale G, Hayes DF (2013). **Recommendations for human epidermal growth factor receptor 2 testing in breast cancer: American society of clinical oncology/College of American pathologists clinical practice guideline update**. J Clin OncolL: Official J Am Soc Clin Oncol.

[26] Gown AM (2008). **Current issues in ER and HER2 testing by IHC in breast cancer**. Mod Pathol.

[27] Yuan Y, Failmezger H, Rueda OM, Ali HR, Gräf S, Chin SF, Schwarz RF, Curtis C, Dunning MJ, Bardwell H, Johnson N, Doyle S, Turashvili G, Provenzano E, Aparicio S, Caldas C, Markowetz F (2012). **Quantitative image analysis of cellular heterogeneity in breast tumors complements genomic profiling**. Sci Transl Med.

