# Supplementary figures and images for: GoIFISH: a system for the quantification of single cell heterogeneity from IFISH images
Source: Genome Biol. 2014 Aug 26;15(8):442. doi: 10.1186/s13059-014-0442-y (PMC4167144; doi:10.1186/s13059-014-0442-y)

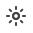

Supplement: Additional file 1 — GoIFISH software. Source code for use directly in MATLAB. Binary files for use outside of MATLAB (For Mac and Windows OS) and the required MATLAB Compiler Runtime, are available for download at www.sourceforge.net/projects/goifish/ due to size constraints. [file 13059_2014_442_MOESM1_ESM.zip › src/brightness.png]

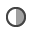

Supplement: Additional file 1 — GoIFISH software. Source code for use directly in MATLAB. Binary files for use outside of MATLAB (For Mac and Windows OS) and the required MATLAB Compiler Runtime, are available for download at www.sourceforge.net/projects/goifish/ due to size constraints. [file 13059_2014_442_MOESM1_ESM.zip › src/contrast.png]

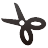

Supplement: Additional file 1 — GoIFISH software. Source code for use directly in MATLAB. Binary files for use outside of MATLAB (For Mac and Windows OS) and the required MATLAB Compiler Runtime, are available for download at www.sourceforge.net/projects/goifish/ due to size constraints. [file 13059_2014_442_MOESM1_ESM.zip › src/CutIcon.png]

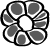

Supplement: Additional file 1 — GoIFISH software. Source code for use directly in MATLAB. Binary files for use outside of MATLAB (For Mac and Windows OS) and the required MATLAB Compiler Runtime, are available for download at www.sourceforge.net/projects/goifish/ due to size constraints. [file 13059_2014_442_MOESM1_ESM.zip › src/DCIS.png]

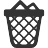

Supplement: Additional file 1 — GoIFISH software. Source code for use directly in MATLAB. Binary files for use outside of MATLAB (For Mac and Windows OS) and the required MATLAB Compiler Runtime, are available for download at www.sourceforge.net/projects/goifish/ due to size constraints. [file 13059_2014_442_MOESM1_ESM.zip › src/DeleteIcon.png]

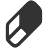

Supplement: Additional file 1 — GoIFISH software. Source code for use directly in MATLAB. Binary files for use outside of MATLAB (For Mac and Windows OS) and the required MATLAB Compiler Runtime, are available for download at www.sourceforge.net/projects/goifish/ due to size constraints. [file 13059_2014_442_MOESM1_ESM.zip › src/EraseIcon.png]

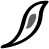

Supplement: Additional file 1 — GoIFISH software. Source code for use directly in MATLAB. Binary files for use outside of MATLAB (For Mac and Windows OS) and the required MATLAB Compiler Runtime, are available for download at www.sourceforge.net/projects/goifish/ due to size constraints. [file 13059_2014_442_MOESM1_ESM.zip › src/Fibroblast.png]

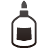

Supplement: Additional file 1 — GoIFISH software. Source code for use directly in MATLAB. Binary files for use outside of MATLAB (For Mac and Windows OS) and the required MATLAB Compiler Runtime, are available for download at www.sourceforge.net/projects/goifish/ due to size constraints. [file 13059_2014_442_MOESM1_ESM.zip › src/glueIcon.png]

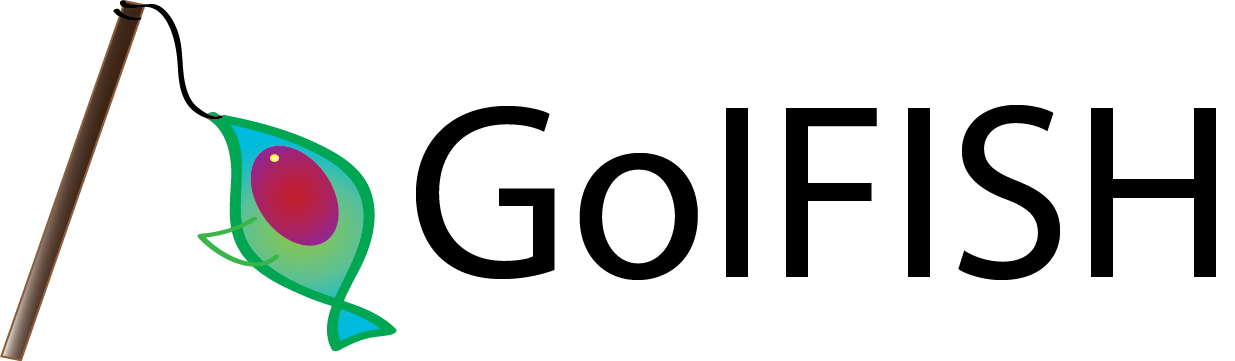

Supplement: Additional file 1 — GoIFISH software. Source code for use directly in MATLAB. Binary files for use outside of MATLAB (For Mac and Windows OS) and the required MATLAB Compiler Runtime, are available for download at www.sourceforge.net/projects/goifish/ due to size constraints. [file 13059_2014_442_MOESM1_ESM.zip › src/goifish.png]

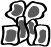

Supplement: Additional file 1 — GoIFISH software. Source code for use directly in MATLAB. Binary files for use outside of MATLAB (For Mac and Windows OS) and the required MATLAB Compiler Runtime, are available for download at www.sourceforge.net/projects/goifish/ due to size constraints. [file 13059_2014_442_MOESM1_ESM.zip › src/Invasive.png]

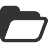

Supplement: Additional file 1 — GoIFISH software. Source code for use directly in MATLAB. Binary files for use outside of MATLAB (For Mac and Windows OS) and the required MATLAB Compiler Runtime, are available for download at www.sourceforge.net/projects/goifish/ due to size constraints. [file 13059_2014_442_MOESM1_ESM.zip › src/LoadIcon.png]

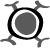

Supplement: Additional file 1 — GoIFISH software. Source code for use directly in MATLAB. Binary files for use outside of MATLAB (For Mac and Windows OS) and the required MATLAB Compiler Runtime, are available for download at www.sourceforge.net/projects/goifish/ due to size constraints. [file 13059_2014_442_MOESM1_ESM.zip › src/lymphocyte.png]

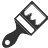

Supplement: Additional file 1 — GoIFISH software. Source code for use directly in MATLAB. Binary files for use outside of MATLAB (For Mac and Windows OS) and the required MATLAB Compiler Runtime, are available for download at www.sourceforge.net/projects/goifish/ due to size constraints. [file 13059_2014_442_MOESM1_ESM.zip › src/PaintIcon.png]

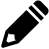

Supplement: Additional file 1 — GoIFISH software. Source code for use directly in MATLAB. Binary files for use outside of MATLAB (For Mac and Windows OS) and the required MATLAB Compiler Runtime, are available for download at www.sourceforge.net/projects/goifish/ due to size constraints. [file 13059_2014_442_MOESM1_ESM.zip › src/pencil.png]

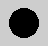

Supplement: Additional file 1 — GoIFISH software. Source code for use directly in MATLAB. Binary files for use outside of MATLAB (For Mac and Windows OS) and the required MATLAB Compiler Runtime, are available for download at www.sourceforge.net/projects/goifish/ due to size constraints. [file 13059_2014_442_MOESM1_ESM.zip › src/Pixel30.png]

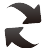

Supplement: Additional file 1 — GoIFISH software. Source code for use directly in MATLAB. Binary files for use outside of MATLAB (For Mac and Windows OS) and the required MATLAB Compiler Runtime, are available for download at www.sourceforge.net/projects/goifish/ due to size constraints. [file 13059_2014_442_MOESM1_ESM.zip › src/RefreshIcon.png]

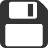

Supplement: Additional file 1 — GoIFISH software. Source code for use directly in MATLAB. Binary files for use outside of MATLAB (For Mac and Windows OS) and the required MATLAB Compiler Runtime, are available for download at www.sourceforge.net/projects/goifish/ due to size constraints. [file 13059_2014_442_MOESM1_ESM.zip › src/SaveFile.png]

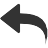

Supplement: Additional file 1 — GoIFISH software. Source code for use directly in MATLAB. Binary files for use outside of MATLAB (For Mac and Windows OS) and the required MATLAB Compiler Runtime, are available for download at www.sourceforge.net/projects/goifish/ due to size constraints. [file 13059_2014_442_MOESM1_ESM.zip › src/UndoIcon.png]

**A**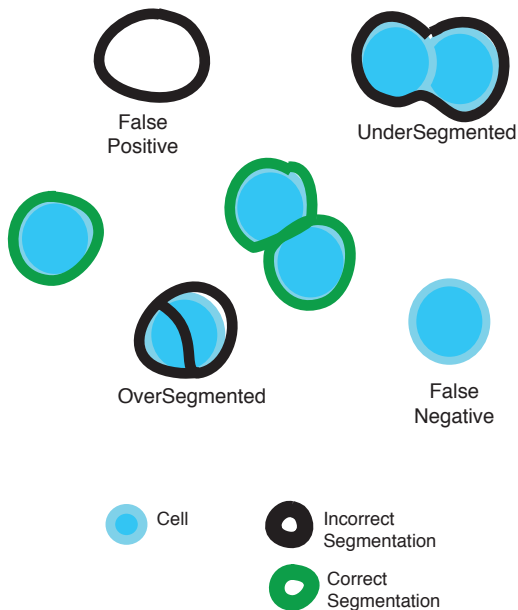**B**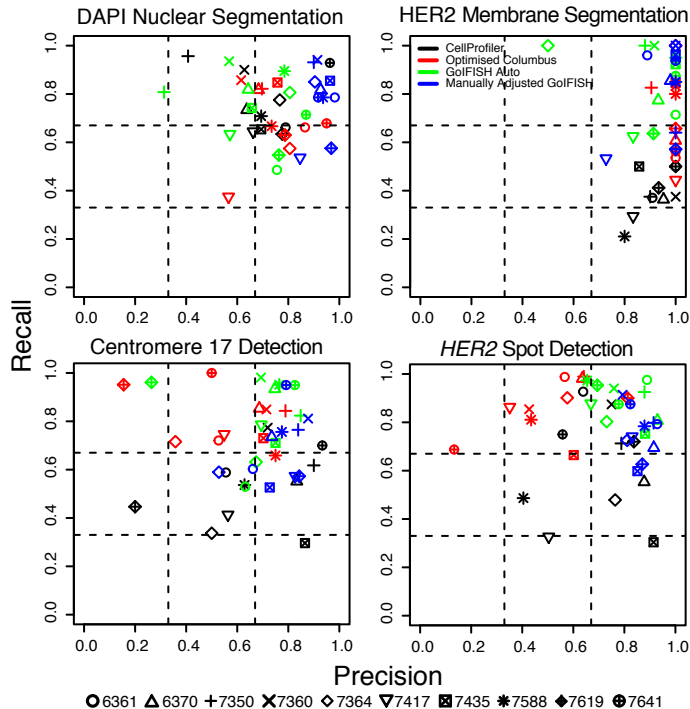

Supplement: Additional file 4 — Figure S1. Precision and recall in cell segmentation. (A) Illustration of the metrics used for Precision-Recall Testing (B) Precision Recall Plots for nuclear segmentation, HER membrane detection, centromere 17 spot detection and HER2 cluster detection. [file 13059_2014_442_MOESM4_ESM.pdf]

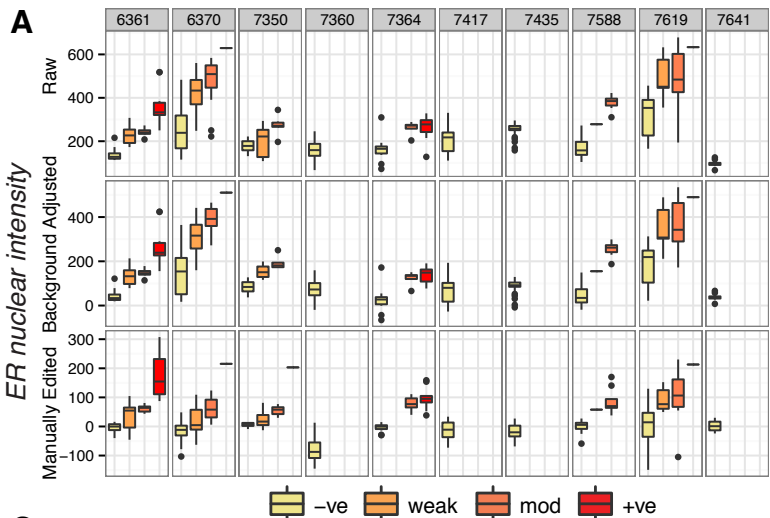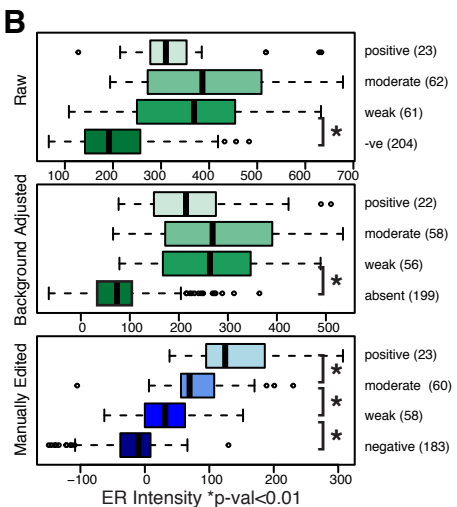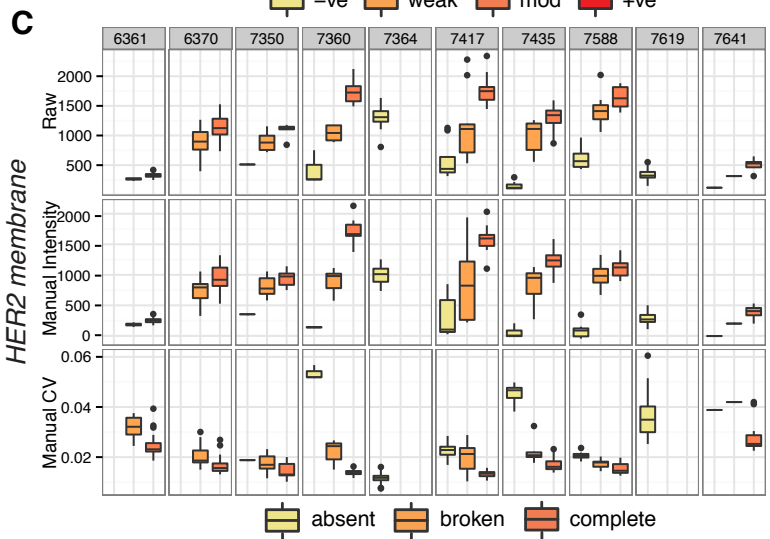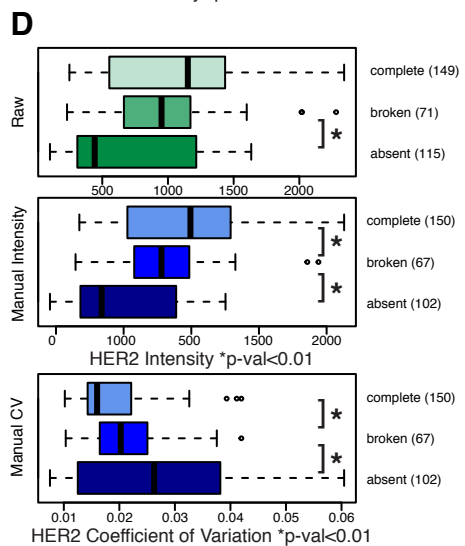

Supplement: Additional file 5 — Figure S2. Correlation between intensity and pathologist scoring. (A) ER staining intensity using GoIFISH raw, background adjusted and manually edited nuclear adjusted intensities compared to semi-quantitative pathologist scored ER intensity for each individual image. (B) Combined distribution of ER intensities across all samples using the methods described in (A) (C) Distribution of GoIFISH raw, GoIFISH manually edited mean HER2 intensities and GoIFISH manually edited coefficient of variation compared to pathologist scored membrane completeness for each individual image. (D) Distribution of HER2 intensities or coefficient of variation across all samples using the methods described in (C). [file 13059_2014_442_MOESM5_ESM.pdf]
