# Supplementary material for: GoIFISH: a system for the quantification of single cell heterogeneity from IFISH images
Source: Genome Biol. 2014 Aug 26;15(8):442. doi: 10.1186/s13059-014-0442-y (PMC4167144; doi:10.1186/s13059-014-0442-y)
Supplement: Additional file 2 — GoIFISH user manual. A user guide for the operation of GoIFISH. [file 13059_2014_442_MOESM2_ESM.pdf]

GoIFISH: A MATLAB toolbox for quantification of single cell heterogeneity in IFISH images

User guide for software described in "GoIFISH: A system for the quantification of single cell heterogeneity from IFISH images"

Trinh et al, 2014

anne.trinh@cruk.cam.ac.uk

Contents

1 Introduction 2

2 File Conversion using Bio-Formats 2

2.1 Using Bio-Formats 2

2.2 Stitching Mosaic Images 3

3 Nuclear Segmentation 3

4 Membrane Staining 5

4.1 Implementation 6

5 Spot Detection 6

6 Postprocessing 7

6.1 Mapping Segmentations to DAPI 7

6.2 Visualisation 8

7 "GoIFISHWrapper": A wrapper for batch analyses 8

7.1 ParamFile.m 9

7.2 GoIFISHWrapper 9

8 Using the GUI 11

8.1 Installation and Operation 11

8.2 Loading the Data & Preprocessing 12

8.3 Background Intensity Measurements 13

8.3.1 Guidelines for background subtraction 13

8.3.2 Implementing background subtraction 14

8.4 DAPI Segmentation 14

8.5 Manual Editing Toolbox 17

8.6 Membrane Staining 18
8.7 Spot Detection 19
8.8 Post Segmentation Processing 19
8.8.1 Collating channel information 19
8.8.2 Cell Labelling 20
8.9 Saving Progress 22

9 Troubleshooting **24**
9.1 Optimal Image Properties for Use 24
9.2 Quick Guide to Different Magnifications 24
9.3 Potential Issues 24

1 Introduction

The GoIFISH package contains code developed for quantitative measurement of features from IFISH images. The toolbox contains both a user interface, where manual changes to segmentation results can be easily achieved, and scripts which can be implemented via command line.

This software available at www.sourceforge.net/projects/goifish under the GNU General Public License version 2. A stand alone version of the GUI is also available for both Mac and Windows Operating Systems, and requires installation of MATLAB Compiler Runtime V 7.14 (provided)

The package was scripted in `MATLAB Version 7.11.0.584 (R2010b)` with `Image Processing Toolbox Version 7.1` and `Statistics Toolbox 7.4`. It is dependent on OMERO Bio-formats for the conversion of file formats for loading [1].

The following sections describe how to perform segmentations using command line. For an in depth description of how to use the GUI, please go to Section 8.

The program is suited for images attained at 60x magnification. It can be applied to those at lower magnification (40x and 20x), however, may suffer from poorer performance as spots are smaller and more difficult to see. Keep in mind that the image itself is dependent on the type of microscope and the detector used, thus some images captured at 40x may be better processed at 60x and vice versa.

2 File Conversion using Bio-Formats

2.1 Using Bio-Formats

The raw .zvi files were converted to .mat files using the OMERO Bio-Formats toolbox [1]. This is available for download from www.openmicroscopy.org/site/support/bio-formats4/. The individual z-stacks can be opened:

```
data = bfopen('Sample7641_Raw.zvi');
data = data{1};
images = data(:,1);
save('Sample7641_new.mat', 'images');
```

When loading into the GoIFISH GUI, each file is expected to contain a cell array of images, of which a maximum of 5 is permitted. There is no restriction of channel numbers for command line processing.

Alternatively, images in TIFF format can be used directly in the GUI eg. extracted from ImageJ, or as shown below:

```
planeSeries=zeros(size(images{1},1), size(images{1},2), 1, 5, 'uint16');
for i=1:5
planeSeries(:,:,:,i)=images{i};
```

```
end
bfsave(planeSeries, 'Sample7641_new.tiff')
```

2.2 Stitching Mosaic Images

The function CollateMosaic has been included to extract images from a raw file format (eg. zvi or .lif) and save it as a .mat file for loading into the GUI. The function stitches together mosaic images, depending on the input parameters described below and in Figure 1.

The main input variables for the function are:

- dir = the directory of the file
- nrow = the number of subimages in each row
- ncol = the number of subimages in each column
- SortBy = whether all subimages of the same stain is listed first (0) or all stains for a sublocation is listed first (1)
- ByRow = is the image sorted by column (0) or row (1)
- Snake = is the image is stitched together in a snake-like manner (0 or 1)
- nchan = the number of stains.

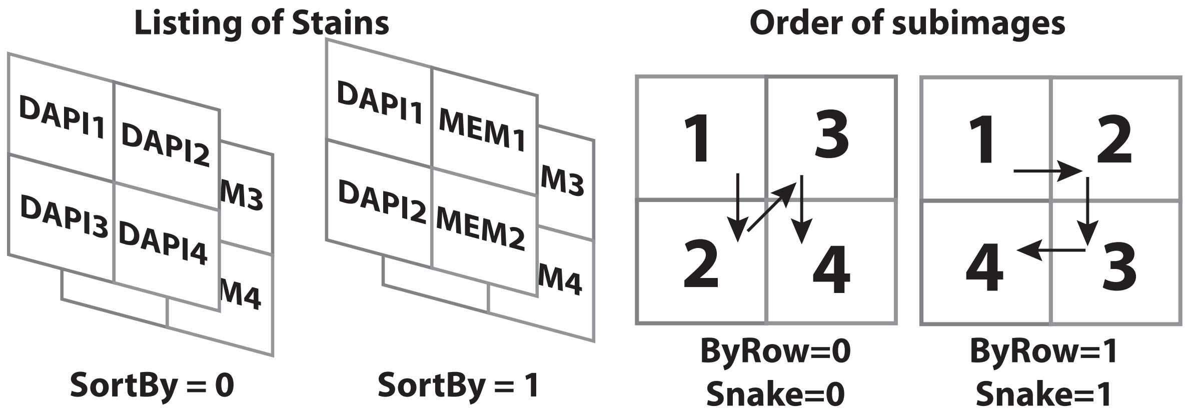

Figure 1: Examples of SortBy, ByRow and Snake inputs into CollateMosaic

We can stitch together a 2x2 image which is sorted by stain, row and in snake manner

```
images = CollateMosaic('Example_Mosaic.zvi', 2, 2, 0, 1, 1, 5);
imview(imadjust(images{1}))
```

3 Nuclear Segmentation

Cell segmentation can be performed using either a 'seeded' method [2] or 'Hminima' method [3] depending on the number of cells present, the contrast within the image, and user time.

Whilst the 'Hminima' method is purely automated, the 'seeded' method will require user inputs to register where cells are located.

The segmentation is performed iteratively using the 'Hminima' method: After variations in intensity less than a given value T are suppressed in the image I, watershed segmentation is performed. A classifier is applied to select appropriately shaped fragments, and the remainder is subjected to another round of segmentation with a slightly reduced threshold. This is applied until the lower threshold is reached.

There is also an option to 'autocorrect' oversegmented fragments in this process. Fragments which are classified as being too small or having an odd shape will be merged with surrounding fragments if the solidity and the deviation from the 'theoretical area' is reduced:

For an oversegmented fragment f we list all neighboring fragments $\mathbf{g} = \{g_1, \ldots, g_N\}$ within a radius of r.

Each union $c^j = f \cup \mathbf{g}^j$ of f with a subset of neighboring fragments $\mathbf{g}^j \subseteq \mathbf{g}$ is a potential extension of f into a full cell. The index j ranges over all members of the powerset of $\mathbf{g}$, i.e., the set of all its subsets.

To select the best extension c^*, we first score all possible extensions c^j by a function $S_F(c^j)$ that compares a feature F (e.g. solidity) of the combined fragments to the average of that feature in the individual fragments:

$$S_F(c^j) = F(c^j) - \frac{1}{n_j} \sum_{i=1}^{n_j} F(c_i^j), \text{ where } n_j = |c^j|, \tag{1}$$

and then sum all feature-wise scores into a final score: $S(c^j) = \sum S_F(c^j)$, which we use to select the optimal extension c^* as

$$c^* = \underset{j}{\operatorname{argmax}} \; S(c^j). \tag{2}$$

If $\mathbf{g} = \emptyset$ or if there is no positive maximum of the score, then $c^* = f$.

Table 1: Optional parameters for DAPI cell segmentation

Parameter	Description
`'mode'`	'imhmin': using H-minima transform to suppress small intensity depths. 'seeded': Good for strong edge boundaries.
`'efiltT'`	Entropy threshold to determine foregound. Between 0 (Selects everything) and 1 (selects nothing) Omitting this will auto detect the background
`'merge'`	Binary index to automerge undersegmented cells. Default off (0)
`'thresh'`	Thresholds to use in boundary detection. Segmentation performed iteratively between high and low value between (0, 1)
`'mag'`	Magnification Used. Default is `'60x'`. `'40x'` and `'20x'` can also be specified.
`'seed'`	Map of cell locations in Seeded Watershed.

```
% perform DAPI segmentation
% use H-minima Transform at intensity depths 0.3 to 0.15
% Automatically determine the background-foreground threshold and report this value
```

```
% Automatically merge
DAPIIm=imadjust(images{1});
[DAPIMap efiltT] = CellSegmentIF(DAPIIm, 'mode',  'imhmin', 'thresh', [0.14 0.3],...
     'merge', 1, 'mag', '60x');
imview(SegArea(DAPIIm, DAPIMap))
% Find morphological properties
DAPIProps=regionprops(DAPIMap, images{1}, 'Area', 'Perimeter', 'MeanIntensity');
DAPIProps
```

This will perform one iteration of segmentation, and the result should be similar to that shown in Figure 5. To implement the seeded segmentation, it is recommended to attain a seed map using the GUI.

4 Membrane Staining

The segmentation of membranes involves a Voronoi segmentation, followed by potential refinement using active contours. A combination of nuclear voronoi segmentation and membrane intensity map from the image is used to detect cell boundaries. Membranes in negatively stained samples will will be along nuclear edges.

This usually results in good segmentation, however may be jagged depending on the staining in the original image. To reduce this, we have added the option of using active contours to align the segments to the contours observed within the image using two methods: the Chan-Vese [4], and the Localised Segmentation method [5].

The Chan-Vese method attempts to minimise the force associated with a curve ϕ and in doing so solves the following PDE:

$$\frac{\partial \phi}{\partial t} = \delta_\epsilon[\mu(\frac{\nabla \phi}{|\nabla \phi|}) - \nu - \lambda_1(u_o - c_1)^2 + \lambda_2(u_o - c_2)^2] = 0 \quad (3)$$

where λ_1 and λ_2 are coefficients relating to the force on the inside and the outside of the curve, both set to 1. ν is a penalty associated with the the area within the curve (set at 0), and μ optimises the length of the curve to keep it smooth, which is set at 65. δ_ϵ is the differential of the HeavySide Function, which looks at the area around the curve, and ϵ has here been set to 1. These values were selected from the corresponding publication [4], and can be altered in command line implementation.

The Localised Segmentation method uses a similar approach, and includes an indicator term $B(x, y)$ which applies the force term locally within a radius of r. λ is used in the place of μ and is assigned the value 0.05. The force coefficients are set at 1.

4.1 Implementation

Membrane segmentation can be performed after attaining a nuclei segmentation map. The inputs include a map of the DAPI segmentation, and the membrane image of interest. If the latter is omitted, nuclear voronoi segmentation is performed. The two outputs of `EllipseVoronoi` are: an `EdgeMap` of the segment boundaries, and an `AreaMap` which shows non-connected cell regions.

It is possible to scale the image to a smaller resolution and perform the membrane detection to reduce segmentation time. A variable second input parameter `Scale` will rescale the image to a fraction of the original image. This value must be between 0 and 1. If omitted, a value of 1 will be assigned and no rescaling will be performed.

Using the output `AreaMap`, the segmentation can be further refined using active contours. The user can indicate the type used (`'CV'` for Chen-Vese method or `'Loc'` localised segmentation).

The input `Properties` is required to perform the smoothing algorithm. It is a vector containing the following values: `[No Iterations, Time Step,` μ, ν, ϵ, λ_1, λ_2`]`. Where a parameter is not required, place a 0 in its position.

```
% Running Membrane Segmentation, followed by localised Segmentation
% Rescale image to 50% to perform segmentation
% Properties in form of [ No Iterations, dt, mu, v, eps, L1, L2]
HER2Im=imadjust(images{2});
[EdgeMap AreaMap]=EllipseVoronoi(DAPIMap,  HER2Im, 0.5);
imview(SegArea(HER2Im, EdgeMap));
imview(SegArea(HER2Im, AreaMap));
Properties=[100 2 0 1 eps  0.05 0];
[newAreaMap, newEdgeMap]= ActiveContSmooth(AreaMap, HER2Im, 'Loc', Properties);
```

The output of this segmentation is shown in Figure 7.

5 Spot Detection

Spot detection is performed using two separate functions to determine

1. Individual spots and
2. Clusters of spots which cannot be differentiated by eye

The first function `SpotSearch` aims to find individual spots. A Laplacian of Gaussian filter is applied to determine spot candidates. These are then filtered according to spot size (default value 15 pixels) and relative intensity in the gradient image (between 0 and 1, this will be automatically be detected at 1.5 standard deviation from the mean). A `DAPIMap` will be required to ensure these spots lie within the main cellular area. There is an option to select spots which are circular in shape in order to avoid artefacts based on SVM.

The second function `SpotRegDetect` looks at the gradient image to determine local maxima, or clusters. This function will also require an 'minimum cluster size' and will be set by default to 15 pixels.

```
% perform Spot Detection.
% Min Size 15, Automatic Thresholding, Do not Optimise Shape
ERBIm=rangeScale(images{5});
Spots1 = SpotSearch(ERBIm, DAPIMap, 15, 0, 0);
imview(SegArea(DAPIIm, Spots1));
% Perform cluster detection
Spots2 = SpotRegDetect(ERBIm, DAPIMap, 15);
% Combine the two:
NewSpots=logical(Spots1+Spots2);
imview(SegArea(ERBIm, NewSpots))
```

6 Postprocessing

6.1 Mapping Segmentations to DAPI

In order to attain useful information about each cell, we will need to map all segmentations to the DAPI channel. The function `AdjustOverlap` is provided to do this: It will take two segmentations and map the second to the first. Entering in the original image used, and a background intensity information will also give you information about the staining: Note that we wish to obtain the raw intensity values for the HER2 and Spot channel, hence, we are referring to the original `image` to ensure comparability between samples.

```
% Map HER2 to DAPI
% Assume background intensity value of 0
[HER2DAPI, HER2Props]=AdjustOverlap(DAPIMap, AreaMap, images{2}, 0, 'mem');
imview(SegArea(DAPIIm, HER2DAPI))
% Map the spots to DAPI:
[Spot2DAPI, SpotProps]=AdjustOverlap(DAPIMap, NewSpots, ERBIm, 0, 'spot');
imview(SegArea(DAPIIm, Spot2DAPI))
% Combine Properties
HER2Props
SpotProps
OutputMatrix=[struct2mat(DAPIProps), struct2mat(HER2Props), struct2mat(SpotProps)];
```

Both `HER2Props` and `SpotProps` should have the same number of entries as `DAPIProps`. Note that the `'Mean Intensity'` in the HER2 Properties refers to a cytoplasmic intensity. `'MembraneIntensity'` refers to intensity at the membrane. Adjusted Intensities are calculated for the membrane.

6.2 Visualisation

Once this is complete, one can generate heatmaps of the relative intensity of the staining within an image

```
% HeatMap of the HER2 channel
HER2_HM=IntensityOverlap(HER2DAPI, [HER2Props.MembraneIntensity]);
imview(HER2_HM)
% Can compose this as an overlay with the DAPI channel:
HER2_overlay=cat(3, 0.5*im2double(DAPIIm)+0.75*HER2_HM, ...
0.5*im2double(DAPIIm), 0.5*im2double(DAPIIm));
```

Heatmaps can be overlaid with one another to create topology maps which highlight variations of two features within the same image. Limits can be applied to scale the images based on the maximum intensity expected from a general population. Alternatively, if one enters in single values, they will be considered as thresholds

```
% Create Topology map of HER2 protein and spot intensity.
% Set HER2 protein limit [0 2000]
% Set Spot Area limit [0 800]
Topo_HER2 = Topology(DAPIMap,  [HER2Props.MembraneIntensity], [0 2000],...
 [SpotProps.Area], [0 800]);
imview(Topo_HER2)
% Enter in thresholds of 300 for protein and 50 for spots.
Topo_Class = Topology(DAPIMap,  [HER2Props.MembraneIntensity], 300,...
 [SpotProps.Area], 50);
imview(SegArea(DAPIIm, Topo_Class))
```

The two maps above are shown in Figure 9A and B. Classification based on thresholds are often arbitrary, hence, topology maps may be useful for the visualisation of subtle differences between samples

7 "GoIFISHWrapper": A wrapper for batch analyses

In order to easily perform batch analyses, a wrapper has been constructed to analyse images on command line. In order to operate this, two pieces of information are required: the directory of the file for analysis and a parameter file `'ParamFile.m'` file which needs to be edited for each analysis.

The advantages of running the Wrapper over the GUI include the capability of running samples with more than 5 channels, analysing raw image files (eg. 'zvi', 'lif'), and automatic stitching of mosaic images (See section 1 for more information). The output of the wrapper is a .mat file which can be loaded into the GUI to check the segmentation results.

7.1 ParamFile.m

The parameter file contains a series of Parameters which need to be filled out in order to run the analysis: If the original `ParamFile.m` becomes corrupted, the original text is embedded within `‘ParamFile_README.txt’` which can be copied to create a new file. A description of the parameters, permitted values and their original values are listed in Table 2

Table 2: Summary of the parameters required for Batch Analysis

Variable	Description	Default
Image Properties		
`Resolution`	Magnification of image. Accepts `’60x’`, `’40x’` or `’20x’`	`’60x’`
`StainSeries`	Order of the stains within the channel in a cell array. Accepts `’DAPI’`, `’Mem’` (Membrane Stain), `’Spot’`, `’Nuc’`(Nuclear Stain)	{`’DAPI’`, `’Mem’`, `’Spot’`, `’Nuc’`, `’Spot’`}
`Nrow`	Mosaic Image: Number of rows of subimages	1
`Ncol`	Mosaic Image: Number of columns of subimages	1
`SortBy`	Mosaic Image: Are images listed by stain (0) or location first (1). See section 1	1
`ByRow`	Mosaic Image: Stitch by row? (0 = no, 1 = yes) See section 1	1
`Snake`	Mosaic Image: Images stitched by snake arrangement? (0 = no, 1 = yes) See section 1	1
DAPI Properties		
`Merge`	Merge oversegmented fragments? `’On’` or `’Off’`	`’On’`
`Thresh`	Intensity depth Thresholds at which to perform segmentation. Must be a vector with 2 values within (0, 1)	[0.14 0.3]
Membrane Properties		
`Type`	Segmentation type performed: `’Default’`, `’ChanVese’` or `’Localised’`	`’Default’`
`Scale`	Rescales the membrane image to perform faster segmentation. Less than or equal to 1	0.25
`ChanVese`	Parameters for Chan-Vese segmentation. See section 4	[100 2 65 0 1 1 1]
`Localised`	Parameters for Localised segmentation. See section 4	[100 2 0 0 eps 0.05 0]
Spot Segmentation Properties<br>NB: If there are multiple spot images, include a vector containing the designated value for each image.		
`SpotSize`	Minimum spot size accepted in pixels	[15 15] (2 channels)
`IntensityThreshold`	Intensity threshold used to select spots between [0 1]. 0=automatic detection	[0 0] (2 channels)
`SpotOpt`	Optimise the spot selection by including morphological constraints? (0 = no, 1 = yes)	[0 1] (2 channels)
Report Properties		
`ReportProps`	Report properties of each cell after segmentation? `’On’` or `’Off’`	`’Off’`

Please ensure quotations are used appropriately for text-based inputs, otherwise they will not be accepted.

7.2 GoIFISHWrapper

Once the ParamFile.m is edited, the GoIFISHWrapper can be run.

Currently accepted image inputs include:

.mat file the images must be present in a single cell array of variable name `images`

.TIF file

raw image file : must be maximum projection image. z-stacks not accepted. Number of subimages (`Nrow, Ncol`) and the orientation of the layout (`SortBy, ByRow, Snake`) must be specified

Note that currently, mosaic images in maximum projection raw image format can be processed. In order to run these, the number of rows and columns of the composed image must be specified, in addition to information on how to sort the subimages (see section 1).

Note that multiple Parameter Files may be created for a Batch Job, each specific for a particular image. The `GoIFISHWrapper` permits a secondary variable which is a string containing the name of the new Parameter File, omitting the '.m' suffix

```
   % Set up Batch Processing of file:
% 1. Change the following properties in the Param File
Ncol=2;
Nrow=2;
SortBy=0;
ByRow=1;
Snake=1;
% 2. Run the Wrapper
GoIFISHWrapper('Example_Mosaic.zvi')
% 3. Running the Wrapper if a separate Parameter File 'New_Param.m' exists
Save ParamFile.m as New_Param.m
GoIFISHWrapper('Example_Mosaic.zvi', 'New_Param')
```

8 Using the GUI

A overview of the GUI and quick description of its functionality is presented in Figure 2. We will discuss how to run an example analysis with the given sample image, 7641.

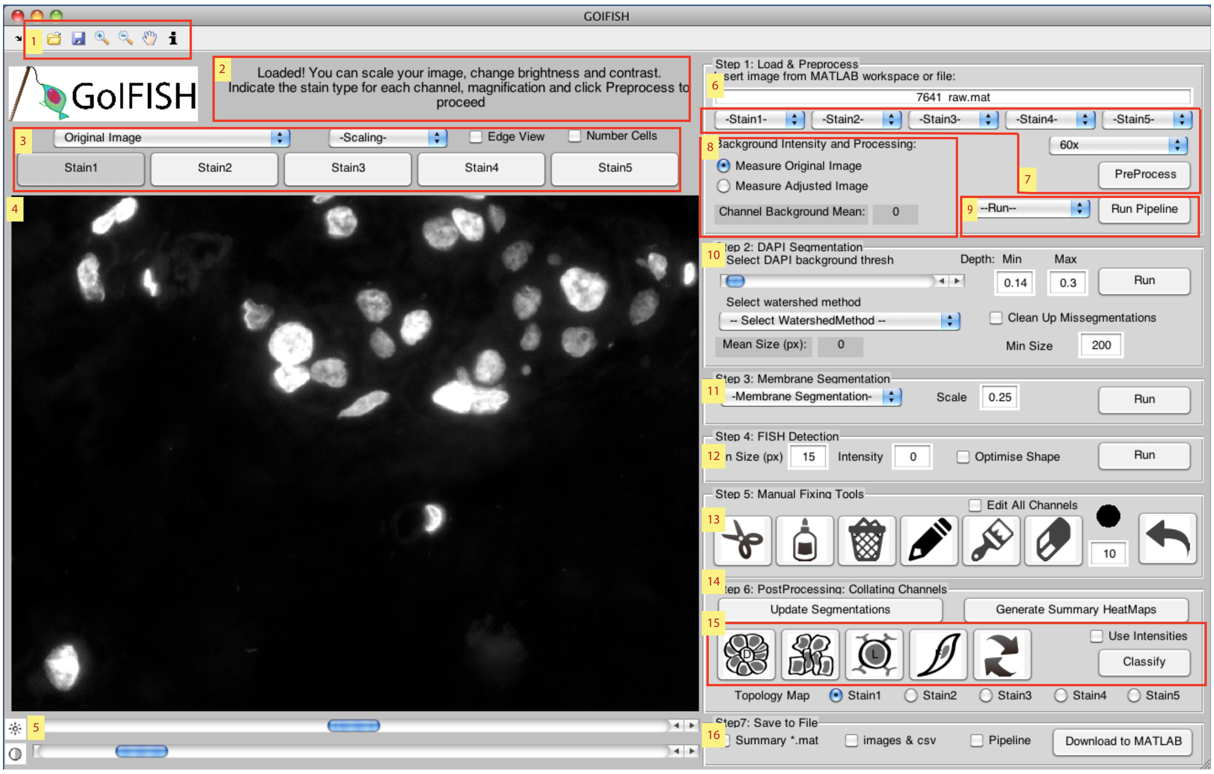

Figure 2: Overview of the interface. (1) Load and save images, zoom and pan tools (2) Message Box giving real time feedback (3) Viewing Toolbar: View Stain Channels, Segmentations with numbers and edges. Adjust automatic scaling of input image (4) Plot Area (5) Brightness and Contrast: Automatically set to 0 and 1 (6) Name of the file. Can also load a cell array of images from MATLAB environment by typing in its name (7) Preprocessing: Select Stains corresponding to each channel and magnification (8) Background Intensity Adjustment. Also reports the intensity of the background in each channel (9) Segmentation using Default Settings. Can run all channels in one go, or can run DAPI separately (10) Change the DAPI watershed properties (11) Membrane Segmentation Toolbar (12) Spot Detection Toolbar (13) Manual Editing Toolbar (14) Postprocessing: Registers all segmentations with respect to the DAPI channel. Following this topology maps can be generated and classification can be performed(15) Cell classification. 4 classes permitted (16) Save Toolbar: Can save summary .mat files, images and txt file with properties, or Progress File. Click on blue save button in (1) to complete. Can also directly import to MATLAB workspace

8.1 Installation and Operation

The GUI can be called within the MATLAB environment as a simple call to `GOIFISH` in the terminal space after setting the correct path to the folder.

When running the GUI outside of MATLAB, first install MATLAB Compiler Runtime (appropriate .dmg file or .exe file is available). On the first execution, the program may need to be run from the command line. After this first initiation, calling the application directly is sufficient:

```
On a MAC:
 1. Install MATLAB Compiler Runtime
 2. Set Terminal to the appropriate working file.
  eg. cd /goifish-code/GOIFISH_App/distrib/
 3. Execute the application (initial run):
  ./run_GOIFISH.sh /Applications/MATLAB/MATLAB_Compiler_Runtime/v714/
  (May be different if MCR directory is not installed at default location).
 4. Open app by double clicking
 On PC:
 1. Install MATLAB Compiler Runtime
 2. Double click application
```

8.2 Loading the Data & Preprocessing

Images can be loaded as a .mat or .tiff file or as a cell array from the workspace. After successful loading, the first image in series will be presented. These will be automatically adjusted to ensure 1% of pixels is saturated at lower and higher intensities. Whilst this is generally good for nuclear or cytoplasmic images, it may saturate spots. The 'RangeScale' option is recommended for spot detection. Additionally, there is an option of disabling any scaling if this is not required. Brightness and contrast of each image can be adjusted (Figure 2, part 5) to adjust for auto-fluorescence or optimise contrast to ensure good segmentation. Note that image adjustment is mainly to improve the user-experience and segmentation results. Intensity outputs can be attained either from the adjusted image or from the raw file.

The user will need to indicate the image resolution (60x, 40x or 20x) and the stain type in each channel, of which one must be DAPI in order to proceed (See Figure 2, Part 7). If a DAPI channel is not present, a combination of nuclear stains could potentially be used to create an "imitation" DAPI channel. Following this, a quick preprocessing is applied to assign the stain type to each image (pushbutton 'Preprocess') to ensure only one DAPI channel is present. After this, segmentations on either the DAPI channel or all channels using default parameters can be performed (See Figure 2, Part 9) .

The user can also run the automatic segmentation with user-defined input arguments described in later sections. This may be particularly useful if the 'default' options provide poor segmentation results.

Loading the example image:

```
1. Open the given file '7641_raw.mat'.
  You will see the DAPI channel presented as in Figure 1.
  Click 'Stain2' to see what the HER2 stain will look like.
2. Select stains in the following order:
  'DAPI', 'Membrane', 'FISH', 'Nuclear', 'FISH'
3. Change the scaling in the two 'FISH' channels to 'rangeScale'.
```

```
 You will see that channel 5 will have less auto-fluorescence
4. Set magnification to '60x'
5. Click 'Preprocess'. This will ensure that all channels are labelled and only
  1 DAPI channel is present.
6. Using the contrast slider below the plot area, change the contrast and brightness
 if necessary. The default value is 1 for contrast and 0 for brightness. This may
 affect segmentation results, but not necessarily intensity measurements.
```

8.3 Background Intensity Measurements

The user has the option of selecting a 'background' intensity which can be subtracted from the measured intensity in order to account for differences in background between samples. This involves the calculation of a single background intensity value per stain and subtracting this either from the original image, which will be useful for comparisons between samples, or a 'user optimised image' which allows the user to focus on the heterogeneity between cells within a single image.

Additionally, a 'Nuclear Adjusted Intensity' will take into account fluorescence in the cytoplasmic region and subtract this from each nuclear measurement. This takes into account regional variations within the same image.

8.3.1 Guidelines for background subtraction

For optimal background subtraction it is important to select a representative background area for each specific channel or marker, as described in Table 3 and illustrated in Figure 3.

Table 3: Guidelines for background subtraction

Stain Type	Optimal Background Areas
nuclear IF (including DAPI)	Cell-free area with no artefacts, debris nor auto-fluorescence
membrane IF	Cell-free area with no artefacts, debris nor auto-fluorescence
FISH	Signal-free area within nuclei

For single images (non-mosaic) photographed with a constant exposure time, one area selected for background subtraction should be sufficient. However, in cases with high variation in the background intensity due to high auto-fluorescence in the tissue, the strongly auto-fluorescent areas **should not** be selected for background subtraction as this will give an artificially high background intensity reading.

It is strongly recommended that cells within highly auto-fluorescent IF regions are discarded from further analysis. The intensity values of the IF channels may be artificially low after background subtraction and may interfere with FISH intensity readings. Cell deletion can easily be achieved using the trash or eraser tool in the manual editing toolbox (See section 8.6)

8.3.2 Implementing background subtraction

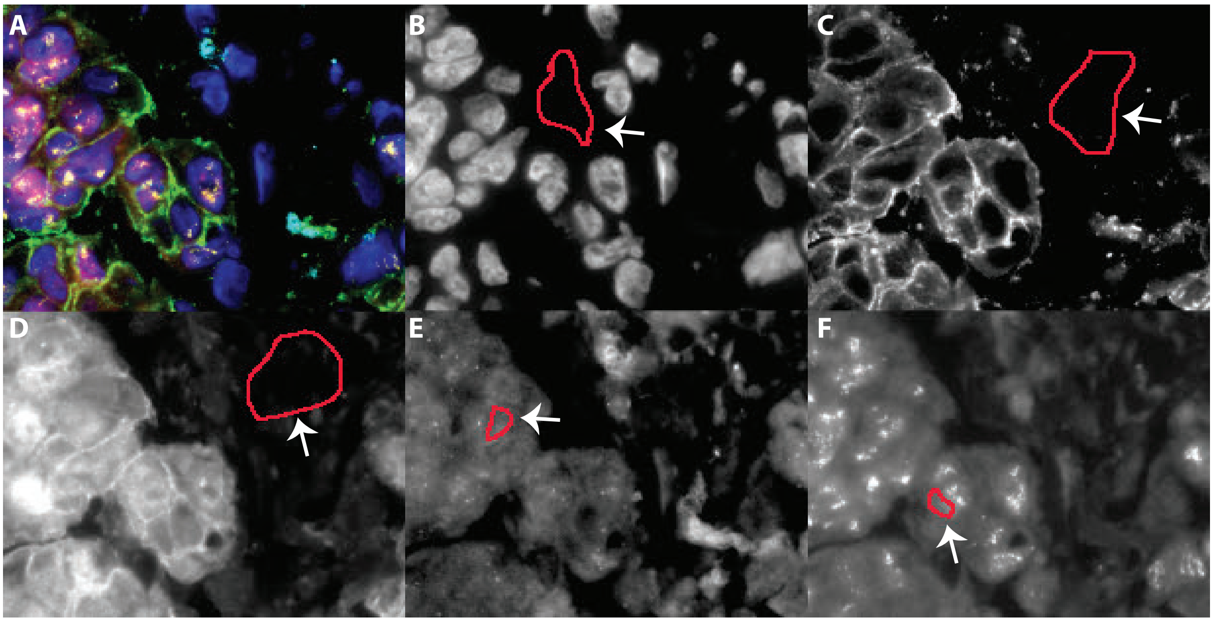

Figure 3: Guidelines for background subtraction in images with DAPI, membrane and nuclei protein stainings and FISH. (A) Original image with stainings for DAPI (blue), HER2 protein (green), ER (red), *HER2* gene (yellow) and centromere 17 (cyan). (B-F) Regions for background subtraction: (B) DAPI channel (C) membrane staining (HER2 protein) (D) nuclei protein (ER) (E) FISH channel (centromere 17) and (F) FISH channel (*HER2*).

Defining a background intensity:

```
1. Select 'Measure Original Image'. This will measure the intensity of the original image,
 regardless of any alterations in contrast or brightness performed.
2. Go to View > Background Region
3. Click on the brush tool to paint a background region. Right click once you have
 finished and the shape should be filled in. Continue painting a number of regions
 until satisfied. Press escape on the keyboard to exit.
4. Use the eraser in a similar manner to remove any regions overlapping cells.
5. You should see the 'Channel Background Mean' will be increased from 0.
 Here, it is around 215 for channel 5.
6. Repeat with other channels to select the background areas.
```

8.4 DAPI Segmentation

The user can adjust the parameters required to run the DAPI segmentation. The first step involves determining a suitable background intensity (entropy threshold) to discriminate cell from background (Figure 5). The two watershed methods are available for use: seeded, which requires user input, and H-minima which is completely automated. The H-minima transform requires a maximum and minimum intensity for depth suppression: The default is set from 10 to 30% variation in local intensity.

An option is available for oversegmented samples whereby cell fractions are merged to optimise

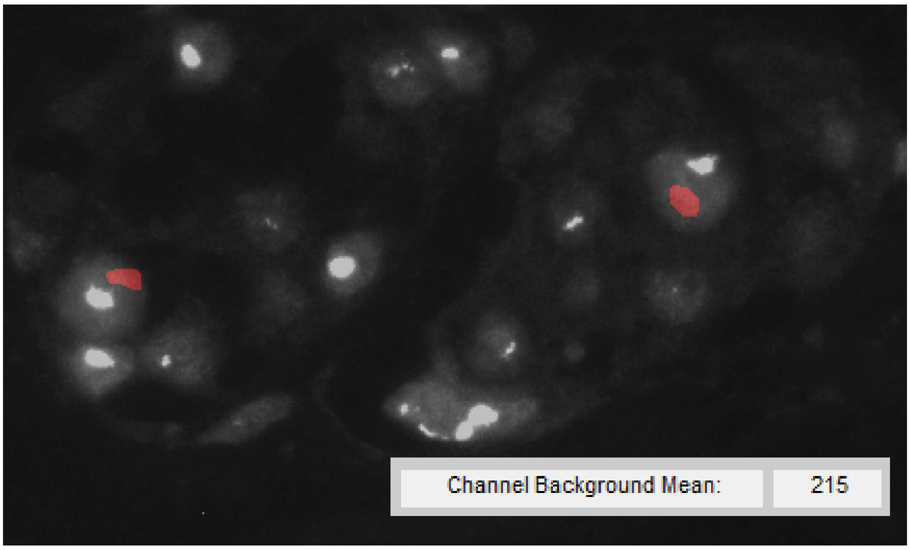

Figure 4: Background Adjustment of HER2 loci channel: Selected background regions are shown in red. The intensity is Calculated to be 215.

morphological parameters (in particular, to maximise solidity). All nuclear stains will adopt the same segmentation as the DAPI channel.

Running DAPI Segmentation Using Default Settings:

```
1. Choose what parts of the pipeline to run using default settings.
 The options include ‘DAPI Only’, ‘Other Channels’ and ‘All Channels’.
 As membrane and spot detection is dependent on DAPI, the user has
 the option of optimising this segmentation before running all others.
 Select ‘DAPI Only’ for now.
2. Click on ‘Run Pipeline’. This will take about 15 seconds for the given sample
  but will increase for images with a high number of cells
3. The output will be shown when complete. If not present, ensure you are on
  Stain 1 and View > Initial Segmentation
```

Running a Seeded DAPI Segmentation:

```
1. The background threshold should still be 0.42 from the previous analysis.
   Otherwise, set it to this value
2. Select watershed Method: Seeded
3. Click on the pencil tool and draw dots on cells
4. Click ‘Run’.
5. Turn on the edge view and number view.
6. To remove small cells, type in 2000 as a ‘Minimum Size’.
7. This process can be reversed by the ‘Undo’ button
```

We see that both methods give similar results, however the seeded watershed can split cells with poorly defined boundaries (eg. cell 7 and 8) with the additional given information (Figure 5B).

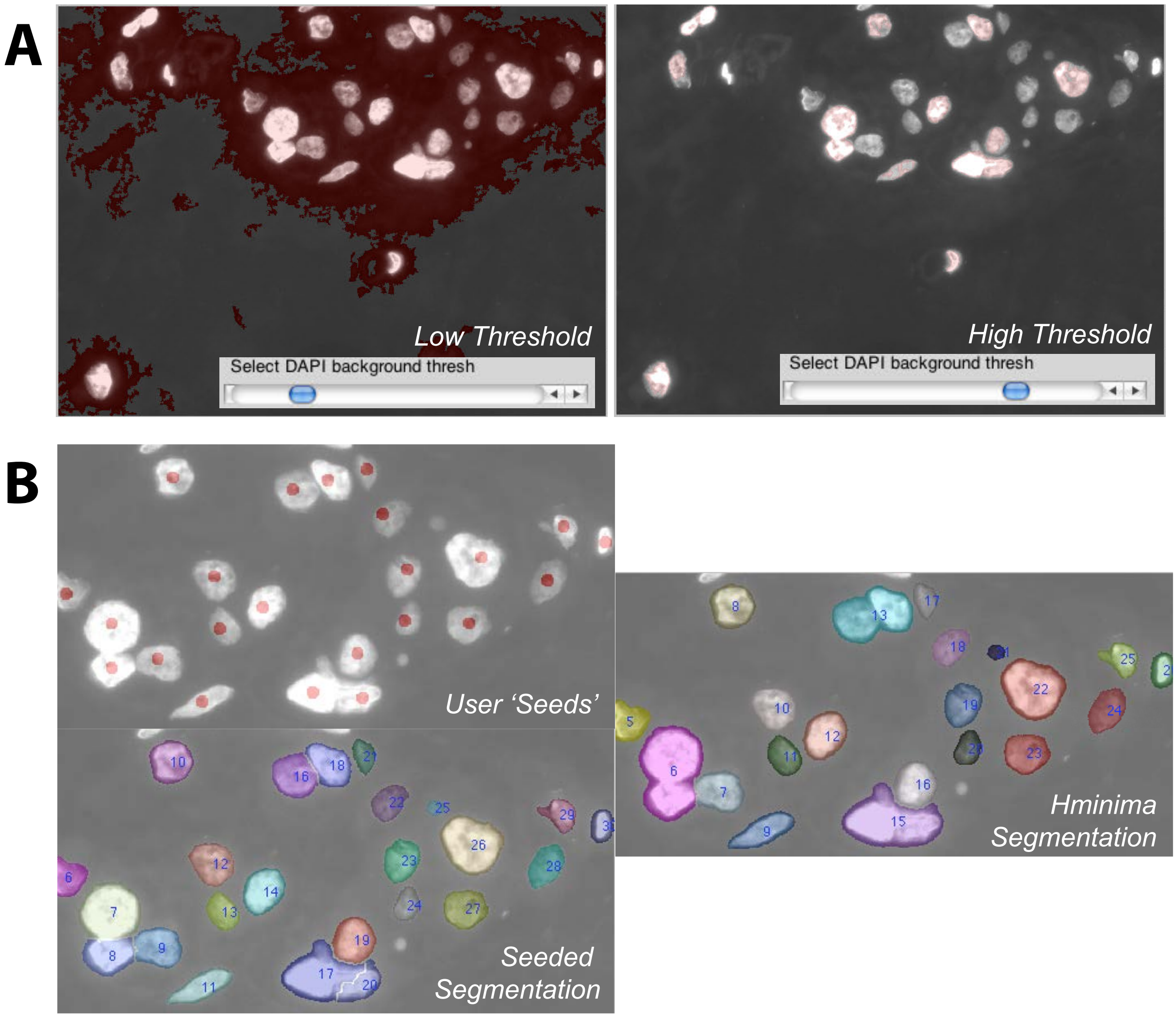

Figure 5: Processes involved in DAPI Segmentation.Top: Selecting an optimal background threshold. The corresponding selected area is highlighted in red. Bottom: Outputs from a seeded watershed compared to a H-minima transform watershed. Selected seeds are shown as red dots in the top image.

8.5 Manual Editing Toolbox

This toolbox contains a number of functions which can be used to easily edit the segmentation if high accuracy for subsequent analysis is required, as shown in Figure 6. The splitting and merging tools are for use mainly in cell segmentations, whereby the user clicks to draw an edge in undersegmented cells (shown in Figure 6) or clicks a number of oversegmented fractions to merge them into a single cell. Paintbrush and eraser tools are also available to fill poorly segmented cells, edit spots, or fill in or delete large regions. The pencil tool is used simply to draw dots - in the case of editing a spot detection result or setting seeds for a watershed.

The size of this brush can be changed by manually entering a diameter value (default set to 10). All functions are completed by right clicking, and to exit a mode, press 'Esc' on the keyboard. Each cell is uniquely identified, thus a colour change is expected when a modification has occurred and a new cell number is generated.

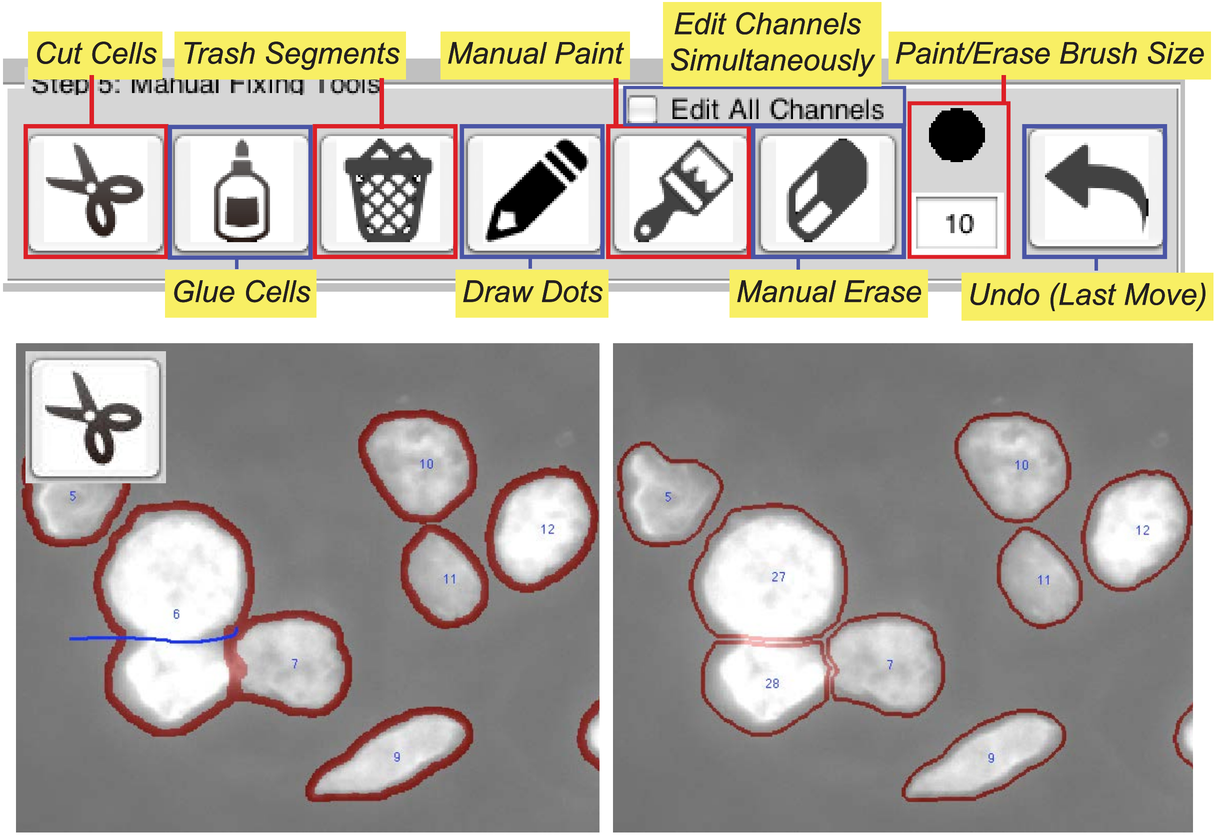

Figure 6: Manual Fixing Toolbar with description of functionality. An example of cutting a segment into two. The blue line indicates the 'manual segmentation path'

Please note well the following when performing segmentations:

1. The undo button will only undo the **last** input command.
2. **Starting and ending** a particular segmentation process will involve **left and right clicking** respectively.
3. After selecting a particular process, that particular process will be functional until the escape button is pressed.

4. Turning on **Edit All Channels** will allow you to apply an edit to all channels. Eg. a merge performed on the DAPI channel will apply to the Membrane channel provided that a default segmentation has been performed on both. Thus, it is recommended to perform cell splits using the membrane channel to ensure the cell is fully segmented, rather than the reverse.

Splitting a cell:

```
1. Re-perform the H-minima segmentation.
 Notice cell number 6 is missegmented as there is not a strong intensity
  gradient between these two cells.
2. Use the ‘Cut’ tool to segment this cell.
2. Right click to finish a particular ‘cut’
3. Press ‘esc’ to exit the ‘Cut’ mode.
```

8.6 Membrane Staining

In order to perform the segmentations, the plot area must display the stain which the user intends to work on. In this example, as we have indicated that Channel 2 is Membranous, membrane segmentation cannot be performed if we are viewing any of the other channels. This is to ensure the correct segmentation is performed on the correct channel. The same applies to Spot Detection.

The default option for membrane segmentation is a nuclear Voronoi Segmentation which is also weighted by intensity of the image. Images with minimal staining will obtain contours which resemble outlines of the nuclei.

Following this, active contour refinement of membrane staining can be performed, or alternatively, the user can manually edit the segmentation. Two smoothing operations are shown: Chan Vese and Localised Segmentation [4, 5]. The Chen Vese method tends to give smoother membrane borders, whereas the Localised method usually results in more jagged cell borders along local maxima, as shown in Figure 7. The computation times for automatic refinement is more expensive for images with larger number of cells, but can be computed within the matter of minutes.

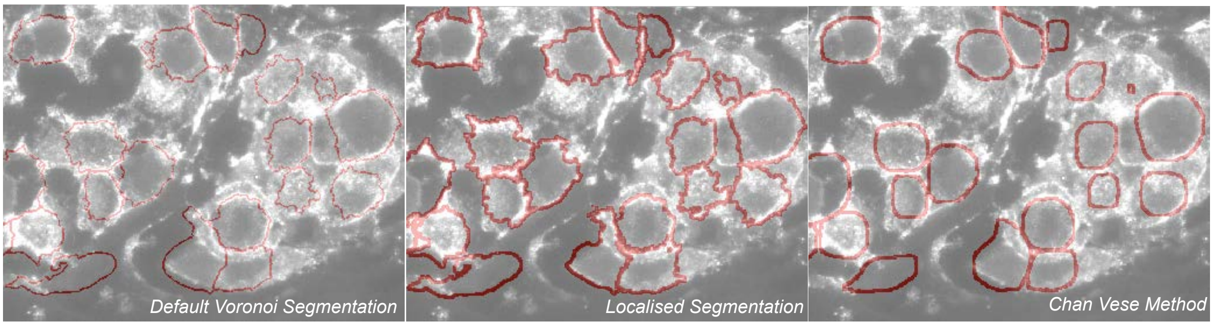

Figure 7: Example of membrane Segmentation. The left shows the default segmentation using Voronoi segmentation. This is passed into active contour smoothing as a starting point. The smoothed image is shown on the right

```
1. Ensure you are viewing stain 2.
2. Select Membrane Segmentation > Default
3. Set scale value to 0.25. This will perform edge detection
on a smaller sized image
4. Press ‘Run’.
```

8.7 Spot Detection

There are three factors to consider in optimal segmentation of spot images: the contrast of the image, a mininum spot size, and a relative intensity threshold (between [0 1]) to define the spot regions.

The contrast within an image is critical for good spot detection. It is recommended that images are ‘rangeScaled’ in order to prevent saturation of spot regions, and contrast is adjusted such that very little background can be seen.

A minimum spot area is set to 15 pixels for a 60x image. The intensity threshold is that used to select between spots and background. This is automatically detected as 1.5 standard deviations away from the mean when the Intensity Threshold is set to 0.

There is also an option for shape optimisation, which is geared towards accurate detection of low intensity spots. All spots must be circular in shape and solid in order to pass this stage of selection. This option is particularly useful for low intensity control markers, such as centromere 17.

Defining ERBB2/HER2 spots using the following procedure should give results similar to that shown in Figure 8

```
1. Ensure you are viewing stain 5.
2. Run the FISH detection using the default parameters:
    Minimum spot size 15, intensity threshold to 0. (auto-detect)
3. Perform the same in Channel 3.
```

8.8 Post Segmentation Processing

Following segmentation, the information from each channel needs to be mapped to the DAPI channel, to construct a feature matrix for each cell. This information is used to generate heat maps, topology maps, and cell classification for further analysis outside the GUI. Topology maps can be constructed for two stains of interest: the colour will indicate the relative ratio between two stains, whilst the intensity will reflect how highly the stains are expressed.

8.8.1 Collating channel information

After segmenting nuclei, spots and membranes, this information needs to be collated in order generate heatmaps, cell classifications and summary information. All segmentations will be

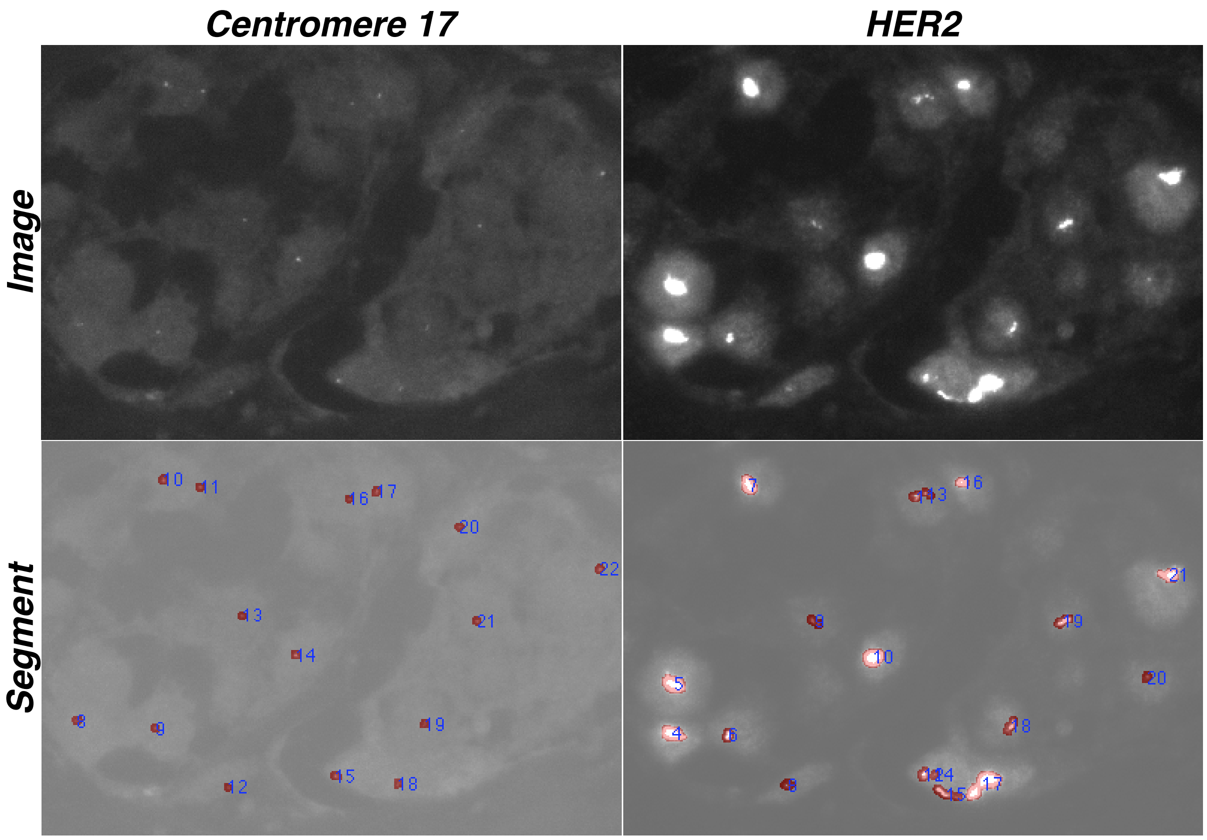

Figure 8: Results of FISH detection in the *Centromere 17* and *HER2* channel using the default settings

mapped to the DAPI channel.

```
1. Click 'Update Segmentations'
2. Turn on number view and flick through the Stains
   The numbers should be in the same location in each channel
3. Click on 'Generate HeatMaps'
   You will see a red heatmap with variable intensity of the DAPI channel
4. Click the different stain options to see the variation between different
  pairs. The closer to magenta, the higher the expression in the viewing
  channel. The more yellow a cell is, the higher the expression in the
  selected channel. A mix between the two will give a red cell.
 5. View the HER2 membrane and select Stain 5. (Figure 8C)
 Note that this shows the variability within one image.
 To make global comparisons, it is recommended to
 create topology maps in the MATLAB environment with global limits. (See section 6).
```

8.8.2 Cell Labelling

Cell labelling can be performed after DAPI nuclei segmentation. 4 labels are available: DCIS, invasive tumour cell, lymphocyte-like cell and normal cells or fibroblasts. User labels can be

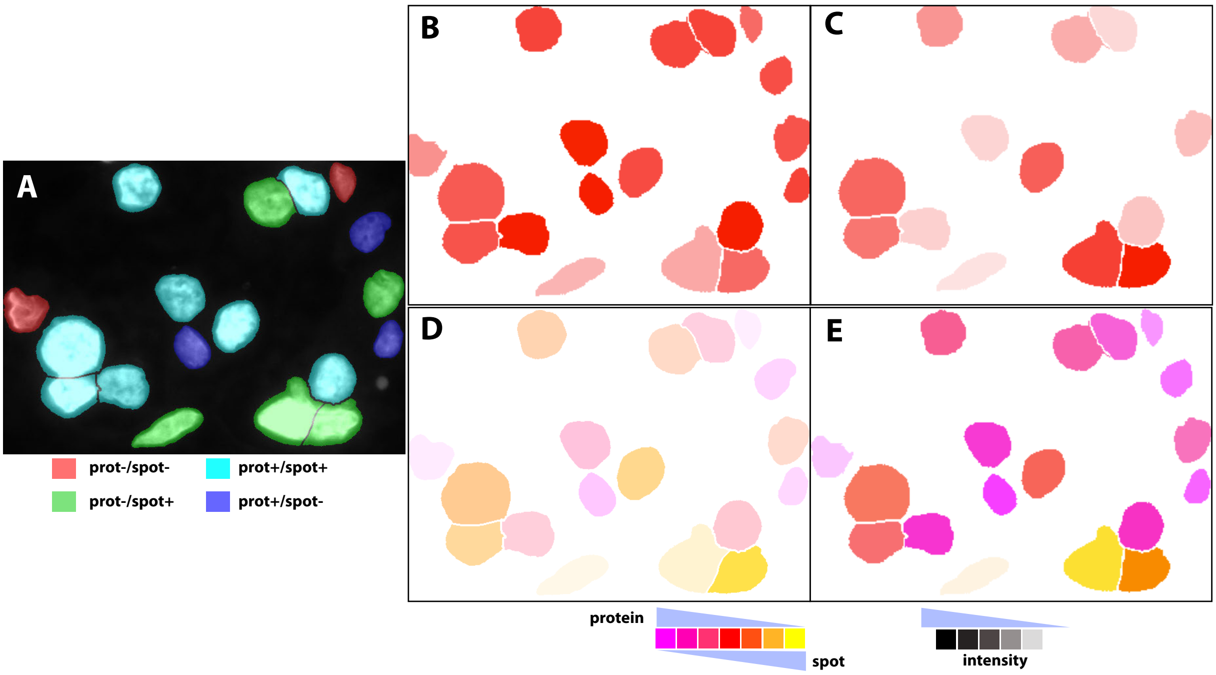

Figure 9: Topology maps attained from combing HER2 protein and spot data (A) classification based on two thresholds (B) Topology map attained in MATLAB environment by setting an intensity limit between [0, 2000] and spot area between [0, 800] (C) Topology map from GUI which scales data according to the range observed within the image. Good way of visualising variability but cannot be used as a global comparison

reset using the 'refresh button'. Cells are then classified and automatic assignments are shown in View: Classifier Cell Labels. When the 'Classifier Cell Labels' are displayed, the user can manually edit misclassified cells by directly labelling them with the correct cell class.

Classification may be performed simply to focus on tumour cells, but may also useful when an internal control is required. The following procedure will reproduce the results shown in Figure 10

```
1. View 'User Cell Labels'
   All cells should be red and thus, unlabelled.
2. We know that the long cell in the middle is 'normal'.
   Click on the 'fibroblast' icon an label them as such. Right click once finished.
3. Similarly, the large cells are cancer cells.
4. Click on 'Classify'
   Most of the larger rounder cells will appear as 'green tumour'.
   Cells with irregular morphology are labelled as 'yellow normal'.
5. Select classify by intensity and re-run
6. Using extra intensity information from other channels, some cells
which have low expression are now normal cells.
7. We know the cells in the upper left hand corner are normal.
Go to View: Classifier Cell Labels. Label them as fibroblasts. The
 classification as been manually curated for inconsistencies.
```

The option of using only morphological parameters or added intensity information is up to the user. Whilst the latter adds extra information, it is useful if the tumour population of interest displays somewhat heterogeneous behaviour in membrane staining or copy number. There is an option of adding an extra class to differentiate between two types of cancer cell, such as invasive compared to epithelial if required. A maximum of 4 classes are permitted.

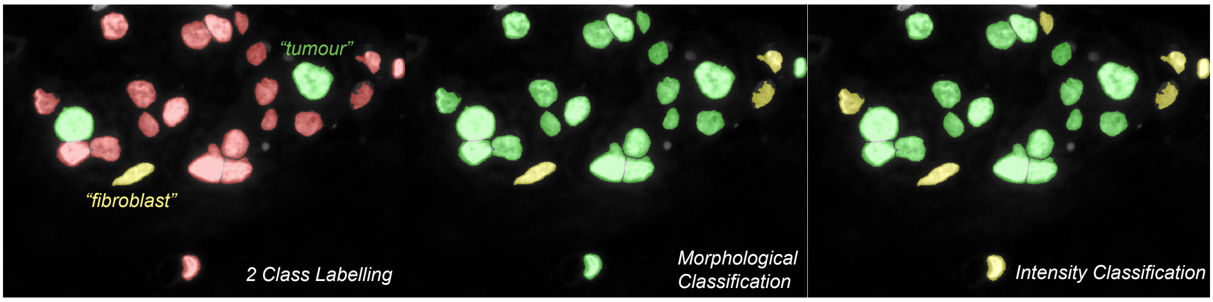

Figure 10: Classification using only morphological results, and with added intensity information.

8.9 Saving Progress

A number of options are available when saving your work.

1. Saving .mat file for further analysis in MATLAB
2. Saving summary .csv and .png images of the segmentation result
3. Saving a .mat progress file to continue analysis at a future date
4. Downloading directly to the MATLAB environment as a structure called `State`

The first and fourth option give the same information, however the latter directly downloads this information into MATLAB without being saved first. When imported to the user workspace, a single structure named 'State' will appear containing the following information:

The second option involves saving all segmentations to a series of png files for visual inspection, and summarising the properties in a .csv file. Segmentation results with corresponding cell numbers will be saved, as will heatmaps if they are generated. The properties of one particular cell will occupy a single row in the .csv file. Most of the information saved will be Areas, Intensities with and without background adjustment, and x-y co-ordinates.

The third option effectively saves all information inputted and recorded in GoIFISH for continued analysis at a later date, or in order to capture all intermediates obtained in the program. The filename will be of the form '_Pipeline.mat' and can be directly loaded into GoIFISH.

```
1. If using the GUI through MATLAB, click on ‘Download to MATLAB’.
  A structure called ‘State’ will appear in your workspace.
2. Check the last two tick boxes.
3. Save (button top left)
4. Saved folder will have segmented images with numbered cells, a txt
   file of cellular properties, and a Pipeline which can be loaded directly
```

Table 4: Summary of the outputs of the analysis in the `State` structure

Variable	Description
`imageStack`	Original Loaded Images
`CellForeground`	Map of Area considered to contain cells Area
`CellClassification`	Map of Final cell class labels
`FinalSegmentation`	The final segmentation maps for each channel
`EdgeView`	Outline of the final segmentation
`CellProperties`	A structural element containing information about each stain. Stains would be referred to as the type and channel it is present in. (ie. DAPI2 will indicate that channel 2 is DAPI). Properties including Area, perimeter, solidity, Mean Intensity and copy number are recorded.
`HeatMap`	Heatmaps generated (if performed) of relative intensity to one another
`background`	The regions indicated as background
`imProps`	Contains information about the stains and the resolution used
`watershed`	Parameters at which the watershed was run
`ACProps`	Active Contours properties
`SDProps`	Spot detection Properties

```
into the GUI at a later date.
```

9 Troubleshooting

9.1 Optimal Image Properties for Use

GoIFISH was developed for the analysis of high resolution images stained with IFISH. We have described the optimal types of images for use in GoIFISH in Table 5.

Table 5: Optimal properties of images for analysis in

	GUI	GoIFISHWrapper (Command Line)
File Format	.mat or .tiff	.tiff .zvi Use bio-formats to convert other formats
Image Size	12 megapixels for comfortable use	Theoretically unlimited
Number of stains	Up to 5. Must include DAPI	Unlimited but must include DAPI
Number of Cells	Under 1000 for comfortable use	Theoretically unlimited
Cell Size (px)	Optimally 60x magnification (2500-10000 px)<br>20x and 40x magnification also available (250-3000 px)	
Mosaic Images	Use `CollateMosaic` to stitch together. Final image should be less than 12 megapixels	

9.2 Quick Guide to Different Magnifications

The following parameters change depending on the magnification used. For instance, spot detection is more difficult to detect in lower resolution images. The following table gives a summary of the default values used at different magnifications. Note that these serve as a general guideline and may not be the best suited to your images.

Table 6: Parameters which are dependent on resolution

Parameter	60x	40x	20x
Expected Cell Size (px)	2500-10000	900-3000	250-1000
Minimum nuclear size (px)	300	200	100
Nearest neighbour distance (merging, in px)	75	55	25
Minimum Scaling in Membrane Detection	0.25	0.5	0.75
Minimum Spot Size	15	10	5
Spot Threshold	mean + 3 SD	mean + 2 SD	mean + 1 SD

9.3 Potential Issues

Under the following conditions, the default segmentation parameters may not be optimal. We have created a list of suggestions in these scenarios in Table 7.

Table 7: Difficulties which may be experienced using GoIFISH

Issue	Possible Solution
DAPI Segmentation	
Undersegmentation	Image may have poor contrast:<br>Try to improve the brightness and contrast, or lower the minimum threshold in segmentation (eg. from 0.14 to 0.1).<br>Can also perform seeded watershed.
Oversegmentation	Thresholds applied may need to be increased (eg. 0.14 - 0.3 to 0.2 - 0.36).<br>Check the resolution information is correct
Cell Boundaries Indistinguishable	Unfortunately, if a human has difficulty in this scenario, so will a computer!<br><br>Remove the indistinguishable clump and focus on the rest of the image
Cells are completely missed	The background threshold may be too high or the image has poor contrast
Spot Detection	
Clumps not detected	Turn off 'optimise morphology'.<br>May also need to reduce the intensity threshold applied. Determine the 'optimal' threshold and enter a slightly lower value for comparison
Regions of Autofluorescence detected	In low contrast images (eg. of centromeric regions) this may be a problem:<br><br>Turn on 'optimise morphology'<br>Ensure DAPI segmentation is correct: only spots within nuclei will be segmented
Missed Single Spots	Turn of 'optimise segmentation' and reduce the intensity required.<br>Try to also lower the Minimum spot size<br>Ensure DAPI segmentation is correct: only spots within nuclei will be segmented
GUI Issues	
GUI doesn't start	Note there is a 30 second opening time using MATLAB Runtime Compiler<br>If the user has MATLAB on the machine, it is highly recommended to process images in the MATLAB environment<br>Ensure the correct working directory is set in MATLAB if operating from there
Cannot load Image	Image must be .mat or .TIF image
Segmentation time too long	Is an issue if image is too large. We suggest limiting images to 1000 cells, and maximum image size of 3000 x 4000 pixels.<br>Images can be broken into subimages and processed individually<br>Try segmenting image using `GoIFISHWrapper` and then load into GUI
GUI Stalls	This may be an issue if in use for a long time or large image is being processed:<br>Try restarting the GUI
Error in manual editing	There may be a lag in large images:<br>Draw lines slowly<br>Ensure the 'escape' button is pressed after each segmentation<br>Ensure the correct 'view' is switched on. Background regions can only be selected under 'background view'
Other Issues	Please send example image, segmentation protocol and error message to author

References

[1] Melissa Linkert, Curtis T. Rueden, Chris Allan, Jean-Marie Burel, Will Moore, Andrew Patterson, Brian Loranger, Josh Moore, Carlos Neves, Donald Macdonald, Aleksandra Tarkowska, Caitlin Sticco, Emma Hill, Mike Rossner, Kevin W. Eliceiri, and Jason R. Swedlow. Metadata matters: access to image data in the real world. *J Cell Biol*, 189(5):777–782, May 2010.

[2] Fernand Meyer and Serge Beucher. Morphological segmentation. In *Journal of visual communication and image representation* [2], pages 21–46.

[3] Pierre Soille. *Morphological image analysis: principles and applications*. Springer-Verlag New York, Inc., 2003.

[4] T. F. Chan and L. A. Vese. Active contours without edges. *IEEE Trans Image Process*, 10(2):266–277, 2001.

[5] Shawn Lankton and Allen Tannenbaum. Localizing region-based active contours. *IEEE Trans Image Process*, 17(11):2029–2039, Nov 2008.
