# Supplementary material for: GoIFISH: a system for the quantification of single cell heterogeneity from IFISH images
Source: Genome Biol. 2014 Aug 26;15(8):442. doi: 10.1186/s13059-014-0442-y (PMC4167144; doi:10.1186/s13059-014-0442-y)
Supplement: Additional file 3 — Supplementary information. Supplementary information includes detailed experimental methods for the staining of images, and all code for the generation of plots and statistical analyses in R. [file 13059_2014_442_MOESM3_ESM.pdf]

Supplementary Information for GoIFISH: A system for the quantification of single cell heterogeneity from IFISH images

Trinh et al, 2014

July 24, 2014

Contents

1 Detailed description of methods **3**
1.1 Immunostaining Protocol . . . 3
1.2 FISH probes preparation . . . 3

2 Description of Data attained from images: Comparative study between GoIFISH, Cellprofiler and Columbus **5**
2.1 Precision Recall Data . . . 5
2.2 Single Cell Measurements . . . 8

3 Cell Detection: Precision and Recall. Capturing the average of an image **15**
3.1 Nuclear Detection . . . 15
3.2 HER2 membrane Detection . . . 18
3.2.1 Performance . . . 18
3.3 Spot Detection . . . 21
3.3.1 Single Spots . . . 21
3.3.2 ERBB2 . . . 25
3.4 Figure2 . . . 30

4 Biological comparison: Positivity and Intensity **35**
4.1 ER Intensity Comparisons . . . 35
4.1.1 Searching for trend in individual samples . . . 35
4.1.2 Anova to test for direct comparability . . . 38
4.1.3 Combining All Samples . . . 41
4.2 HER2 protein expression . . . 45
4.2.1 HER2 positivity and negativity: Is there a difference between these samples? . . . 45
4.2.2 Grouping all samples and comparing positivity vs negativitiy . . . 50

4.2.3 HER2 membrane completeness 54
4.2.4 Combining all samples together 58
4.2.5 Looking at Higher Order Moments 60

5 Accuracy in Spot Detection: Measuring intra-tumour heterogeneity 73
5.1 Correlating copy number between raw and counted values 73
5.2 Direct Comparison to area 86
5.3 Combining Area and Intensity 93

6 Inferring Sample Diversity 96
6.1 ER and HER2 96
6.2 HER2 copy number vs protein 99

7 Software Performance 104
7.1 Classification Performance 104
7.1.1 Differentiating between luminal and epithelial cells 104
7.1.2 Tumour vs Stromal Cells 107
7.2 Background Intensity Variation 111
7.2.1 Heterogeneity in background regions 114
7.3 Benchmarking Differences in Manual Editing 116

8 Session info: R-packages and their versions used for this analysis 120

1 Detailed description of methods

1.1 Immunostaining Protocol

The FFPE tissue blocks were sectioned 3-4 μM thick and put on superfrost plus slides (Thermo scientific, art nr J1800MNZ) and baked at 60C° for 45 min and 37C° over night (ON). The sections were deparaffinizated for 2x10 min in xylene, follwed by denaturation in 2x 3 min Abs. EtOH, 2x3 min in 95 % EtOH anddH2O before heat induced antigene retrieval in 1x Citrate buffer, pH 6 for 40 min in a steamer, cooled down for 30 min at RT. Washed 2x3 min in dH2O and 3 min in 1x PBS. The tissue was digested with 0,000005 % pepsin (diluted in 0,02 M HCL) for 10 min at 37 degree, followed by 2x3 min wash in dH2O and 3 min in 1x PBS. 10 min in RT with 10 prosent goat serum (GS), followed by primary C-erbB2 antibody (NCL-L-CB11, Novocastro) incubation, 1/100 diluted in 5 % GS for 60 min (See Table 1 for list of antibodies). Washed 2x 3 min in PBST (1x PBS+ 0,001 % tween 40). 30 min incubation with secondary biotinylated antibody (goat anti mouse IgG,(biotin conjugated),M30115, Life Technologies), 1/100 dilution in PBS for 30 min. Primary ER antibody incubation 1/100 diluted in 5 % GS, incuabted for 60 min, washed 2x 3 min in PBST before 30 min incubation with fluorescence labelled secondary antibodies (streptavidin Alexa fluore 488 goat anti mouse IgG(Y1)(S-11223, Life Technologies), and Alexa Fluor 594 anti mouse(A-11005, Life Technologies) (1/100 diluted in 10 pros% GS)). Washed 3x3 min in PBST, 3 min in dH2O.All antibodies were incubated in humidiation chamber at RT. The samples were post fixed in fresh made ice cold carnoy's fix (36 ml abs EtOH and 12 ml acetic acid), washed 3 min in dH2O and rehydrated in graded alcohol and air dried before adding the FISH probes and coverslides before denaturation at 75C° for 2 min and hybridized at 37C° ON in a hybridizer (DAKO hybridizer). After hybridization the coverslides were removed and the slides washed for 2 min in post hybridization buffer 1 (0,4 xSSC/0,3 % igepal) at RT, followed by 2 min post hybridization buffer 1 at 74C° for exact 2 min, and 2 min in post hybridization buffer 2 (2xSSC/0,1% igepal) and 3 min in 2xSSC, air dried and mounted with DAPI counterstain and microscoped and photographed with 25 z-stacks in a Axiovision M1 microscope.

1.2 FISH probes preparation

The bacterial artificial chromosomes (BAC) of interest were selected from the UCSC Genome Browser and purchased as plasmid inserts in E.*Coli* from Source Bioscience (RP11-170N19,RP11-909L10, RP11-94L15) (See Table 2). The bacteria were cultivated according to the manufacturer instructions. The plasmids were isolated with plasmid maxi kit (Qiagen) and labelled with fluorescent dUTPs by nick translation (Nick translation kit, Invitrogen); HER2 was labelled with spectrum Orange (Abbott) dUTP and CEP17 with HyPer5-dCTP (GE Healthcare dUTP).

For a stronger signal two overlapping BAC probes were used. To validate the correct position of the probes, the probes were labelled with new fluorescences and hybridized together with the PathVysion HER-2 DNA probe kit from Abbott, approved for clinical

use. The BAC probes overlapped nicely with Abbotts commercial probes(data not shown).

Table 1: Antibody information

Antibodies	product	manufacturer
c-erbB2 oncoprotein	NCL-L-CB11	Novocastro/Leica
Estrogen Receptor	NCL-L-ER-6F11	Novocastro/Leica
Goat anti mouse, IgG (biotin)	M30115	Life technologies
Alexa Fluor Goat Anti mouse IgG	A-11005	Life technologies
Streptavidin Alexa Fluor 488 conjugate	S-11223	Life technologies

Table 2: BAC probe information

Name	Clone ID	Location	Fluorescence	manufacturer
Cent17	RP11-720N19	17q11.1	Spectrum orange	Abbott
Cent17	RP11-909L10	17q11.1-11.2	Spectrum orange	Abbott
HER2	RP11-94L15	17q12	Cy-5	GE healthcare
HER2	RP11-909L6	17q12	Cy-5	GE healthcare

2 Description of Data attained from images: Comparative study between GoIFISH, Cellprofiler and Columbus

The following packages are required for statistical analysis in R:

```
## Source the functions required to transform data
source("functions/readData.R")
source("functions/functions.R")
library("MASS")
library("knitr")
library("clinfun")
library("RColorBrewer")
library("plotrix")
library("scales")

##
## Attaching package:  'scales'
##
## The following object is masked from 'package:plotrix':
##
##     rescale

library("cluster")
library("e1071")
library("ggplot2")
library("lattice")
library("latticeExtra")

##
## Attaching package:  'latticeExtra'
##
## The following object is masked from 'package:ggplot2':
##
##     layer
```

2.1 Precision Recall Data

The first thing we want to look at is Precision-Recall Data. Given a particular folder name, we run `read.PrecisionRecall` to collate the information into a list of matrices:

```
tempfile = read.PrecisionRecall("data/New_PR_Tests")
summary(tempfile)
```

```
##           Length Class  Mode
## PR_Cent17  6     -none- list
## PR_DAPI    6     -none- list
## PR_HER2    8     -none- list
## PR_ERBB2  12     -none- list
```

```
load("data/Collated_PR_Data.RData")
summary(PR_ERBB2)
```

```
##                    Length Class      Mode
## CellProfiler_clust 12     data.frame list
## CellProfiler       12     data.frame list
## ColumbusA_clust    12     data.frame list
## ColumbusA          12     data.frame list
## ColumbusB_clust    12     data.frame list
## ColumbusB          12     data.frame list
## ColumbusO_clust    12     data.frame list
## ColumbusO          12     data.frame list
## GOIFISH_clust      12     data.frame list
## GOIFISH            12     data.frame list
## ManualGF_clust     12     data.frame list
## ManualGF           12     data.frame list
```

```
summary(PR_ERBB2$CellProfiler)
```

```
##     NCorrect         UnderSeg        OverSeg          FalseNeg
##  Min.   : 11.0   Min.   : 2.0   Min.   : 0.00   Min.   :  8.0
##  1st Qu.: 38.0   1st Qu.: 6.0   1st Qu.: 0.00   1st Qu.: 25.5
##  Median : 55.5   Median : 9.0   Median : 1.00   Median : 42.0
##  Mean   : 56.0   Mean   :19.5   Mean   : 5.70   Mean   : 74.5
##  3rd Qu.: 67.2   3rd Qu.:21.2   3rd Qu.: 1.75   3rd Qu.:123.2
##  Max.   :120.0   Max.   :81.0   Max.   :46.00   Max.   :236.0
##     FalsePos      AreaOverlap       AreaDiff        SolidDiff
##  Min.   :10.0   Min.   :0.159   Min.   :0.415   Min.   :0.852
##  1st Qu.:19.5   1st Qu.:0.348   1st Qu.:1.193   1st Qu.:0.914
##  Median :22.0   Median :0.446   Median :1.905   Median :0.930
##  Mean   :32.1   Mean   :0.494   Mean   :1.937   Mean   :0.938
##  3rd Qu.:45.0   3rd Qu.:0.573   3rd Qu.:2.006   3rd Qu.:0.967
##  Max.   :66.0   Max.   :0.994   Max.   :5.766   Max.   :1.001
##    PerimDiff          APDiff         LargeArea       SmallArea
##  Min.   :0.674   Min.   :0.697   Min.   : 0.0   Min.   : 0
##  1st Qu.:1.456   1st Qu.:0.851   1st Qu.: 7.5   1st Qu.: 0
##  Median :1.774   Median :0.924   Median : 9.5   Median : 0
##  Mean   :1.973   Mean   :1.002   Mean   :13.4   Mean   : 2
```

```
##  3rd Qu.:2.323   3rd Qu.:1.041   3rd Qu.:22.2   3rd Qu.: 0
##  Max.   :4.431   Max.   :1.825   Max.   :31.0   Max.   :19
```

This returns an obkect which contains 4 Precision-Recall objects, relating to the 4 main segmentations used (DAPI, HER2 membrane, cent17 and ERBB2). The function also returns this as a list.

If we look at each of the Precision-Recall objects, it is a list of matrices with names describing how the data was attained. `PR_ERBB2` is considered both as individual spots as well as clusters of spots. Each method will have the following parameters:

NCorrect : The number of cells which have been correctly identified

UnderSeg : Number of cells which have been undersegmented. These are "false negatives"

OverSeg : Number of oversegmented cells. These are "false positives"

FalseNeg : Number of cells which have been completely missed (non segmentation error)

FalsePos : Cells which are detected by image analysis software but not present in the original image. These may be artefacts, or cells with too low intensity in the image.

AreaOverlap : Comparison of how well a segmented cell overlaps with the corresponding cell in the true standard. A score of 1 indicates they overlap 100%, and a score of 0 indicates poor overlap. We expect samples with high numbers of binarisation errors to have a lower overlap for instance.

AreaDiff : Calculated as the difference between areas in the image and the true standard. (Nsegmented/Ntrue) We could ideally see samples around 1 for perfect segmentation. Samples with high values have larger cell boundaries in the automated image, and negative values indicate the size is smaller.

PerimDiff : Difference in perimeter, calculated as the ratio between the segmented cell and the gold standard. Ideally, this value should be 1. Note that some cells might have the same area but different perimeters.This suggests that the segmented image has cells of the correct area, however, their outlines would differ greatly, accounting for the binarisation errors or lower area overlap.

SolidDiff : Difference in solidity, calculated as the ratio between the segmented cell and the gold standard. Ideally, this value should be 1. This looks at how well the segment fits into a sphere, or solid a segment is.

APDiff : Difference in the area to perimeter ratio between the segmented cell and the gold standard. Ideally, this value should be one.

LargeArea : Item is much larger (2x or more) compared to the actual cell size.

SmallArea : Item is much smaller (quarter of size or less) compared to the actual cell size.

2.2 Single Cell Measurements

Single cell measurements were collected from Columbus, CellProfiler and GOIFISH. This includes protein intensity measurements, as well as spot areas and intensities. These are mapped to a true standard which is pathologist scored. We can collate this information using `read.IFISHInfo`

```
Output=read.IFISHInfo("data/Protein_expression_intensities/",
                      "data/CellManualScores/")

## Warning:  - not meaningful for factors
## Warning:  - not meaningful for factors

load("data/Compiled_CellData.RData")
summary(Output)

##                        Length Class  Mode
## ManIFISH               10     -none- list
## ColumbusC_CN           10     -none- list
## ColumbusC_Protein      10     -none- list
## CP_CN                  10     -none- list
## CP_Protein             10     -none- list
## ColumbusM_CN           10     -none- list
## ColumbusM_Protein      10     -none- list
## GF_CN                  10     -none- list
## GF_Protein             10     -none- list
## Columbus_Protein       10     -none- list
## Columbus_CN            10     -none- list
## ManGF_CN               10     -none- list
## ManGF_Protein          10     -none- list
## ManGF_Prot_notscored 10     -none- list

summary(ManIFISH)

##      Length Class      Mode
## 6361 20     data.frame list
## 6370 20     data.frame list
## 7350 20     data.frame list
## 7360 20     data.frame list
## 7364 20     data.frame list
## 7417 20     data.frame list
## 7435 20     data.frame list
## 7588 20     data.frame list
## 7619 20     data.frame list
## 7641 20     data.frame list
```

```r
summary(ManIFISH$'6361')
```

```
##      CellNo        GOIFISH      GOIFISHUnder   ManGoIFISH
##  Min.   : 1.0   Min.   : 2.0   Min.   :1     Min.   : 1.0
##  1st Qu.:10.5   1st Qu.:13.0   1st Qu.:1     1st Qu.:12.5
##  Median :20.0   Median :31.0   Median :1     Median :34.0
##  Mean   :20.0   Mean   :27.9   Mean   :1     Mean   :29.9
##  3rd Qu.:29.5   3rd Qu.:43.0   3rd Qu.:1     3rd Qu.:46.5
##  Max.   :39.0   Max.   :57.0   Max.   :1     Max.   :56.0
##                                NA's   :20    NA's   :4
##  ManGoIFISHUnder   ColumbusC       ColCUnder       CellProfiler
##  Mode:logical     Min.   : 1.0   Min.   :0.000   13      : 2
##  NA's:39          1st Qu.:14.0   1st Qu.:0.000   58      : 2
##                   Median :21.0   Median :0.000   12      : 1
##                   Mean   :19.5   Mean   :0.205   14      : 1
##                   3rd Qu.:25.0   3rd Qu.:0.000   17      : 1
##                   Max.   :34.0   Max.   :1.000   (Other):30
##                   NA's   :2                      NA's   : 2
##     CP_Under        ColumbusM    ColMUnder      ERBB2.CN.true
##  Min.   :0.000   29      : 2   Min.   :0.000   Min.   :1.00
##  1st Qu.:0.000   51      : 2   1st Qu.:0.000   1st Qu.:1.00
##  Median :0.000   1       : 1   Median :0.000   Median :2.00
##  Mean   :0.216   11      : 1   Mean   :0.135   Mean   :1.85
##  3rd Qu.:0.000   14      : 1   3rd Qu.:0.000   3rd Qu.:2.00
##  Max.   :1.000   (Other):30   Max.   :1.000   Max.   :4.00
##  NA's   :2       NA's   : 2   NA's   :2
##  cent17.CN.true ERBB2.score cent17.score ER.score  HER2.prot.score
##  Min.   :1.00   scat:39     scat:39      -ve :14   -ve: 0
##  1st Qu.:1.00   clus: 0     clus: 0      weak: 8   +ve:39
##  Median :1.00   mix : 0     mix : 0      mod : 6
##  Mean   :1.56                            +ve :11
##  3rd Qu.:2.00
##  Max.   :3.00
##
##  HER2.membrane.score   ERBB2.CN2      cent17.CN2
##  absent  : 0         Min.   :1.00   Min.   :1.00
##  broken  :10         1st Qu.:1.00   1st Qu.:1.00
##  complete:29         Median :2.00   Median :1.00
##                      Mean   :1.85   Mean   :1.56
##                      3rd Qu.:2.00   3rd Qu.:2.00
##                      Max.   :4.00   Max.   :3.00
##
```

The `Output` of the function contains a Manually Scored Matrix, and a series of matrices

which describes the method used and whether the output is IF (Protein) or FISH (CN).

Let's have a quick look at the ManIFISH parameters:

CellNo Cell number assigned in manual segmentation

GOiFISH Corresponding cell number in GOiFISH

GOiFISHUnder Is the cell poorly segmented in GoIFISH.

ColumbusC Corresponding cell number in Columbus

ColCUnder index of [0,1] to indicate whether the cell is undersegmented (1 is yes)

CellProfiler : Cell number of corresponding CellProfiler segmentation

CP_Under : index of [0,1] to indicate whether the cell is undersegmented (1 is yes)

ColumbusM : Cell number of corresponding ColumbusC segmentation CURRENTLY EMPTY

ColMUnder : index of [0,1] to indicate whether the cell is undersegmented (1 is yes)

ERBB2.CN.true : Manual ERBB2 copy number counts

cent17.CN.true : Manual cent17 copy number counts

ERBB2.score : Arrangement of ERBB2 spots: 1 is scattered, 2 is clustered and 3 is a mix

cent17.score : Arrangement of cent17 spots: 1 is scattered, 2 is clustered and 3 is a mix

ER.score : ER nuclear intensity: 0 is neg, 1 is weak, 2 moderate, 3 is strong

HER2.prot.score : HER2 protein intensity: 0 is negative, 1 is positive

HER2.membrane.score : Continuity of HER2 membrane: either "absent", "broken" or "complete".

ERBB2.CN2 : ERBB2 copy number - amplified is given a value of 22 for analysis

cent17.CN2 : cent17 copy number - amplified is given a value of 22 for analysis

Using the previous command read.IFISHInfo, we also get protein and copy number information per cell

```
summary(Output$GF_Protein$"6361")
```

```
##     GOIFISH         CellNo      GOIFISHUnder ER.score  HER2.prot.score
##  Min.   : 2.0   Min.   : 1.0   Min.   :1     -ve :14   -ve: 0
##  1st Qu.:13.0   1st Qu.:10.5   1st Qu.:1     weak: 8   +ve:39
##  Median :31.0   Median :20.0   Median :1     mod : 6
##  Mean   :27.9   Mean   :20.0   Mean   :1     +ve :11
##  3rd Qu.:43.0   3rd Qu.:29.5   3rd Qu.:1
##  Max.   :57.0   Max.   :39.0   Max.   :1
##                                NA's   :20
##  HER2.membrane.score   DAPI_Area       DAPIPerim      DAPIInt
##  absent  : 0         Min.   : 1908   Min.   :177   Min.   : 948
##  broken  :10         1st Qu.: 6655   1st Qu.:370   1st Qu.:1219
##  complete:29         Median : 8375   Median :407   Median :1396
##                      Mean   : 8040   Mean   :400   Mean   :1368
##                      3rd Qu.: 9540   3rd Qu.:444   3rd Qu.:1480
##                      Max.   :13863   Max.   :578   Max.   :1836
##
##   DAPI_backAdj    DAPINucInt      HER2Area      HER2Solidity
##  Min.   : 770   Min.   : 438   Min.   : 2219   Min.   :0.702
##  1st Qu.:1041   1st Qu.: 772   1st Qu.: 7450   1st Qu.:0.834
##  Median :1218   Median : 969   Median : 9944   Median :0.863
##  Mean   :1191   Mean   : 933   Mean   : 9332   Mean   :0.863
##  3rd Qu.:1303   3rd Qu.:1070   3rd Qu.:10903   3rd Qu.:0.899
##  Max.   :1658   Max.   :1407   Max.   :15950   Max.   :0.954
##
##  HER2Perimeter  HER2cytoInt    HER2memInt   HER2backAdj      ER_Int
##  Min.   :190   Min.   :204   Min.   :232   Min.   :125   Min.   :113
##  1st Qu.:410   1st Qu.:235   1st Qu.:281   1st Qu.:174   1st Qu.:141
##  Median :493   Median :253   Median :309   Median :202   Median :234
##  Mean   :480   Mean   :258   Mean   :310   Mean   :203   Mean   :236
##  3rd Qu.:525   3rd Qu.:268   3rd Qu.:341   3rd Qu.:235   3rd Qu.:308
##  Max.   :688   Max.   :384   Max.   :419   Max.   :313   Max.   :518
##
##    ER_backAdj       ERNucInt         HER2_var       HER2_range
##  Min.   : 19.1   Min.   :-41.80   Min.   : 2423   Min.   : 49.2
##  1st Qu.: 46.8   1st Qu.:  7.91   1st Qu.: 3284   1st Qu.: 57.3
##  Median :139.6   Median : 47.89   Median : 5026   Median : 70.9
##  Mean   :141.8   Mean   : 72.43   Mean   : 5422   Mean   : 71.7
##  3rd Qu.:213.3   3rd Qu.:112.64   3rd Qu.: 6838   3rd Qu.: 82.7
##  Max.   :423.8   Max.   :260.34   Max.   :11136   Max.   :105.5
##
##  HER2_skewness        HER2_SD
##  Min.   :-0.6439   Min.   : 49.2
##  1st Qu.: 0.0856   1st Qu.: 57.3
```

```
##  Median : 0.4704   Median : 70.9
##  Mean   : 0.4041   Mean   : 71.7
##  3rd Qu.: 0.6842   3rd Qu.: 82.7
##  Max.   : 1.4777   Max.   :105.5
##
```

```
summary(Output$CP_CN$"6361")
```

```
##   CellProfiler     CellNo        CP_Under     cent17.CN.true cent17.score
##  13      : 2    Min.   : 1.0   Min.   :0.000   Min.   :1.00   scat:39
##  58      : 2    1st Qu.:10.5   1st Qu.:0.000   1st Qu.:1.00   clus: 0
##  12      : 1    Median :20.0   Median :0.000   Median :1.00   mix : 0
##  14      : 1    Mean   :20.0   Mean   :0.216   Mean   :1.56
##  17      : 1    3rd Qu.:29.5   3rd Qu.:0.000   3rd Qu.:2.00
##  (Other):30     Max.   :39.0   Max.   :1.000   Max.   :3.00
##  NA's    : 2                   NA's   :2
##    cent17.CN2   cent17_CN_spot   cent17_Int     cent17_Area
##  Min.   :1.00   Min.   :0.00   Min.   :0.014   Min.   : 20
##  1st Qu.:1.00   1st Qu.:1.00   1st Qu.:0.020   1st Qu.: 27
##  Median :1.00   Median :1.00   Median :0.024   Median : 31
##  Mean   :1.56   Mean   :1.43   Mean   :0.035   Mean   : 48
##  3rd Qu.:2.00   3rd Qu.:2.00   3rd Qu.:0.050   3rd Qu.: 69
##  Max.   :3.00   Max.   :4.00   Max.   :0.076   Max.   :121
##                 NA's   :2      NA's   :8       NA's   :8
##  ERBB2.CN.true  ERBB2.score   ERBB2.CN2     ERBB2_CN_spot    ERBB2_Int
##  Min.   :1.00   scat:39     Min.   :1.00   Min.   :0.00   Min.   :0.078
##  1st Qu.:1.00   clus: 0     1st Qu.:1.00   1st Qu.:1.00   1st Qu.:0.110
##  Median :2.00   mix : 0     Median :2.00   Median :2.00   Median :0.142
##  Mean   :1.85               Mean   :1.85   Mean   :2.51   Mean   :0.205
##  3rd Qu.:2.00               3rd Qu.:2.00   3rd Qu.:3.00   3rd Qu.:0.272
##  Max.   :4.00               Max.   :4.00   Max.   :8.00   Max.   :0.557
##                                            NA's   :2      NA's   :8
##    ERBB2_Area
##  Min.   : 26.0
##  1st Qu.: 61.2
##  Median : 93.0
##  Mean   :117.3
##  3rd Qu.:163.5
##  Max.   :294.0
##  NA's   :5
```

Let's look at the protein information first: There are 10 items (Corresponding to the 10 samples used), and contains the following features (Manually scored features are also present for ease in analysis later)

CellProfiler Cell No. This column would be "ColumbusC" "GoIFISH" etc in the respective analyses

CellNo Manual cell number

CP_Under is the cell undersegmented (and to be excluded from later analysis)

DAPI_Area area of nuclei

DAPI_Int average DAPI intensity within the nuclei

CytoArea area of cytoplasmic region

HER2cytoInt HER2 average Intensity of the cytoplasmic region.

ER.score Manual score

HER2.prot.score Manual score

HER2.membrane.score Manual score

ER_Int ER average intensity within the nucleus

HER2memInt HER2 average Intensity of the membrane.

HER2backAdj/ ERbackAdj : Only in GoIFISH. Intensity after performing a baseline background subtraction.

ERNucInt : ER nuclear intensity after performing a background detection per cell.

In the copy number data, we have the following descriptions:

CellProfiler Cell No. This column would be "ColumbusC" "GoIFISH" etc in the respective analyses

CellNo Manual cell number

CP_Under is the cell undersegmented (and to be excluded from later analysis)

cent17.CN.true or cent17.CN2 Manual: see ManIFISH

cent17.score Manual: see ManIFISH

cent17_CN_spot : measured number of cent 17 spots

cent17_Int : average intensity of the cent17 spots

cent17_Area : Total area occupied within a cell by cent17 spots

ERBB2.CN.true or ERBB2.CN2 Manual: see ManIFISH

ERBB2.score Manual: see ManIFISH

ERBB2_CN_spot : number of ERBB2 spots

ERBB2_Int : average ERBB2 intensity of spots

ERBB2_Area : Total area occupied within a cell by ERBB2 spots

3 Cell Detection: Precision and Recall. Capturing the average of an image

In this section, we will assess the precision and recall of each segmentation method. These results are attained from MATLAB. We make a comparison between 3 different methods: Columbus, Cell Profiler and `GoIFISH` (using both default settings and manually edited result) to a hand-segmented gold standard.

3.1 Nuclear Detection

```
# Load existing Precision-Recall data.  If this hasn't been loaded
load("data/Collated_PR_Data.RData")
# see what properties is available
colnames(PR_DAPI$ColumbusC)

##  [1] "NCorrect"    "UnderSeg"    "OverSeg"     "FalseNeg"    "FalsePos"
##  [6] "AreaOverlap" "AreaDiff"    "SolidDiff"   "PerimDiff"   "APDiff"
## [11] "LargeArea"   "SmallArea"
```

When we load the above file, we should see the precision-recall outputs using various softwares. The tables show a number of parameters including the number of correctly segmented cells, the number of over and under segmented cells, cells which have been completely missed in the segmentation or those which do not appear in the gold standard. In addition, there are deviation values for a number of morphological properties from the true standard.

First, we can make a plot of precision and recall for all the datasets:

```
## (Example) calculate precision and recall :
testPrecision = with(PR_DAPI$ColumbusC, NCorrect/(NCorrect + OverSeg + FalsePos))
testRecall = with(PR_DAPI$ColumbusC, NCorrect/(NCorrect + UnderSeg + FalseNeg))
testFScore = 2 * testPrecision * testRecall/(testPrecision + testRecall)
## Run the Precision_Recall Function for all the samples
PR_DAPI = sapply(names(PR_DAPI), function(x) Calc.PRF(PR_DAPI[[x]], "DAPI"),
    USE.NAMES = T, simplify = F)
## Merge these values together and plot the result
Precision.Recall.Plot(PR_DAPI[c(-4)])  # Sample 4 is the optimal Columbus Samples
```

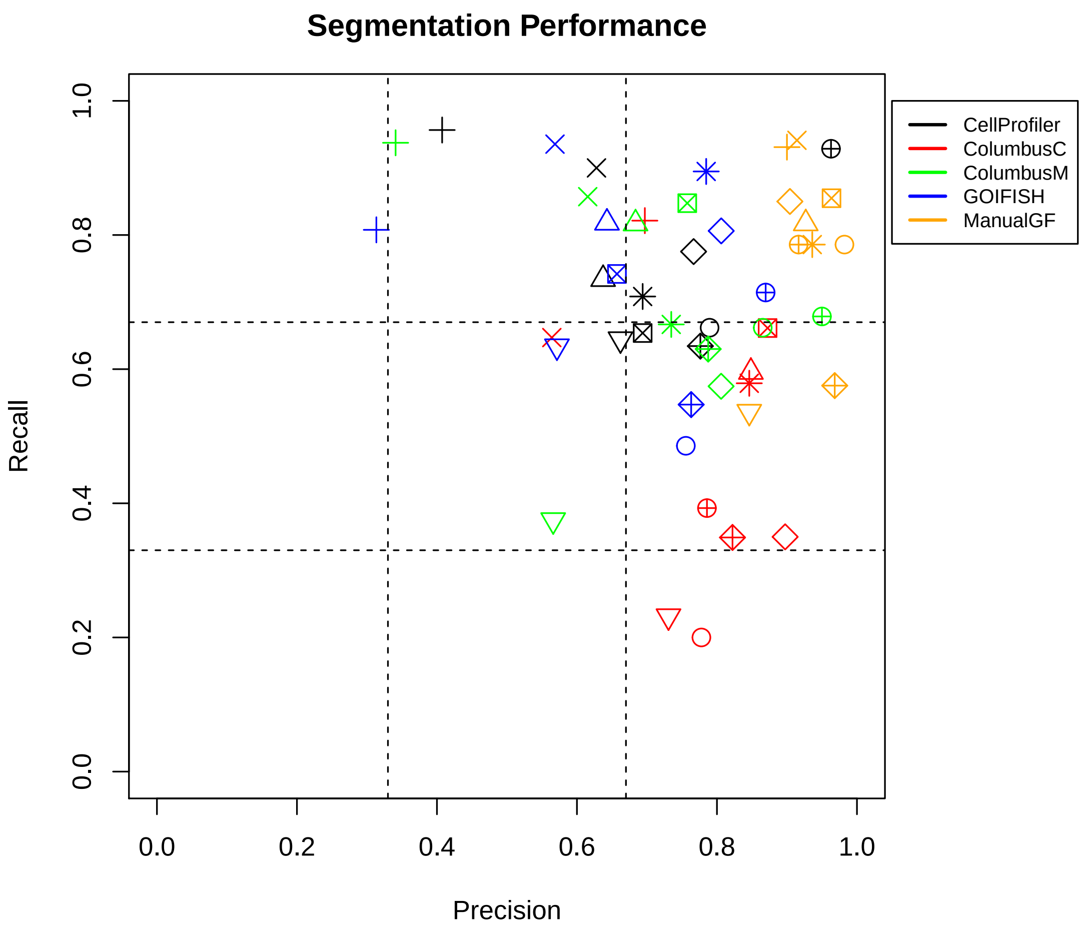

Here we see that CellProfiler outperforms the other methods. Columbus M on average performs better than Columbus C. CellProfiler and GoIFISH also perform similarly. We can construct independent Precision, Recall Line graphs which highlight the performance of each sample.

```
# Plot the Precision, Recall and F Score per sample
PRF.LinePlot(PR_DAPI[c(1:3, 5:6)])
```

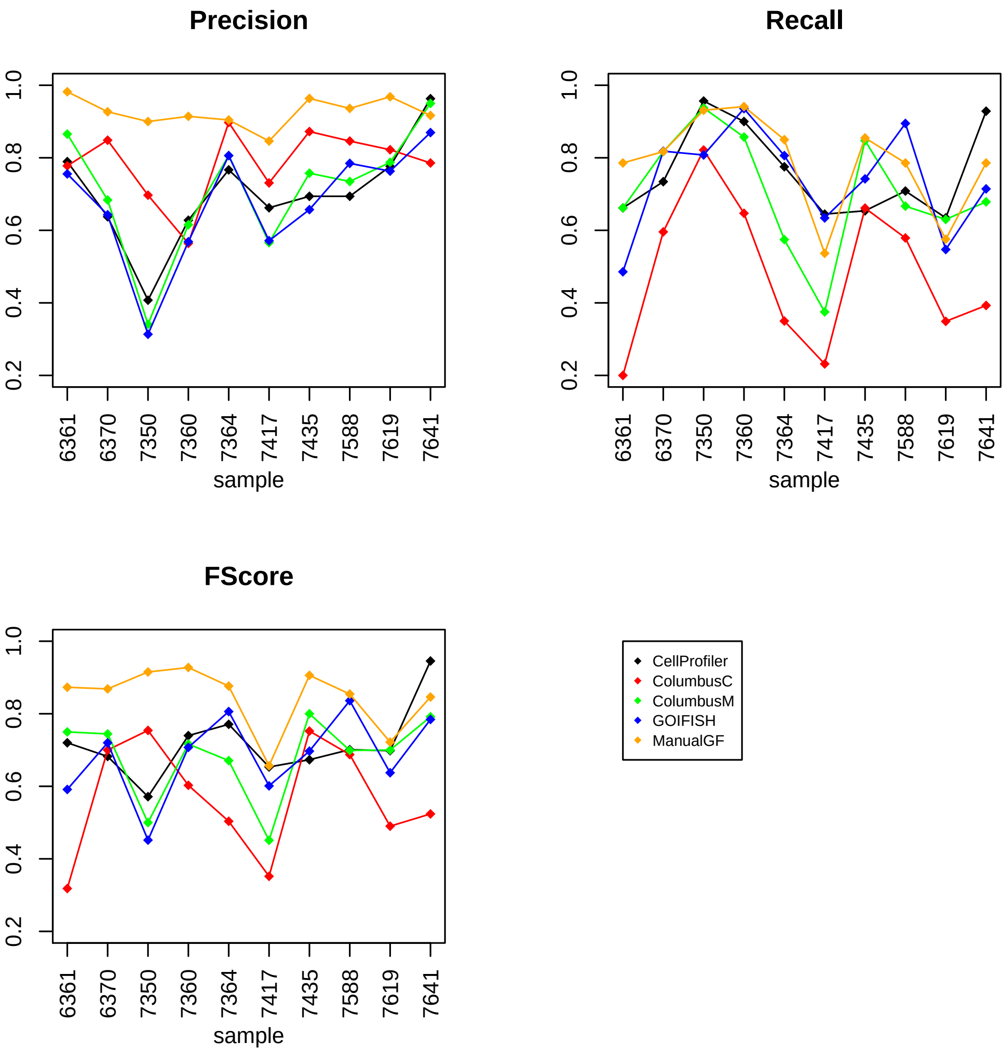

We see that method C has better precision, however, method M has better Recall. There is one sample, 7350, which had an improved output using method C compared to M. GoIFISH on average has good recall, and performs the best in samples 7364 and 7588.

The next thing we can test is the distributions of morphological parameters:

```
Morph.Variation.Plot(PR_DAPI, ylim = c(-1.5, 1.5))
```

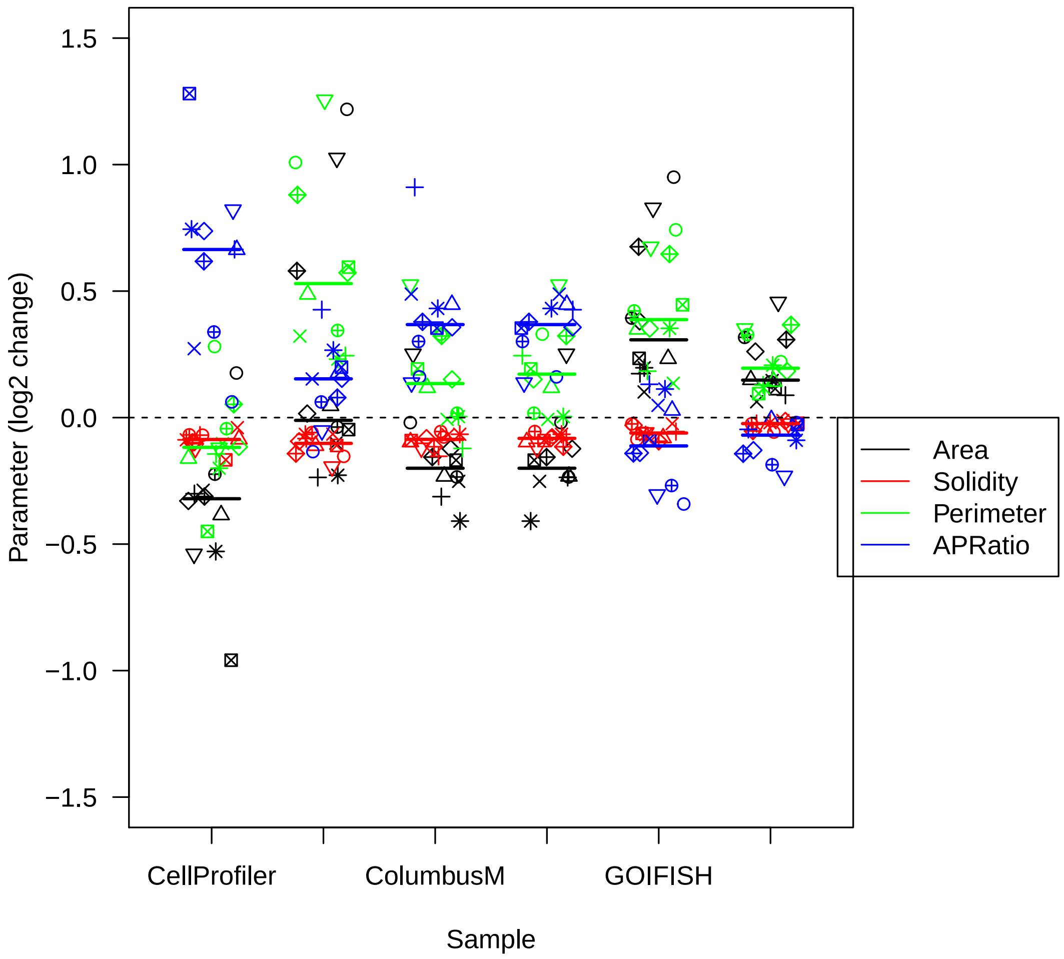

We see here that the perimeter differences are smaller in Columbus M, but the area different is slightly smaller in ColumbusC. GoIFISH on average has a larger segmentation with area and perimeters being higher, however, the Area-Perimeter ratio is closer to 0 compared to the other segmentation techniques.

3.2 HER2 membrane Detection

3.2.1 Performance

First we load the outputs of HER2 PR-Testing There are currently 4 items representing the 4 methods in columbus (A, B, C, F), CellProfiler and `GoIFISH`. The inputs of each matrix is in the same format as in DAPI.

As above, we calculate the precision and recall. Here, we do not consider non-

segmentation related false positives as the absence of distinct boundaries will impede our ability to segment all membranes in the image.

```
# Precision, Recall calculations for HER2 protein outlines
PR_HER2 = sapply(names(PR_HER2), function(x) Calc.PRF(PR_HER2[[x]], "HER2"),
    USE.NAMES = T, simplify = F)
```

We see that most samples have high precision, but lower recall. Looking at sample by sample basis:

```
# Plot the precision/recall distributions
Precision.Recall.Plot(PR_HER2[-6])
```

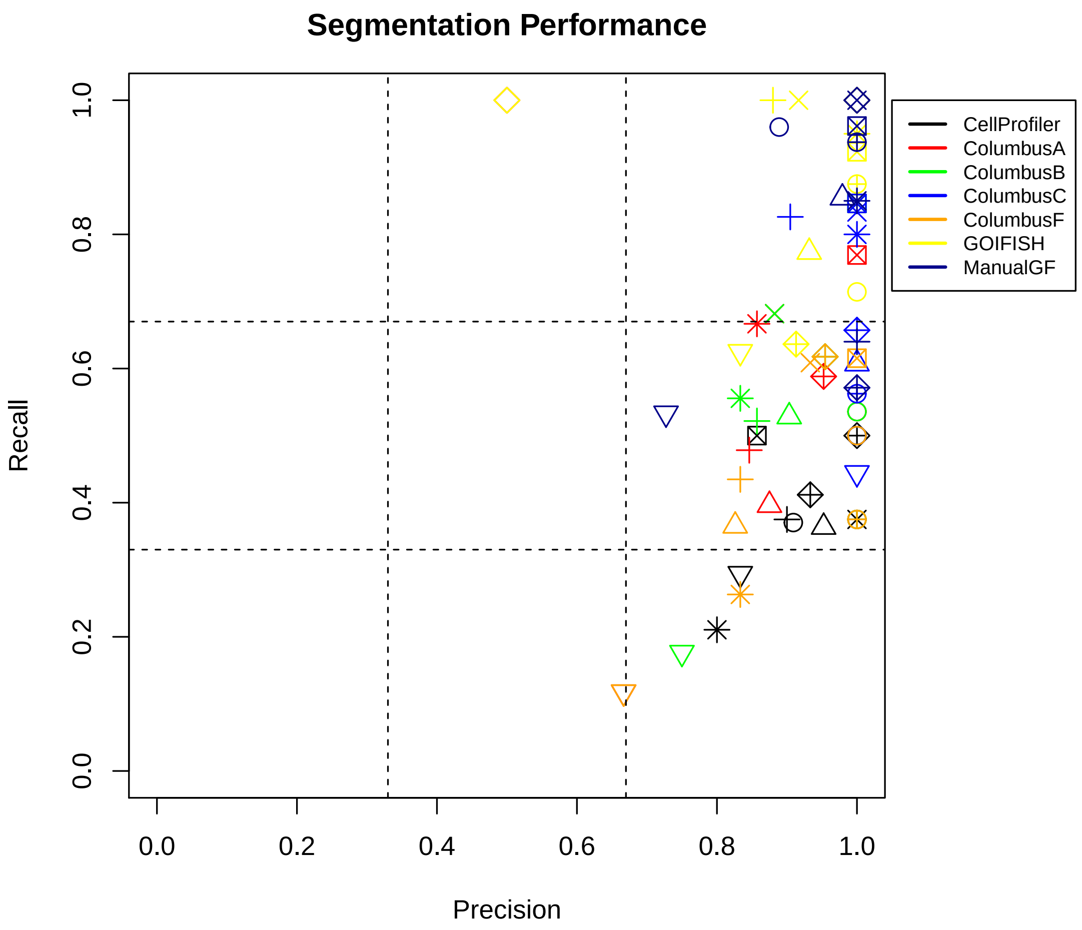


```
# Plot the Precision, Recall and F Score per sample
PRF.LinePlot(PR_HER2[-6])
```

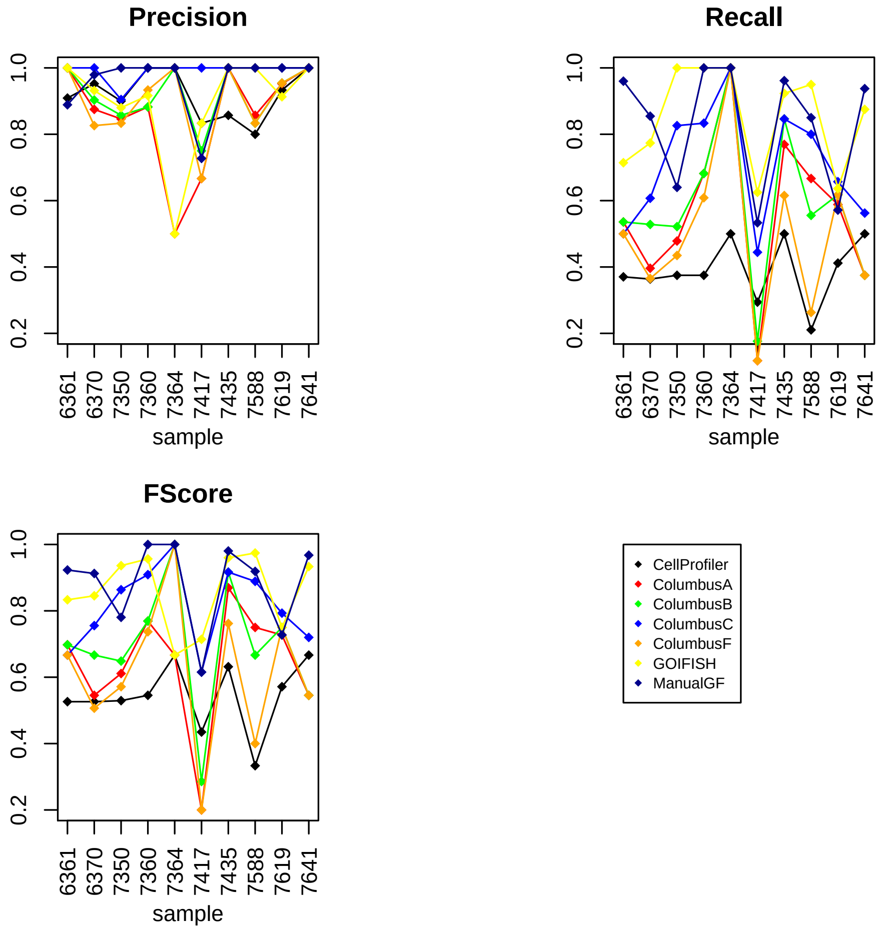

Check the Morphological Parameters:

```
Morph.Variation.Plot(PR_HER2, ylim = c(-2, 2))
```

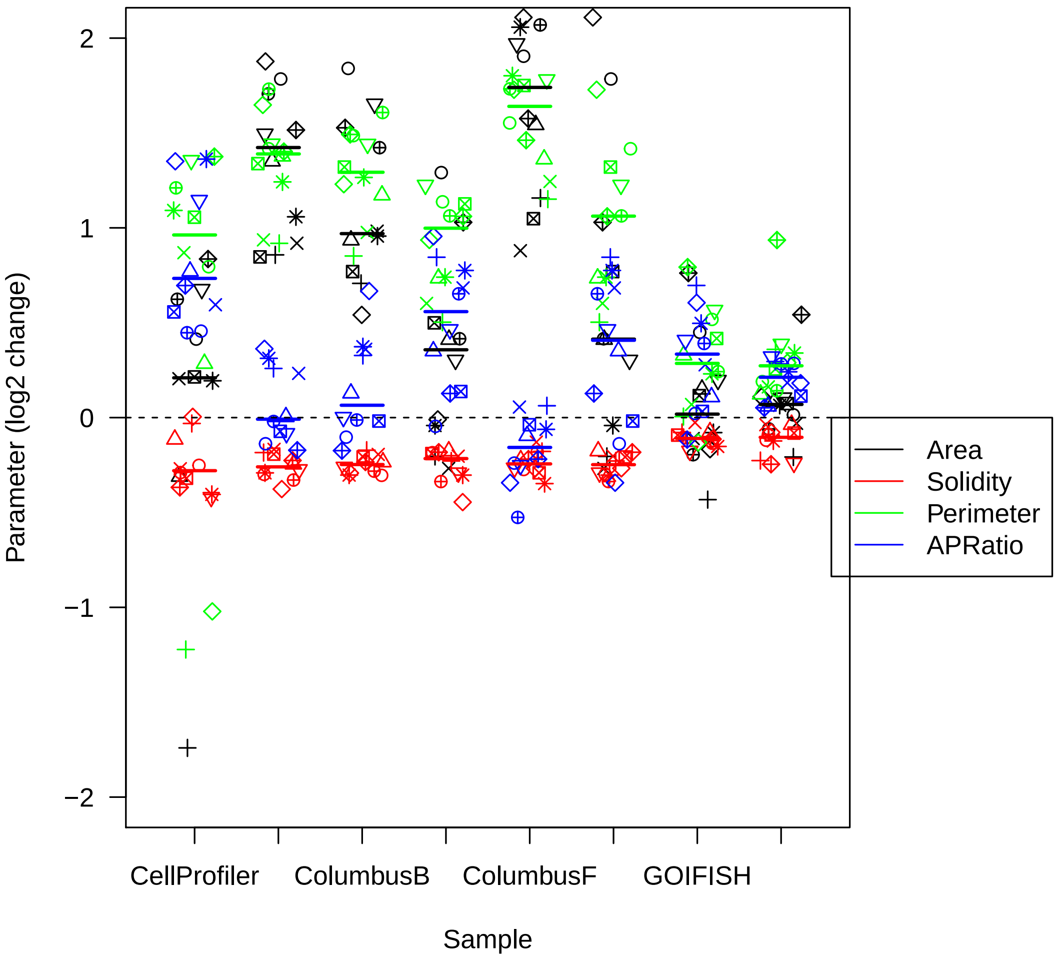

From this result, we can summarise which is the best segmentation to use for the Columbus samples as follows:

3.3 Spot Detection

3.3.1 Single Spots

```
# Precision, Recall calculations for HER2 protein outlines
PR_Cent17 = sapply(names(PR_Cent17), function(x) Calc.PRF(PR_Cent17[[x]], "DAPI"),
    USE.NAMES = T, simplify = F)
```

We the results here are very image dependent - results from the same image often cluster together. ()Eg, most of the diamonds perform quite poorly - this is probably a

Table 3: Optimal Paramters for Columbus Segmentation

Sample	DAPI Method	Cytoplasmic Method	Spot
6361	M	A	A
6370	M	C	A
7350	C	C	A
7360	M	C	B
7364	M	F	A
7417	M	C	A
7435	M	B	A
7588	M	C	A
7619	M	C	B
7641	M	C	A

reflection of the image quality rather than the segmentation accuracy).

```
# Plot the precision/recall distributions
Precision.Recall.Plot(PR_Cent17[-4])
```

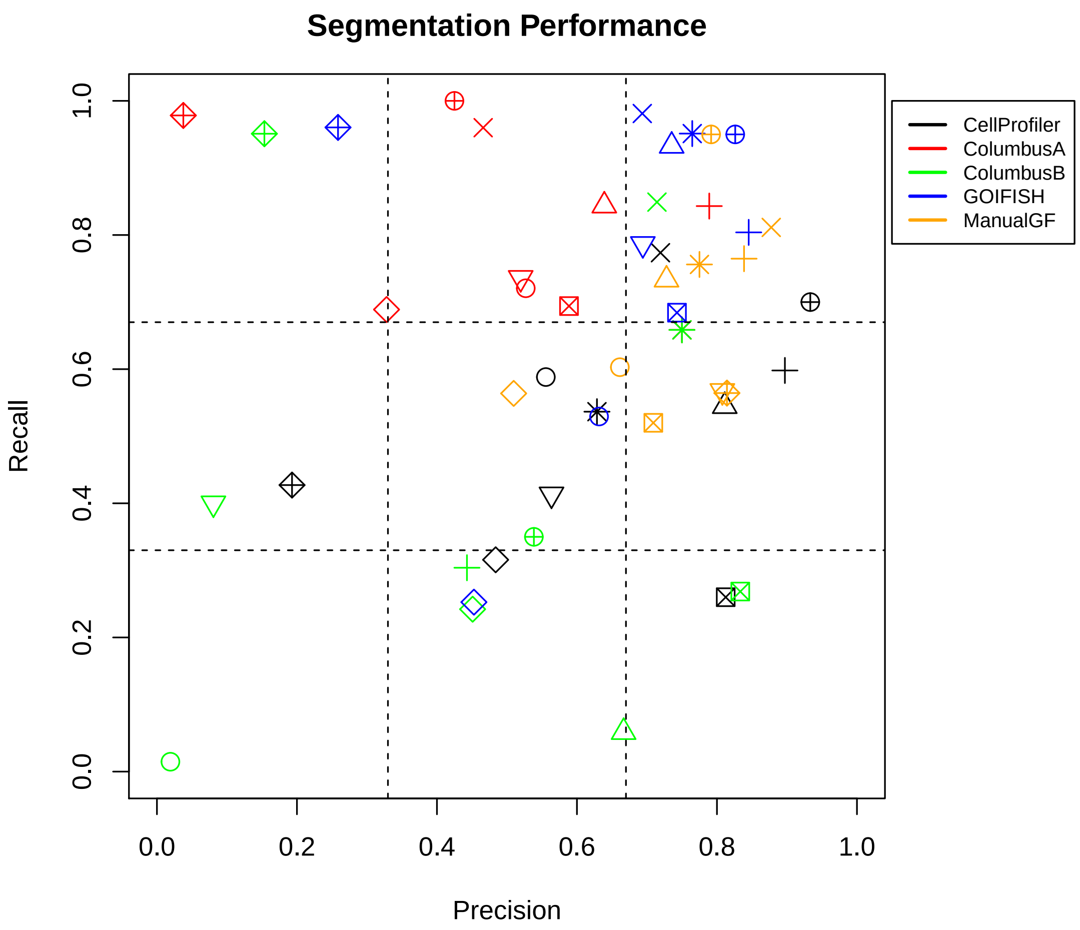


```
# Plot the Precision, Recall and F Score per sample
PRF.LinePlot(PR_Cent17[-4])
```

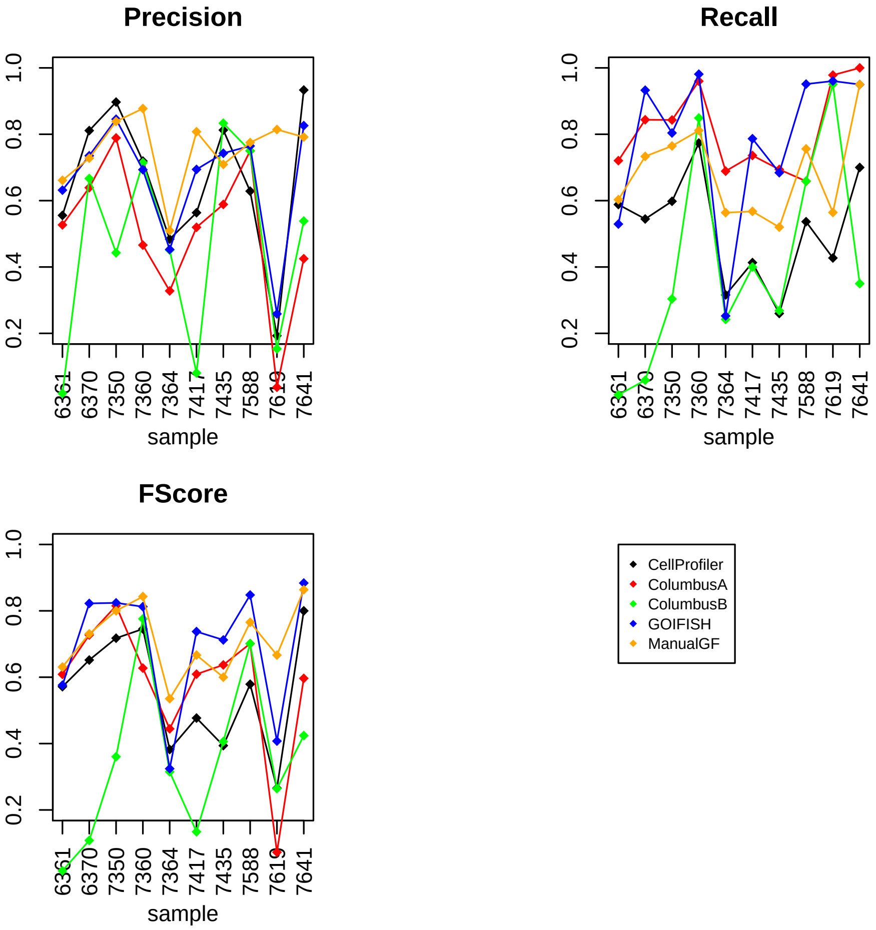

Check the Morphological Parameters:

```
Morph.Variation.Plot(PR_Cent17, ylim = c(-2, 2))
```

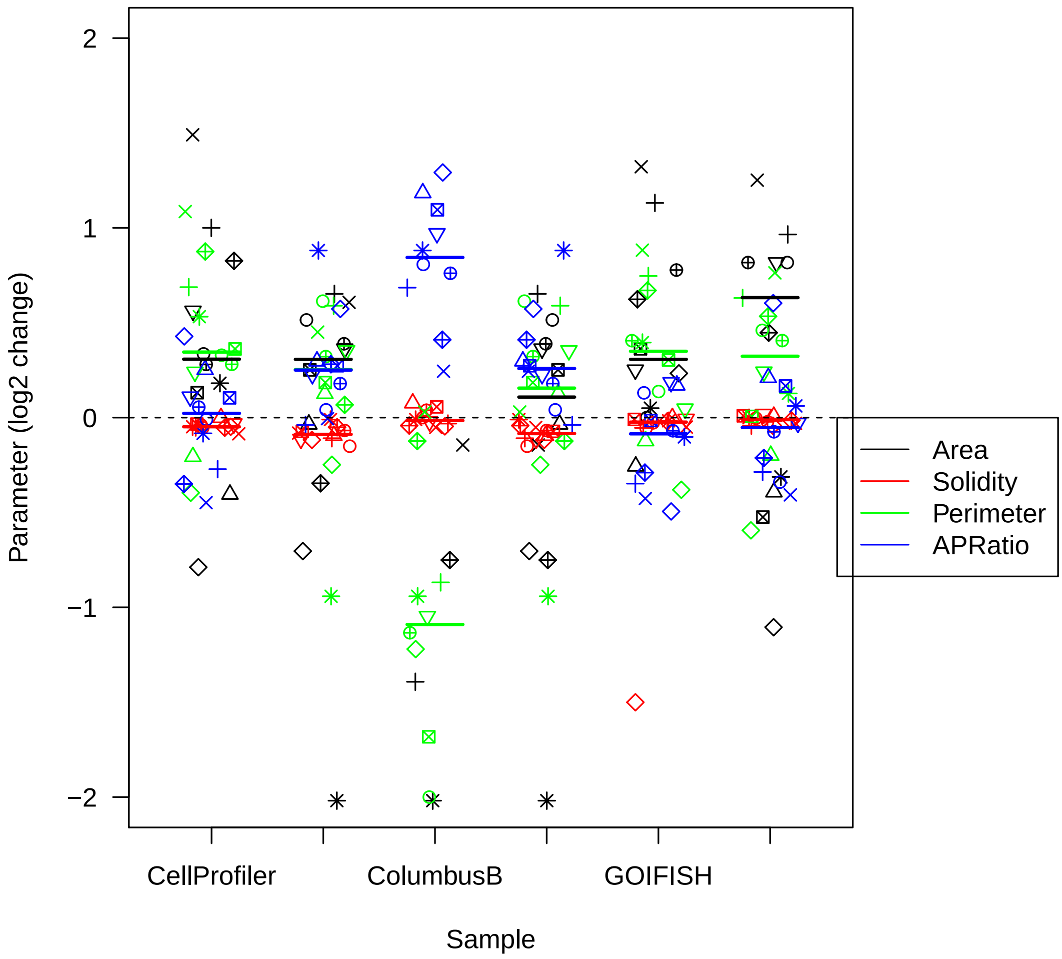

3.3.2 ERBB2

Individual spots in a cluster may be difficult to count by eye - especially on a maximum projected image. To counteract this problem, we looked at the precision-recall of individual spots (if present), and of the clusters themselves:

```
# Precision, Recall calculations for HER2 protein outlines
PR_ERBB2 = sapply(names(PR_ERBB2), function(x) Calc.PRF(PR_ERBB2[[x]], "Spot"),
    USE.NAMES = T, simplify = F)
```

```
# Plot the precision/recall distributions
x1 = grep("clust", names(PR_ERBB2))
Precision.Recall.Plot(PR_ERBB2[-x1])
```

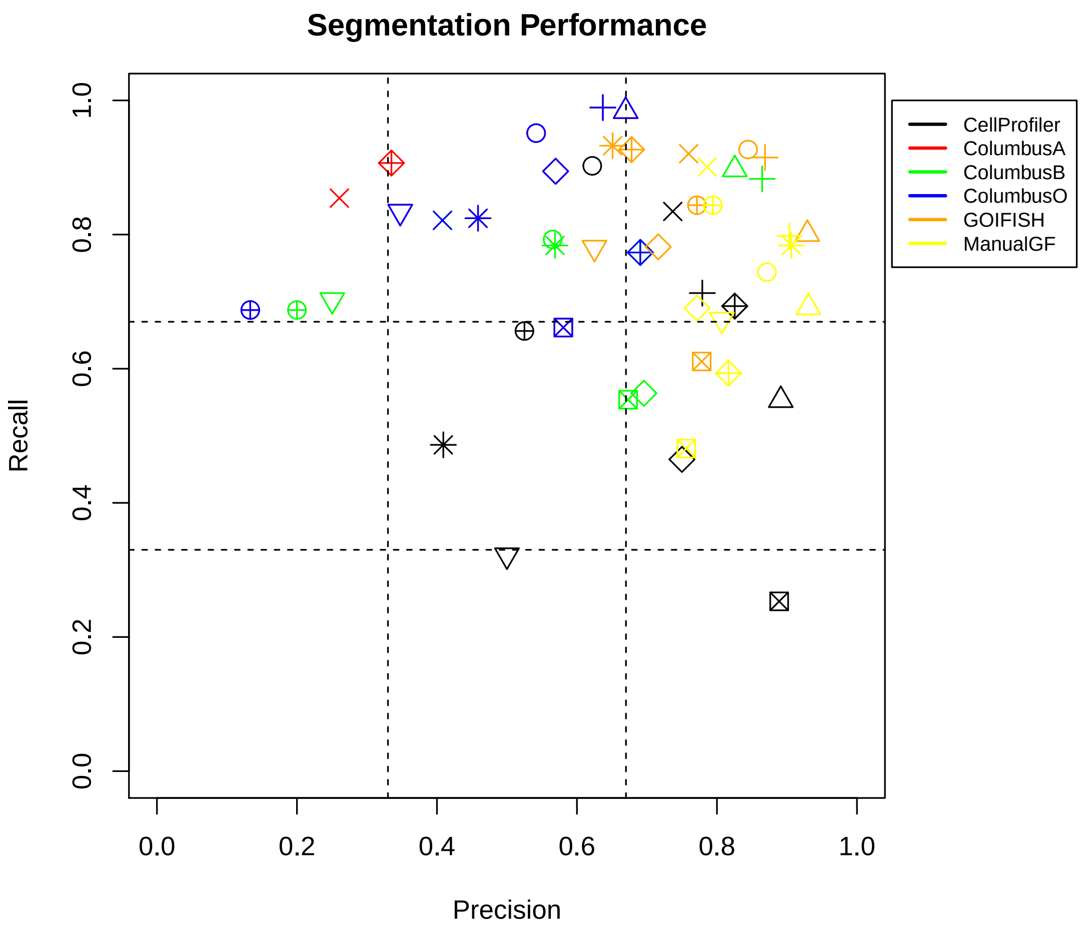


```
Precision.Recall.Plot(PR_ERBB2[x1])
```

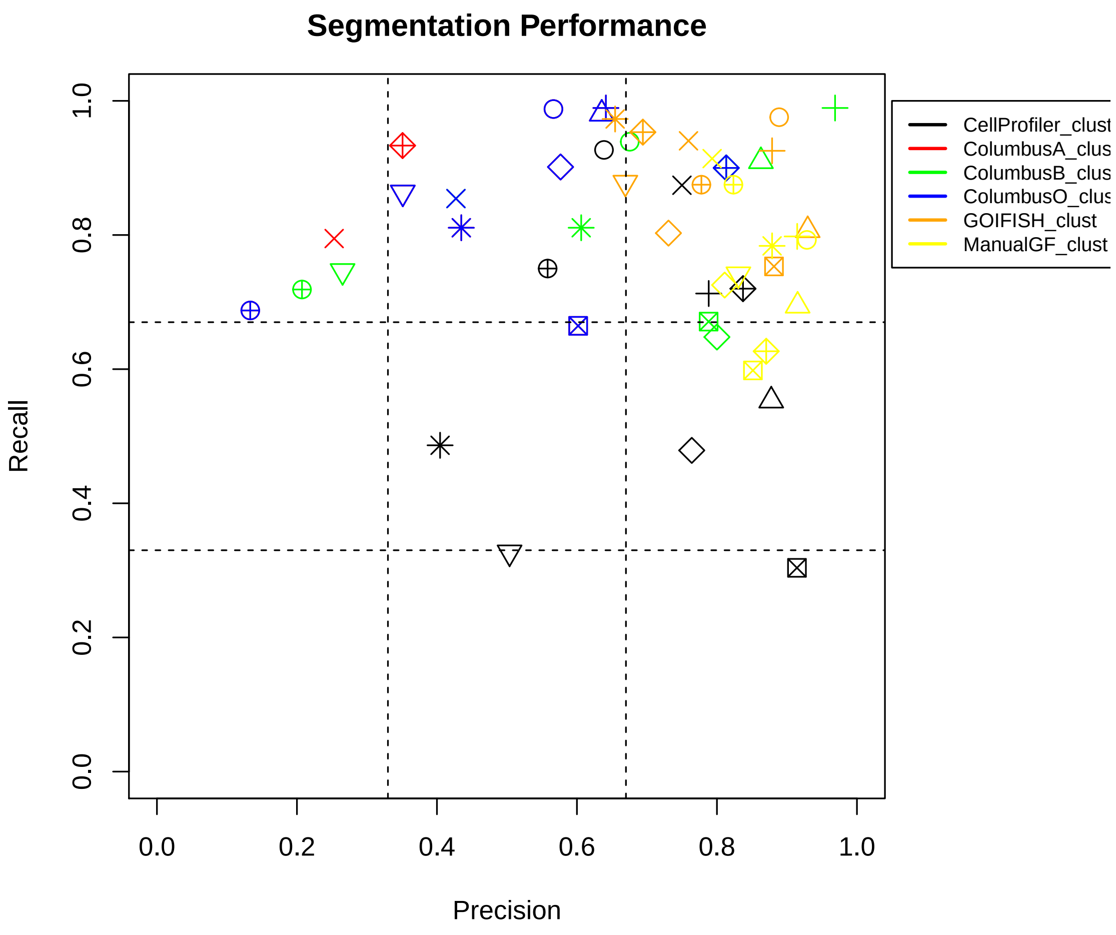


```
# Plot the Precision, Recall and F Score per sample
PRF.LinePlot(PR_ERBB2[-x1])
```

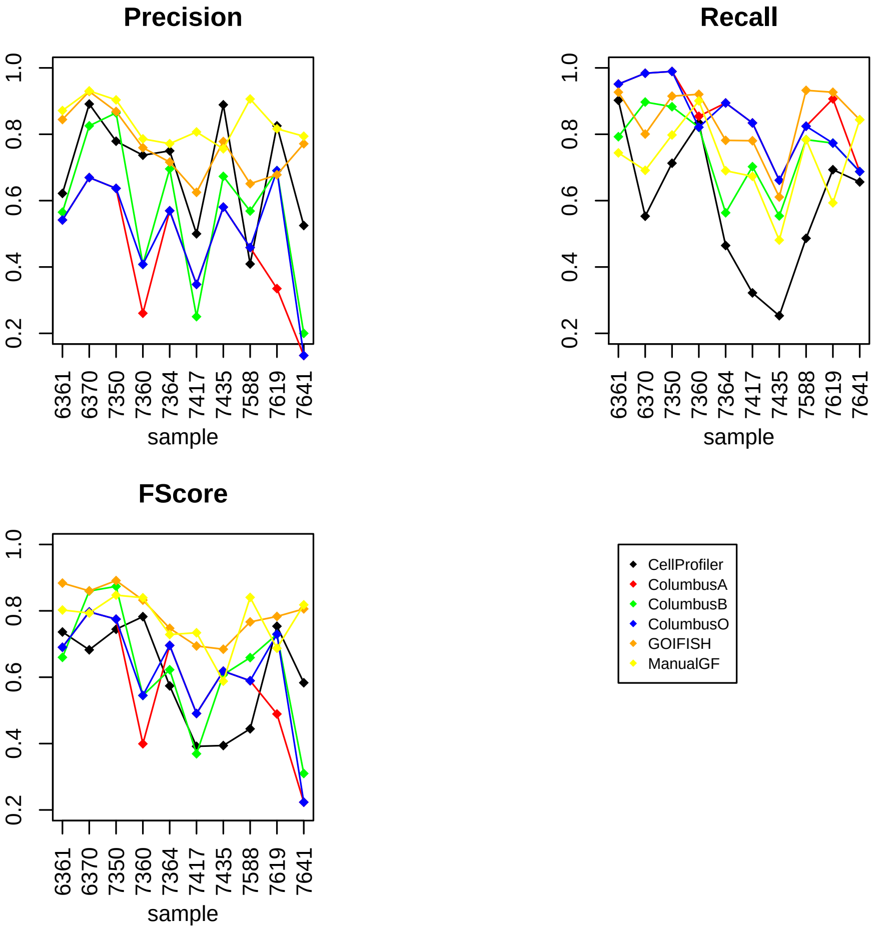


```
PRF.LinePlot(PR_ERBB2[x1])
```

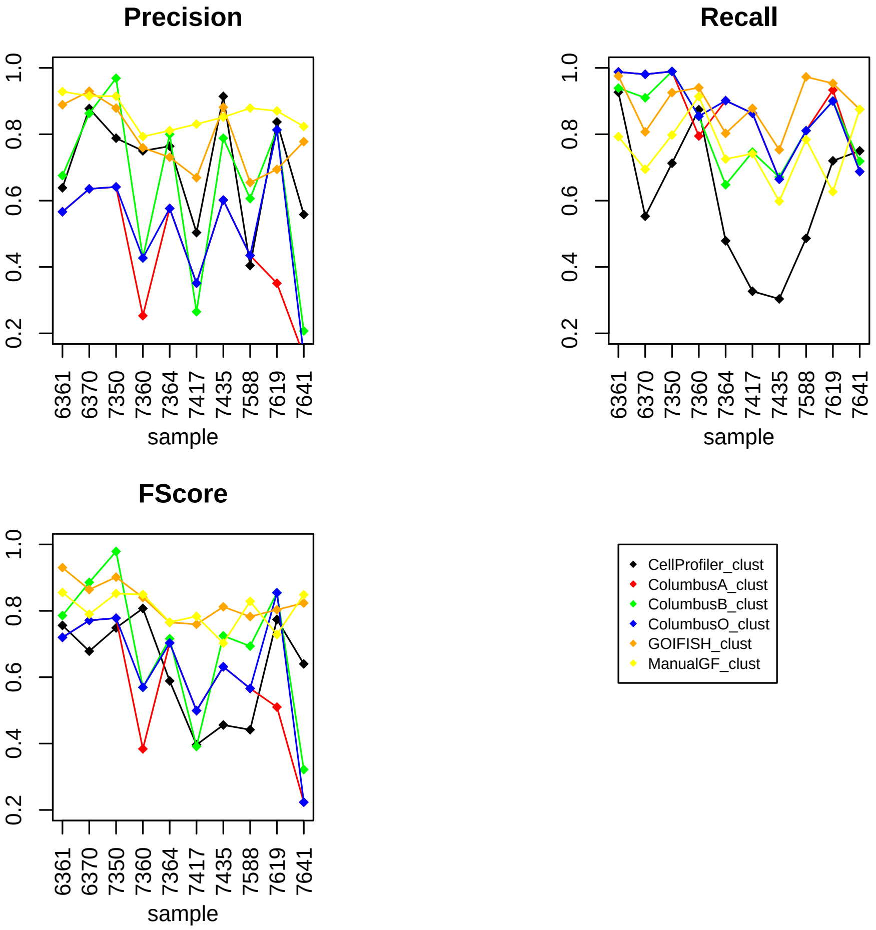

Check the Morphological Parameters:

```
Morph.Variation.Plot(PR_Cent17, ylim = c(-3, 1))
```

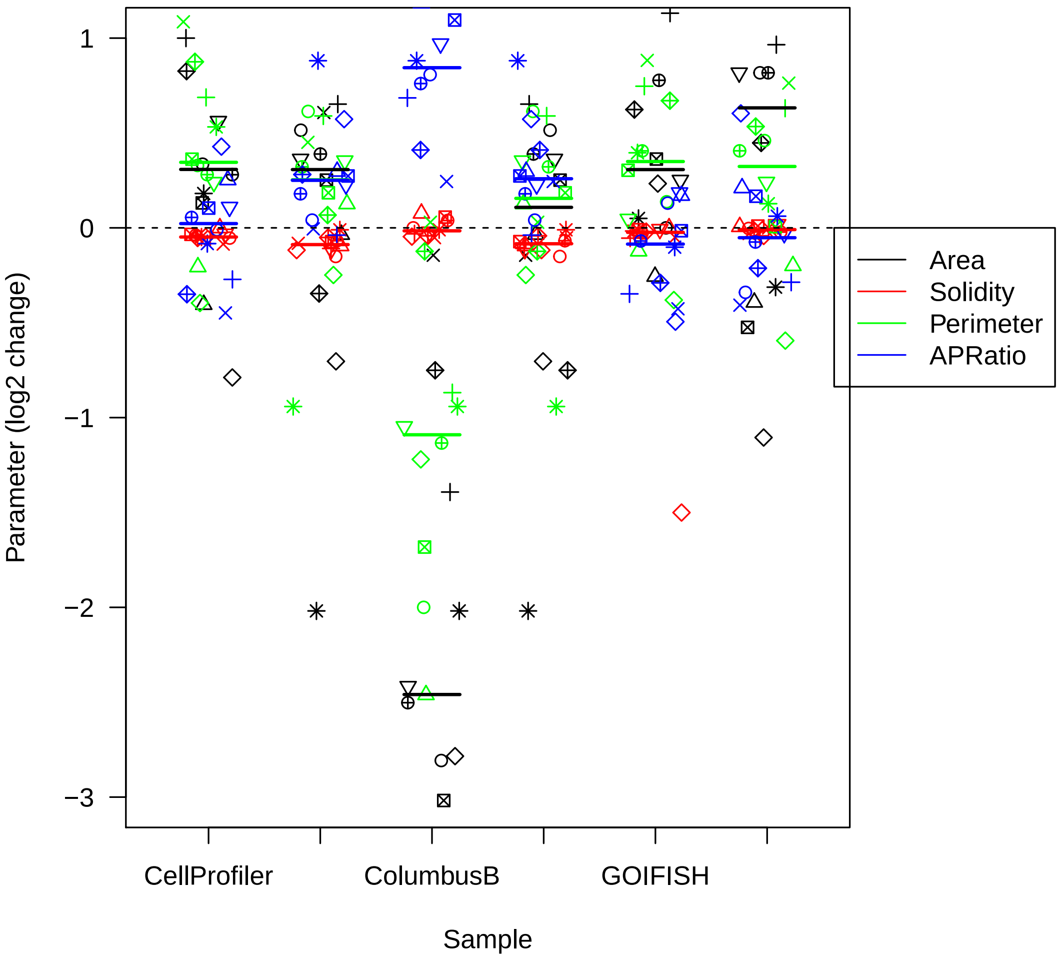

We see that most methods perform better as clusters, and thus use this as a measurement of performance

3.4 Figure2

```
# Create plots containing the best performing columbus method
par(mfrow = c(2, 2))
# DAPI
Precision.Recall.Plot(PR_DAPI[-c(2:3)])
# HER2
Precision.Recall.Plot(PR_HER2[c(1, 6:8)])
# cent17
```

```r
Precision.Recall.Plot(PR_Cent17[c(1, 4:6)])
# ERBB2
Precision.Recall.Plot(PR_ERBB2[c(1, 7, 9, 11)])
```

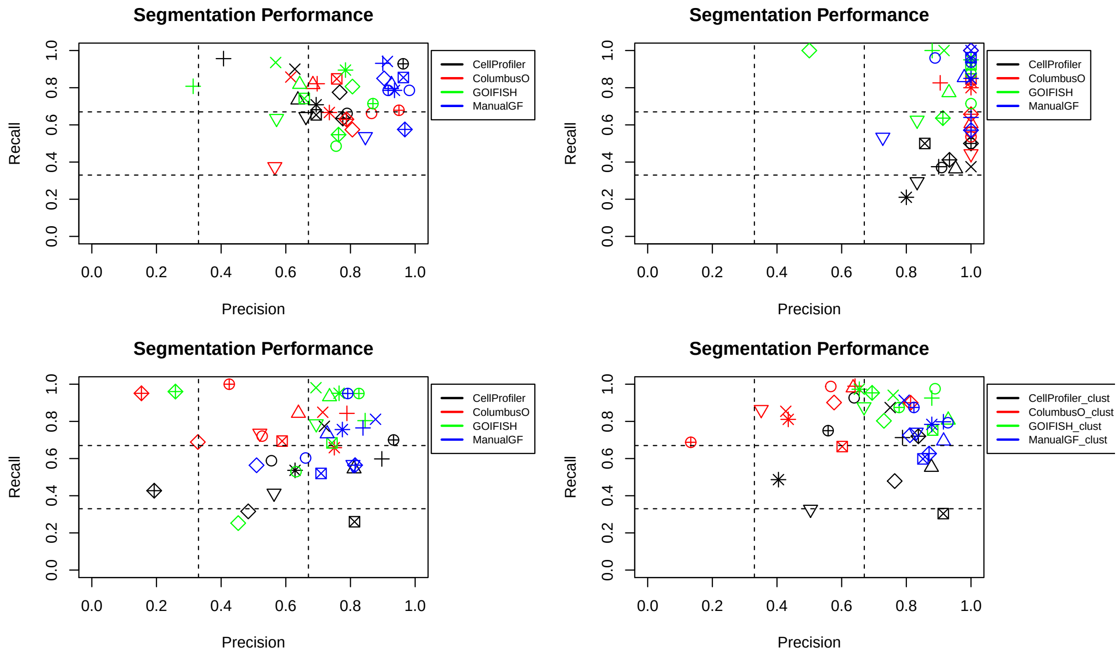


```r
# Calculate Mean F Scores
varnames=c("DAPI","HER2","cent17","ERBB2 clusters")
corrDAPI=sapply(c(1, 4:6), function(x) mean(PR_DAPI[[x]]$FScore))
corrHER2=sapply(c(1, 6:8), function(x) mean(PR_HER2[[x]]$FScore))
corrcent=sapply(c(1,4:6), function(x) mean(PR_Cent17[[x]]$FScore))
corrERBB2=sapply(c(1,7,9,11),function(x) mean(PR_ERBB2[[x]]$FScore) )
corr=cbind(corrDAPI, corrHER2, corrcent, corrERBB2)
colnames(corr)=varnames
rownames(corr)=c("CellProfiler", "Columbus", "GoFISH", "GoIFISH Adjusted")

polar.plot(corr, c(0, 90, 180, 270),labels = varnames,c(0, 90, 180, 270), main="Average

legend(0.4,0.4, c("CellProfiler", "Optimised Columbus", "GOIFISH Auto",
                  "GOIFISH Adjusted"), col=c(1:4), lwd=2, cex=0.8)
```

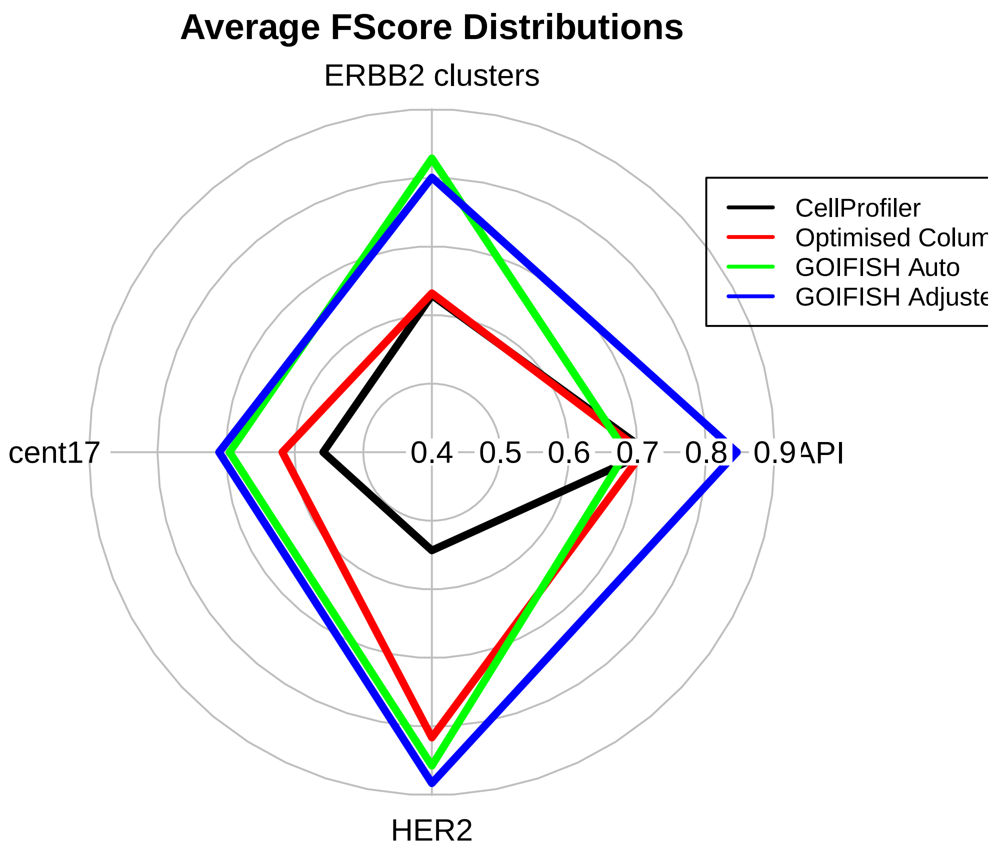


```
## Obtain the Mean FScores for each method:
corr

##                    DAPI   HER2 cent17 ERBB2 clusters
## CellProfiler      0.7156 0.5432 0.5584         0.6288
## Columbus          0.7078 0.8160 0.6180         0.6317
## GoFISH            0.6832 0.8570 0.6948         0.8282
## GoIFISH Adjusted 0.8446 0.8826 0.7102         0.8004
```

Next, we summarise the parameters for each sample, first we look at the area-perimeter ratio

```
# Calculate Property Scores:DAPI
HER2_AP=sapply(c(1, 6:8), function(x) PR_HER2[[x]]$APDiff)
HER2_solid=sapply(c(1, 6:8),function(x) PR_HER2[[x]]$SolidDiff)
DAPI_AP=sapply(c(1, 4:6), function(x) PR_DAPI[[x]]$APDiff)
DAPI_solid=sapply(c(1, 4:6),function(x) PR_DAPI[[x]]$SolidDiff)
```

```
corrA=cbind(DAPI_AP,HER2_AP)
corrA=log2(corrA)
corrB=cbind(DAPI_solid, HER2_solid)
corrB=log2(corrB)

tcols=c("gray", "pink", "lightgreen", "cornflowerblue")

###Plot the difference in AP-ratio
boxplot(corrA, col=rep(tcols, 2), xaxt="n",
        ylab="Difference in Area-Perimeter Ratio", outpch=NA)
tcols2=rep(c("black", "red", "darkgreen", "darkblue"), 2)
for (i in 1:8){
stripchart(corrA[ ,i], method = "jitter", jitter=0.3, pch = 18, col = tcols2[i],
           vertical=T, add = TRUE, at=i)}

abline(v=4.5)
abline(h=log2(1.25), lty=2)
abline(h=log2(0.75), lty=2)
```

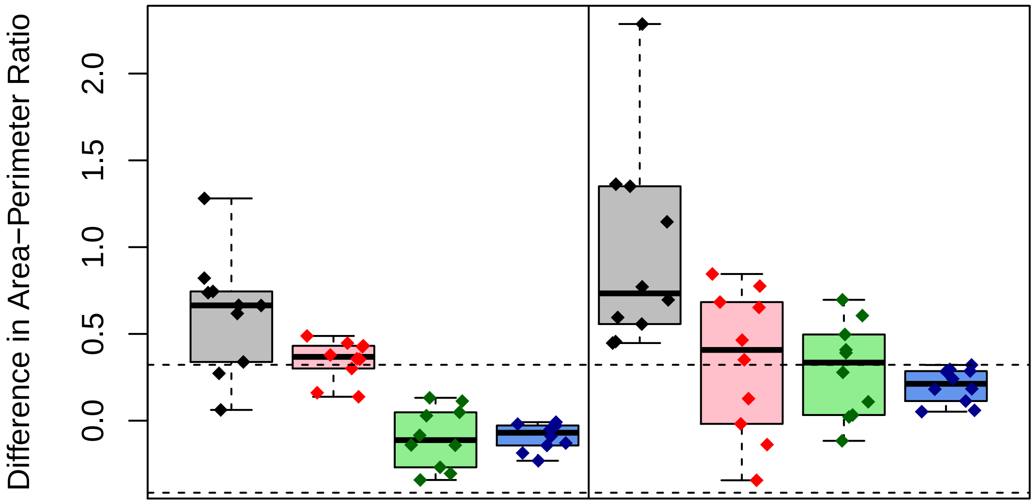

```
# Plot solidity differences
boxplot(corrB, col=rep(tcols, 2), xaxt="n", ylab="Differences in Solidity")
for (i in 1:8){
stripchart(corrB[ ,i], method = "jitter", jitter=0.3, pch = 18, col = tcols2[i], vertical
            add = TRUE, at=i)}
abline(v=4.5)
abline(h=log2(1.1), lty=2)
abline(h=log2(0.9), lty=2)
```

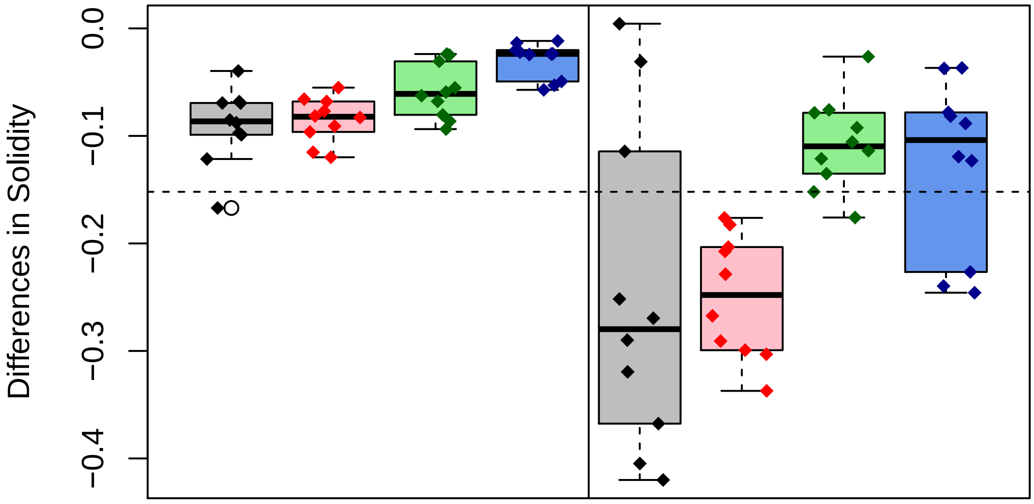

4 Biological comparison: Positivity and Intensity

Here, we check whether the membrane intensity is correlated with completeness or positivity.

Here is an example dataset:

```
load("data/Compiled_CellData.RData")
head(CP_Protein$"7588")

##   CellProfiler CellNo CP_Under DAPI_Area DAPI_Int Cyto_Area HER2cytoInt
## 1            6      2        0      6081   167.10     37445       609.0
## 2           14      3        0      6326   164.04     17313       216.0
## 3           12      4        0      5850   145.85     21233       317.9
## 4           10      7        0      4832   132.64     20605       272.5
## 5           17      8        0      3311    80.33     25840       429.0
## 6           26     10        0      4329   108.00     20754       380.7
##   ER.score HER2.prot.score HER2.membrane.score   ER_Int HER2memInt HER2_SD
## 1      mod             +ve            complete 0.006588    0.05683  0.2315
## 2      mod             +ve              broken 0.005495    0.05736  0.2325
## 3      mod             +ve            complete 0.006753    0.06141  0.2401
## 4      mod             +ve              broken 0.006641    0.05698  0.2318
## 5      mod             +ve              broken 0.005912    0.06207  0.2413
## 6      mod             +ve              broken 0.006362    0.03941  0.1946
```

Here, we see there are three pathologist scores: one for ER status, HER2 status and HER2 membrane continuity. Additionally, there are parameters obtained from an automated platform (ie. Columbus): These include mean DAPI intensity, ER intensity, Cytoplasmic and Membrane intensities for each method used.

4.1 ER Intensity Comparisons

4.1.1 Searching for trend in individual samples

Here, we plot the intensities correlating to all pathologist labelled cells for all ten images. Ideally, we would like to see: 1. That intensity increases with positivity. 2. Cells of the same class will have similar intensities across all samples. Eg. All negative cells will be close to 0, and all positive cells close to 100.

```
# Plot the outputs: ER
OutputCP=IntensityComparison(CP_Protein, "ER.score", "ER_Int",F, "CellProfiler")
```

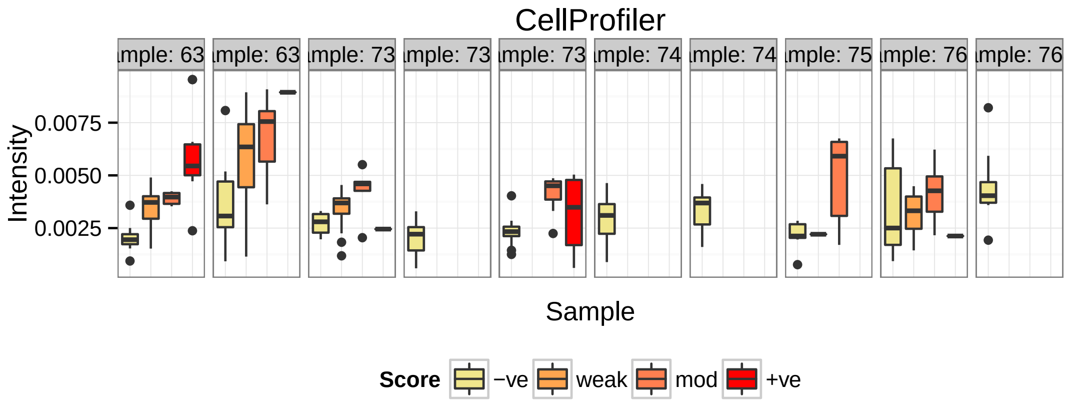


```
OutputCol=IntensityComparison(Columbus_Protein, "ER.score", "ER_Int", F, "Columbus")
```

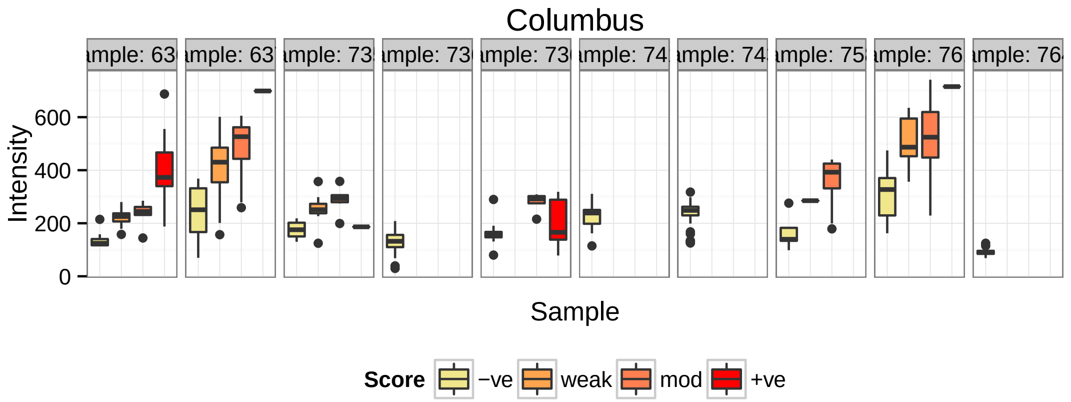


```
Output=IntensityComparison(GF_Protein, "ER.score", "ER_Int",F, "GOIFISH")
```

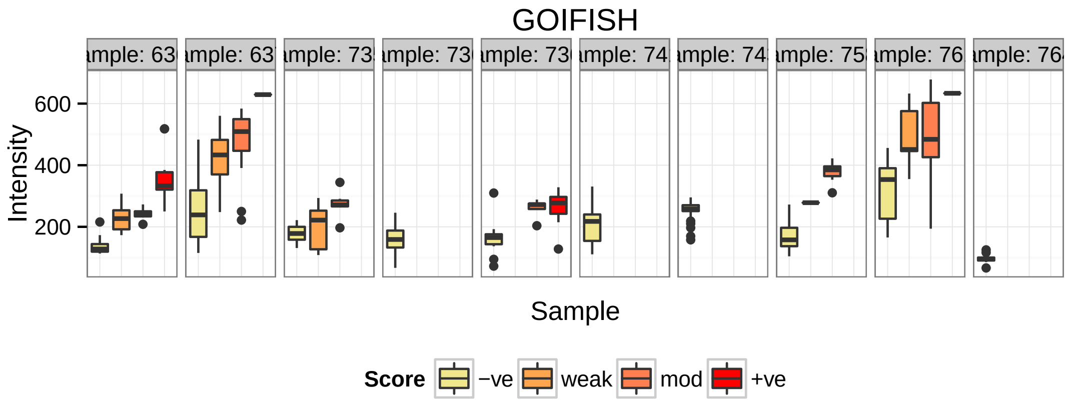


```
Output=IntensityComparison(GF_Protein, "ER.score", "ER_backAdj",F,
                           "GOIFISH Background Adjusted")
```

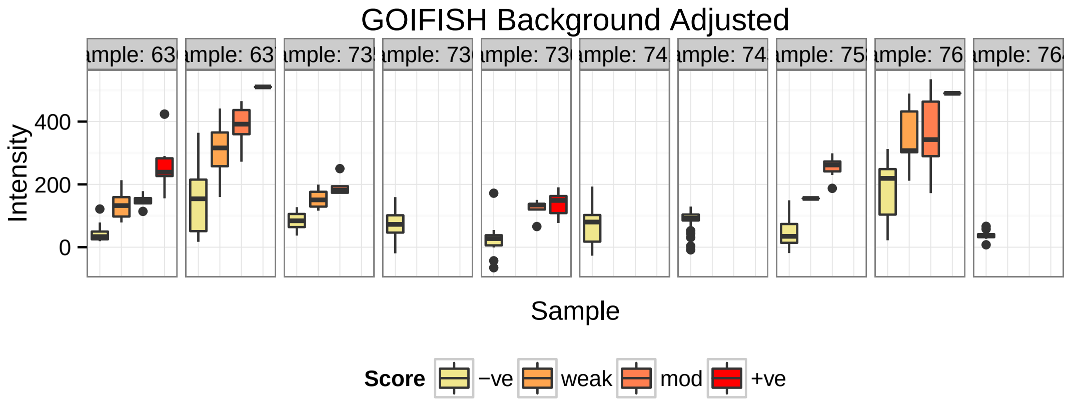


```
OutputGF=IntensityComparison(GF_Protein, "ER.score", "ERNucInt",F,
                             "GOIFISH Nuclear Specific Adjustment")
```

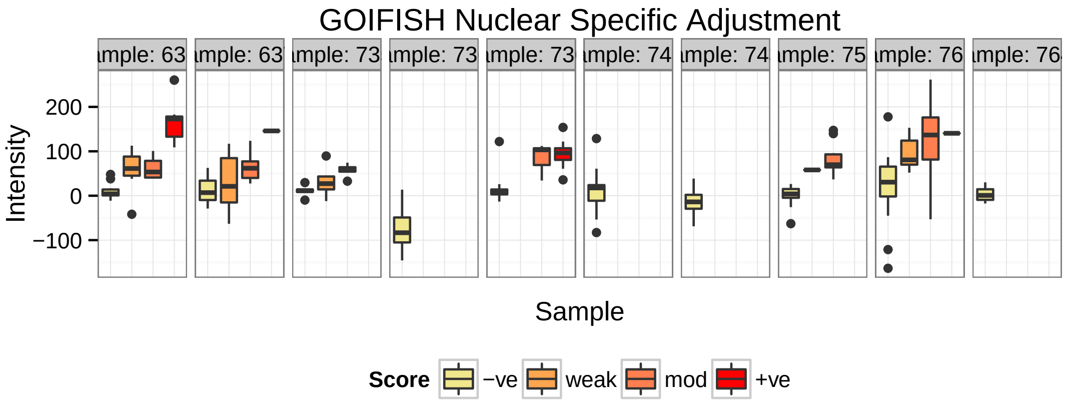


```
OutputMGF=IntensityComparison(ManGF_Protein, "ER.score", "ERNucInt", F,
                              "Manual GOIFISH Nuclear Specific Adjustment")
```

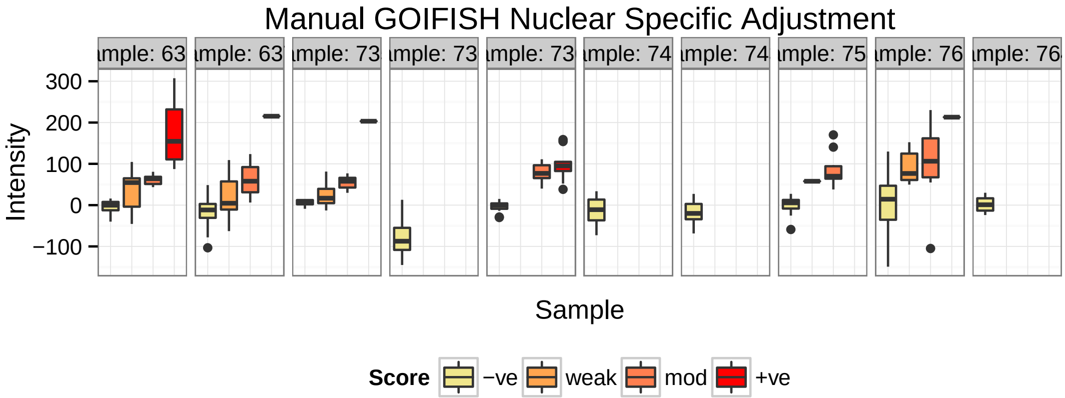

The first plot shows the distribution of intensities with respect to class. Here, we see the baseline intensities (of "negative cells") varies across the samples. The exception to this is the GoIFISH Nuclear Specific Adjustment which appears to have all negative cells centred around 0 (with the exception of 7360 - in this case, the background is higher than the cell intensity and we can regard its intensity to be 0).

4.1.2 Anova to test for direct comparability

To determine whether any of these samples are directly comparable, we implement an anova and Tukey Honest Significant Differences test. The direct output of this shown in the top right plot: All pairwise tests using the baseline intensities are performed. Those which are centred around "0" have no difference.

To look in greater detail of which samples can be directly compared, heatmaps of the -log10Pvalue and the differences in means. The diagonal of both plots show the

significance value (-log10(0.05)) and 0 difference respectively. Thus, in the pvalue plot, all samples which are darker than this shade of green will have a significant pvalue and hence, a difference in the baseline intensities. All samples which are light green can be directly compared.

In the difference plot, 0 difference is showed by a light blue background. We see that sample 7614 has a lower baseline intensity value (indicated by more red colours). Samples 6370, 7588 and 7619 have bluer colours, indicating these have a higher baseline intensity.

We can look at the Output of `IntensityComparison` to determine how many pairwise comparisons can be performed:

```
# Find number of pairwise samples which can be performed:
Nsamp = length(which(OutputCP$TukeyHSD[, 4] > 0.05))
Nsamp[2] = length(which(OutputGF$TukeyHSD[, 4] > 0.05))
Nsamp[3] = length(which(OutputCol$TukeyHSD[, 4] > 0.05))
Nsamp[4] = length(which(OutputMGF$TukeyHSD[, 4] > 0.05))
Nsamp

## [1] 32 35 20 36

# Calculate Total number of samples:
nrow(OutputCP$TukeyHSD)

## [1] 45

nrow(OutputGF$TukeyHSD)

## [1] 45

nrow(OutputCol$TukeyHSD)

## [1] 45

nrow(OutputMGF$TukeyHSD)

## [1] 45
```

Here, we see that 40% of direct comparisons will be valid in the Columbus case, and almost 90% in CellProfiler and GoIFISH. Note that `GoIFISH` sample 7360 has a negative has negative ER intensities due to background subtraction - in practice, this would be assigned 0.

We can also check whether the upper limit is directly comparable:

```
Nsamp = length(which(OutputCP$TukeyUpper[, 4] > 0.05))
Nsamp[2] = length(which(OutputGF$TukeyUpper[, 4] > 0.05))
Nsamp[3] = length(which(OutputCol$TukeyUpper[, 4] > 0.05))
Nsamp[4] = length(which(OutputMGF$TukeyUpper[, 4] > 0.05))
Nsamp

## [1] 39 27 26 29

# number of comparisons
nrow(OutputCP$TukeyUpper)

## [1] 45

nrow(OutputGF$TukeyUpper)

## [1] 45

nrow(OutputCol$TukeyUpper)

## [1] 45

nrow(OutputMGF$TukeyUpper)

## [1] 45
```

One of the other outputs from MegaBoxPlot is the Jonchkeere test for trend: In all applicable samples, we see if there is a trend of increasing intensity with increase in ER positive classification

```
par(mfrow = c(2, 2))
CorTestImage(rbind(OutputCP$Jonchkeere[, 2], OutputCP$Jonchkeere[, 1]/1000))
title(main = "CellProfiler")
CorTestImage(rbind(OutputCol$Jonchkeere[, 2], OutputCol$Jonchkeere[, 1]/1000))
title(main = "Columbus")
CorTestImage(rbind(OutputGF$Jonchkeere[, 2], OutputGF$Jonchkeere[, 1]/1000))
title(main = "GoIFISH")
```

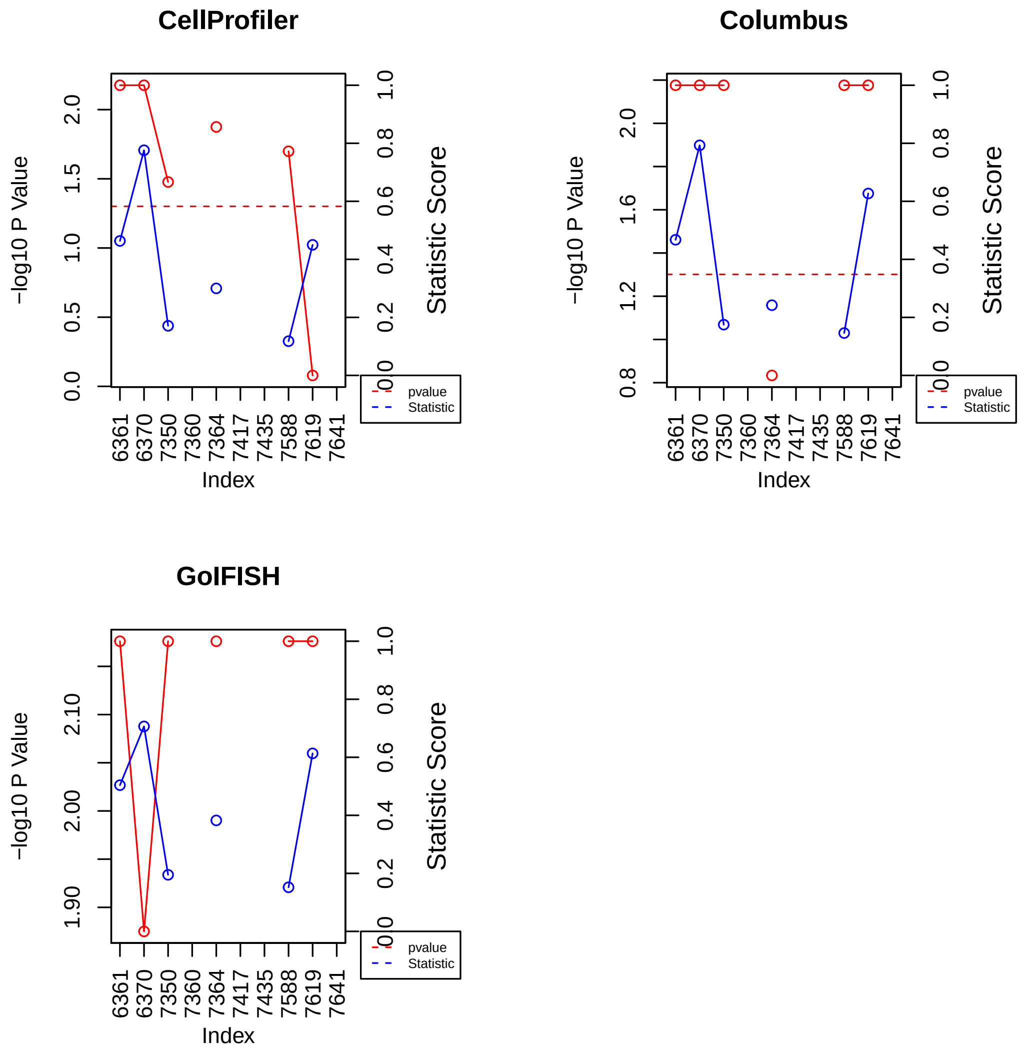

Here, we see 6 samples are tested for Jonchkeere's trend. In red is the -log10pValue, with the threshold of 0.05 shown as a dotted line. The test statistic (divided by 1000 to allow a similar scale) is shown in blue. Here, we see evidence of an increasing trend between measured intensity and pathologist categorisation. Note that there is evidence for 'increasing trend' in all samples except for the CellProfiler 7350 sample, and the Columbus 7364 smaple.

4.1.3 Combining All Samples

We can try to group these samples together, and see if we see a difference between all the classes:

What if we combined all the samples and see where they lie in relation to one another?

```r
par(mfrow=c(3, 2))
CP_ER=unlist(lapply(1:10, function(x) as.character(CP_Protein[[x]]$ER.score)))
CP_ER=cbind(CP_ER, unlist(lapply(1:10, function(x) CP_Protein[[x]]$ER_Int)))

Columbus_ER=unlist(lapply(1:10, function(x) as.character(Columbus_Protein[[x]]$ER.score))[illegible]
Columbus_ER=cbind(Columbus_ER, unlist(lapply(1:10, function(x)
  Columbus_Protein[[x]]$ER_Int)))

GF_ER=unlist(lapply(1:10, function(x) as.character(GF_Protein[[x]]$ER.score)))
GF_ER=cbind(GF_ER, as.numeric(unlist(lapply(1:10, function(x)
  GF_Protein[[x]]$ER_Int))))
GF_ER=cbind(GF_ER, as.numeric(unlist(lapply(1:10, function(x)
  GF_Protein[[x]]$ER_backAdj))))
GF_ER=cbind(GF_ER, as.numeric(unlist(lapply(1:10, function(x)
  GF_Protein[[x]]$ERNucInt))))

MGF=unlist(lapply(1:10, function(x) as.character(ManGF_Protein[[x]]$ER.score)))
MGF=cbind(MGF, as.numeric(unlist(lapply(1:10, function(x) ManGF_Protein[[x]]$ERNucInt)))[illegible]

## Plot the Boxplots of intensities
## Run function for specialised boxplots
BoxPlotCompare(CP_ER[ ,2],CP_ER[ ,1], "Cellprofiler")
BoxPlotCompare(Columbus_ER[ ,2],Columbus_ER[ ,1], "Columbus")
BoxPlotCompare(GF_ER[ ,2],GF_ER[ ,1], "GOIFISH")
BoxPlotCompare(GF_ER[ ,3],GF_ER[ ,1], "GOIFISH background Adjusted")
BoxPlotCompare(GF_ER[ ,4],GF_ER[ ,1], "GOIFISH nuclear Adjusted")
BoxPlotCompare(MGF[ ,2],MGF[ ,1], "Manual GOIFISH nuclear Adjusted")
```

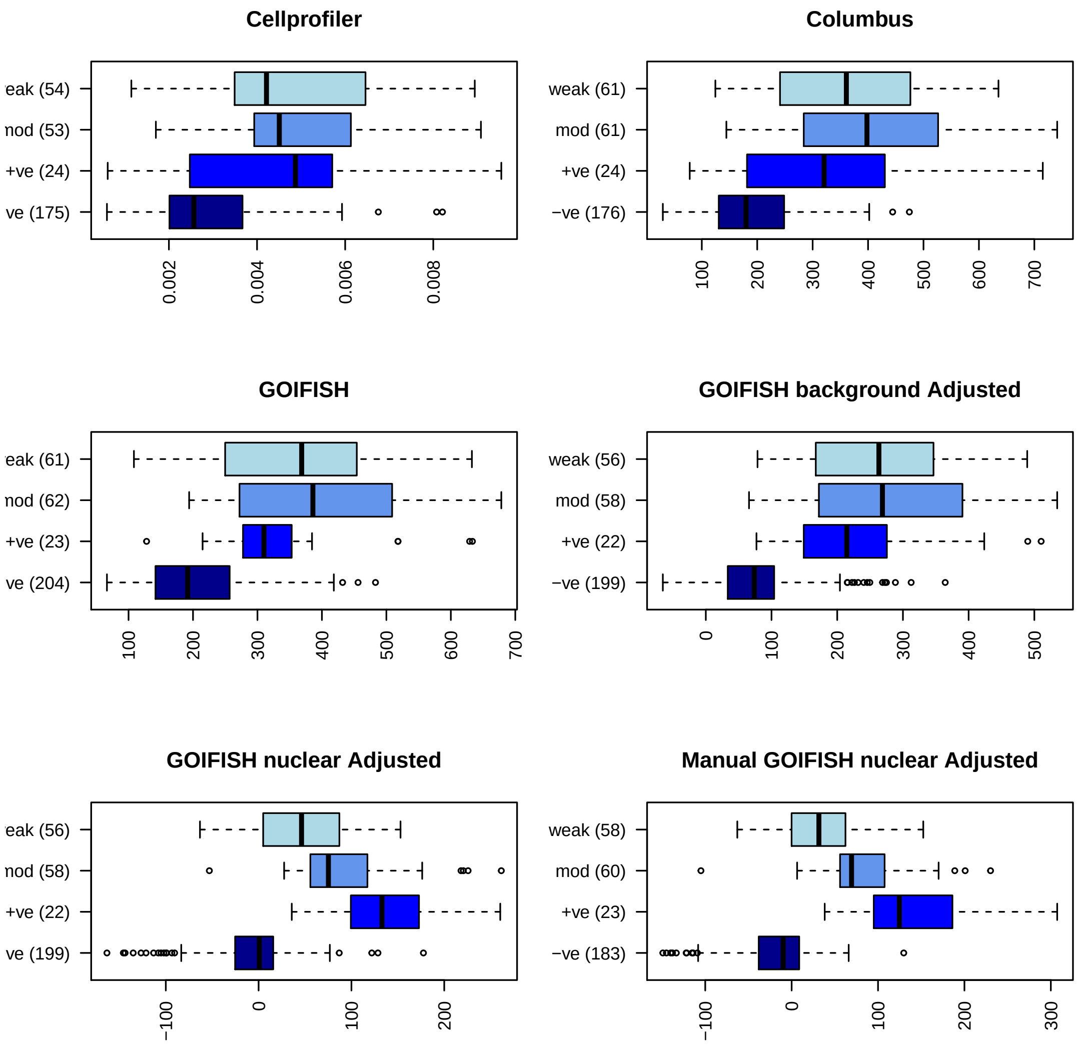

The nuclear adjusted GoIFISH samples show a nice difference between negative, weak, moderate and positive. We can check for all samples whether there is some type of difference between the classes and how strong it is, by running a series of t-tests:

```
par(mfrow = c(3, 2))
Matrix.Ttest(CP_ER)
title(main = "CellProfiler")
Matrix.Ttest(Columbus_ER)
title(main = "Columbus")
Matrix.Ttest(GF_ER)
title(main = "GF_raw")
Matrix.Ttest(GF_ER[, c(1, 3)])
title(main = "GF_adj")
```

```
Matrix.Ttest(GF_ER[, c(1, 4)])
title(main = "GF_raw")
Matrix.Ttest(MGF)
title(main = "Manual GoIFISH")
```

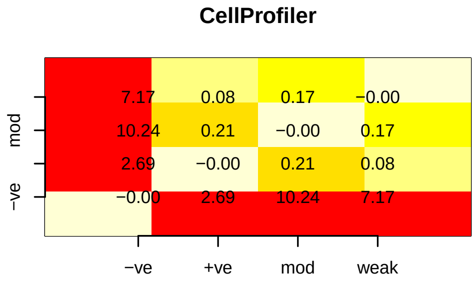

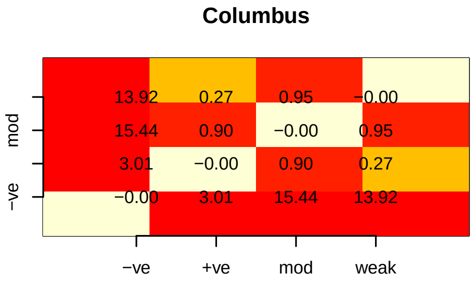

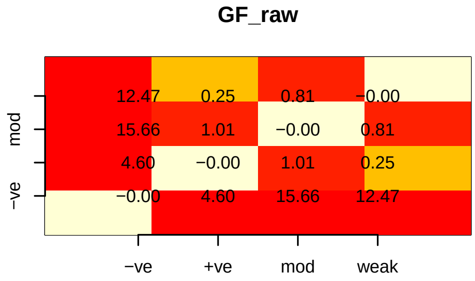

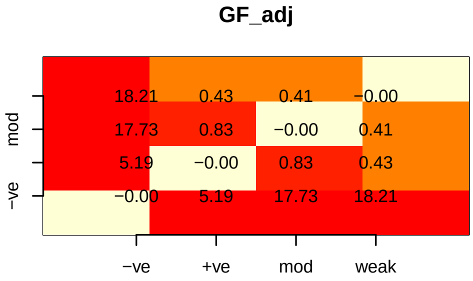

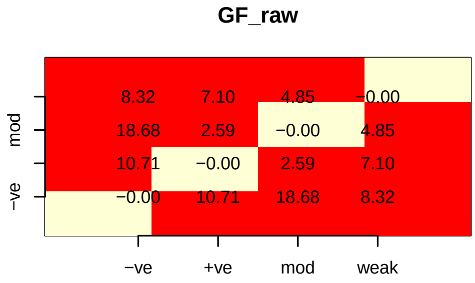

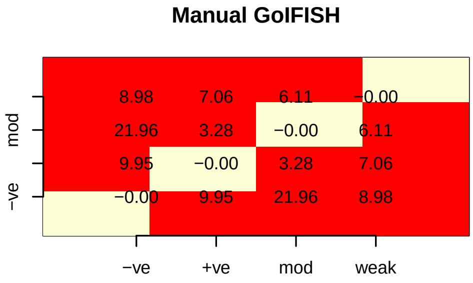

The -1og10pvalues are indicated on each comparison. A significant value is expected to be higher than 0.05 or 1.3 in -log10 scale. We see that the comparisons between positive, moderate and weak become significant after performing cell specific normalisation. However, when we simply compare ”negative samples” to ”the rest”, all samples showed significant results.

We test to see if there is an increasing trend amongst all samples by running a Jonckeree -Terpsa test for trend: we expect to see an increase in intensity as we progress from absent to positive.

```r
## JT test:
JT_HER2=rep(NA, 6)
# CellProfiler
JT_HER2[1]=jonckheere.test(as.numeric(CP_ER[ ,2]), factor(CP_ER[,1],
                     levels=c("-ve", "weak","mod","+ve"), ordered=T),
                      nperm=1000)$p.value
# Columbus
JT_HER2[2]=jonckheere.test(as.numeric(Columbus_ER[ ,2]), factor(Columbus_ER[,1],
                    levels=c("-ve", "weak","mod","+ve"), ordered=T),
                      nperm=1000)$p.value
# GoIFISH
JT_HER2[3]=jonckheere.test(as.numeric(GF_ER[ ,2]),
                      factor(GF_ER[,1],
                      levels=c("-ve", "weak","mod","+ve"), ordered=T),
                          nperm=1000)$p.value
JT_HER2[4]=jonckheere.test(as.numeric(GF_ER[ ,3]),
                      factor(GF_ER[,1],
                      levels=c("-ve", "weak","mod","+ve"), ordered=T),
                      nperm=1000)$p.value
JT_HER2[5]=jonckheere.test(as.numeric(GF_ER[ ,4]),
                      factor(GF_ER[,1],
                      levels=c("-ve", "weak","mod","+ve"), ordered=T),
                      nperm=1000)$p.value
JT_HER2[6]=jonckheere.test(as.numeric(MGF[ ,2]),
                      factor(MGF[,1],
                      levels=c("-ve", "weak","mod","+ve"), ordered=T),
                      nperm=1000)$p.value
names(JT_HER2)=c("CP", "Col", "GF_raw", "GF_Adj", "GF_nuc", "ManGF")
t(JT_HER2)

##         CP   Col GF_raw GF_Adj GF_nuc ManGF
## [1,] 0.002 0.002  0.002  0.002  0.002 0.002
```

We see that the increasing trend is significant in all of the above samples.

4.2 HER2 protein expression

We repeat the analysis for HER2 protein in a similar manner as described above. Here, we are interested in two types of analysis: Whether membrane or cytoplasmic intensities correlate with the given scores, and whether continuity in the membrane can be detected:

4.2.1 HER2 positivity and negativity: Is there a difference between these samples?

Next, we want to check whether the positivity or negativitiy of pathologist scoring is related to the intensity of the sample.

```
par(mfrow = c(3, 1))
memOutCP = IntensityComparison(CP_Protein, "HER2.prot.score", "HER2memInt",
    F, "CellProfiler membrane")
```

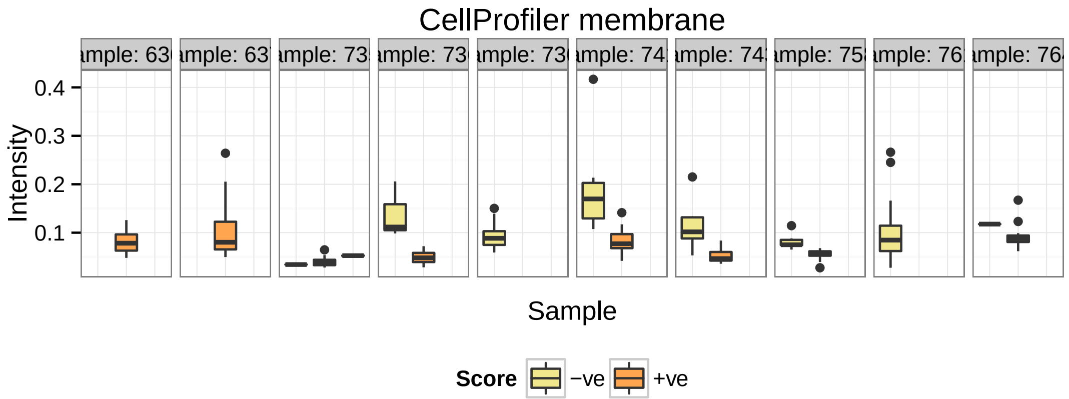


```
memOutCol = IntensityComparison(Columbus_Protein, "HER2.prot.score", "HER2memInt",
    F, "Columbus membrane")
```

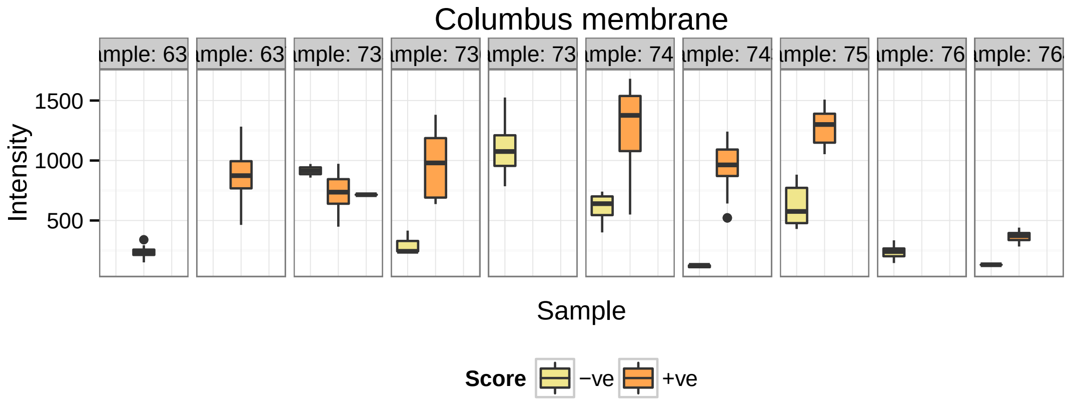


```
memOutGF = IntensityComparison(GF_Protein, "HER2.prot.score", "HER2memInt",
    F, "GF Raw membrane")
```

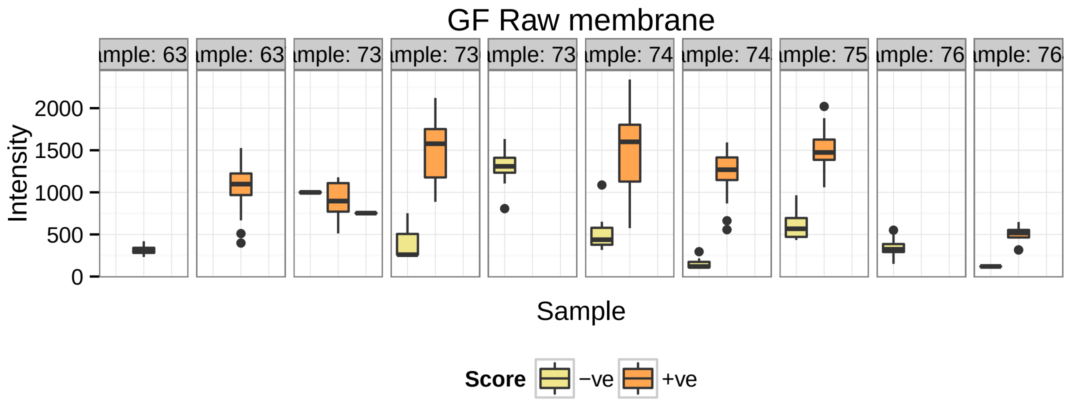


```
memOutGFback = IntensityComparison(GF_Protein, "HER2.prot.score", "HER2backAdj",
    F, "GF Adjusted Membrane")
```

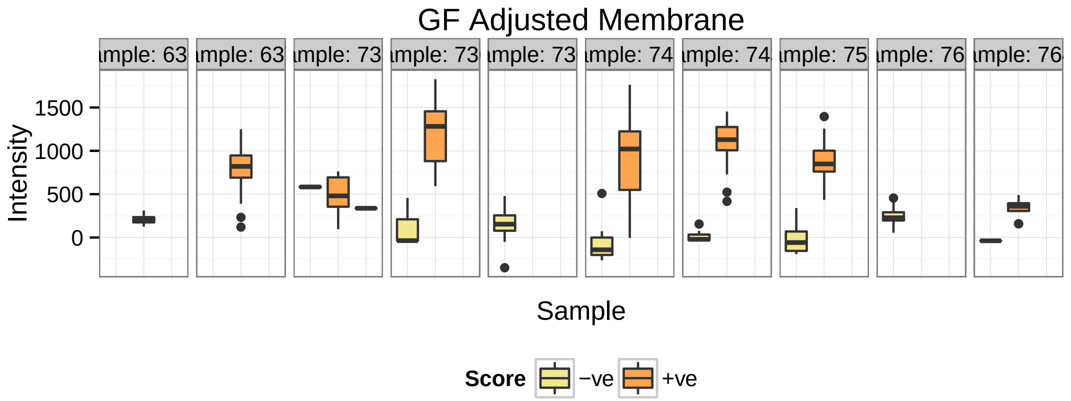


```
Man0 = IntensityComparison(ManGF_Protein, "HER2.prot.score", "HER2memInt", F,
    "GF Manual Raw membrane")
```

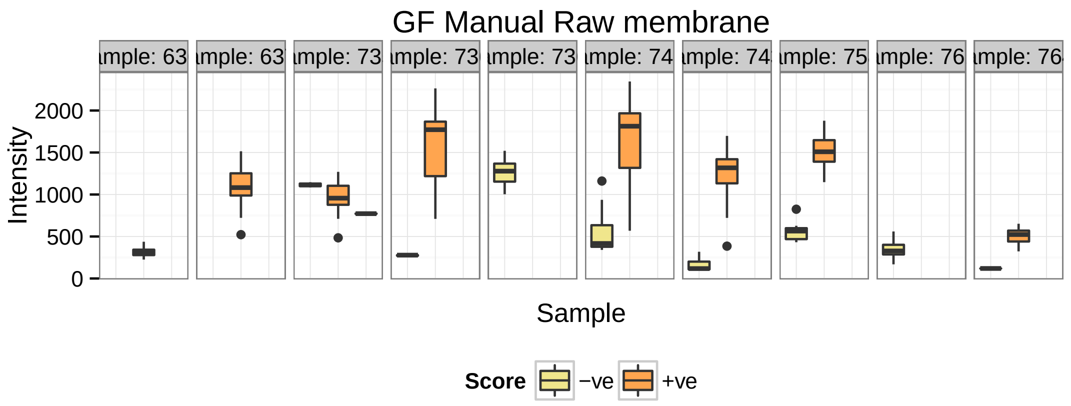


```
Man2 = IntensityComparison(ManGF_Protein, "HER2.prot.score", "HER2backAdj",
    F, "GF Manual Adjusted Membrane")
```

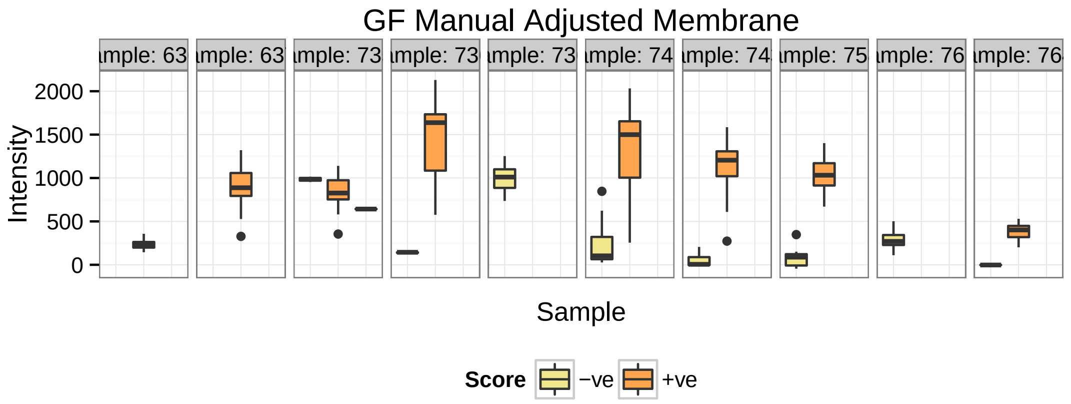


```
# Find number of pairwise samples which can be performed:
Nsamp = length(which(memOutCP$TukeyHSD[, 4] > 0.05))
Nsamp[2] = length(which(memOutGFback$TukeyHSD[, 4] > 0.05))
Nsamp[3] = length(which(memOutCol$TukeyHSD[, 4] > 0.05))
Nsamp[4] = length(which(Man2$TukeyHSD[, 4] > 0.05))
Nsamp

## [1] 25 17 10 14

# Calculate Total number of samples:
nrow(memOutCP$TukeyHSD)

## [1] 28
```

```
# Check for the upper limit
Nsamp = length(which(memOutCP$TukeyUpper[, 4] > 0.05))
Nsamp[2] = length(which(memOutGFback$TukeyUpper[, 4] > 0.05))
Nsamp[3] = length(which(memOutCol$TukeyUpper[, 4] > 0.05))
Nsamp[4] = length(which(Man2$TukeyUpper[, 4] > 0.05))
Nsamp

## [1] 32 18 15 19
```

We can apply a Jonchkeere test for trend amongst all samples

```
# Gather the JT HER2 intensity measurements
par(mfrow = c(2, 2))
CorTestImage(rbind(memOutCP$Jonchkeere[, 2], memOutCP$Jonchkeere[, 1]/1000))
title(main = "CP mem")
CorTestImage(rbind(memOutCol$Jonchkeere[, 2], memOutCol$Jonchkeere[, 1]/1000))
title(main = "Col mem")
CorTestImage(rbind(memOutGF$Jonchkeere[, 2], memOutGF$Jonchkeere[, 1]/1000))
title(main = "GF mem")
CorTestImage(rbind(memOutGFback$Jonchkeere[, 2], memOutGFback$Jonchkeere[, 1]/1000))
title(main = "GF adjust mem")
```

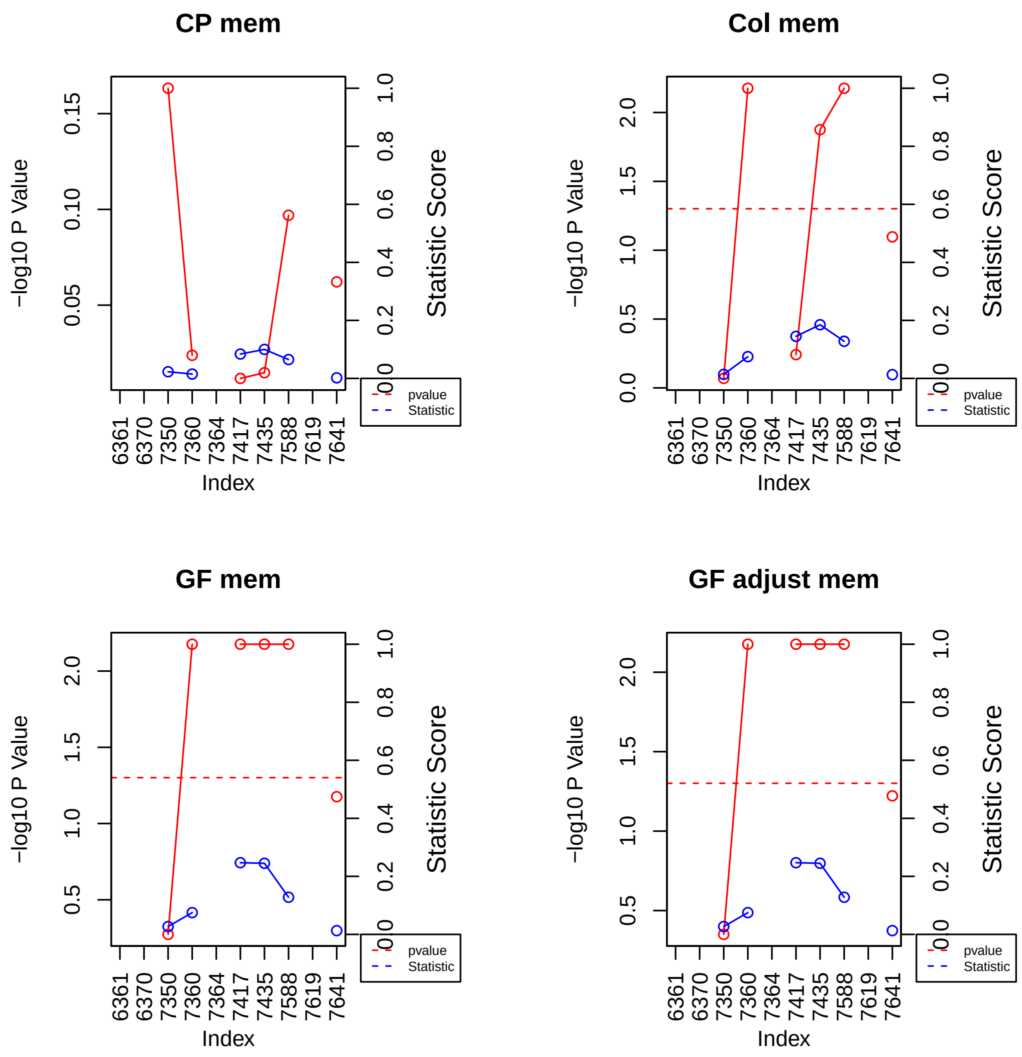

In the GoIFISH samples, we see that about half the scorings are significant. Sample 7364 may have low scroing as most cells are negative.

4.2.2 Grouping all samples and comparing positivity vs negativitiy

```
par(mfrow=c(3, 2))
CP_HER2=unlist(lapply(1:10, function(x) as.character(CP_Protein[[x]]$HER2.prot.score)))
CP_HER2=cbind(CP_HER2, unlist(lapply(1:10, function(x)
  as.character(CP_Protein[[x]]$HER2memInt))))

Columbus_HER2=unlist(lapply(1:10, function(x)
  as.character(Columbus_Protein[[x]]$HER2.prot.score)))
```

```
Columbus_HER2=cbind(Columbus_HER2, unlist(lapply(1:10, function(x)
  as.character(Columbus_Protein[[x]]$HER2memInt))))

GF_HER2=unlist(lapply(1:10, function(x) as.character(GF_Protein[[x]]$HER2.prot.score)))
GF_HER2=cbind(GF_HER2, unlist(lapply(1:10, function(x)
  as.character(GF_Protein[[x]]$HER2memInt))))
GF_HER2=cbind(GF_HER2, unlist(lapply(1:10, function(x)
  as.character(GF_Protein[[x]]$HER2backAdj))))

MGF_HER2=unlist(lapply(1:10, function(x)
  as.character(ManGF_Protein[[x]]$HER2.prot.score)))
MGF_HER2=cbind(MGF_HER2, unlist(lapply(1:10, function(x)
  as.character(ManGF_Protein[[x]]$HER2backAdj))))

## Plot the Boxplots of intensities
BoxPlotCompare(CP_HER2[ ,2], CP_HER2[ ,1],"CellProfiler")
BoxPlotCompare(Columbus_HER2[ ,2], Columbus_HER2[ ,1],"Columbus")
BoxPlotCompare(GF_HER2[ ,2],GF_HER2[ ,1],"GOIFISH")
BoxPlotCompare(GF_HER2[ ,3],GF_HER2[ ,1],"GOIFISH background adj")
BoxPlotCompare(MGF_HER2[ ,2],MGF_HER2[ ,1],"GOIFISH manual background adj")
# CellProfiler
 t.test(as.numeric(CP_HER2[, 2])~factor(CP_HER2[ ,1]))

##
##  Welch Two Sample t-test
##
## data:  as.numeric(CP_HER2[, 2]) by factor(CP_HER2[, 1])
## t = 4.503, df = 97.09, p-value = 1.863e-05
## alternative hypothesis: true difference in means is not equal to 0
## 95 percent confidence interval:
##  0.01843 0.04748
## sample estimates:
## mean in group -ve mean in group +ve
##           0.10647           0.07351

# Columbus
 t.test(as.numeric(Columbus_HER2[, 2])~factor(Columbus_HER2[ ,1]))

##
##  Welch Two Sample t-test
##
## data:  as.numeric(Columbus_HER2[, 2]) by factor(Columbus_HER2[, 1])
## t = -4.648, df = 175.9, p-value = 6.553e-06
## alternative hypothesis: true difference in means is not equal to 0
```

```r
## 95 percent confidence interval:
##  -332.7 -134.4
## sample estimates:
## mean in group -ve mean in group +ve
##             554.6             788.1

# GoIFISH
 t.test(as.numeric(GF_HER2[, 2])~factor(GF_HER2[ ,1]))

##
##  Welch Two Sample t-test
##
## data:  as.numeric(GF_HER2[, 2]) by factor(GF_HER2[, 1])
## t = -6.497, df = 247.9, p-value = 4.465e-10
## alternative hypothesis: true difference in means is not equal to 0
## 95 percent confidence interval:
##  -465.1 -248.7
## sample estimates:
## mean in group -ve mean in group +ve
##             680.9            1037.8

# GoIFISH background adjusted
t.test(as.numeric(GF_HER2[, 3])~factor(GF_HER2[ ,1]))

##
##  Welch Two Sample t-test
##
## data:  as.numeric(GF_HER2[, 3]) by factor(GF_HER2[, 1])
## t = -17.97, df = 319.9, p-value < 2.2e-16
## alternative hypothesis: true difference in means is not equal to 0
## 95 percent confidence interval:
##  -666.8 -535.2
## sample estimates:
## mean in group -ve mean in group +ve
##             151.3             752.4

# Manual GoIFISH cell adjusted
t.test(as.numeric(MGF_HER2[, 2])~factor(MGF_HER2[ ,1]))

##
##  Welch Two Sample t-test
##
## data:  as.numeric(MGF_HER2[, 2]) by factor(MGF_HER2[, 1])
## t = -7.761, df = 232.6, p-value = 2.654e-13
```

```
## alternative hypothesis: true difference in means is not equal to 0
## 95 percent confidence interval:
##  -497.3 -295.9
## sample estimates:
## mean in group -ve mean in group +ve
##             505.2             901.8
```

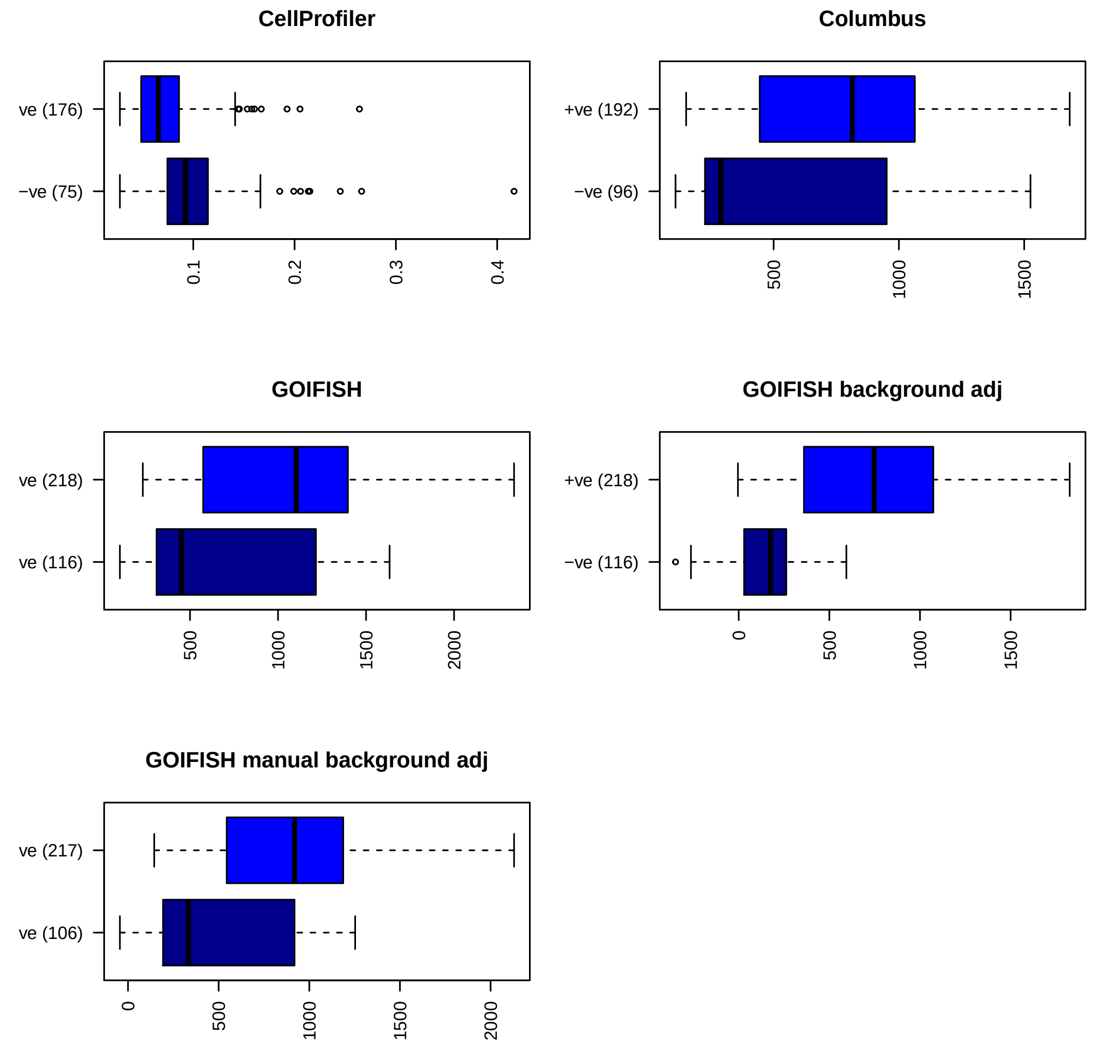

It appears that the comparison between positive and negative samples is significant using all methods.

4.2.3 HER2 membrane completeness

A second variable to consider when scoring samples clinically is the continuity of the membrane in a HER2 sample.

```
OutputCP = IntensityComparison(CP_Protein, "HER2.membrane.score", "HER2memInt",
    F, "CellProfiler")
```

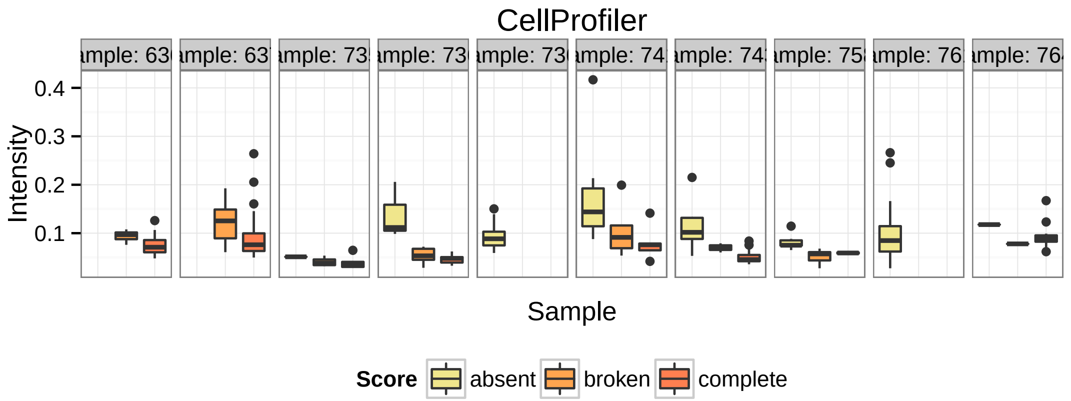


```
OutputCol = IntensityComparison(Columbus_Protein, "HER2.membrane.score", "HER2memInt",
    F, "Columbus")
```

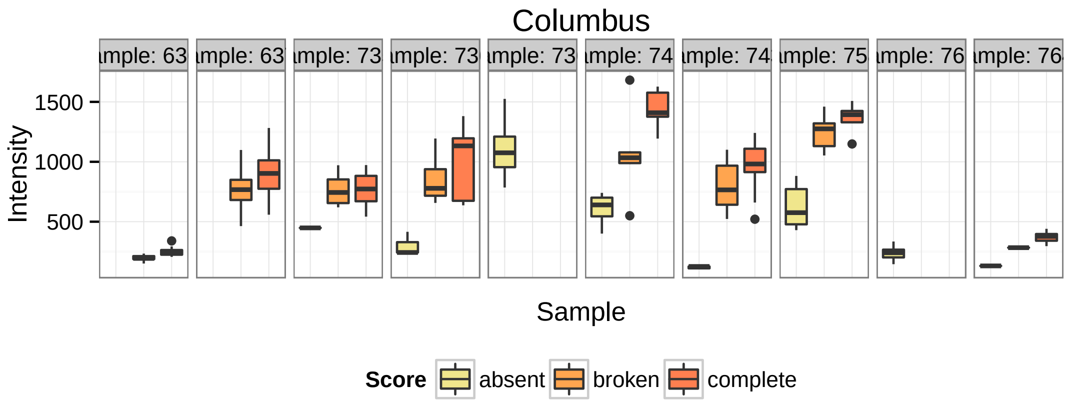


```
OutputGF = IntensityComparison(GF_Protein, "HER2.membrane.score", "HER2memInt",
    F, "GOIFISH")
```

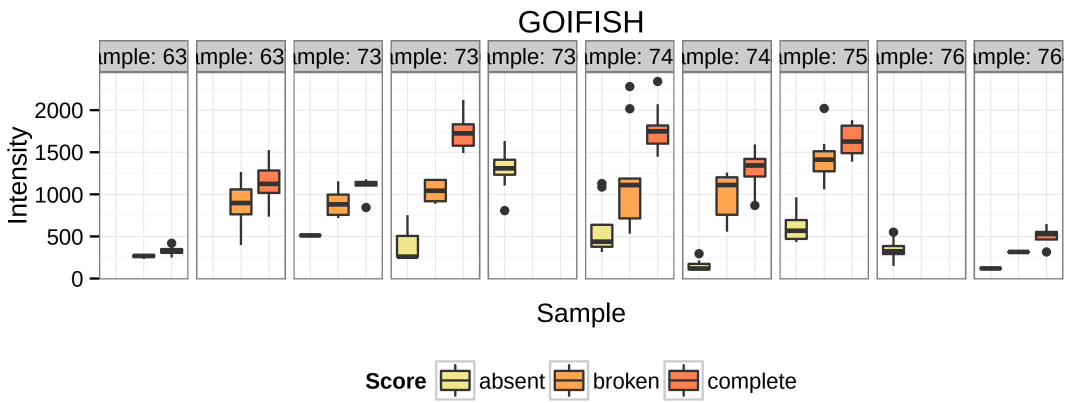


```
OutputGFAdj = IntensityComparison(GF_Protein, "HER2.membrane.score", "HER2backAdj",
    F, "GOIFISH Background Adjusted")
```

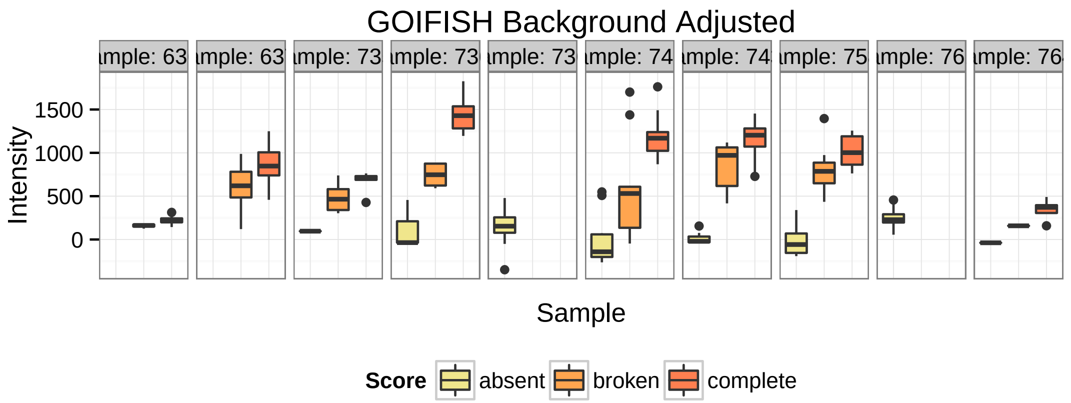


```
OutputMGF = IntensityComparison(ManGF_Protein, "HER2.membrane.score", "HER2backAdj",
    F, "GOIFISH Manual Background Adjusted")
```

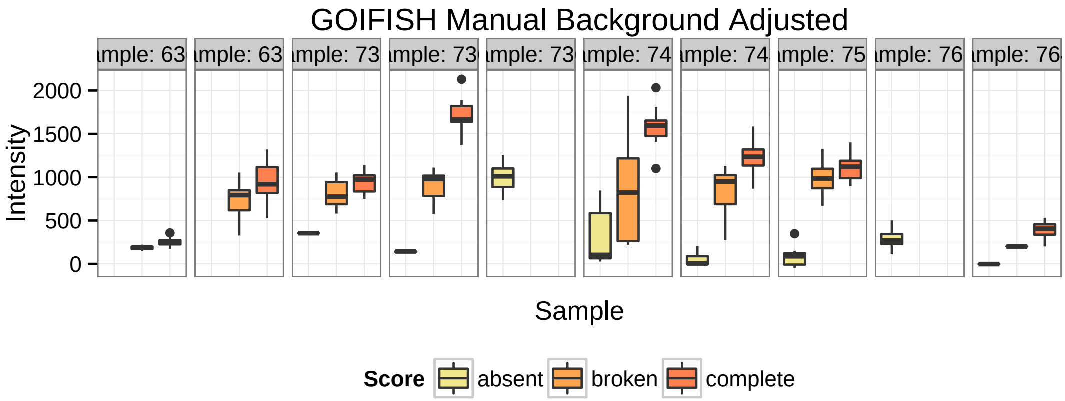

It appears that the intensity is variable even after adjustment. We can check whether the intensity actually does increase in each sample.

```
par(mfrow = c(3, 2))
CorTestImage(rbind(OutputCP$Jonchkeere[, 2], OutputCP$Jonchkeere[, 1]/1000))
title(main = "CellProfiler")
CorTestImage(rbind(OutputCol$Jonchkeere[, 2], OutputCol$Jonchkeere[, 1]/1000))
title(main = "Columbus")
CorTestImage(rbind(OutputGF$Jonchkeere[, 2], OutputGF$Jonchkeere[, 1]/1000))
title(main = "GoIFISH")
CorTestImage(rbind(OutputGFAdj$Jonchkeere[, 2], OutputGFAdj$Jonchkeere[, 1]/1000))
title(main = "GoIFISH Adjusted")
CorTestImage(rbind(OutputMGF$Jonchkeere[, 2], OutputMGF$Jonchkeere[, 1]/1000))
title(main = "GoIFISH Adjusted")
```

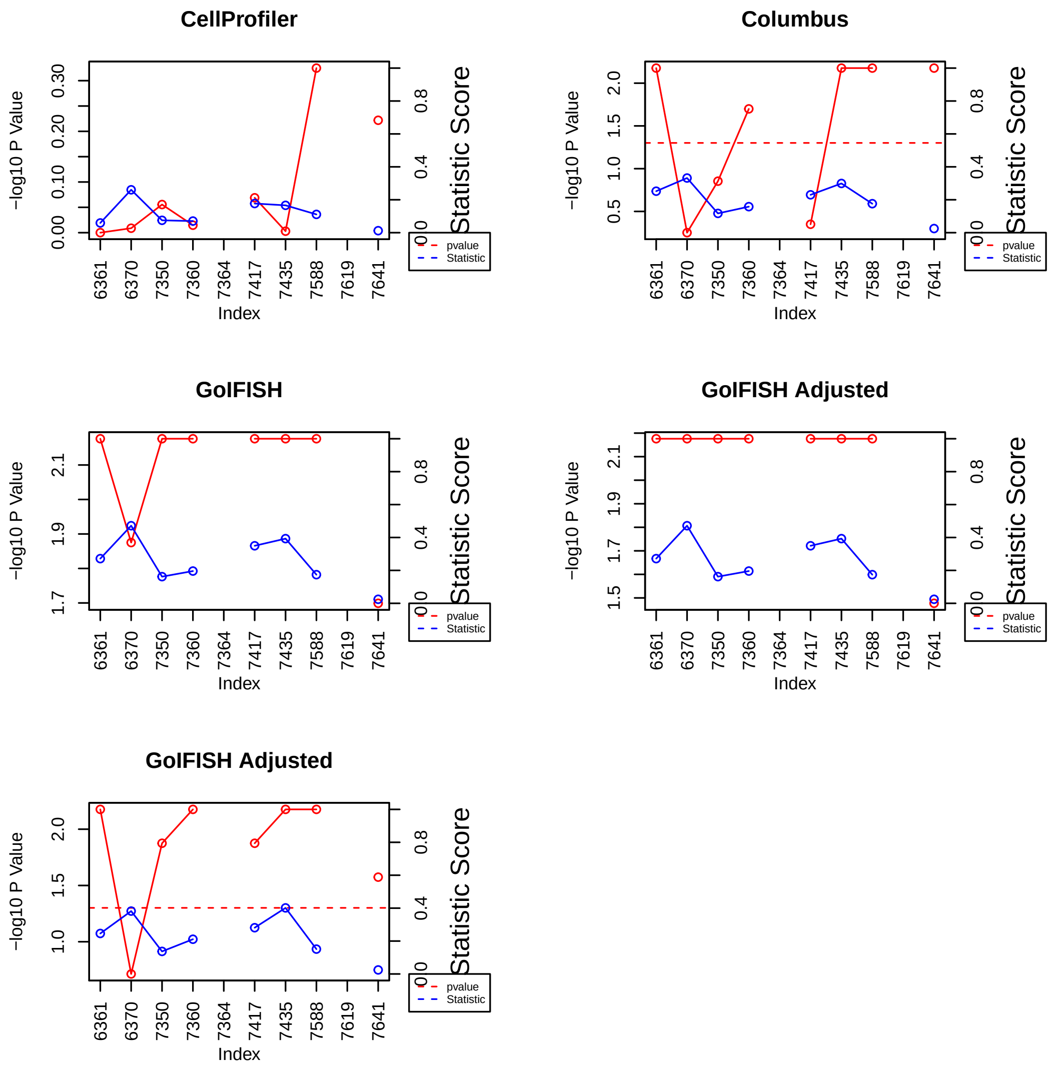

It appears that GoIFISH performs better than CellProfiler and Columbus in displaying an increasing trend within a single image. However globally, there is not much similarity between samples.

```
# Find number of pairwise samples which can be performed:
Nsamp = length(which(OutputCP$TukeyHSD[, 4] > 0.05))
Nsamp[2] = length(which(OutputGFAdj$TukeyHSD[, 4] > 0.05))
Nsamp[3] = length(which(OutputCol$TukeyHSD[, 4] > 0.05))
Nsamp[4] = length(which(OutputMGF$TukeyHSD[, 4] > 0.05))
Nsamp

## [1] 25 25 13 18

# Calculate Total number of samples:
```

```
nrow(OutputCP$TukeyHSD)

## [1] 28

# Check for the upper limit
Nsamp = length(which(OutputCP$TukeyUpper[, 4] > 0.05))
Nsamp[2] = length(which(OutputGFAdj$TukeyUpper[, 4] > 0.05))
Nsamp[3] = length(which(OutputCol$TukeyUpper[, 4] > 0.05))
Nsamp[4] = length(which(OutputMGF$TukeyUpper[, 4] > 0.05))
Nsamp

## [1] 21  6  6  6
```

It appears that direct comparisons can be made between two thirds of all pairwise comparisons

4.2.4 Combining all samples together

```
par(mfrow=c(3, 2))
CP_HER2=unlist(lapply(1:10, function(x) as.character(CP_Protein[[x]]$HER2.membrane.score
CP_HER2=cbind(CP_HER2, unlist(lapply(1:10, function(x)
  (CP_Protein[[x]]$HER2memInt))))

Columbus_HER2=unlist(lapply(1:10, function(x)
  as.character(Columbus_Protein[[x]]$HER2.membrane.score)))
Columbus_HER2=cbind(Columbus_HER2, unlist(lapply(1:10, function(x)
  (Columbus_Protein[[x]]$HER2memInt))))

GF_HER2=unlist(lapply(1:10, function(x)
   as.character(GF_Protein[[x]]$HER2.membrane.score)))
GF_HER2=cbind(GF_HER2, (unlist(lapply(1:10, function(x)
  GF_Protein[[x]]$HER2memInt))))
GF_HER2=cbind(GF_HER2, (unlist(lapply(1:10, function(x)
  as.character(GF_Protein[[x]]$HER2backAdj)))))

MGF_HER2=unlist(lapply(1:10, function(x) ManGF_Protein[[x]]$HER2.membrane.score))
MGF_HER2=cbind(MGF_HER2, as.numeric(unlist(lapply(1:10, function(x)
  ManGF_Protein[[x]]$HER2backAdj))))
# Plot the Boxplots of intensities
BoxPlotCompare(CP_HER2[ ,2], CP_HER2[ ,1], "CellProfiler")
BoxPlotCompare(Columbus_HER2[ ,2], Columbus_HER2[ ,1], "Columbus")
BoxPlotCompare(GF_HER2[ ,2], GF_HER2[ ,1], "GOIFISH")
BoxPlotCompare(GF_HER2[ ,3], GF_HER2[ ,1], "GOIFISH background adjusted")
```

```r
BoxPlotCompare(MGF_HER2[ ,2], MGF_HER2[ ,1], "Manual GOIFISH background adjusted")
```

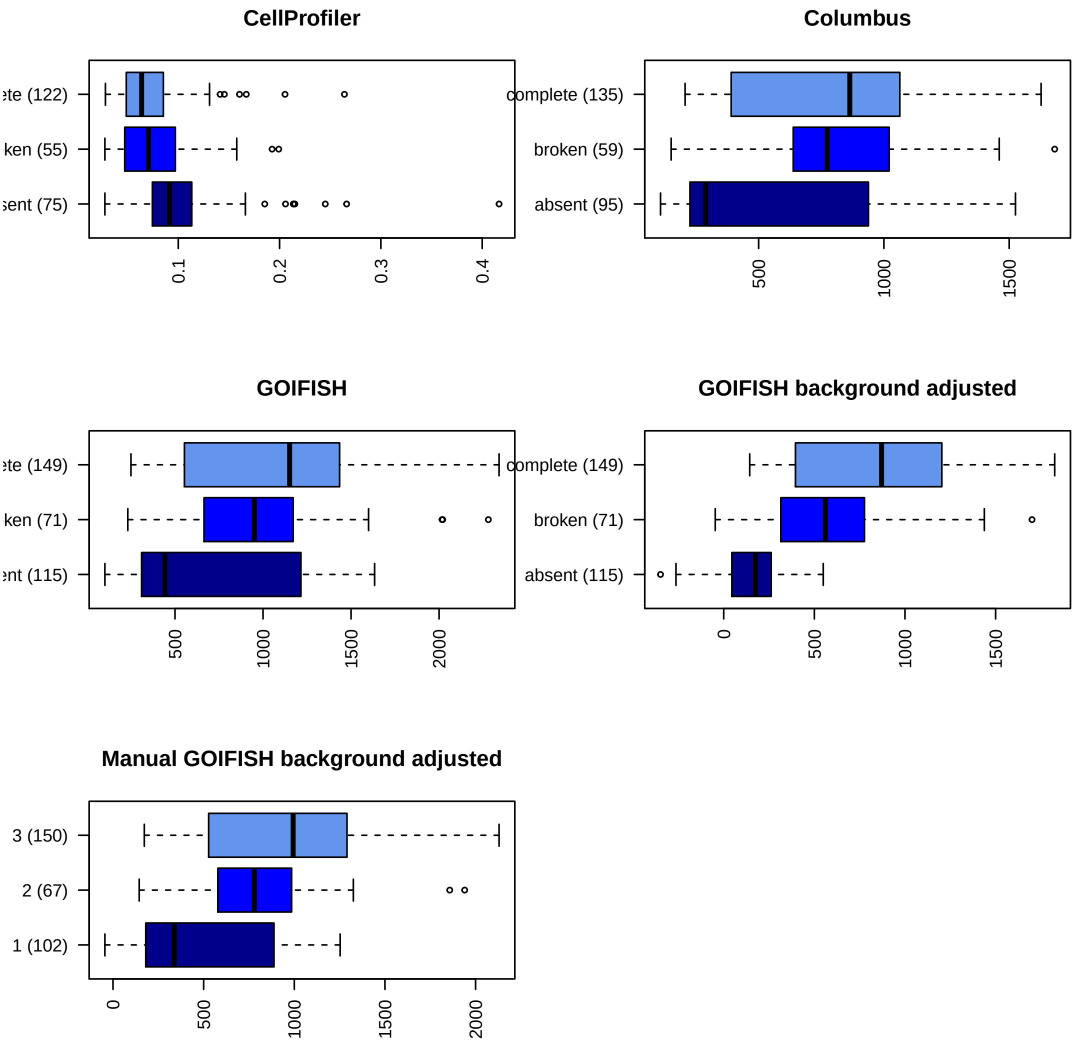

We need to check whether these differences are significant: We can run a series of T.tests for each sample

```r
par(mfrow = c(3, 2))
Matrix.Ttest(CP_HER2)
title(main = "CellProfiler")
Matrix.Ttest(Columbus_HER2)
title(main = "Columbus")
Matrix.Ttest(GF_HER2[, c(1, 2)])
title(main = "GoIFISH Raw")
Matrix.Ttest(GF_HER2[, c(1, 3)])
```

```
title(main = "GoIFISH BackAdjusted")
Matrix.Ttest(MGF_HER2[, c(1, 2)])
title(main = "Man GoIFISH Back Adjusted")
```

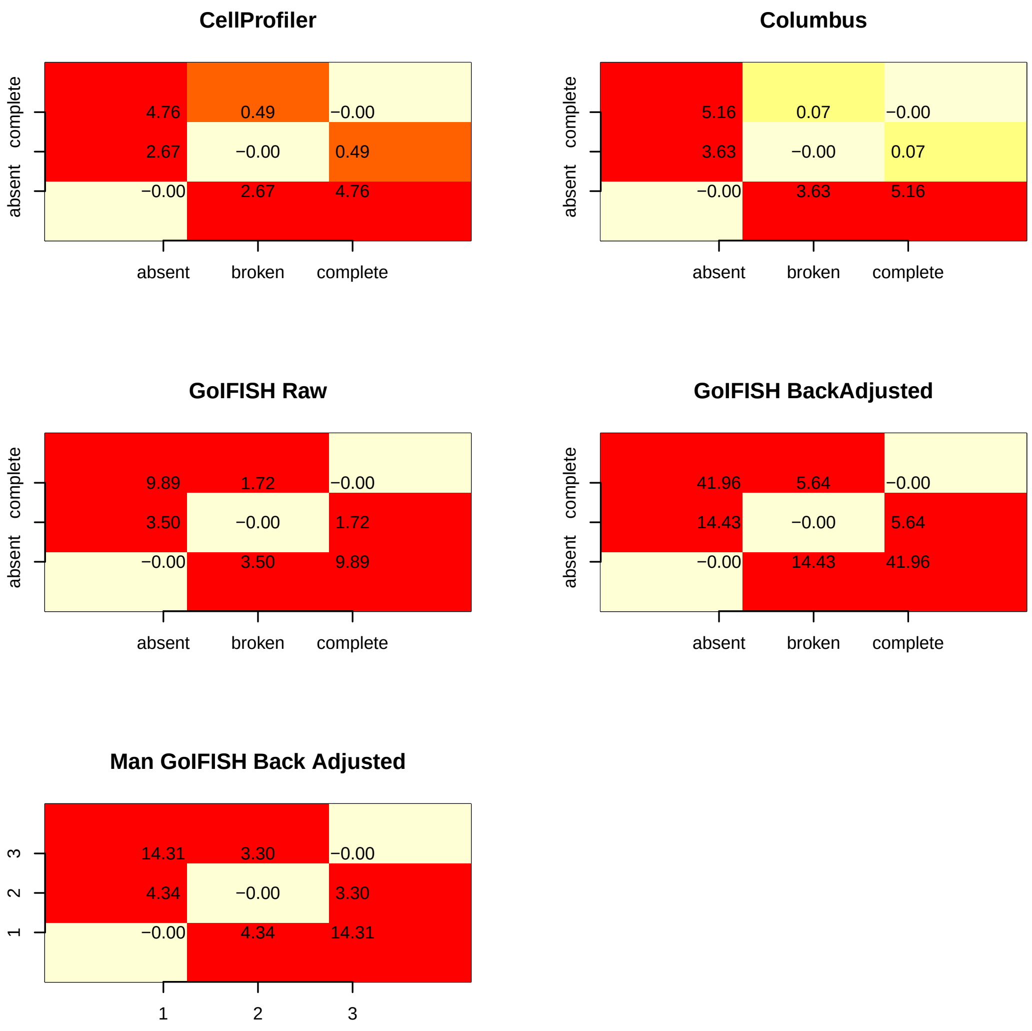

We see that after adjustment, the cells can be differentiated according to intensity in the GOIFISH and manually edited GOIFISH samples.

4.2.5 Looking at Higher Order Moments

We have so far investigated the mean, however, the membrane completeness could be described using higher order features including variance or skewness:

```r
## Look at Std Devs
A=IntensityComparison(CP_Protein, "HER2.membrane.score", "HER2_SD", F,
                      "Cellprofiler HER2 SD")
```

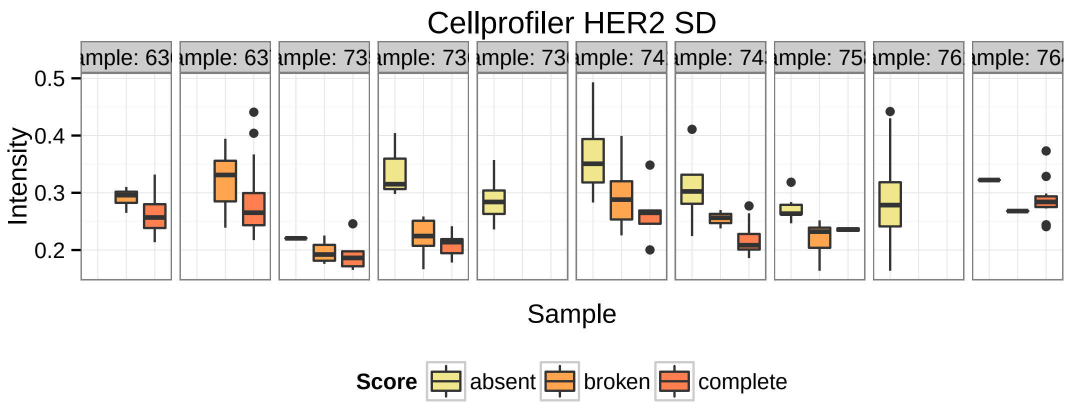


```r
B=IntensityComparison(Columbus_Protein, "HER2.membrane.score", "HER2_SD", F,
                      "Columbus HER2 SD")
```

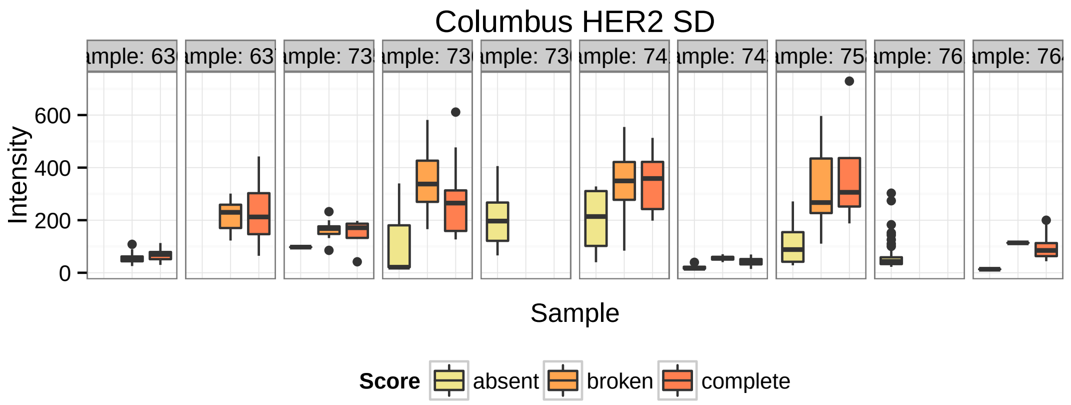


```r
C=IntensityComparison(GF_Protein, "HER2.membrane.score", "HER2_SD", F,
                      "GOIFISH HER2 SD")
```

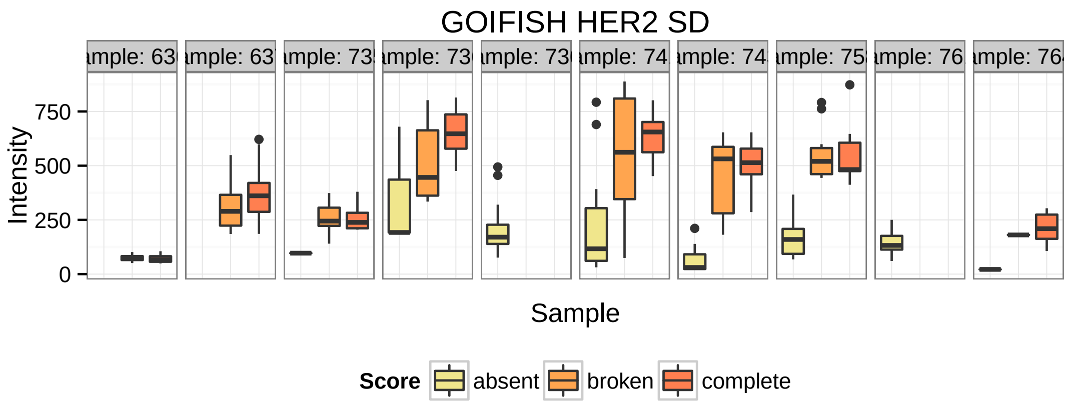


```
D=IntensityComparison(ManGF_Protein, "HER2.membrane.score", "HER2_SD", F,
                      "GOIFISH Manual HER2 SD")
```

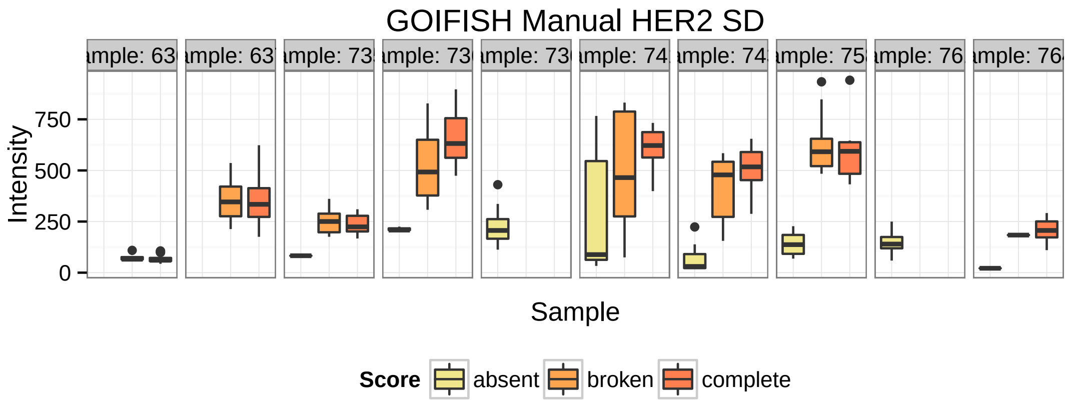


```
## Look at range
B2=IntensityComparison(Columbus_Protein, "HER2.membrane.score", "HER2_range",
                       F,"Columbus HER2 range" )
```

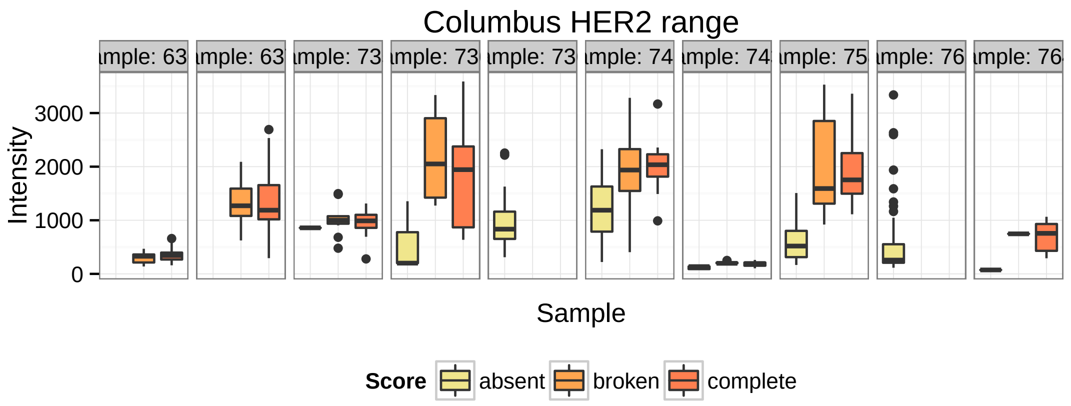


```
C2=IntensityComparison(GF_Protein, "HER2.membrane.score", "HER2_range",
                       F, "GOIFISH HER2 range")
```

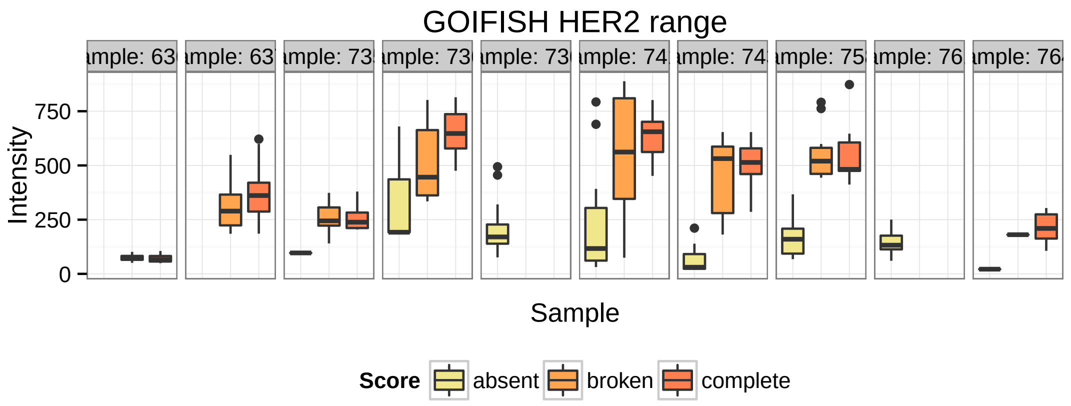


```
D2=IntensityComparison(ManGF_Protein, "HER2.membrane.score", "HER2_range",
                       F,"GOIFISH Manual HER2 range" )
```

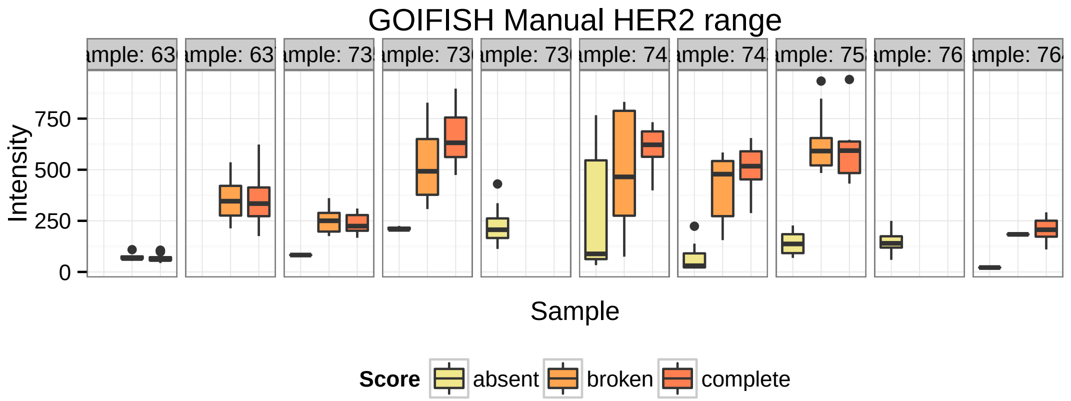


```
## Look at skewness
C3=IntensityComparison(GF_Protein, "HER2.membrane.score", "HER2_skewness", F, "GOIFISH HE
```

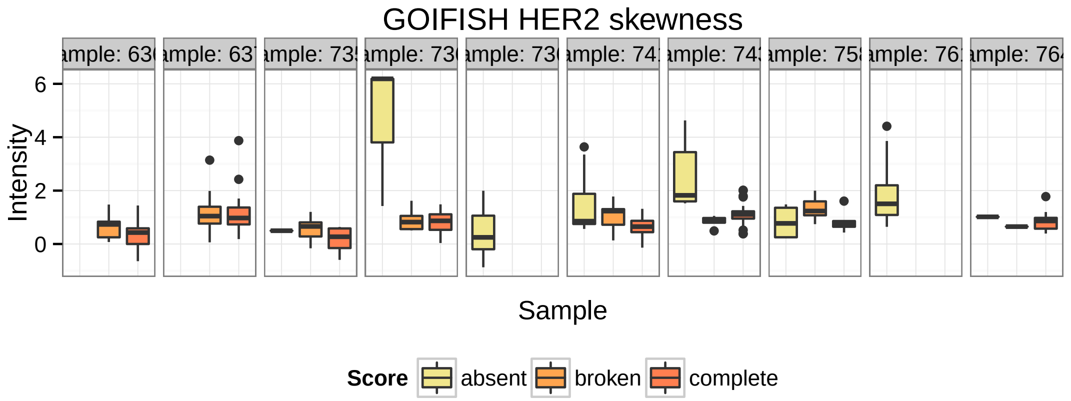


```
D3=IntensityComparison(ManGF_Protein, "HER2.membrane.score", "HER2_skewness", F, "GOIFISH
```

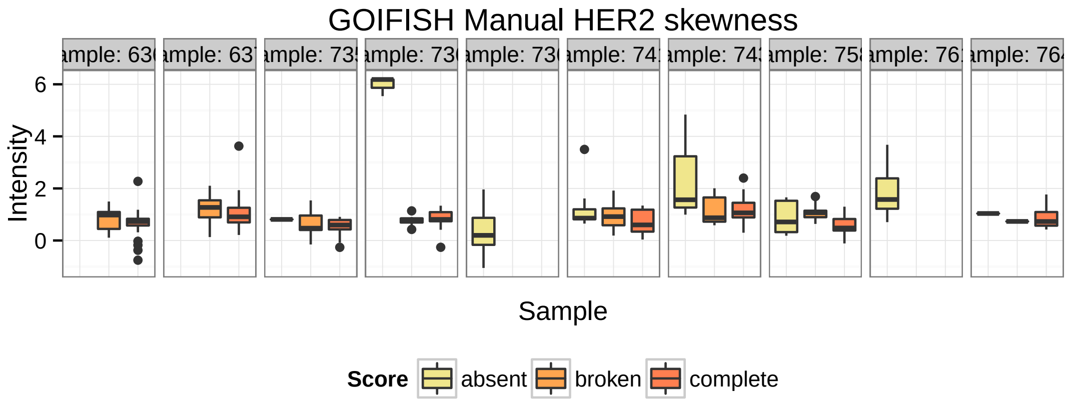


```
#Can also look at variance to mean ratio in order to standardise samples
CP_Protein=VarNormalise2(CP_Protein, "HER2_SD", "HER2memInt", "HER2varMeanRat")
Columbus_Protein=VarNormalise2(Columbus_Protein,"HER2_SD", "HER2memInt", "HER2varMeanRat
GF_Protein=VarNormalise2(GF_Protein,"HER2_SD", "HER2memInt",  "HER2varMeanRat")
ManGF_Protein=VarNormalise2(ManGF_Protein,"HER2_SD", "HER2memInt", "HER2varMeanRat")

A4=IntensityComparison(CP_Protein, "HER2.membrane.score", "HER2varMeanRat", F,
          "CellProfiler HER2 variance to mean Ratio" )
```

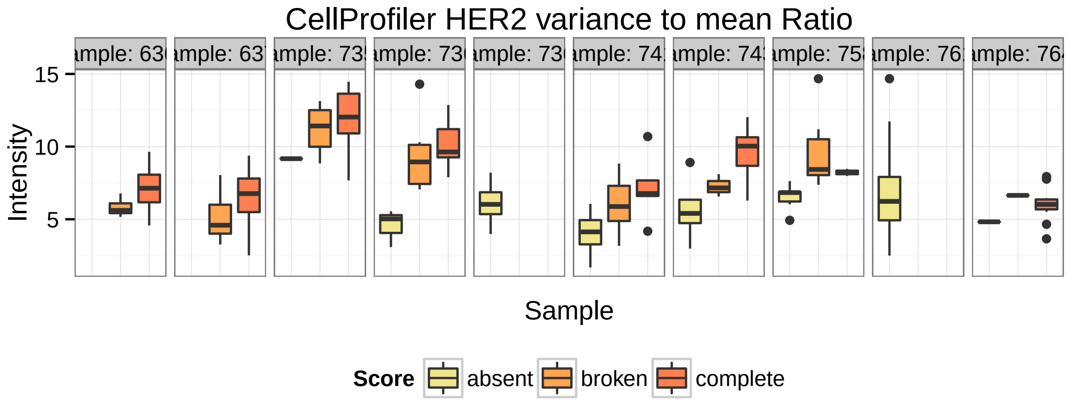


```
B4=IntensityComparison(Columbus_Protein, "HER2.membrane.score", "HER2varMeanRat",
                       F,"Columbus HER2 variance to mean Ratio")
```

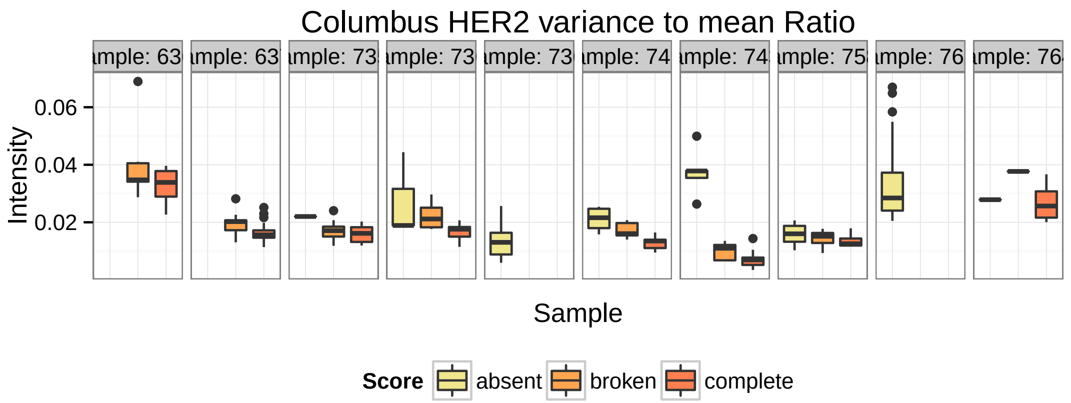


```
C4=IntensityComparison(GF_Protein, "HER2.membrane.score", "HER2varMeanRat", F,
                       "GOIFISH HER2 variance to mean ratio")
```

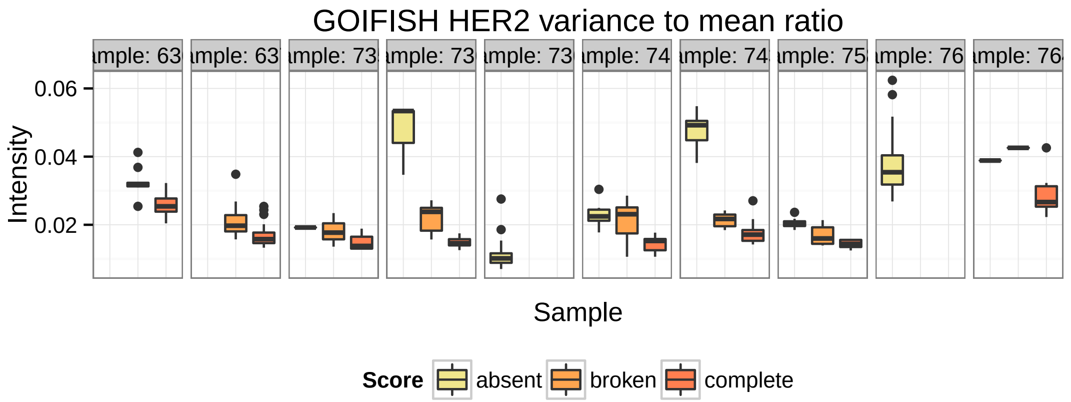


```
D4=IntensityComparison(ManGF_Protein, "HER2.membrane.score", "HER2varMeanRat",
                       F,"GOIFISH Manual HER2 variance to mean Ratio" )
```

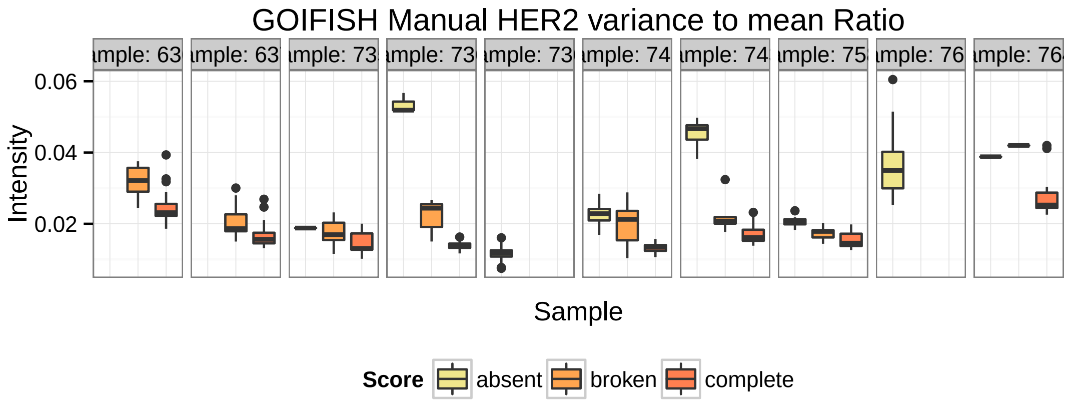

Compare after combining all these values whether the distribution is significant or not:

```
par(mfrow=c(3,2))

CP_HER2=cbind(CP_HER2, unlist(lapply(1:10, function(x) CP_Protein[[x]]$HER2_SD)))
CP_HER2=cbind(CP_HER2, unlist(lapply(1:10, function(x) CP_Protein[[x]]$HER2varMeanRat)))

Columbus_HER2=cbind(Columbus_HER2, unlist(lapply(1:10, function(x)
  Columbus_Protein[[x]]$HER2_SD)))
Columbus_HER2=cbind(Columbus_HER2, unlist(lapply(1:10, function(x)
  Columbus_Protein[[x]]$HER2varMeanRat)))

GF_HER2=cbind(GF_HER2, as.numeric(unlist(lapply(1:10, function(x)
  GF_Protein[[x]]$HER2_SD))))
GF_HER2=cbind(GF_HER2, as.numeric(unlist(lapply(1:10, function(x)
  GF_Protein[[x]]$HER2_range))))
GF_HER2=cbind(GF_HER2, as.numeric(unlist(lapply(1:10, function(x)
  GF_Protein[[x]]$HER2_skewness))))
GF_HER2=cbind(GF_HER2, as.numeric(unlist(lapply(1:10, function(x)
  GF_Protein[[x]]$HER2varMeanRat))))

MGF_HER2=cbind(MGF_HER2, as.numeric(unlist(lapply(1:10, function(x)
  ManGF_Protein[[x]]$HER2_SD))))
MGF_HER2=cbind(MGF_HER2, as.numeric(unlist(lapply(1:10, function(x)
  ManGF_Protein[[x]]$HER2_range))))
MGF_HER2=cbind(MGF_HER2, as.numeric(unlist(lapply(1:10, function(x)
  ManGF_Protein[[x]]$HER2_skewness))))
MGF_HER2=cbind(MGF_HER2, as.numeric(unlist(lapply(1:10, function(x)
  ManGF_Protein[[x]]$HER2varMeanRat))))
```

```
##SD boxplots
BoxPlotCompare(GF_HER2[ ,4], GF_HER2[ ,1], "GOIFISH sd")
BoxPlotCompare(MGF_HER2[ ,3], MGF_HER2[ ,1], "Manual GOIFISH sd")
BoxPlotCompare(Columbus_HER2[ ,3], Columbus_HER2[ ,1], "Columbus sd")
BoxPlotCompare(CP_HER2[ ,3], CP_HER2[ ,1], "Cellprofiler sd")
## Range boxplots
BoxPlotCompare(GF_HER2[ ,5], GF_HER2[ ,1], "GOIFISH range")
BoxPlotCompare(MGF_HER2[ ,4], MGF_HER2[ ,1], "Manual GOIFISH range")
```

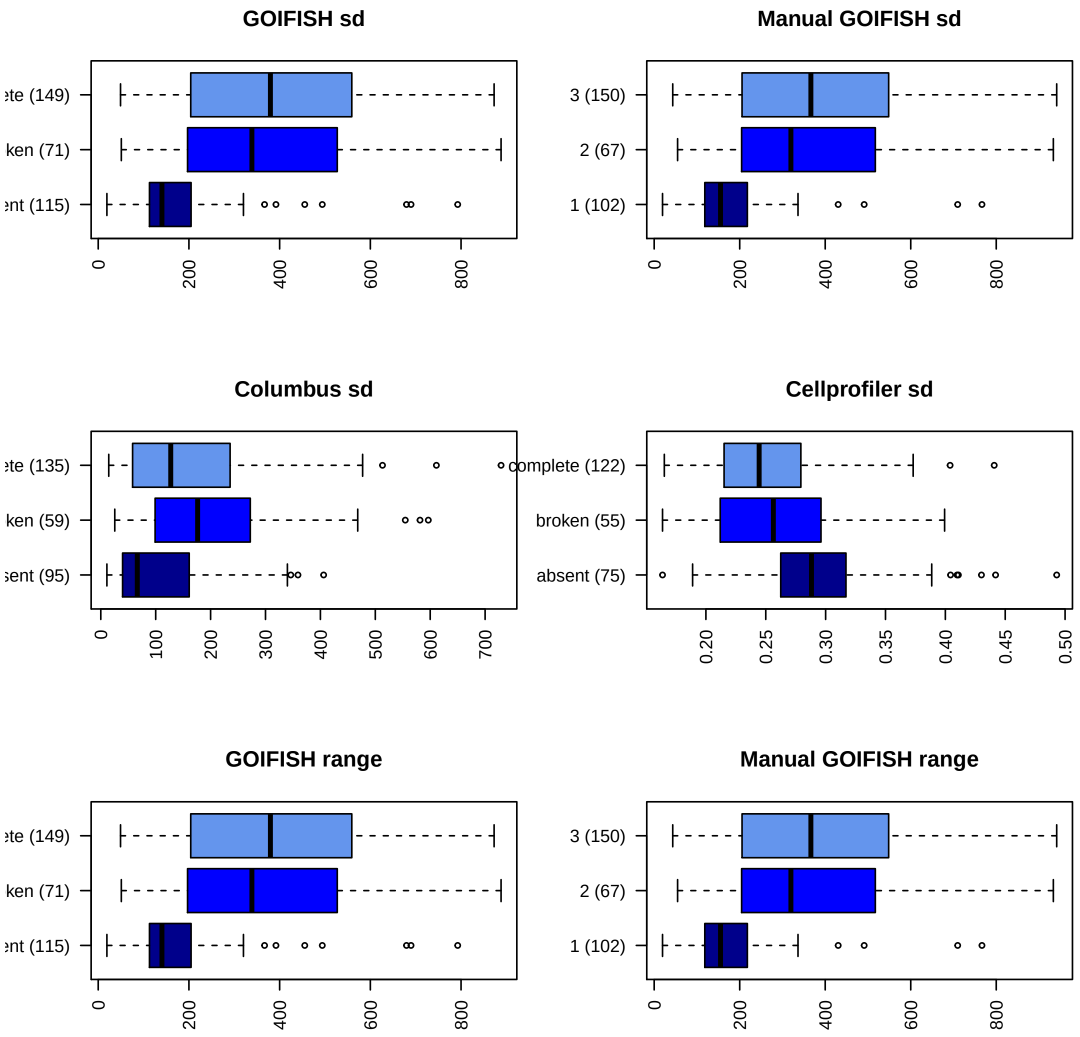


```
## skewness boxplots
BoxPlotCompare(GF_HER2[ ,6], GF_HER2[ ,1], "GOIFISH skewness")
```

```r
BoxPlotCompare(MGF_HER2[ ,5], MGF_HER2[ ,1], "Manual GOIFISH skewness")
## coefficient of variance boxplots
BoxPlotCompare(GF_HER2[ ,7], GF_HER2[ ,1], "GOIFISH var to mean")
BoxPlotCompare(MGF_HER2[ ,6], MGF_HER2[ ,1], "Manual GOIFISH var to mean")
BoxPlotCompare(Columbus_HER2[ ,4], Columbus_HER2[ ,1], "Columbus var to mean")
BoxPlotCompare(CP_HER2[ ,4], CP_HER2[ ,1], "cellprofiler var to mean")
```

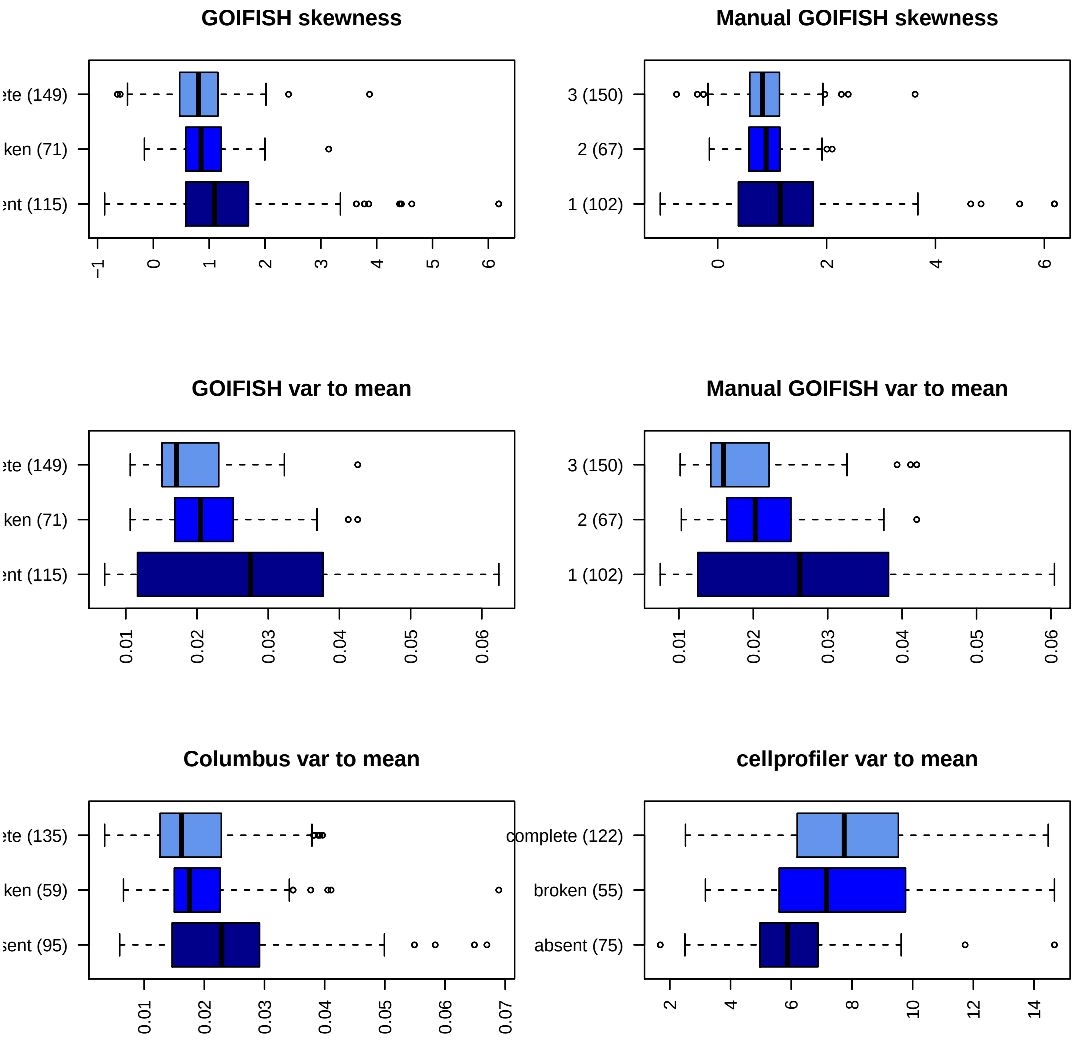

Perform t-tests to check whether any of the above trends are significant:

```r
par(mfrow = c(3, 2))
Matrix.Ttest(GF_HER2[, c(1, 4)])
title(main = "GoIFISH variance")
Matrix.Ttest(MGF_HER2[, c(1, 3)])
```

```
title(main = "Man GoIFISH variance")
Matrix.Ttest(GF_HER2[, c(1, 5)])
title(main = "GoIFISH range")
Matrix.Ttest(MGF_HER2[, c(1, 4)])
title(main = "Man GoIFISH range")
Matrix.Ttest(GF_HER2[, c(1, 6)])
title(main = "GoIFISH skewness")
Matrix.Ttest(MGF_HER2[, c(1, 5)])
title(main = "Man GoIFISH skewness")
```

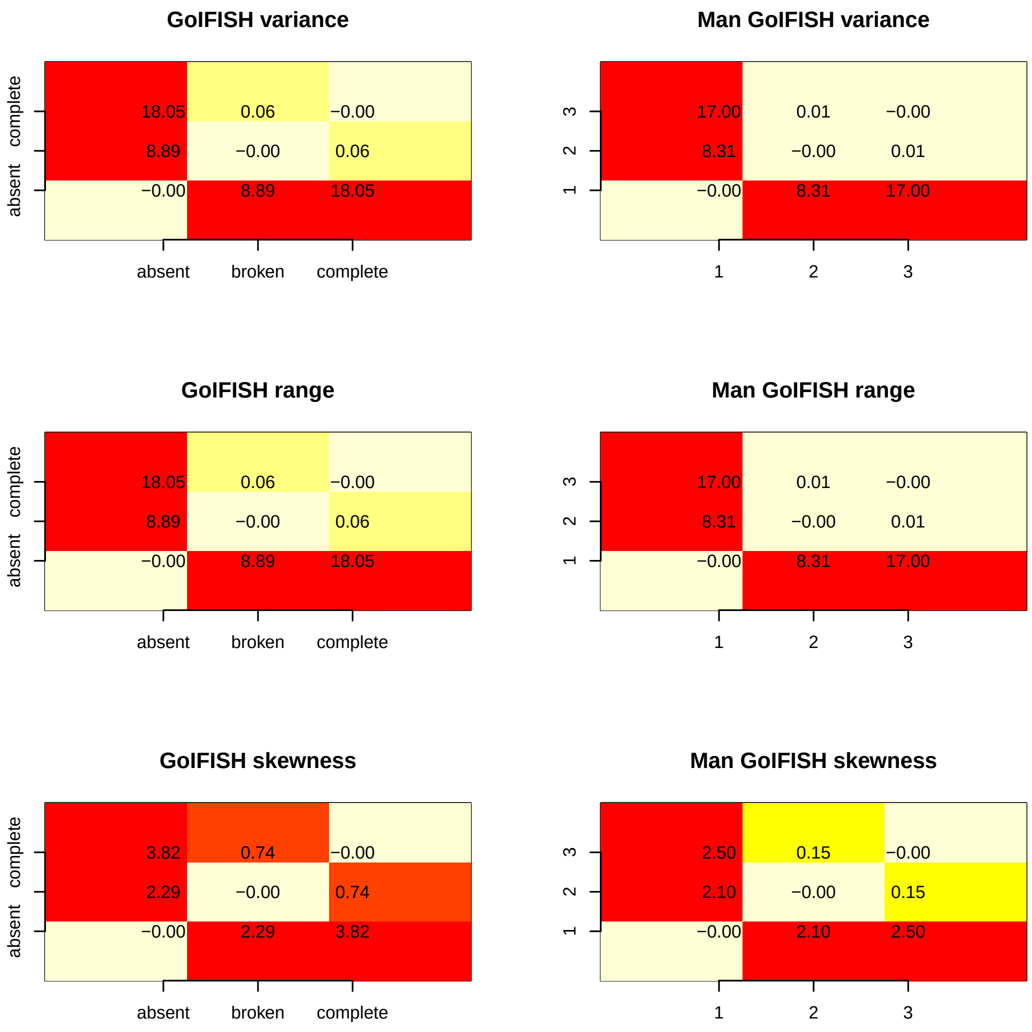

```
Matrix.Ttest(GF_HER2[, c(1, 7)])
title(main = "GoIFISH std/mean")
Matrix.Ttest(MGF_HER2[, c(1, 6)])
title(main = "Man GoIFISH std/mean")
Matrix.Ttest(Columbus_HER2[, c(1, 3)])
title(main = "Columbus sd")
Matrix.Ttest(Columbus_HER2[, c(1, 4)])
title(main = "Columbus sd/mean ratio")
Matrix.Ttest(CP_HER2[, c(1, 3)])
title(main = "CP sd")
Matrix.Ttest(CP_HER2[, c(1, 4)])
title(main = "CP sd/mean ratio")
```

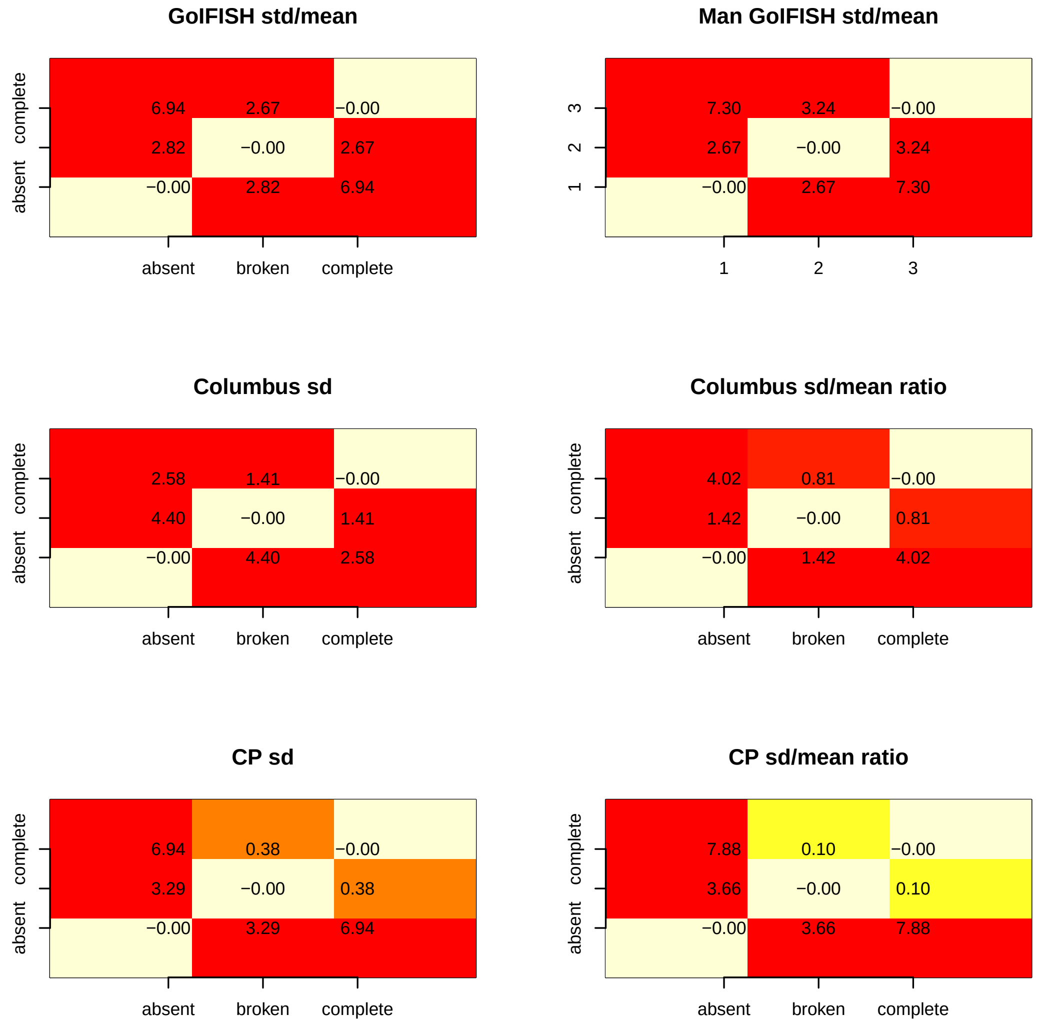

Note that the p values may be highly dependent on the sample sizes used. We can make a plot to see what their distributions are, and whether we should have neglected any specific analyses

```
HER2pos = sapply(1:10, function(x) table(na.omit(CP_Protein[[x]][, "HER2.prot.score"])))
HER2mem = sapply(1:10, function(x) summary(na.omit(CP_Protein[[x]][, "HER2.membrane.score
par(mfrow = c(1, 2))
# do the barplots
barplot(HER2pos, names = names(CP_Protein), col = c(1, 2), las = 2)
legend("topright", levels(CP_Protein[[1]][, "HER2.prot.score"]), col = c(1:2),
    lwd = 2)
barplot(HER2mem, names = names(CP_Protein), col = c(1:4), las = 3)
legend("topright", levels(CP_Protein[[1]][, "HER2.membrane.score"]), col = c(1:4),
    lwd = 2)
```

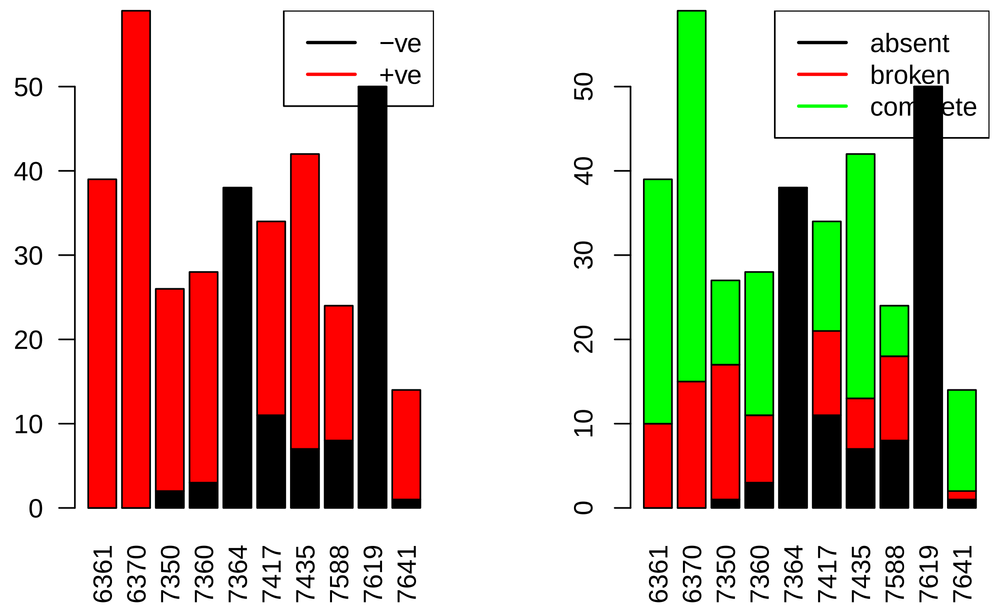

Samples 7364 and 7419 may not have been useful to the above analysis due to low numbers of positive cells

5 Accuracy in Spot Detection: Measuring intra-tumour heterogeneity

Here, we wish to investigate how closely manual detection or counting of spots correlate with automated detection of spots. We look at comparing at the cell level, detection rates for copy number, spot areas and intensities. For each cell, spot area, spot number and average intensity (using Channel3 or 5 respectively) were determined.

If we load the dataset, we notice that there is a list of 10 dataframes containing the merged CN data from manual and from automated analysis. Looking at the column names, we see that the main ouputs from the manual counting are copynumber and a class status (0 is the weakest and 3 is the strongest. For sample 6370, we see 33 cells have high HER2 spot expression, indicating higher copy number in these samples). From Columbus segmentation, there are 6 variables: copy number, spot area and spot intensity for both HER2 and for cent17. In all analyses, correlation testing will be performed only in cells with good segmentation results to ensure that nuclear segmentation does not confound the results.

We can now do some analysis on copy number in the manual and the columbus analyses:

5.1 Correlating copy number between raw and counted values

Here, we determine whether the number of spots using each method correlate: Note that some samples are reported to be 'amplified' and a distinct number of copy numbers connot be attained manually in these samples. We set these values arbitrarily at 21. First, we look at individual cent17 samples

```
## Is there a direct correlation between manual and counts
par(mfrow = c(5, 4))
# Plot CP cent 17
for (i in 1:10) {
    smoothScatter(CP_CN[[i]]$cent17_CN_spot, CP_CN[[i]]$cent17.CN2, xlab = "CP",
        ylab = "Manual", main = sprintf("cent17_%s", names(CP_CN)[i]))
    abline(0, 1)
}

## KernSmooth 2.23 loaded
## Copyright M. P. Wand 1997-2009

# Columbus cent 17
for (i in 1:10) {
    smoothScatter(Columbus_CN[[i]]$cent17_CN_spot, Columbus_CN[[i]]$cent17.CN2,
        xlab = "ColM", ylab = "Manual", main = sprintf("cent17_%s", names(CP_CN)[i]))
    abline(0, 1)
}
```

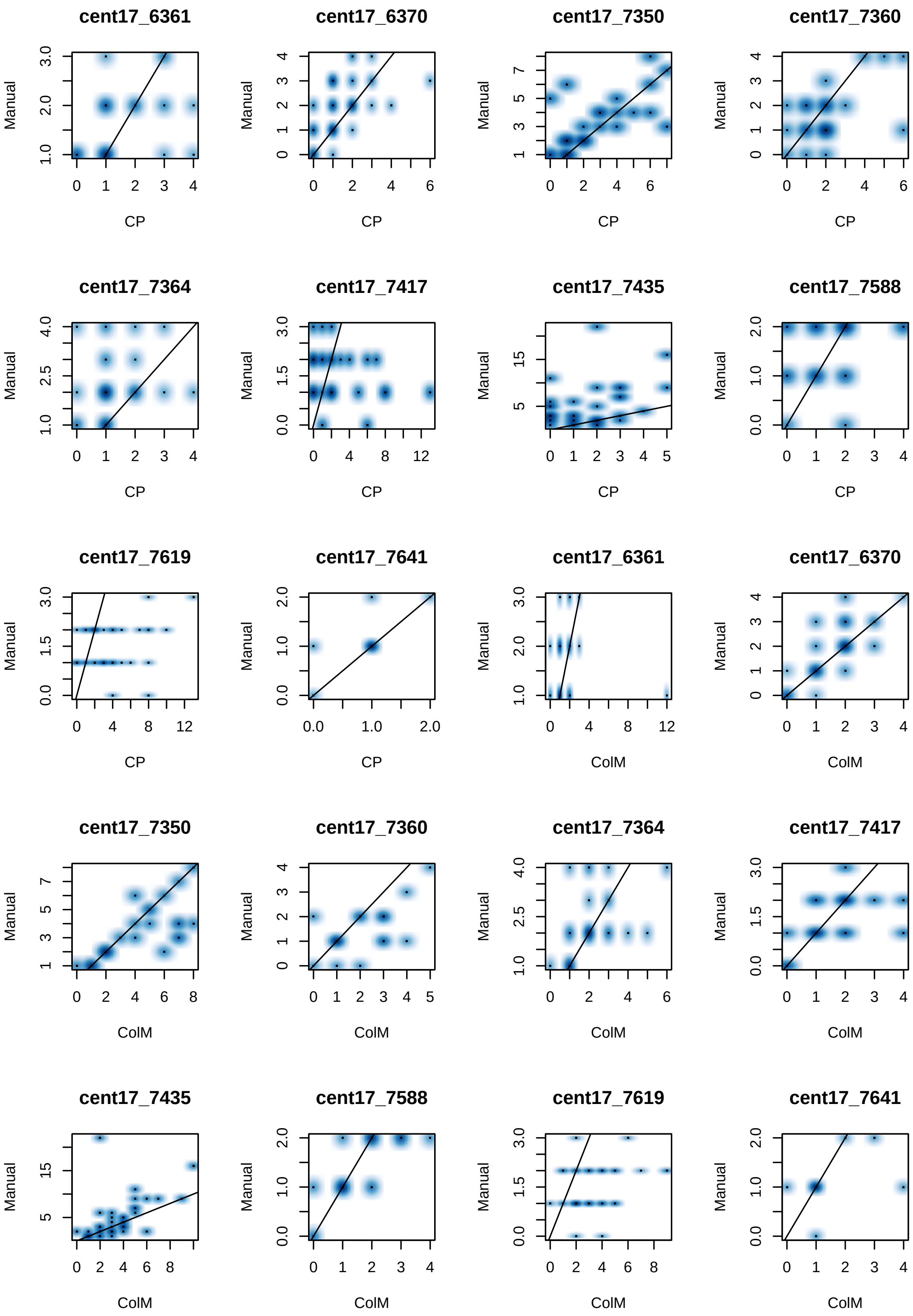
cent17_6361
cent17_6370
cent17_7350
cent17_7360
cent17_7364
cent17_7417
cent17_7435
cent17_7588
cent17_7619
cent17_7641
cent17_6361
cent17_6370
cent17_7350
cent17_7360
cent17_7364
cent17_7417
cent17_7435
cent17_7588
cent17_7619
cent17_7641
Manual
CP
ColM

```r
# Goifish cent 17

for (i in 1:10) {
    smoothScatter(GF_CN[[i]]$cent17_CN_spot, GF_CN[[i]]$cent17.CN2, xlab = "GF",
        ylab = "Manual", main = sprintf("cent17_%s", names(CP_CN)[i]))
    abline(0, 1)
}
# Plot Goifish manually edited cent 17
for (i in 1:10) {
    smoothScatter(ManGF_CN[[i]]$cent17_CN_spot, ManGF_CN[[i]]$cent17.CN2, xlab = "ManGF"
        ylab = "Manual", main = sprintf("cent17_%s", names(CP_CN)[i]))
    abline(0, 1)
}
```

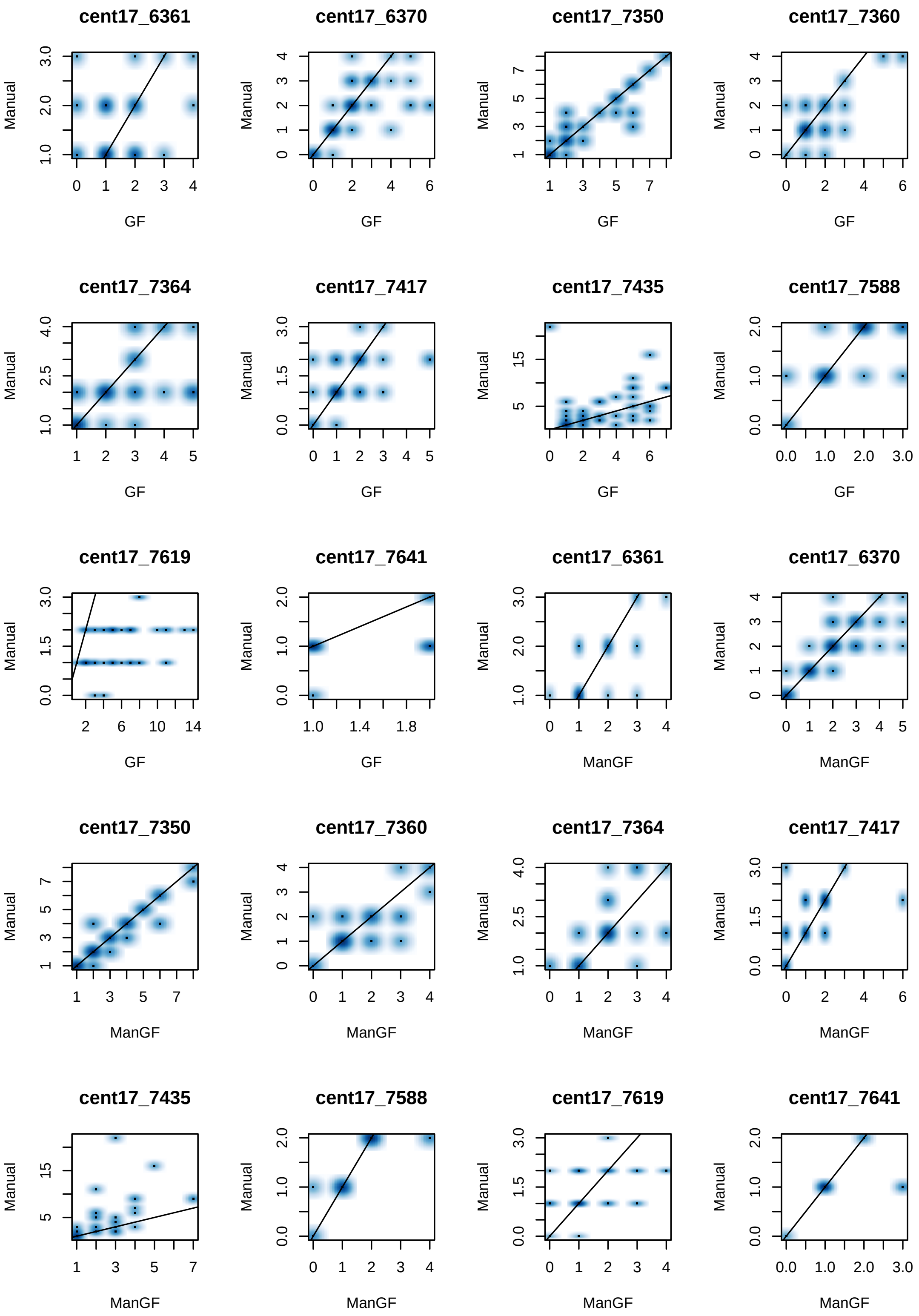
cent17_6361
cent17_6370
cent17_7350
cent17_7360
cent17_7364
cent17_7417
cent17_7435
cent17_7588
cent17_7619
cent17_7641
Manual
GF
cent17_6361
cent17_6370
cent17_7350
cent17_7360
cent17_7364
cent17_7417
cent17_7435
cent17_7588
cent17_7619
cent17_7641
Manual
ManGF

We can do the same with HER2 spots:

```
par(mfrow = c(5, 4))
# Cell Profiler
for (i in 1:10) {
    smoothScatter(CP_CN[[i]]$ERBB2_CN_spot, CP_CN[[i]]$ERBB2.CN2, xlab = "CP",
        ylab = "Manual", main = sprintf("HER2_%s", names(CP_CN)[i]))
    abline(0, 1)
}
# COlumbus
for (i in 1:10) {
    smoothScatter(Columbus_CN[[i]]$ERBB2_CN_spot, Columbus_CN[[i]]$ERBB2.CN2,
        xlab = "Col", ylab = "Manual", main = sprintf("HER2_%s", names(CP_CN)[i]))
    abline(0, 1)
}
```

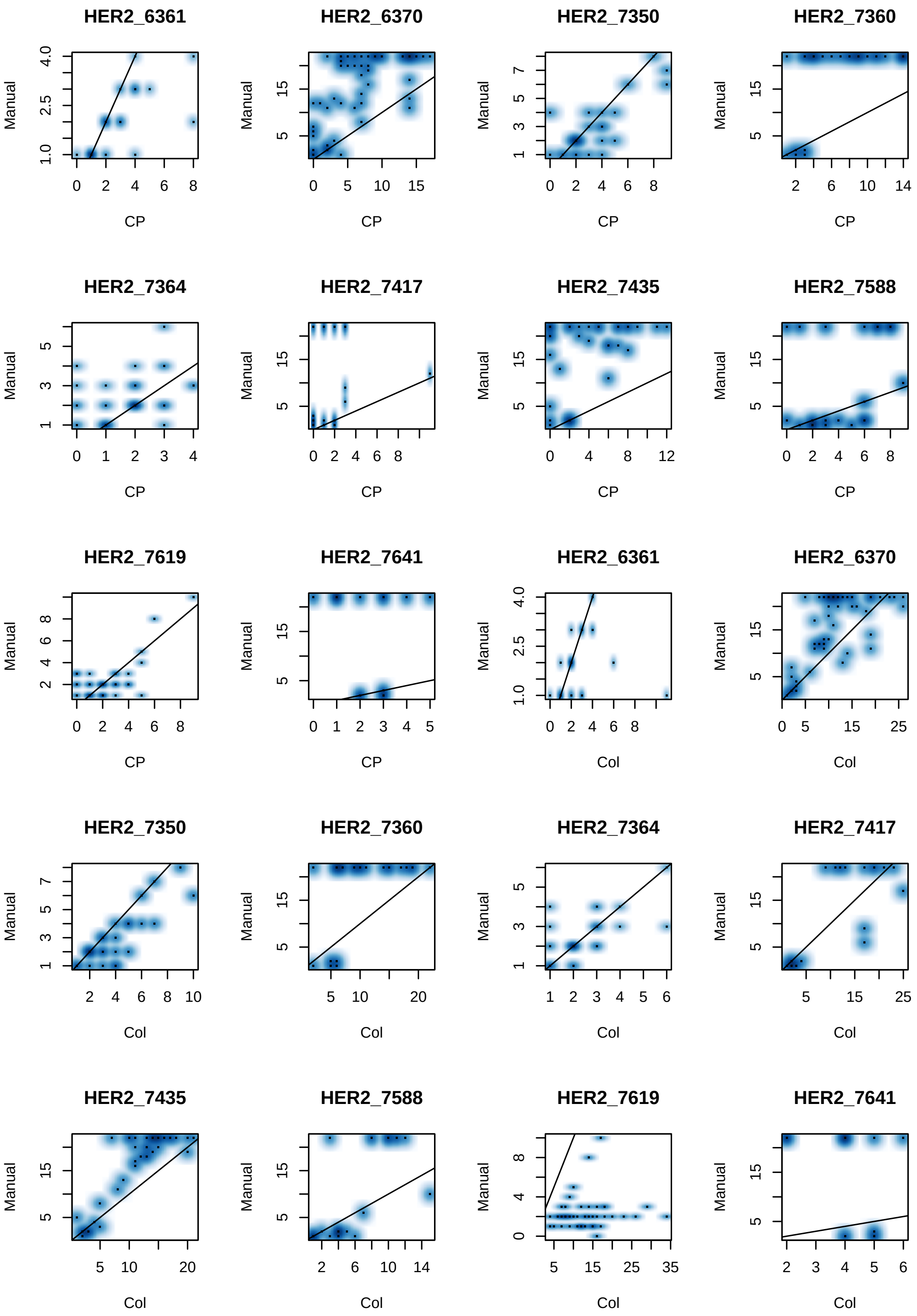
HER2_6361
HER2_6370
HER2_7350
HER2_7360
HER2_7364
HER2_7417
HER2_7435
HER2_7588
HER2_7619
HER2_7641
HER2_6361
HER2_6370
HER2_7350
HER2_7360
HER2_7364
HER2_7417
HER2_7435
HER2_7588
HER2_7619
HER2_7641
Manual
CP
Col

```r
# Goifish
for (i in 1:10) {
    smoothScatter(GF_CN[[i]]$ERBB2_CN_spot, GF_CN[[i]]$ERBB2.CN2, xlab = "GF",
        ylab = "Manual", main = sprintf("HER2_%s", names(CP_CN)[i]))
    abline(0, 1)
}
# Manual Goifish
for (i in 1:10) {
    smoothScatter(ManGF_CN[[i]]$ERBB2_CN_spot, ManGF_CN[[i]]$ERBB2.CN2, xlab = "GF",
        ylab = "Manual", main = sprintf("HER2_%s", names(CP_CN)[i]))
    abline(0, 1)
}
```

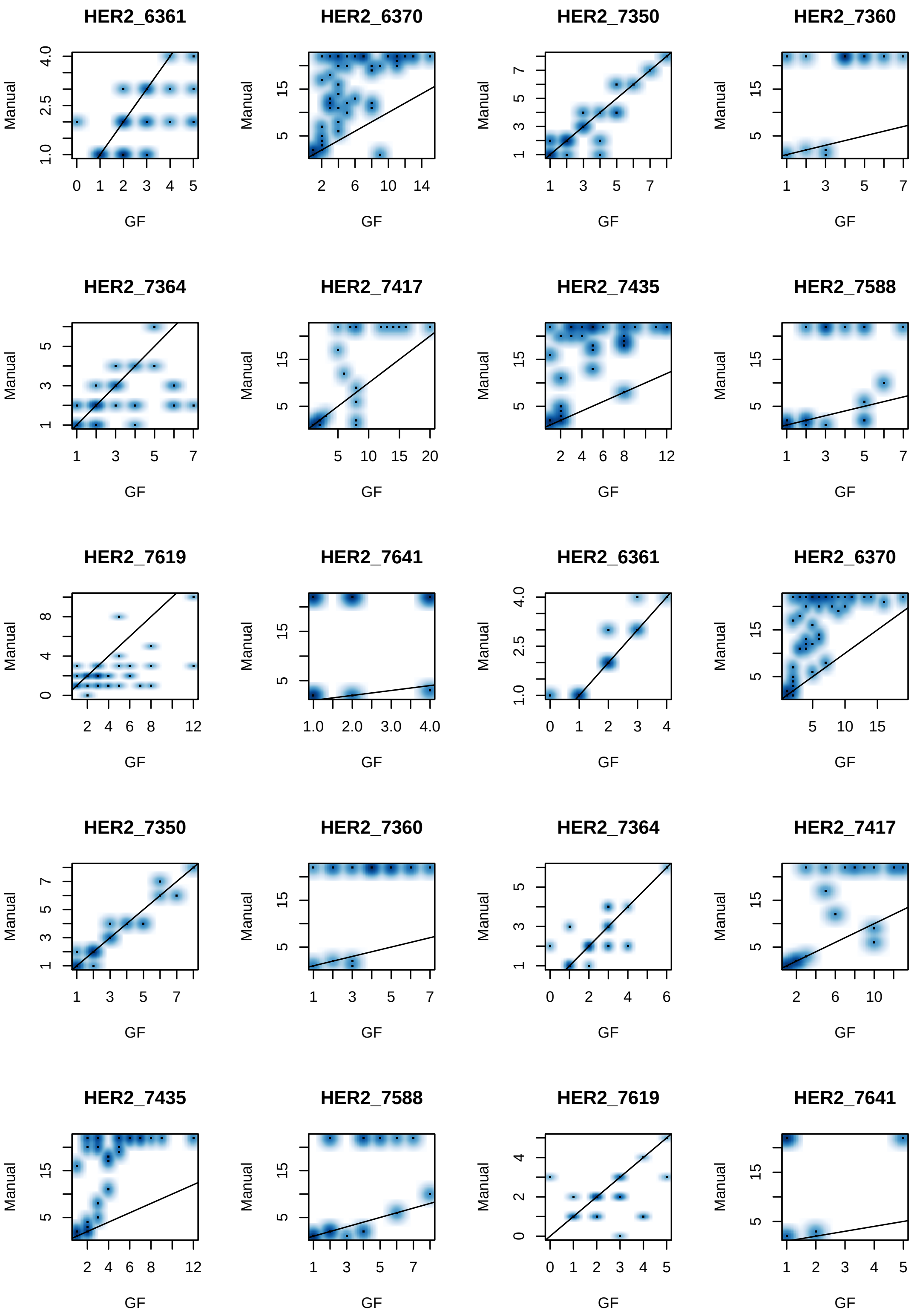
HER2_6361
HER2_6370
HER2_7350
HER2_7360
HER2_7364
HER2_7417
HER2_7435
HER2_7588
HER2_7619
HER2_7641
HER2_6361
HER2_6370
HER2_7350
HER2_7360
HER2_7364
HER2_7417
HER2_7435
HER2_7588
HER2_7619
HER2_7641
Manual
GF

Note that the darker area in this plot correspond to a larger number of overlapping points. The correlation appears to be variable depending onthe sample used: For example, smaple 7619 had an over-exposed Cent17 channel resulting in a high false positive rate and should be discarded from further analysis.

We can summarise the direct correlation coefficient for all samples, by stacking them together:

```
## CellProfiler
CP_spot = unlist(lapply(c(1:8, 10), function(x) CP_CN[[x]]$cent17.CN2))
CP_spot = cbind(CP_spot, unlist(lapply(c(1:8, 10), function(x) CP_CN[[x]]$ERBB2.CN2)))
CP_spot = cbind(CP_spot, unlist(lapply(c(1:8, 10), function(x) CP_CN[[x]]$cent17_CN_spot
CP_spot = cbind(CP_spot, unlist(lapply(c(1:8, 10), function(x) CP_CN[[x]]$ERBB2_CN_spot)
CP_spot = cbind(CP_spot, unlist(lapply(c(1:8, 10), function(x) CP_CN[[x]]$cent17.CN2)))
CP_spot = cbind(CP_spot, unlist(lapply(c(1:8, 10), function(x) CP_CN[[x]]$ERBB2.CN2)))
CP_spot[, 5:6] = sapply(5:6, function(x) replace(CP_spot[, x], 22, NA))
CP_spot_idx = which(unlist(lapply(c(1:8, 10), function(x) CP_CN[[x]][, 3])) >
    0)
## Columbus
Col_spot = unlist(lapply(c(1:8, 10), function(x) Columbus_CN[[x]]$cent17.CN2))
Col_spot = cbind(Col_spot, unlist(lapply(c(1:8, 10), function(x) Columbus_CN[[x]]$ERBB2.
Col_spot = cbind(Col_spot, unlist(lapply(c(1:8, 10), function(x) Columbus_CN[[x]]$cent17
Col_spot = cbind(Col_spot, unlist(lapply(c(1:8, 10), function(x) Columbus_CN[[x]]$ERBB2_
Col_spot = cbind(Col_spot, unlist(lapply(c(1:8, 10), function(x) Columbus_CN[[x]]$cent17
Col_spot = cbind(Col_spot, unlist(lapply(c(1:8, 10), function(x) Columbus_CN[[x]]$ERBB2.
Col_spot[, 5:6] = sapply(5:6, function(x) replace(Col_spot[, x], 22, NA))
Col_spot_idx = which(unlist(lapply(c(1:8, 10), function(x) Columbus_CN[[x]][,
    3])) > 0)
## GoIFISH
GF_Spot = unlist(lapply(c(1:8, 10), function(x) GF_CN[[x]]$cent17.CN2))
GF_Spot = cbind(GF_Spot, unlist(lapply(c(1:8, 10), function(x) GF_CN[[x]]$ERBB2.CN2)))
GF_Spot = cbind(GF_Spot, unlist(lapply(c(1:8, 10), function(x) GF_CN[[x]]$cent17_CN_spot
GF_Spot = cbind(GF_Spot, unlist(lapply(c(1:8, 10), function(x) GF_CN[[x]]$ERBB2_CN_spot)
GF_Spot = cbind(GF_Spot, unlist(lapply(c(1:8, 10), function(x) GF_CN[[x]]$cent17.CN2)))
GF_Spot = cbind(GF_Spot, unlist(lapply(c(1:8, 10), function(x) GF_CN[[x]]$ERBB2.CN2)))
GF_Spot[, 5:6] = sapply(5:6, function(x) replace(GF_Spot[, x], 22, NA))
GF_spot_idx = which(unlist(lapply(c(1:8, 10), function(x) GF_CN[[x]][, 3])) >
    0)
## Manual GoIFISH
MGF_Spot = unlist(lapply(c(1:8, 10), function(x) ManGF_CN[[x]]$cent17.CN2))
MGF_Spot = cbind(MGF_Spot, unlist(lapply(c(1:8, 10), function(x) ManGF_CN[[x]]$ERBB2.CN2
MGF_Spot = cbind(MGF_Spot, unlist(lapply(c(1:8, 10), function(x) ManGF_CN[[x]]$cent17_CN
MGF_Spot = cbind(MGF_Spot, unlist(lapply(c(1:8, 10), function(x) ManGF_CN[[x]]$ERBB2_CN_
MGF_Spot = cbind(MGF_Spot, unlist(lapply(c(1:8, 10), function(x) ManGF_CN[[x]]$cent17.CN
MGF_Spot = cbind(MGF_Spot, unlist(lapply(c(1:8, 10), function(x) ManGF_CN[[x]]$ERBB2.CN2
```

```r
MGF_Spot[, 5:6] = sapply(5:6, function(x) replace(MGF_Spot[, x], 22, NA))
MGF_spot_idx = which(unlist(lapply(c(1:8, 10), function(x) ManGF_CN[[x]][, 3])) >
    0)

## STart making plots here
par(mfrow = c(3, 3))
SmoothScatterCompare(CP_spot[-CP_spot_idx, 3], CP_spot[-CP_spot_idx, 1], "cent17",
    "CellProfiler")
SmoothScatterCompare(CP_spot[-CP_spot_idx, 4], CP_spot[-CP_spot_idx, 2], "her2",
    "CellProfiler")
SmoothScatterCompare(Col_spot[-Col_spot_idx, 3], Col_spot[-Col_spot_idx, 1],
    "cent17", "ColM")
SmoothScatterCompare(Col_spot[-Col_spot_idx, 4], Col_spot[-Col_spot_idx, 2],
    "her2", "ColM")
SmoothScatterCompare(GF_Spot[-GF_spot_idx, 3], GF_Spot[-GF_spot_idx, 1], "cent17",
    "GF")
SmoothScatterCompare(GF_Spot[-GF_spot_idx, 4], GF_Spot[-GF_spot_idx, 2], "her2",
    "GF")
SmoothScatterCompare(MGF_Spot[-GF_spot_idx, 3], MGF_Spot[-GF_spot_idx, 1], "cent17",
    "MGF")
SmoothScatterCompare(MGF_Spot[-GF_spot_idx, 4], MGF_Spot[-GF_spot_idx, 2], "her2",
    "MGF")
```

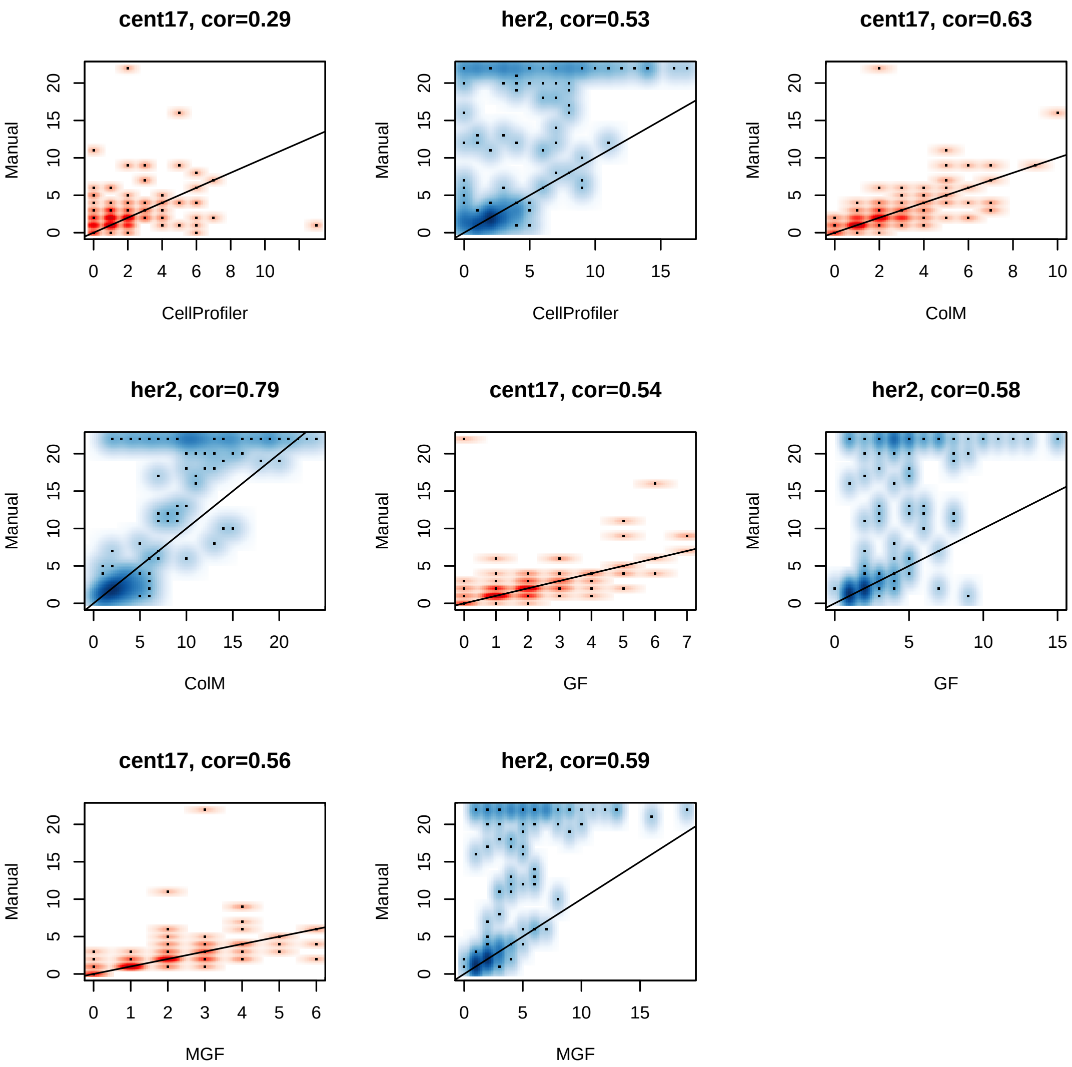


```r
## Perform correlation testing for CellProfiler and COlumbus first
Cent17CNCorr=sapply(1:10, function(x) cor.test(
  as.numeric(CP_CN[[x]][ ,"cent17_CN_spot"]), as.numeric(CP_CN[[x]][, "cent17.CN.true"])
  use="complete")[3:4])
ERBB2CNCorr=sapply(1:10, function(x) cor.test(
  as.numeric(CP_CN[[x]][ ,"ERBB2_CN_spot"]), as.numeric(CP_CN[[x]][, "ERBB2.CN.true"]),
  use="complete")[3:4])
Col_spot17=sapply(1:10, function(x) cor.test(
  as.numeric(Columbus_CN[[x]][ ,"cent17_CN_spot"]),
  as.numeric(Columbus_CN[[x]][, "cent17.CN.true"]), use="complete")[3:4])
Col_spotERB=sapply(1:10, function(x) cor.test(
  as.numeric(Columbus_CN[[x]][ ,"ERBB2_CN_spot"]),
```

```
  as.numeric(Columbus_CN[[x]][, "ERBB2.CN.true"]), use="complete")[3:4])
colnames(Cent17CNCorr)=names(CP_CN)
colnames(ERBB2CNCorr)=names(CP_CN)
## Plot the output here
par(mfrow=c(2,2))
CorTestImage(Cent17CNCorr, main="CP Cent17")
CorTestImage(ERBB2CNCorr, main="CP ERBB2")
CorTestImage(Col_spot17, main="Col Cent17")
CorTestImage(Col_spotERB, main="Col ERBB2")
```

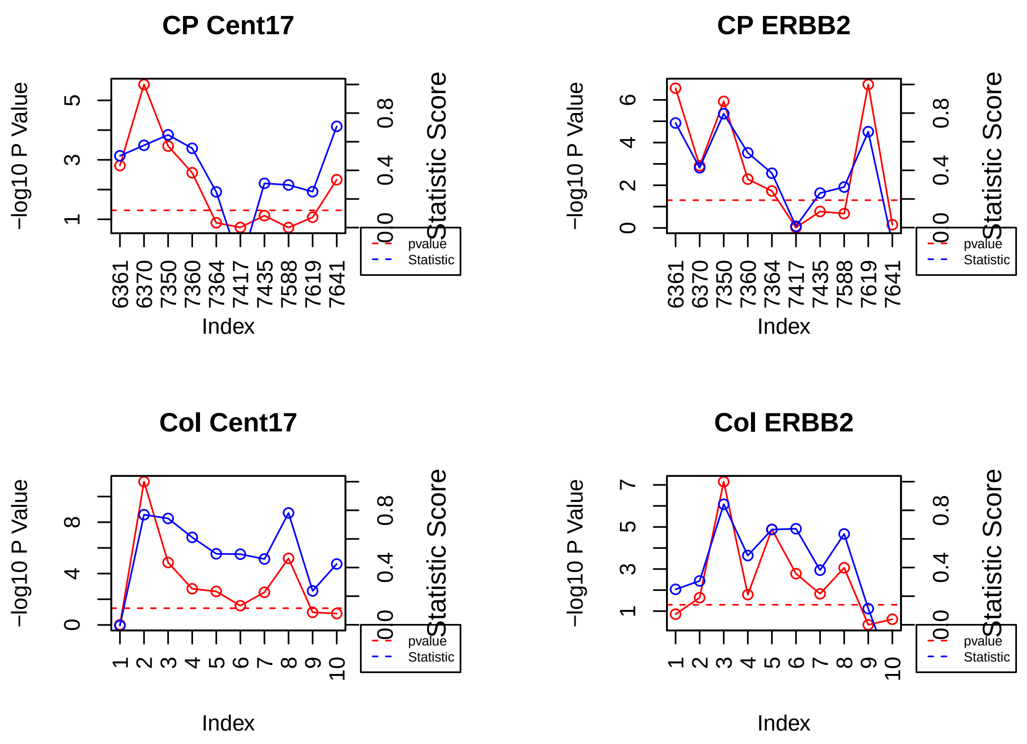


```
# Perform the same plots for GOIFISH
GF_spot17=sapply(1:10, function(x) cor.test(as.numeric(
  GF_CN[[x]][ ,"cent17_CN_spot"]), as.numeric(GF_CN[[x]][, "cent17.CN.true"]),
                                         use="complete")[3:4])
GF_spotERB=sapply(1:10, function(x) cor.test(
  as.numeric(GF_CN[[x]][ ,"ERBB2_CN_spot"]), as.numeric(GF_CN[[x]][, "ERBB2.CN.true"]),
  use="complete")[3:4])
MGF_spot17=sapply(1:10, function(x) cor.test(as.numeric(
  ManGF_CN[[x]][ ,"cent17_CN_spot"]), as.numeric(ManGF_CN[[x]][, "cent17.CN.true"]),
                                          use="complete")[3:4])
```

```r
MGF_spotERB=sapply(1:10, function(x) cor.test(as.numeric(
  ManGF_CN[[x]][ ,"ERBB2_CN_spot"]), as.numeric(ManGF_CN[[x]][, "ERBB2.CN.true"]),
                                              use="complete")[3:4])

## Plot the outpt here
CorTestImage(GF_spot17, main="GF_cent17")
CorTestImage(GF_spotERB, main="GF_ERB")
CorTestImage(MGF_spot17, main="MGF_cent17")
CorTestImage(MGF_spotERB, main="MGF_ERB")
```

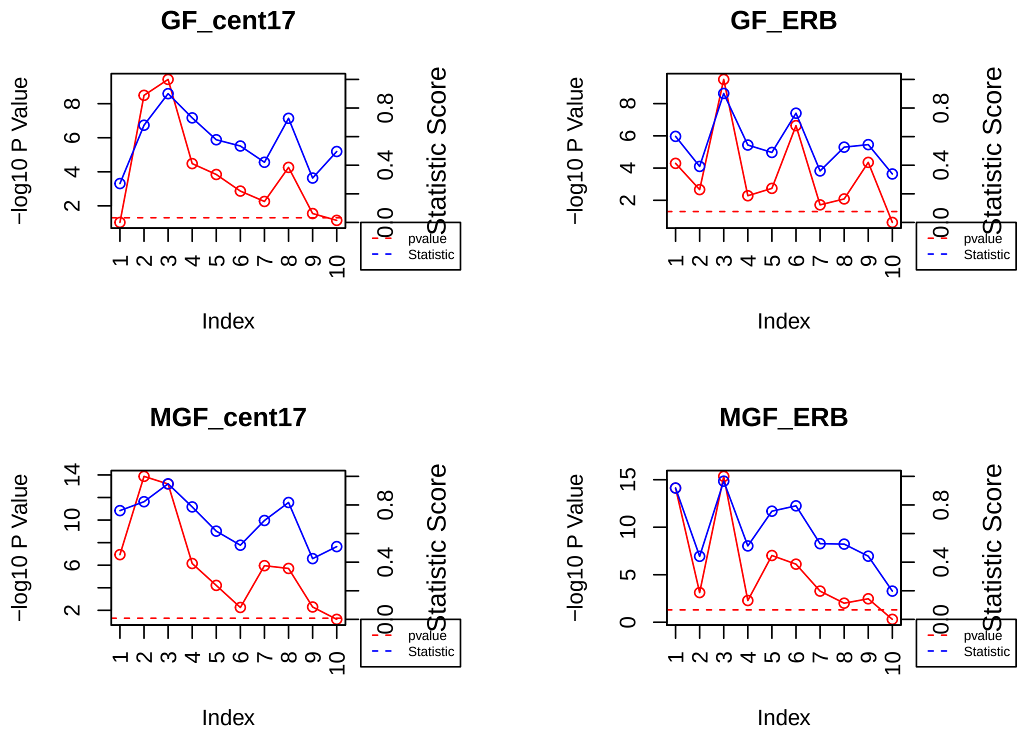

The above plots shows the -log10 p value (Red line) and correlation coefficient (blue lines). The red dotted line indicates a significant p value.

In the manual GoIFISH samples, 7641 is possibly the only case which does not reach a level of significance in comparisons between copy number for both cent17 and HER2. This may be due to the small number of cells present, and the high HER2 amplification seen where distinct spots are not obvious.

The default GoIFISH shows the same, with sample 6361 centromeric detection insignificant, possibly due to low signal and poor segmentation. Columbus suffers in samples 6361, 7419 and 7641. CellProfiler cent17 detection suffers in half its samples, and poor correlation was seen in 4 HER2 samples.

5.2 Direct Comparison to area

Next, we want to see whether the copy number is comparable to a measureable output. We first look at area:

```
## CellProfiler
CP_spot[ ,3]= unlist(lapply(c(1:8, 10), function(x) CP_CN[[x]]$cent17_Area))
CP_spot[ ,4]=unlist(lapply(c(1:8, 10), function(x) CP_CN[[x]]$ERBB2_Area))
## Columbus
Col_spot[ ,3]= unlist(lapply(c(1:8, 10), function(x) Columbus_CN[[x]]$cent17_Area))
Col_spot[ ,4]=unlist(lapply(c(1:8, 10), function(x) Columbus_CN[[x]]$ERBB2_Area))
## GoIFISH
GF_Spot[ ,3]=unlist(lapply(c(1:8, 10), function(x) GF_CN[[x]]$cent17_Area))
GF_Spot[ ,4]=unlist(lapply(c(1:8, 10), function(x) GF_CN[[x]]$ERBB2_Area))
## ManualGoIFISH
MGF_Spot[ ,3]=unlist(lapply(c(1:8, 10), function(x) ManGF_CN[[x]]$cent17_Area))
MGF_Spot[ ,4]=unlist(lapply(c(1:8, 10), function(x) ManGF_CN[[x]]$ERBB2_Area))
## STart making plots here
par(mfrow=c(3,3))

SmoothScatterCompare(CP_spot[ -CP_spot_idx,3], CP_spot[ -CP_spot_idx,1],
                     xlim=c(0, 800), "cent17", "CellProfiler")
SmoothScatterCompare(CP_spot[ -CP_spot_idx,4], CP_spot[ -CP_spot_idx,2],
                     xlim=c(0, 800), "her2", "CellProfiler")

SmoothScatterCompare(Col_spot[ -Col_spot_idx,3], Col_spot[ -Col_spot_idx,1],
                     xlim=c(0, 800), "cent17", "Columbus")
SmoothScatterCompare(Col_spot[ -Col_spot_idx,4], Col_spot[ -Col_spot_idx,2],
                     xlim=c(0, 800), "her2", "Columbus")

SmoothScatterCompare(GF_Spot[ -GF_spot_idx,3], GF_Spot[ -GF_spot_idx,1],
                     xlim=c(0, 800), "cent17", "GF")
SmoothScatterCompare(GF_Spot[ -GF_spot_idx,4], GF_Spot[ -GF_spot_idx,2],
                     xlim=c(0, 800), "her2", "GF")

SmoothScatterCompare(MGF_Spot[ -MGF_spot_idx,3], MGF_Spot[ -MGF_spot_idx,1],
                     xlim=c(0, 800), "cent17", "MGF")
SmoothScatterCompare(MGF_Spot[ -MGF_spot_idx,4], MGF_Spot[ -MGF_spot_idx,2],
                     xlim=c(0, 800), "her2", "MGF")
```

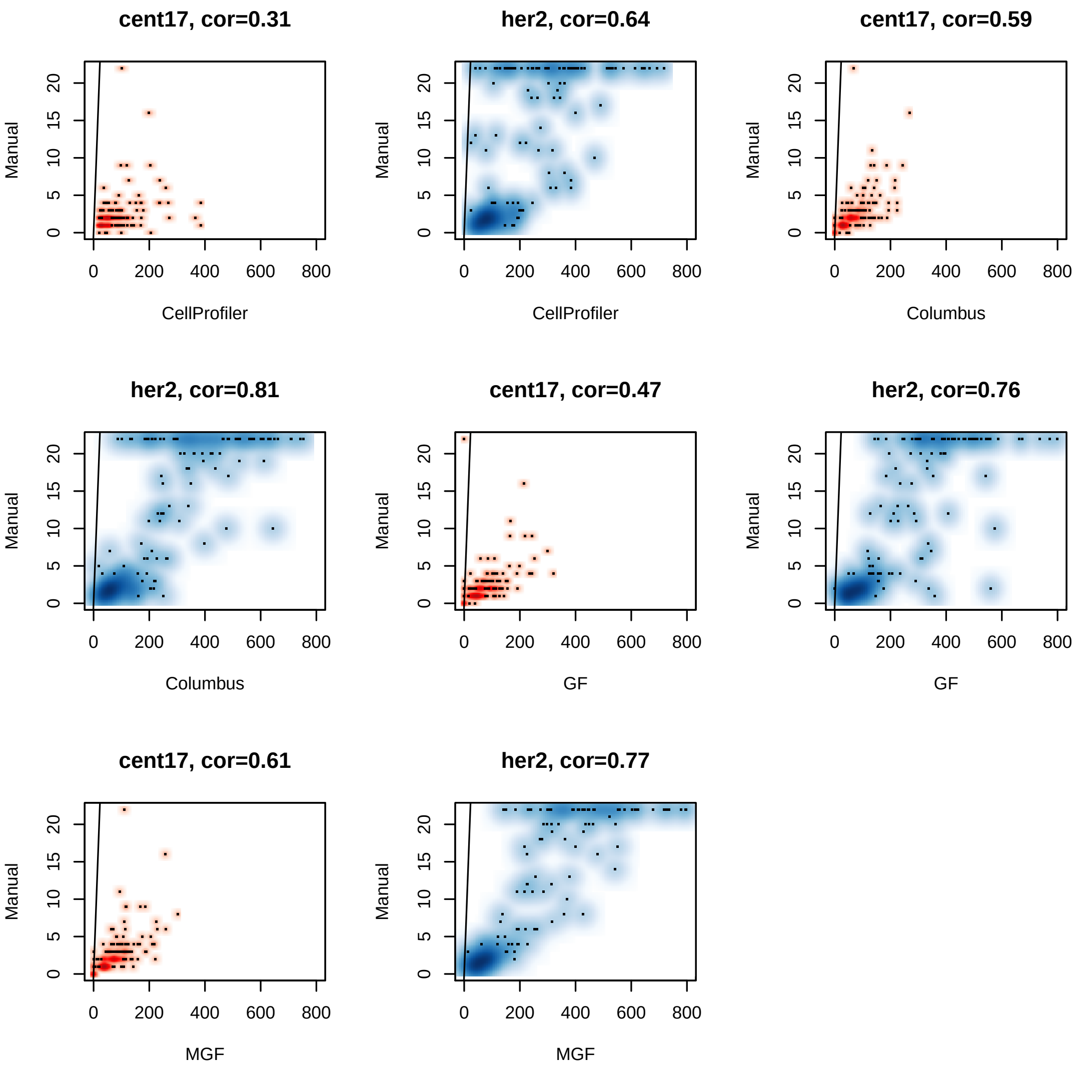

In most samples (except Cellprofiler), we see a good correlation between the spot count and the area. This plateaus out at approximately 400 pixels or 20 spots.

We want to see how these spots are distributed sample wise:

```
par(mfrow=c(1,2))
plot(NA, NA, xlim=c(0, 800), ylim=c(0, 22), ylab="Manual Count", xlab="Estimated Area",
for (i in c(1:8, 10)){
  rmIdx=which(ManGF_CN[[i]][,3]>0)
  points(ManGF_CN[[i]]$ERBB2_Area[-rmIdx], jitter(as.numeric(as.character(
    ManGF_CN[[i]]$ERBB2.CN2[-rmIdx])), amount=0.1), col=i, pch=19)
}

plot(NA, NA, xlim=c(0, 400), ylim=c(0, 10), ylab="Manual Count", xlab="Estimated Area",
```

```
for (i in c(1:8, 10)){
  rmIdx=which(ManGF_CN[[i]][,3]>0)
  points(ManGF_CN[[i]]$cent17_Area, jitter(as.numeric(as.character(
    ManGF_CN[[i]]$cent17.CN2)), amount=0.1), col=i, pch=19)
}

par(xpd=T)
legend(410, 10, c("6361", "6370", "7350", "7360", "7364","7417", "7435", "7588", "7641")
```

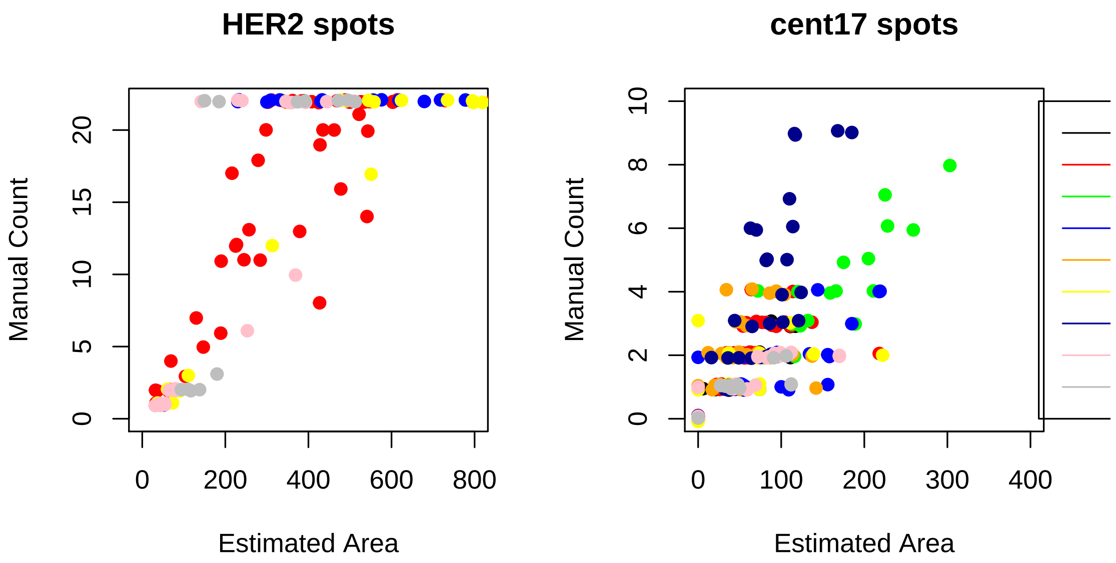

Most of the points do appear to overlap between samples. One exception is possibly the cent17 blue and green sample (7350 and 7435)

We can set a regression on each of the cent17 and HER2 samples to estimated the size of a spot (in pixels)

```
# look at cent17 first. CellProfiler
x1 = which(CP_spot[, 1] == 22)
summary(lm(CP_spot[-c(CP_spot_idx, x1), 3] ~ CP_spot[-c(CP_spot_idx, x1), 1]))

##
## Call:
## lm(formula = CP_spot[-c(CP_spot_idx, x1), 3] ~ CP_spot[-c(CP_spot_idx,
##     x1), 1])
##
## Residuals:
##    Min     1Q Median     3Q    Max
##  -94.2  -35.2  -18.9   15.8  323.3
```

```
##
## Coefficients:
##                                    Estimate Std. Error t value Pr(>|t|)
## (Intercept)                           47.95       7.27    6.59  4.9e-10 ***
## CP_spot[-c(CP_spot_idx, x1), 1]     13.71       2.55    5.38  2.3e-07 ***
## ---
## Signif. codes:  0 '***' 0.001 '**' 0.01 '*' 0.05 '.' 0.1 ' ' 1
##
## Residual standard error: 61.5 on 174 degrees of freedom
##   (102 observations deleted due to missingness)
## Multiple R-squared:  0.143,Adjusted R-squared:  0.138
## F-statistic:   29 on 1 and 174 DF,  p-value: 2.34e-07
```

```
# Columbus
x1 = which(Col_spot[, 1] == 22)
summary(lm(Col_spot[-c(Col_spot_idx, x1), 3] ~ Col_spot[-c(Col_spot_idx, x1),
    1]))
```

```
##
## Call:
## lm(formula = Col_spot[-c(Col_spot_idx, x1), 3] ~ Col_spot[-c(Col_spot_idx,
##     x1), 1])
##
## Residuals:
##    Min     1Q Median     3Q    Max
## -89.41 -18.72  -5.06  12.02 145.94
##
## Coefficients:
##                                      Estimate Std. Error t value Pr(>|t|)
## (Intercept)                             23.55       3.36    7.02  2.2e-11 ***
## Col_spot[-c(Col_spot_idx, x1), 1]     18.17       1.16   15.71  < 2e-16 ***
## ---
## Signif. codes:  0 '***' 0.001 '**' 0.01 '*' 0.05 '.' 0.1 ' ' 1
##
## Residual standard error: 34.7 on 245 degrees of freedom
##   (30 observations deleted due to missingness)
## Multiple R-squared:  0.502,Adjusted R-squared:   0.5
## F-statistic:  247 on 1 and 245 DF,  p-value: <2e-16
```

```
# GoIFISH
x1 = which(GF_Spot[, 1] == 22)
summary(lm(GF_Spot[-c(GF_spot_idx, x1), 3] ~ GF_Spot[-c(GF_spot_idx, x1), 1]))
```

```
##
```

```
## Call:
## lm(formula = GF_Spot[-c(GF_spot_idx, x1), 3] ~ GF_Spot[-c(GF_spot_idx,
##     x1), 1])
##
## Residuals:
##     Min      1Q  Median      3Q     Max
## -126.97  -24.24   -5.54   19.96  212.68
##
## Coefficients:
##                                    Estimate Std. Error t value Pr(>|t|)
## (Intercept)                           30.77       4.16     7.4  2.9e-12 ***
## GF_Spot[-c(GF_spot_idx, x1), 1]    19.39       1.52    12.8  < 2e-16 ***
## ---
## Signif. codes:  0 '***' 0.001 '**' 0.01 '*' 0.05 '.' 0.1 ' ' 1
##
## Residual standard error: 41.4 on 221 degrees of freedom
##   (2 observations deleted due to missingness)
## Multiple R-squared:  0.424,Adjusted R-squared:  0.422
## F-statistic:  163 on 1 and 221 DF,  p-value: <2e-16
```

```r
# Manual GoIFISH
x1 = which(MGF_Spot[, 1] == 22)
summary(lm(MGF_Spot[-c(MGF_spot_idx, x1), 3] ~ MGF_Spot[-c(MGF_spot_idx, x1),
    1]))
```

```
##
## Call:
## lm(formula = MGF_Spot[-c(MGF_spot_idx, x1), 3] ~ MGF_Spot[-c(MGF_spot_idx,
##     x1), 1])
##
## Residuals:
##     Min      1Q  Median      3Q     Max
## -141.98  -24.86   -5.11   12.64  156.90
##
## Coefficients:
##                                      Estimate Std. Error t value Pr(>|t|)
## (Intercept)                             27.13       3.42    7.92  6.1e-14 ***
## MGF_Spot[-c(MGF_spot_idx, x1), 1]    18.99       1.21   15.70  < 2e-16 ***
## ---
## Signif. codes:  0 '***' 0.001 '**' 0.01 '*' 0.05 '.' 0.1 ' ' 1
##
## Residual standard error: 37.3 on 272 degrees of freedom
##   (21 observations deleted due to missingness)
## Multiple R-squared:  0.475,Adjusted R-squared:  0.474
```

```
## F-statistic:  247 on 1 and 272 DF,  p-value: <2e-16
```

We see that the coefficient for Columbus, GoIFISH and the manually curated GoIFISH samples are similar, with one spot designated by an area of approximately 17-20 pixels. A CellProfiler spot is 13.7 pixels, a Columbus spot is 18.2, GoIFISH is 19.4, GoIFISH manually curated is 19.

We can perform the sample with the ERBB2 dataset, omitting amplified cells to ensure linearity of the prediction: both Columbus and the Manually Curated GoIFISH estimate a HER2 spot at approximately the same size (Columbus: 18.9, GOIFISH: 19.5). The automated GoIFISH results predict this value to be around 15 pixels. CellProfiler is 12.6 pixels, and GoIFISH is 11.54.

```
# CellPorfiler
x1 = which(CP_spot[, 2] == 22)
summary(lm(CP_spot[-c(CP_spot_idx, x1), 4] ~ CP_spot[-c(CP_spot_idx, x1), 2]))

##
## Call:
## lm(formula = CP_spot[-c(CP_spot_idx, x1), 4] ~ CP_spot[-c(CP_spot_idx,
##     x1), 2])
##
## Residuals:
##    Min      1Q Median      3Q     Max
## -228.9  -40.1  -13.0   32.2  260.4
##
## Coefficients:
##                                  Estimate Std. Error t value Pr(>|t|)
## (Intercept)                         82.18       8.54    9.62   <2e-16 ***
## CP_spot[-c(CP_spot_idx, x1), 2]     12.64       1.23   10.30   <2e-16 ***
## ---
## Signif. codes:  0 '***' 0.001 '**' 0.01 '*' 0.05 '.' 0.1 ' ' 1
##
## Residual standard error: 75 on 130 degrees of freedom
##   (61 observations deleted due to missingness)
## Multiple R-squared:  0.449,Adjusted R-squared:  0.445
## F-statistic:  106 on 1 and 130 DF,  p-value: <2e-16

# Columbus
x1 = which(Col_spot[, 2] == 22)
summary(lm(Col_spot[-c(Col_spot_idx, x1), 4] ~ Col_spot[-c(Col_spot_idx, x1),
    2]))

##
## Call:
```

```
## lm(formula = Col_spot[-c(Col_spot_idx, x1), 4] ~ Col_spot[-c(Col_spot_idx,
##     x1), 2])
##
## Residuals:
##    Min     1Q Median     3Q    Max
## -129.5  -37.8  -18.7   25.6  402.9
##
## Coefficients:
##                                  Estimate Std. Error t value Pr(>|t|)
## (Intercept)                        51.994      6.921    7.51  3.6e-12 ***
## Col_spot[-c(Col_spot_idx, x1), 2]   18.915      0.943   20.05  < 2e-16 ***
## ---
## Signif. codes:  0 '***' 0.001 '**' 0.01 '*' 0.05 '.' 0.1 ' ' 1
##
## Residual standard error: 67.7 on 163 degrees of freedom
##   (24 observations deleted due to missingness)
## Multiple R-squared:  0.712,Adjusted R-squared:  0.71
## F-statistic:  402 on 1 and 163 DF,  p-value: <2e-16
```

```
# GoIFISH
x1 = which(GF_Spot[, 2] == 22)
summary(lm(GF_Spot[-c(GF_spot_idx, x1), 4] ~ GF_Spot[-c(GF_spot_idx, x1), 2]))
```

```
##
## Call:
## lm(formula = GF_Spot[-c(GF_spot_idx, x1), 4] ~ GF_Spot[-c(GF_spot_idx,
##     x1), 2])
##
## Residuals:
##    Min     1Q Median     3Q    Max
## -168.2  -39.2  -14.1   14.4  463.0
##
## Coefficients:
##                                Estimate Std. Error t value Pr(>|t|)
## (Intercept)                       67.42       8.34    8.09  1.7e-13 ***
## GF_Spot[-c(GF_spot_idx, x1), 2]    14.79       1.19   12.42  < 2e-16 ***
## ---
## Signif. codes:  0 '***' 0.001 '**' 0.01 '*' 0.05 '.' 0.1 ' ' 1
##
## Residual standard error: 80 on 154 degrees of freedom
##   (1 observation deleted due to missingness)
## Multiple R-squared:   0.5,Adjusted R-squared:  0.497
## F-statistic:  154 on 1 and 154 DF,  p-value: <2e-16
```

```r
# Manual GoIFISH
x1 = which(MGF_Spot[, 2] == 22)
summary(lm(MGF_Spot[-c(MGF_spot_idx, x1), 4] ~ MGF_Spot[-c(MGF_spot_idx, x1),
    2]))

##
## Call:
## lm(formula = MGF_Spot[-c(MGF_spot_idx, x1), 4] ~ MGF_Spot[-c(MGF_spot_idx,
##     x1), 2])
##
## Residuals:
##    Min      1Q Median      3Q     Max
## -153.5  -26.8   -7.2   19.2  233.4
##
## Coefficients:
##                                   Estimate Std. Error t value Pr(>|t|)
## (Intercept)                         37.217      5.458    6.82  1.3e-10 ***
## MGF_Spot[-c(MGF_spot_idx, x1), 2]   19.544      0.763   25.61  < 2e-16 ***
## ---
## Signif. codes:  0 '***' 0.001 '**' 0.01 '*' 0.05 '.' 0.1 ' ' 1
##
## Residual standard error: 57.1 on 183 degrees of freedom
##   (20 observations deleted due to missingness)
## Multiple R-squared:  0.782,Adjusted R-squared:  0.781
## F-statistic:  656 on 1 and 183 DF,  p-value: <2e-16
```

5.3 Combining Area and Intensity

The last thing we want to check is whether the correlation improve if we add intensity information to area.

We perform a similar correlation testing to that shown in the previous section.

```r
## Calculate Area and Intensity
CP_CN = VarCombine(CP_CN, "cent17_Area", "cent17_Int", "cent17_ArInt")
CP_CN = VarCombine(CP_CN, "ERBB2_Area", "ERBB2_Int", "ERBB2_ArInt")
Columbus_CN = VarCombine(Columbus_CN, "cent17_Area", "cent17_Int", "cent17_ArInt")
Columbus_CN = VarCombine(Columbus_CN, "ERBB2_Area", "ERBB2_Int", "ERBB2_ArInt")
GF_CN = VarCombine(GF_CN, "cent17_Area", "cent17_backAdj", "cent17_ArInt")
GF_CN = VarCombine(GF_CN, "ERBB2_Area", "ERBB2_backAdj", "ERBB2_ArInt")
ManGF_CN = VarCombine(ManGF_CN, "cent17_Area", "cent17_backAdj", "cent17_ArInt")
ManGF_CN = VarCombine(ManGF_CN, "ERBB2_Area", "ERBB2_backAdj", "ERBB2_ArInt")

# Perform the categorisation CellProfiler
```

```r
CP_spot[, 3] = unlist(lapply(c(1:8, 10), function(x) CP_CN[[x]]$cent17_ArInt))
CP_spot[, 4] = unlist(lapply(c(1:8, 10), function(x) CP_CN[[x]]$ERBB2_ArInt))
## Columbus
Col_spot[, 3] = unlist(lapply(c(1:8, 10), function(x) Columbus_CN[[x]]$cent17_ArInt))
Col_spot[, 4] = unlist(lapply(c(1:8, 10), function(x) Columbus_CN[[x]]$ERBB2_ArInt))
## GoIFISH
GF_Spot[, 3] = unlist(lapply(c(1:8, 10), function(x) GF_CN[[x]]$cent17_ArInt))
GF_Spot[, 4] = unlist(lapply(c(1:8, 10), function(x) GF_CN[[x]]$ERBB2_ArInt))
## ManualGoIFISH
MGF_Spot[, 3] = unlist(lapply(c(1:8, 10), function(x) ManGF_CN[[x]]$cent17_ArInt))
MGF_Spot[, 4] = unlist(lapply(c(1:8, 10), function(x) ManGF_CN[[x]]$ERBB2_ArInt))
## STart making plots here
par(mfrow = c(3, 3))
SmoothScatterCompare(CP_spot[-CP_spot_idx, 3], CP_spot[-CP_spot_idx, 1], "cent17",
    "CellProfiler")

## Warning:  Binning grid too coarse for current (small) bandwidth:  consider
increasing 'gridsize'

SmoothScatterCompare(CP_spot[-CP_spot_idx, 4], CP_spot[-CP_spot_idx, 2], "her2",
    "CellProfiler")

SmoothScatterCompare(Col_spot[-Col_spot_idx, 3], Col_spot[-Col_spot_idx, 1],
    "cent17", "Columbus")
SmoothScatterCompare(Col_spot[-Col_spot_idx, 4], Col_spot[-Col_spot_idx, 2],
    "her2", "Columbus")

SmoothScatterCompare(GF_Spot[-GF_spot_idx, 3], GF_Spot[-GF_spot_idx, 1], "cent17",
    "GF")
SmoothScatterCompare(GF_Spot[-GF_spot_idx, 4], GF_Spot[-GF_spot_idx, 2], "her2",
    "GF")

SmoothScatterCompare(MGF_Spot[-MGF_spot_idx, 3], MGF_Spot[-MGF_spot_idx, 1],
    "cent17", "GF")
SmoothScatterCompare(MGF_Spot[-MGF_spot_idx, 4], MGF_Spot[-MGF_spot_idx, 2],
    "her2", "GF")
```

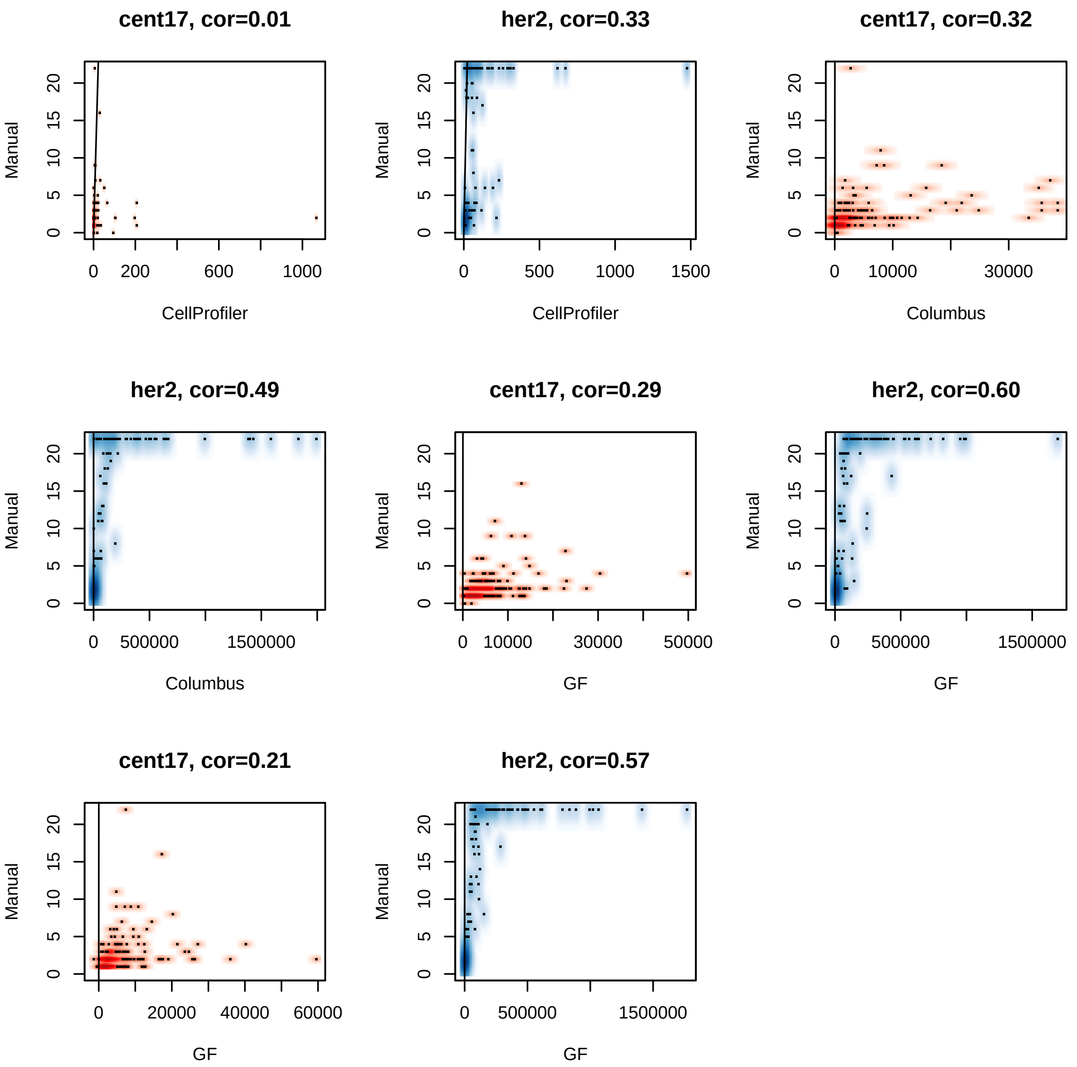

We see that in the cent17 samples, intensity information makes the correlation worse. However, HER2 detection may have a benefit in this added information

6 Inferring Sample Diversity

Now that we have attained several measurements about a sample, we would like to know how many possible clones there may be. We can start off doing this in a simple manner by thresholding based on ER2 and HER2 intensity (from Figure 2): We can set an arbitrary threshold at 50 which includes most negative samples, and diffeentiates against high and moderate. Similarly, we can set this value at around 250 for HER2 background Adjusted Intensity.

6.1 ER and HER2

```
# Cut the samples into pos and neg for each case
ManGF_ERcut=sapply(1:10, function(x) cut(ManGF_Protein[[x]]$ERNucInt, breaks=c(-500, 50,
ManGF_HER2cut=sapply(1:10, function(x) cut(ManGF_Protein[[x]]$HER2backAdj, breaks=c(-1000
# Create a series of bar plots or something to show the number of samples
Sum_ManGF=sapply(1:10, function(x) length(which(
  ManGF_HER2cut[[x]]=="neg" & ManGF_ERcut[[x]]=="neg")))
Sum_ManGF=cbind(Sum_ManGF, sapply(1:10, function(x) length(which(
  ManGF_HER2cut[[x]]=="pos" & ManGF_ERcut[[x]]=="neg"))))
Sum_ManGF=cbind(Sum_ManGF, sapply(1:10, function(x) length(which(
  ManGF_HER2cut[[x]]=="neg" & ManGF_ERcut[[x]]=="pos"))))
Sum_ManGF=cbind(Sum_ManGF, sapply(1:10, function(x) length(
  which(ManGF_HER2cut[[x]]=="pos" & ManGF_ERcut[[x]]=="pos"))))
t1=rowSums(Sum_ManGF)
Sum_ManGF=Sum_ManGF/t1
par(oma=c(1,1.2,1.3,4.1), xpd=F)
barplot(t(Sum_ManGF), col=c("lightpink", "skyblue", "magenta", "darkblue"),
        names=sprintf("%s (%g)",names(ManGF_Protein),t1 ), las=2)
par(xpd=T)
legend(11,1, c("Neg", "HER2pos", "ERpos", "DoublePos"), lwd=2,
       col=c("lightpink", "skyblue", "magenta", "darkblue"))
```

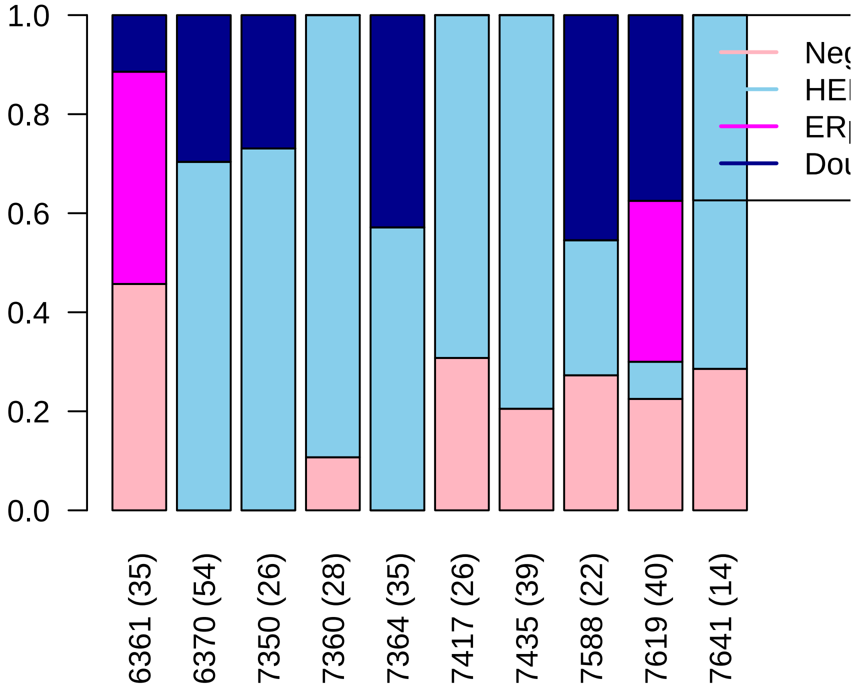

We see here that 4 samples are only HER2 positive. All other samples appear to have some positivity in ER. Only 6361 and 7641 appear to have ER positive cells which are not also HER2 positive.

```
# Test the remainder of the population which has yet to be tested
ManGF_ERcut=sapply(1:10, function(x) cut(ManGF_Prot_notscored[[x]]$ERNucInt,
                    breaks=c(-500, 50, 1000), labels=c("neg", "pos")))
ManGF_HER2cut=sapply(1:10, function(x) cut(ManGF_Prot_notscored[[x]]$HER2backAdj,
                    breaks=c(-1000, 300, 5000), labels=c("neg", "pos")))
# Create a series of bar plots or something to show the number of samples
Sum_ManGF=sapply(1:10, function(x) length(which(
  ManGF_HER2cut[[x]]=="neg" & ManGF_ERcut[[x]]=="neg")))
Sum_ManGF=cbind(Sum_ManGF, sapply(1:10, function(x) length(
  which(ManGF_HER2cut[[x]]=="pos" & ManGF_ERcut[[x]]=="neg"))))
Sum_ManGF=cbind(Sum_ManGF, sapply(1:10, function(x) length(
  which(ManGF_HER2cut[[x]]=="neg" & ManGF_ERcut[[x]]=="pos"))))
Sum_ManGF=cbind(Sum_ManGF, sapply(1:10, function(x) length(
  which(ManGF_HER2cut[[x]]=="pos" & ManGF_ERcut[[x]]=="pos"))))
```

```
t1=rowSums(Sum_ManGF)
Sum_ManGF=Sum_ManGF/t1
par(oma=c(1,1.2,1.3,4.1), xpd=F)
barplot(t(Sum_ManGF), col=c("lightpink", "skyblue", "magenta", "darkblue"),
        names=sprintf("%s (%g)",names(ManGF_Prot_notscored),t1 ), las=2)
par(xpd=T)
legend(11,1, c("Neg", "HER2pos", "ERpos", "DoublePos"), lwd=2,
       col=c("lightpink", "skyblue", "magenta", "darkblue"))
```

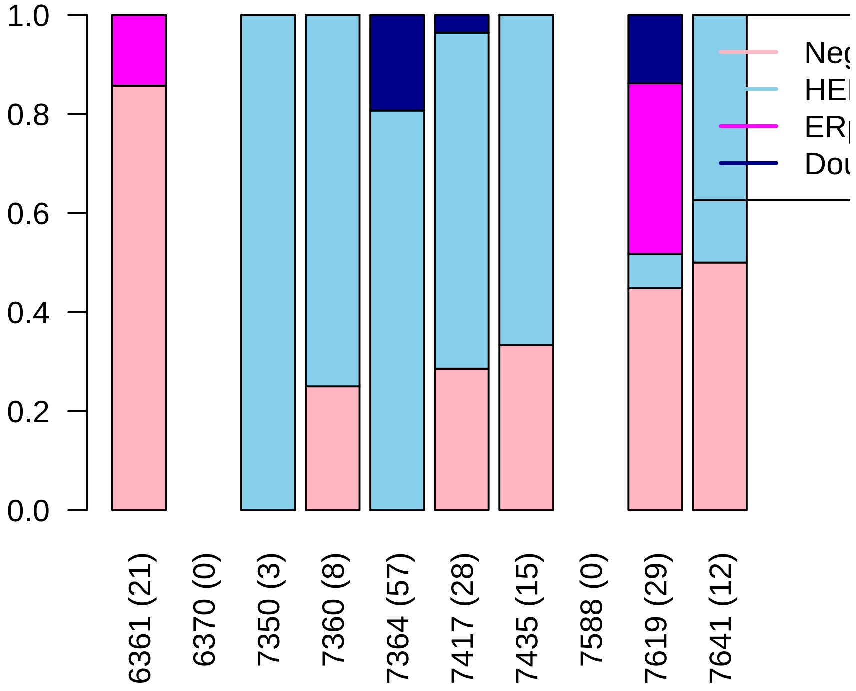


```
# We should check with the manual samples
T_ManGF=sapply(1:10, function(x) length(which(ManGF_Protein[[x]][,"ER.score"]=="-ve" & Ma
T_ManGF=cbind(T_ManGF, sapply(1:10, function(x) length(which(ManGF_Protein[[x]][,"ER.scor
T_ManGF=cbind(T_ManGF, sapply(1:10, function(x) length(which(ManGF_Protein[[x]][,"ER.scor
T_ManGF=cbind(T_ManGF, sapply(1:10, function(x) length(which(ManGF_Protein[[x]][,"ER.scor
t1=rowSums(T_ManGF)
T_ManGF=T_ManGF/t1
par(oma=c(1,1.2,1.3,4.1), xpd=F)
```

```
barplot(t(T_ManGF), col=c("lightpink", "skyblue", "magenta", "darkblue"),
        names=sprintf("%s (%g)",names(ManGF_Protein),t1 ), las=2)
par(xpd=T)
legend(11,1, c("Neg", "HER2pos", "ERpos", "DoublePos"), lwd=2,
       col=c("lightpink", "skyblue", "magenta", "darkblue"))
```

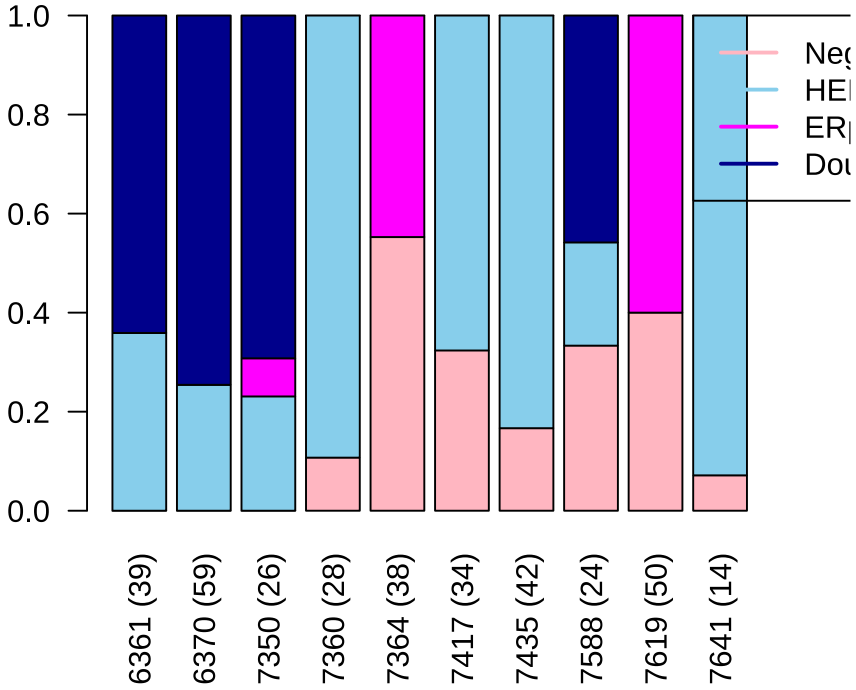

6.2 HER2 copy number vs protein

We can also do a similar approach with HER2 protein and copy number:

```
# Cut the samples into pos and neg for each case
ManGF_HER2cut=sapply(1:10, function(x) cut(ManGF_Protein[[x]]$HER2backAdj,
                breaks=c(-1000, 300, 5000), labels=c("neg", "pos")))
ManGF_spot=sapply(1:10, function(x) cut(ManGF_CN[[x]]$ERBB2_Area,
                breaks=c(-500, 60, 1000), labels=c("neg", "pos")))
class(ManGF_CN[[2]]$ERBB2_Area)

## [1] "integer"
```

```
class(ManGF_CN[[3]]$ERBB2_Area)

## [1] "integer"

# Create a series of bar plots or something to show the number of samples
Sum_ManGF=sapply(1:10, function(x) length(which(
  ManGF_HER2cut[[x]]=="neg" & ManGF_spot[[x]]=="neg")))
Sum_ManGF=cbind(Sum_ManGF, sapply(1:10, function(x) length(
  which(ManGF_HER2cut[[x]]=="pos" & ManGF_spot[[x]]=="neg"))))
Sum_ManGF=cbind(Sum_ManGF, sapply(1:10, function(x) length(
  which(ManGF_HER2cut[[x]]=="neg" & ManGF_spot[[x]]=="pos"))))
Sum_ManGF=cbind(Sum_ManGF, sapply(1:10, function(x) length(
  which(ManGF_HER2cut[[x]]=="pos" & ManGF_spot[[x]]=="pos"))))
t1=rowSums(Sum_ManGF)
Sum_ManGF=Sum_ManGF/t1
par(oma=c(1,1.2,1.3,4.1), xpd=F)
barplot(t(Sum_ManGF), col=c("lightpink", "skyblue", "lightgreen", "darkgreen"),
        names=sprintf("%s (%g)",names(ManGF_Protein),t1 ), las=2)
par(xpd=T)
legend(11,1, c("Neg", "HER2pos", "ERpos", "DoublePos"), lwd=2,
       col=c("lightpink", "skyblue", "lightgreen", "darkgreen"))
```

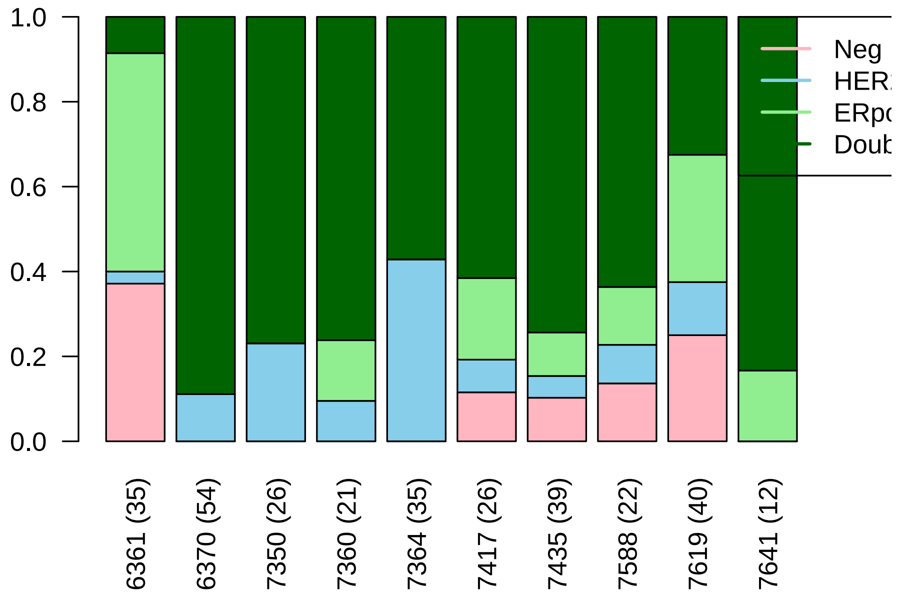

```r
# True Plot
T_ManGF=sapply(1:10, function(x) length(which(
  ManGF_CN[[x]][,"ERBB2.CN2"]<3 & ManGF_Protein[[x]][, "HER2.prot.score"]=="-ve")))
T_ManGF=cbind(T_ManGF, sapply(1:10, function(x) length(which(
  ManGF_CN[[x]][,"ERBB2.CN2"]<3 & ManGF_Protein[[x]][, "HER2.prot.score"]=="+ve"))))
T_ManGF=cbind(T_ManGF, sapply(1:10, function(x) length(which(
  ManGF_CN[[x]][,"ERBB2.CN2"]>2 & ManGF_Protein[[x]][, "HER2.prot.score"]=="-ve"))))
T_ManGF=cbind(T_ManGF, sapply(1:10, function(x) length(which(
  ManGF_CN[[x]][,"ERBB2.CN2"]>2 & ManGF_Protein[[x]][, "HER2.prot.score"]=="+ve"))))
t1=rowSums(T_ManGF)
T_ManGF=T_ManGF/t1
par(oma=c(1,1.2,1.3,4.1), xpd=F)
barplot(t(T_ManGF), col=c("lightpink", "skyblue", "lightgreen", "darkgreen"),
        names=sprintf("%s (%g)",names(ManGF_Protein),t1 ), las=2)
par(xpd=T)
legend(11,1, c("Neg", "HER2pos", "ERpos", "DoublePos"), lwd=2,
       col=c("lightpink", "skyblue", "lightgreen", "darkgreen"))
```

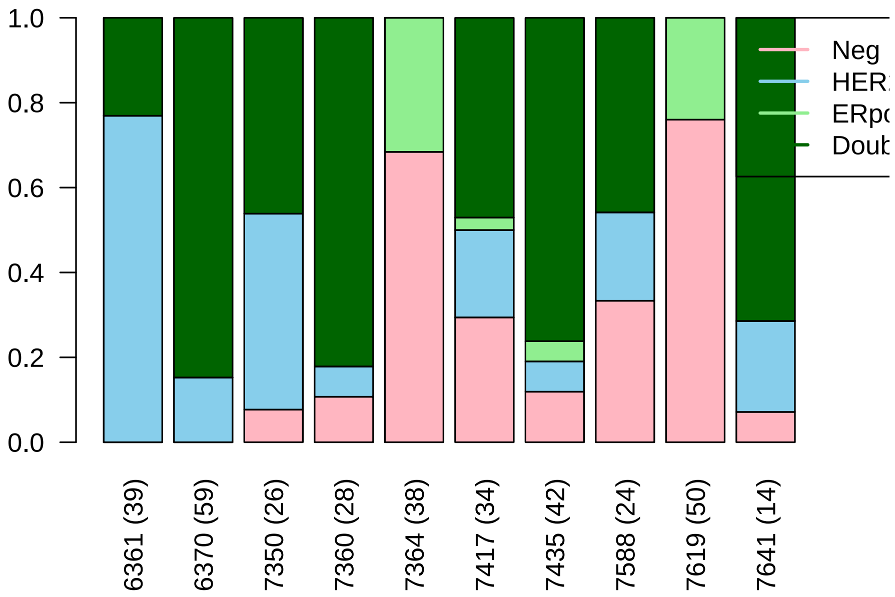

We can also see how copy number relates to spot number (now that we have shown a correlation between the two). We can show show samples which may have a linear relationship with the HER2 protein intensity, and two samples which seem randomly

distributed:

```
palette(c("Black","Red","Green", "Blue", "orange",  "yellow", "DarkBlue", "Pink","Cyan",
CN_adjusted=sapply(1:10, function(x) ManGF_CN[[x]]$ERBB2_Area)
plot(NA, NA, xlab="ERBB2Area", ylab="HER2 intensity",
     xlim=c(0, 800), ylim=c(0, 2500), main="Increasing Trend")
t1=rep(NA, 10)
for (i in c( 6, 8)){
points(CN_adjusted[[i]], ManGF_Protein[[i]]$HER2backAdj, col=(i-1), pch=19)
t1[i]=cor(CN_adjusted[[i]], ManGF_Protein[[i]]$HER2backAdj, use="complete")
}
legend(600, 2500, names(ManGF_CN)[c(6,8)], col=c(5,7), lwd=1)
```

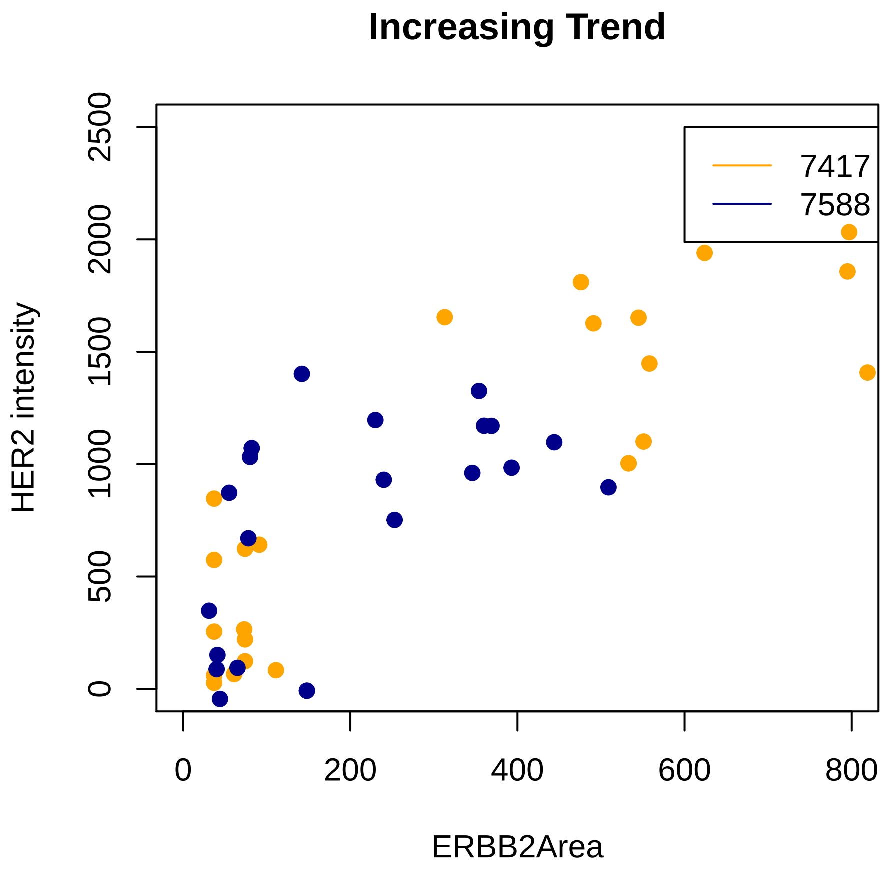


```
plot(NA, NA, xlab="ERBB2Area", ylab="HER2 intensity",
     xlim=c(0, 800), ylim=c(0, 2500), main="Random Distribution")
t1=rep(NA, 10)
```

```
for (i in c( 2, 4)){
points(CN_adjusted[[i]], ManGF_Protein[[i]]$HER2backAdj, col=i, pch=19)
t1[i]=cor(CN_adjusted[[i]], ManGF_Protein[[i]]$HER2backAdj, use="complete")
}
legend(600, 2500, names(ManGF_CN)[c(2,4)], col=c(2,4), lwd=1)
```

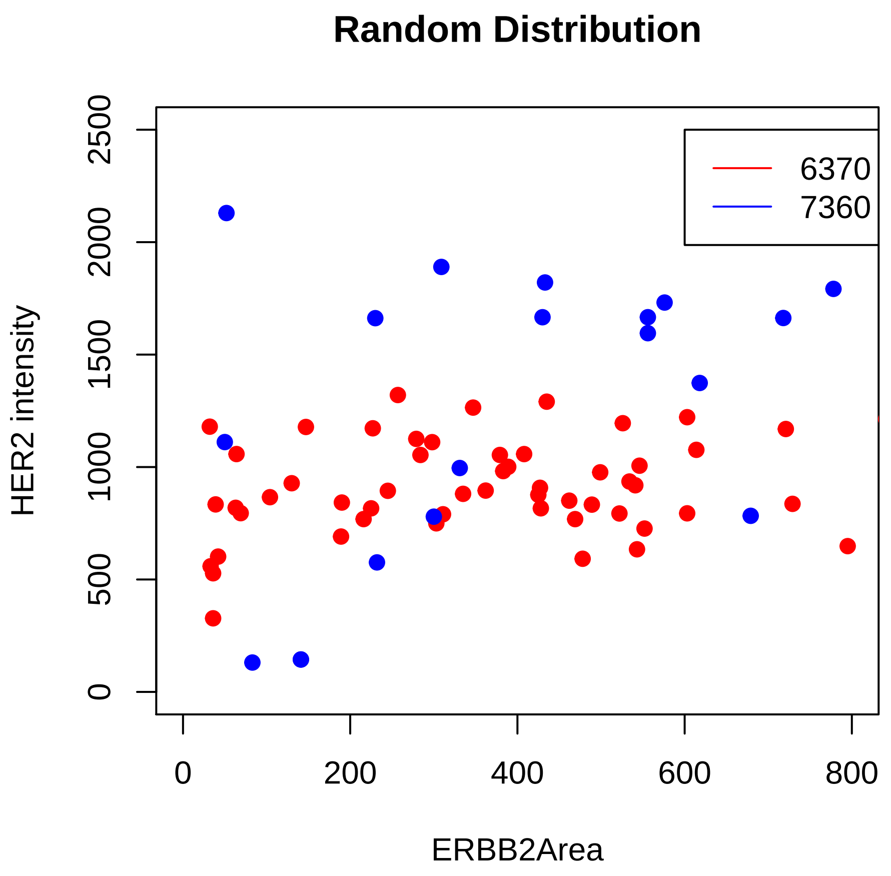


```
names(t1)=names(ManGF_CN)
t1

##    6361    6370    7350    7360    7364    7417    7435    7588    7619    7641
##      NA  0.2218      NA  0.2909      NA      NA      NA      NA      NA      NA
```

Plotting the ratio of ERBB2 to cent17 and plotting against the HER2 intensity shows some interesting trends: For a lot of samples, HER2 expression appears to be independent of the normalised copy number. One exception is sample yellow (7417) which has a somewhat linear behaviour. In contrast, sample 7360 appears to show no link to

copynumber. Sample 7588 has variable copy number status but no increase in HER2 expression

7 Software Performance

7.1 Classification Performance

Here, we assess the prediction accuracy of the SVM used to predict cell labels. In short, 2 test images have been labelled by a pathologist. We load in this data, and select a training-set at random. This training-set will encompass at least 1 cell of each label, and will be used to predict the labels of the rest of the cells.

The number of cells in the training set will grow in number and we will assess the minimum number of cells required to achieve high classificant accuracy. In order to determine the confidence intervals, the training-set will be permuted 500 times for a given set size.

Note this may be a time-intensive process. We have cached the output as an .RData object, which can be reproduced using the following set.seed(15):

7.1.1 Differentiating between luminal and epithelial cells

```
# Sample Number 6361: myoepithelial vs luminal cells load data.
Dat6361 = read.table("data/CellClassification/6361.txt", header = T)
Dat6361.Morph = Dat6361[, 2:7]
yLabel = Dat6361$Labels
summary(factor(yLabel))

##  1  2
## 39 17

# Perform Accuracy Testing on Morphological data only Example SVM
Lab1 = which(yLabel == 1)
Lab2 = which(yLabel == 2)
set.seed(15)
TestSamp = c(sample(Lab1, 1), sample(Lab2, 1))
TestLab = c(1, 2)
TestSet = Dat6361.Morph[TestSamp, ]
model = svm(TestSet, TestLab, kernel = "linear", type = "C-classification")
output = predict(model, Dat6361.Morph)
Accuracy = table(yLabel, output)
```

Given the labels for this data, we expect a minimum of 0.6964 accuracy (assuming that all samples are labelled as the dominant cell type). Using 2 cells as training data an accuracy of 0.7143 is achieved.

We now repeat, permuting the training set multiple times and including more data in the training set:

```
Out1=TestClassifier(Dat6361.Morph, yLabel, 500)

ggplot(Out1, aes(x=N, y=MeanVal)) + ylim(0.2, 1) +
    geom_errorbar(aes(ymin=CI.025, ymax=CI.975), width=.1) +
    geom_line() +
    geom_point()+
    theme_set(theme_bw(18))+
    ggtitle("Accuracy of classifier with variable size of trainings set")+
    xlab("Training Set Size") +
    ylab("Accuracy")
```

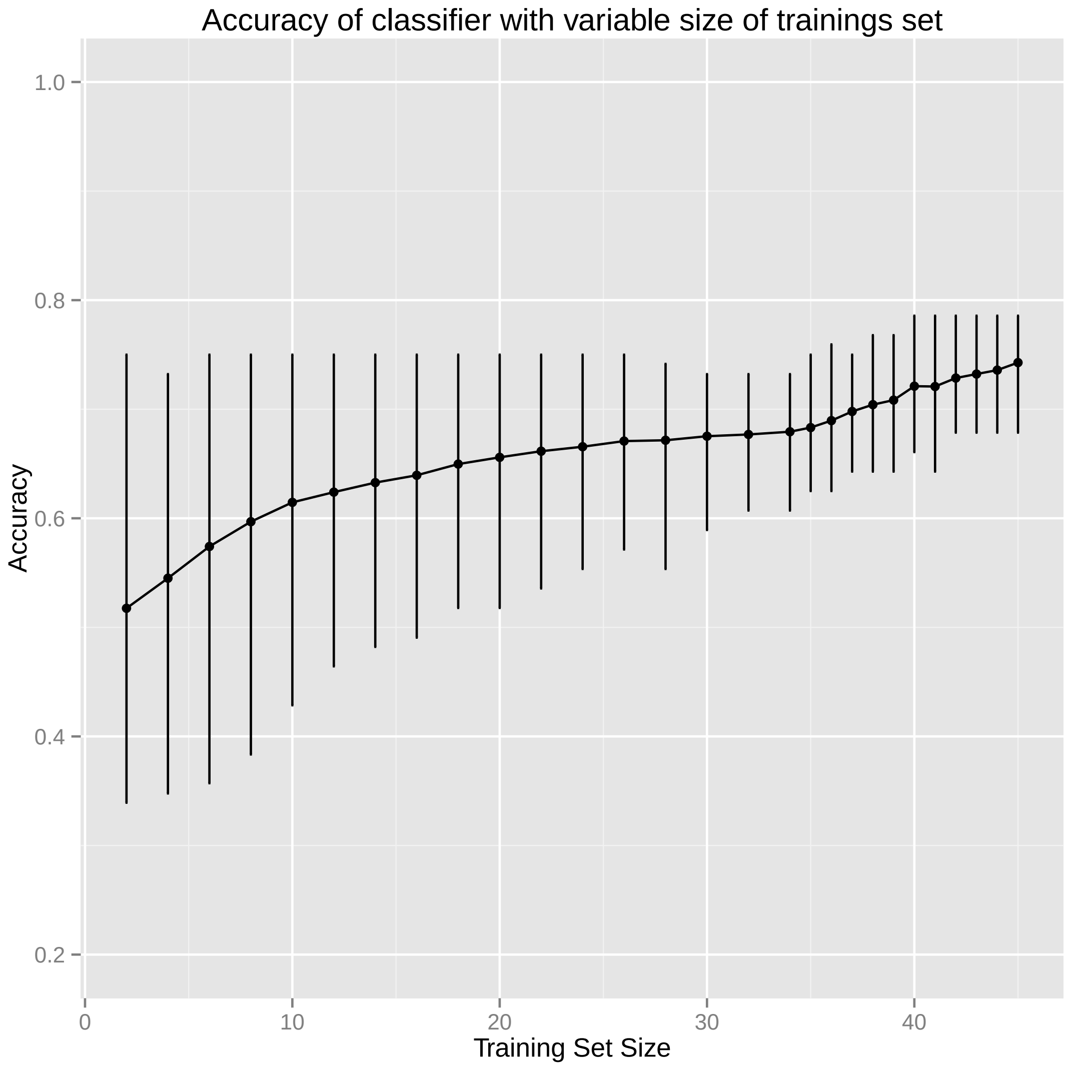

As expected, the mean classifier accuracy increases with larger training size, however, this value is capped at approximately 70% which is similar to random selection. The upper confidence interval limit (approximately 0.7) stays constant throughout the analysis. Thus, as long as two cells with morphology characteristic of the groups of interest are selected, high classification accuracy can still be attained.

We can also test whether including intensity information from the other channels of interest can improve this classification:

```
Dat6361.WInt=Dat6361[ ,-c(1, grep("Location", colnames(Dat6361)),31)]
Dat6361.WInt[is.na(Dat6361.WInt)]=0
Out2=TestClassifier(Dat6361.WInt, yLabel, 500)
ggplot(Out2, aes(x=N, y=MeanVal)) + ylim(0.2, 1)+
    geom_errorbar(aes(ymin=CI.025, ymax=CI.975), width=.1) +
```

```
    geom_line() +
    geom_point()+
    ggtitle("Accuracy of classifier with Stain Information")+
    xlab("Training Set Size") +
    ylab("Accuracy")
```

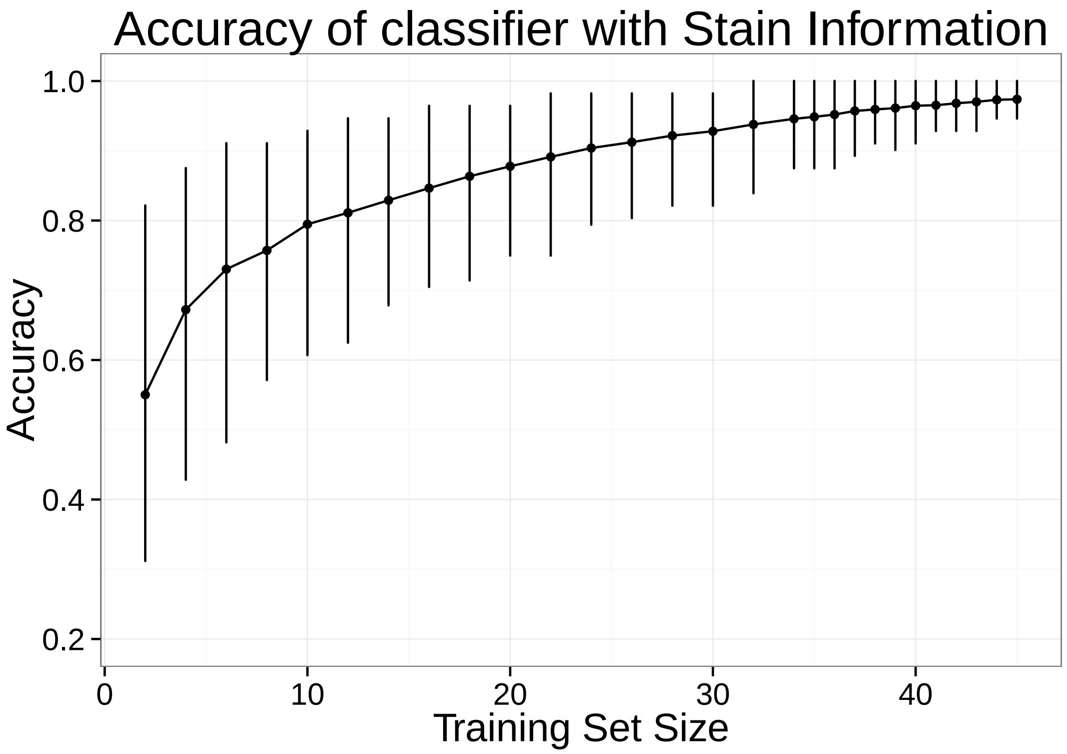

Including the stain information from other channels, we improve the average classification accuracy to over 90% when 22 cells or more are selected. If the training set is well selected, a minimum of 6 cells can give an accuracy of 90%. Note that warnings appear as in the training set, some cells may have have no detected centromeric or her2 spots. Thus, in this circumstance where the morphology of the cells are rather similar (epithelial vs luminal), intensity information can be incorporated into the classifier to improve the classification result.

7.1.2 Tumour vs Stromal Cells

```
# Sample Number 6361: myoepithelial vs luminal cells
# load data.
Dat7347=read.table("data/CellClassification/7347_p2.txt", header=T)
Dat7347.Morph=Dat7347[ ,2:7]
yLabel=Dat7347$Label
```

```r
summary(factor(yLabel))

##  1  2  3
## 49  9 30

# Perform Accuracy Testing on Morphological data only
# Can be performed on 2 and 3 class.
# 2 class: omit the lymphocytes
Lab2=which(yLabel==2)
yNew=factor(yLabel[-Lab2])
levels(yNew)=list("1"=1, "2"=3)
OutSt=TestClassifier(Dat7347.Morph[-Lab2, ], yNew, 500)
theme_set(theme_bw(18))
ggplot(OutSt, aes(x=N, y=MeanVal)) + ylim(0.2, 1)+
    geom_errorbar(aes(ymin=CI.025, ymax=CI.975), width=.1) +
    geom_line() +
    geom_point()+
    ggtitle("Accuracy of classifier: 2 class")+
    xlab("Training Set Size") +
    ylab("Accuracy")
```

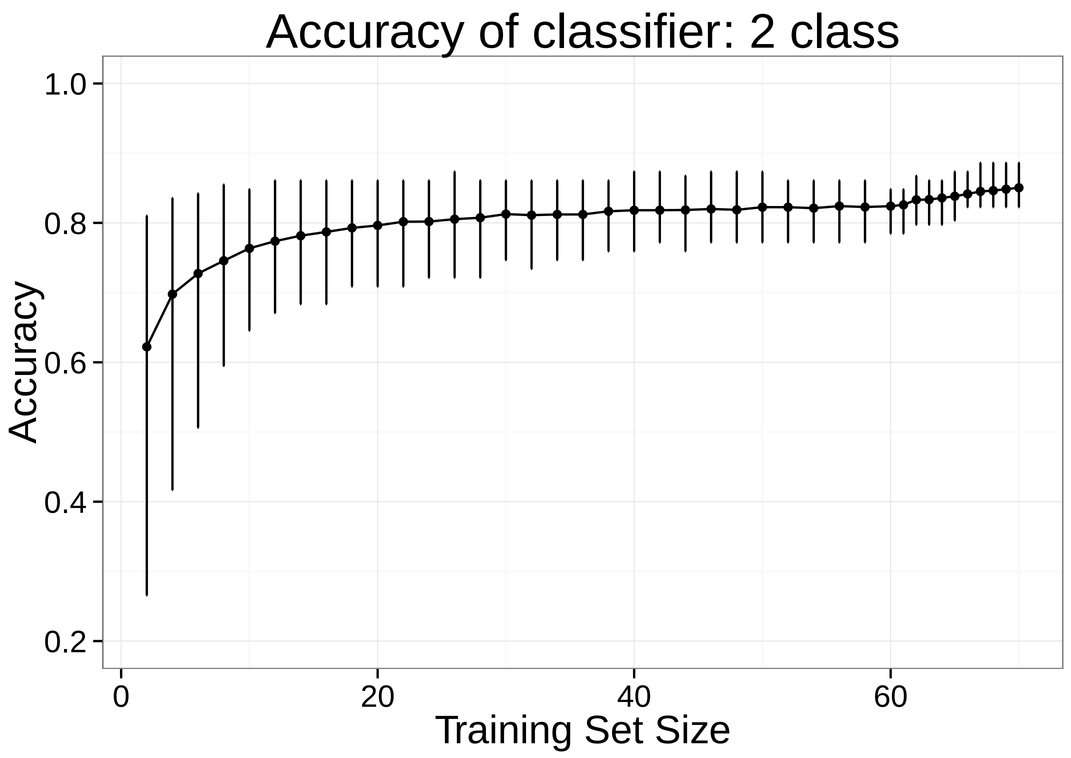

```
# 3 classes:
OutStL=TestClassifier(Dat7347.Morph, yLabel, 500)
theme_set(theme_bw(18))
ggplot(OutStL, aes(x=N, y=MeanVal)) + ylim(0.2, 1)+
    geom_errorbar(aes(ymin=CI.025, ymax=CI.975), width=.1) +
    geom_line() +
    geom_point()+
    ggtitle("Accuracy of classifier: 3 class")+
    xlab("Training Set Size") +
    ylab("Accuracy")
```

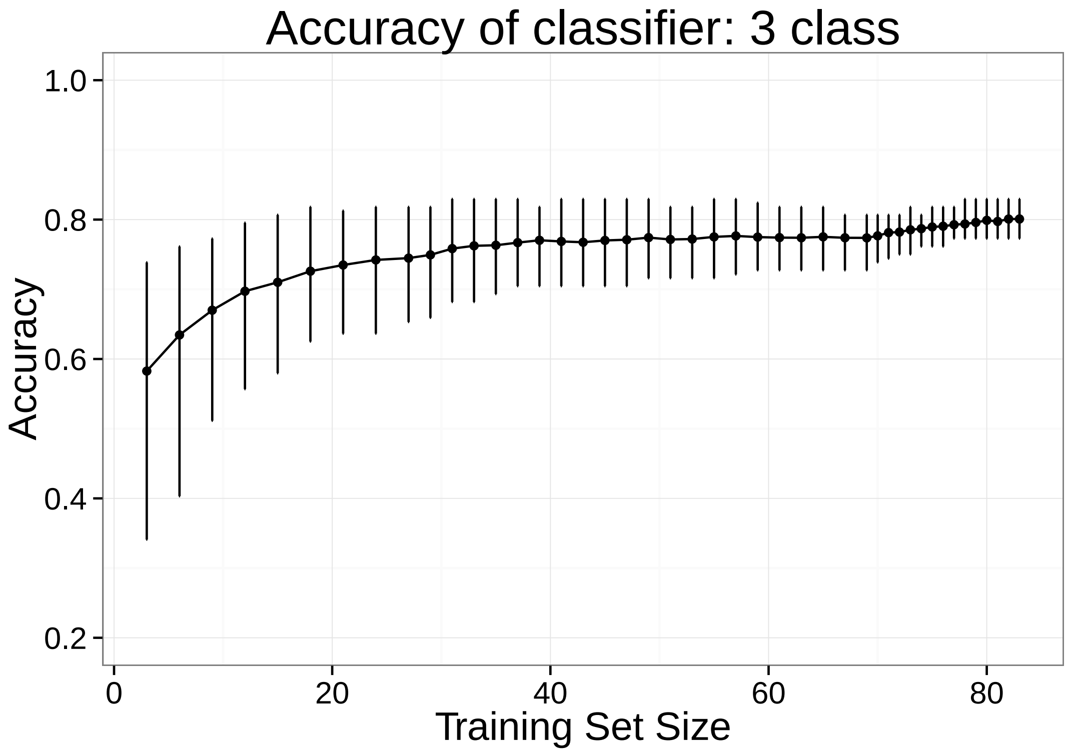

We can add extra stain information and see how the classifier performs:

```
Dat7347.WInt=Dat7347[ ,-c(1, grep("Location", colnames(Dat7347)),31)]
Dat7347.WInt[is.na(Dat7347.WInt)]=0
OutS2=TestClassifier(Dat7347.WInt[-Lab2, ], yNew, 500)
OutStL2=TestClassifier(Dat7347.WInt, yLabel, 500)
# plot the Stromal-cancer classifier with added intensity information
theme_set(theme_bw(18))
ggplot(OutS2, aes(x=N, y=MeanVal)) + ylim(0.2, 1)+
    geom_errorbar(aes(ymin=CI.025, ymax=CI.975), width=.1) +
```

```
    geom_line() +
    geom_point()+
    ggtitle("Accuracy of classifier with variable size of trainings set")+
    xlab("Training Set Size") +
    ylab("Accuracy")
```

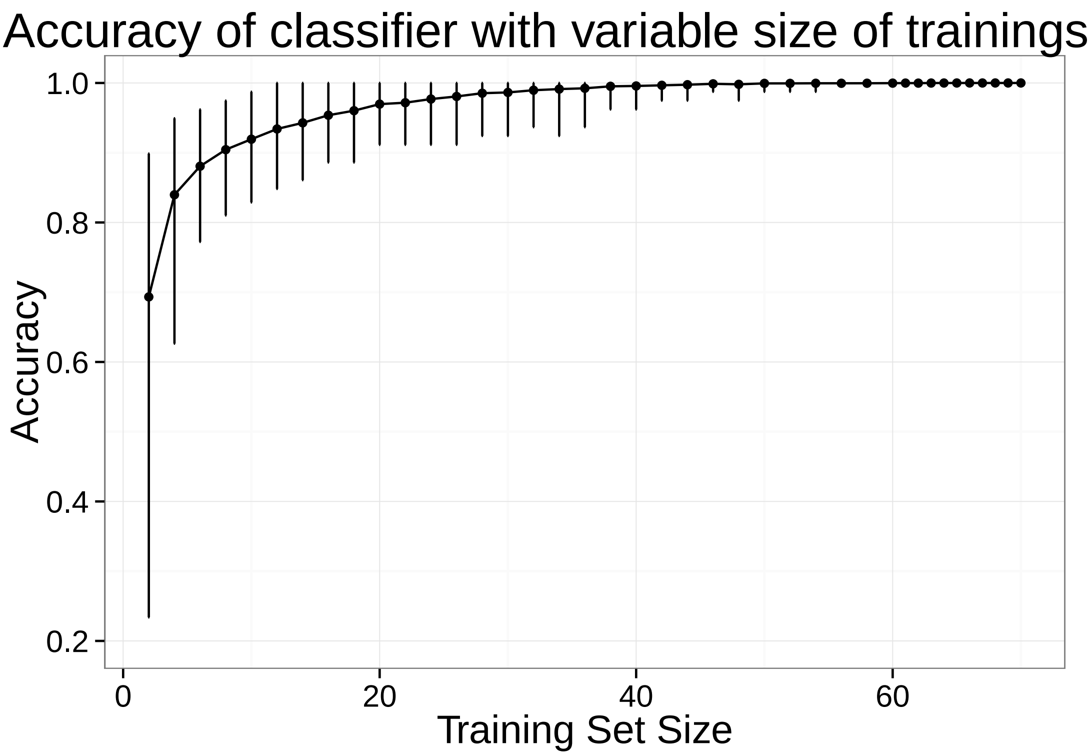


```
# plot the stromal-lymphocyte classifier with added intensity
theme_set(theme_bw(18))
ggplot(OutStL2, aes(x=N, y=MeanVal)) + ylim(0.2, 1)+
    geom_errorbar(aes(ymin=CI.025, ymax=CI.975), width=.1) +
    geom_line() +
    geom_point()+
    ggtitle("Accuracy of classifier with variable size of trainings set")+
    xlab("Training Set Size") +
    ylab("Accuracy")
```

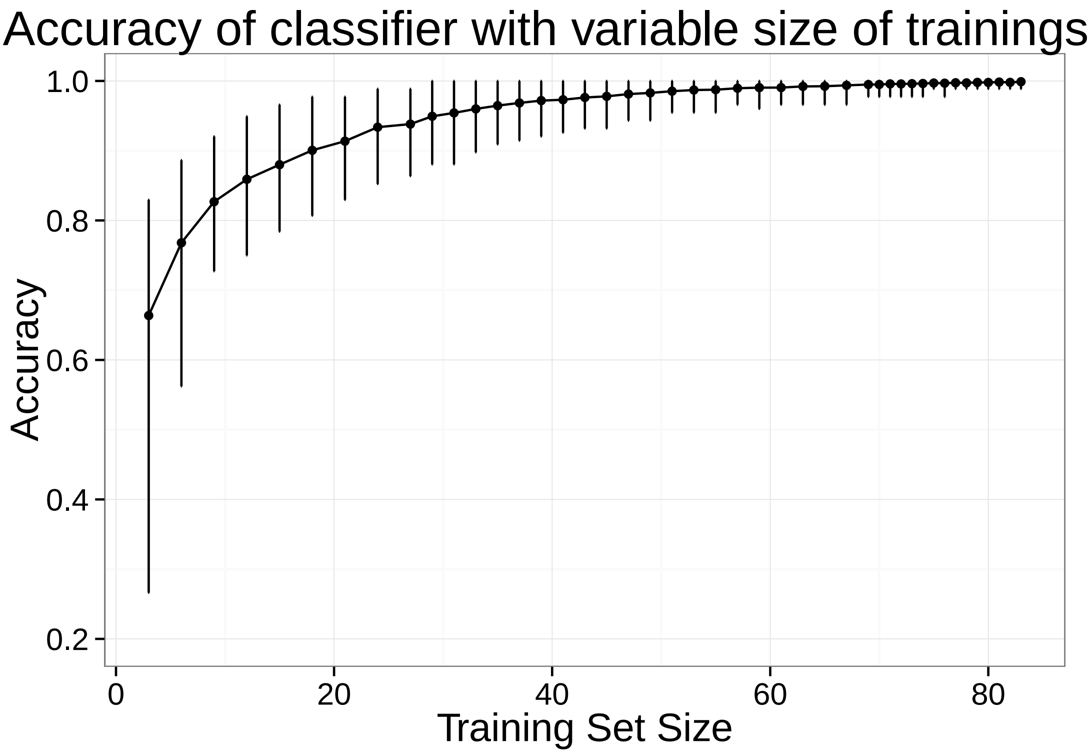

7.2 Background Intensity Variation

In this section we compare the background intensity values attained from 4 observers. Two are trained observers ("T1", "T2"), and two are trained on the spot using the guidelines given in the manual ("O1", "O2").

First we load all background intensities from all 4 observers and see how well they correlate with our gold standard:

```
# Load the dataset:
BackFiles = ReadBackgroundIntensity("data/Background_Stainings/")

# Correlation plot between samples and gold standard
O1 = stack(BackFiles[[1]])
GS = stack(BackFiles[[2]])
T1 = stack(BackFiles[[3]])
T2 = stack(BackFiles[[4]])
plot(O1[, 1], GS[, 1], xlab = "Observers", ylab = "Gold Standard", pch = rep(1:5,
    each = 10))
points(T1[, 1], GS[, 1], pch = rep(1:5, each = 10), col = "red")
points(T2[, 1], GS[, 1], pch = rep(1:5, each = 10), col = "blue")
legend("topleft", c("O1", "T1", "T2"), col = c("black", "red", "blue"), lwd = 2)
```

```
abline(0, 1)
```

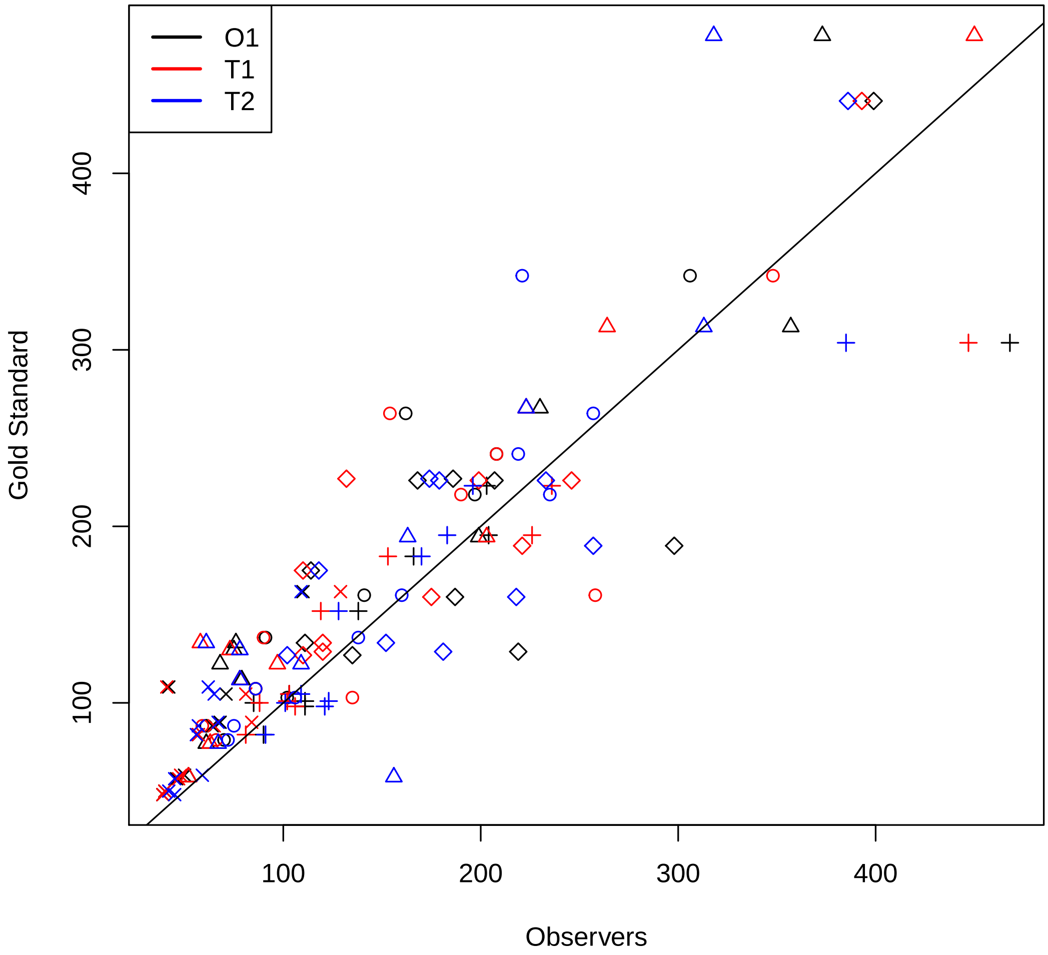


```
# calculate the correlation between samples: Observer 1
cor(O1[, 1], GS[, 1])

## [1] 0.8943

# Trained observer 1
cor(T1[, 1], GS[, 1])

## [1] 0.9158
```

```r
# Trained observer 2
cor(T2[, 1], GS[, 1])

## [1] 0.8862
```

We see that all observers have a correlation close to 0.9 with the gold standard

The second thing we would like to check is the consistency of the scoring across samples: For example, are all nuclear stains scored in the same way

```r
par(mfrow = c(2, 3))
# Obtain a line plot for each of the stains: Nuclear
plot(1:10, GS[1:10, 1], col = "red", type = "b", ylim = c(0, 400), xlab = "Sample",
    ylab = "Background Intensity", main = "DAPI")
lines(1:10, O1[1:10, 1], col = "black", type = "b")
lines(1:10, T1[1:10, 1], col = 3, type = "b")
lines(1:10, T2[1:10, 1], col = 4, type = "b")
# Script for all five stains:
LinePlotIntensity(BackFiles)
```

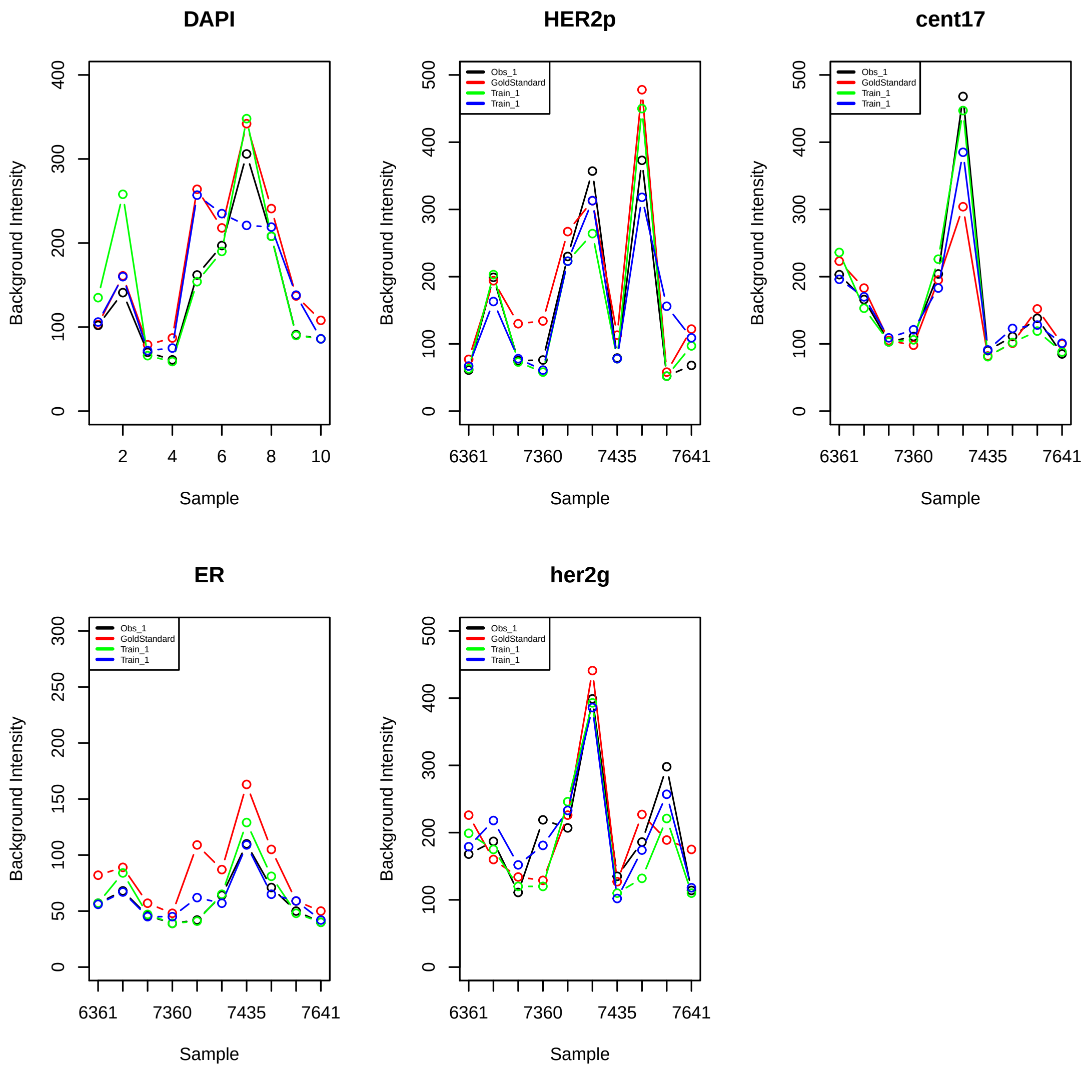

We see that for all observers, the scoring is consistent with the gold standard across most samples.

7.2.1 Heterogeneity in background regions

To determine how heterogeneous a background image is, 4 candidate regions were selected to determine the variability across the image. We compared the standard deviation normalised by the mean (ie. the coefficient of variation):

```
BackVar = read.csv("data/Background_Stainings/GS_areas.csv", header = T)
# create a table of means and SD
Stains = c("DAPI", "Mem", "X1FISH", "Nuclei", "X2FISH")
MeanTable = matrix(NA, nrow = 10, ncol = 5)
```

```
StdTable = matrix(NA, nrow = 10, ncol = 5)
for (i in 1:5) {
    MeanTable[, i] = rowMeans(BackVar[, grep(Stains[i], colnames(BackVar))])
    StdTable[, i] = sapply(1:10, function(x) sd(BackVar[x, grep(Stains[i], colnames(BackV
}
CV = StdTable/MeanTable
rownames(CV) = BackVar$sample
colnames(CV) = c("DAPI", "HER2p", "cent17", "ER", "HER2g")
col.theme = custom.theme(region = brewer.pal(9, "BuPu"))
levelplot(CV[, 5:1], border = "black", border.lwd = 1, par.settings = col.theme,
    shrink = MeanTable * 2/max(MeanTable), colorkey = list(space = "bottom",
        tick.number = 5))
```

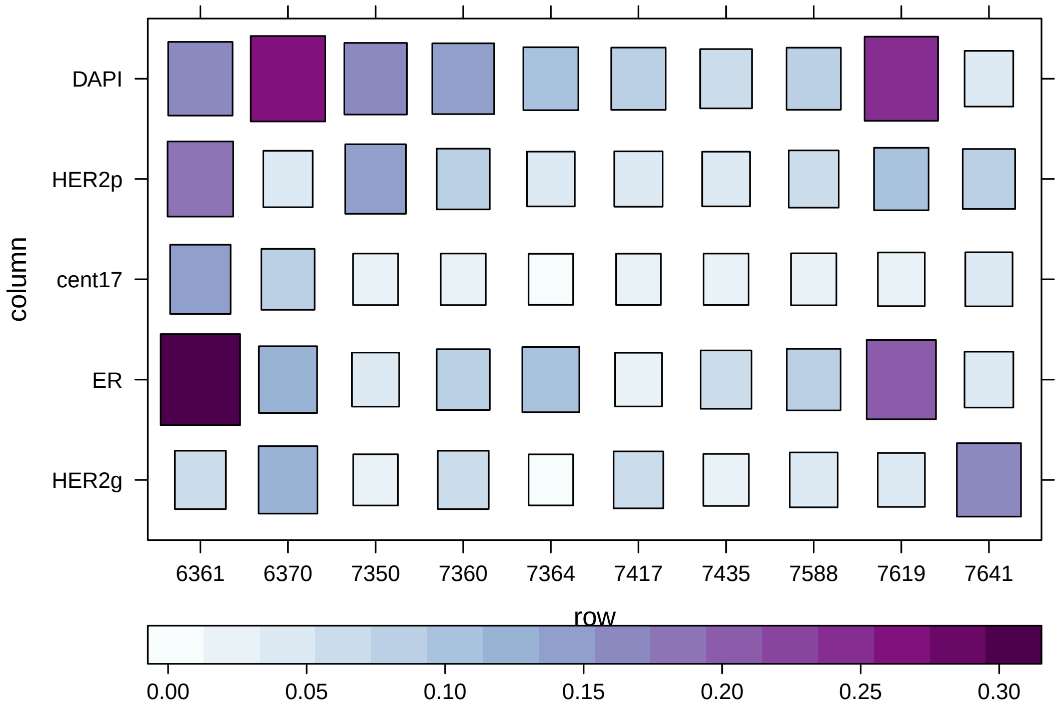

7.3 Benchmarking Differences in Manual Editing

To determine how much user variability is involved with differences in segmentation, 52 poorly segmented cells were fixed by two different observers. This fixing was performed predominantly using a combination of cell cutting and merging, however, the borders can also be painted.

We can plot how the morphology and the intensities of the given cells differ

```
# Load the data file:
InterObs=read.csv('data/inter-obs_seg.csv', header=T)
# Plot the morphology aspect:
plot(InterObs$O2_DAParea, InterObs$O1_DAParea, pch=19,
     xlim=c(0, 18000), ylim=c(0, 18000),
     xlab="Scorer2", ylab="Scorer1",
     main="Morphological Difference in Scoring",
     col="blue")
points(InterObs$O2_HER2Area, InterObs$O1_HER2Area, col=InterObs$HER2.prot, pch=19)
abline(0,1)
```

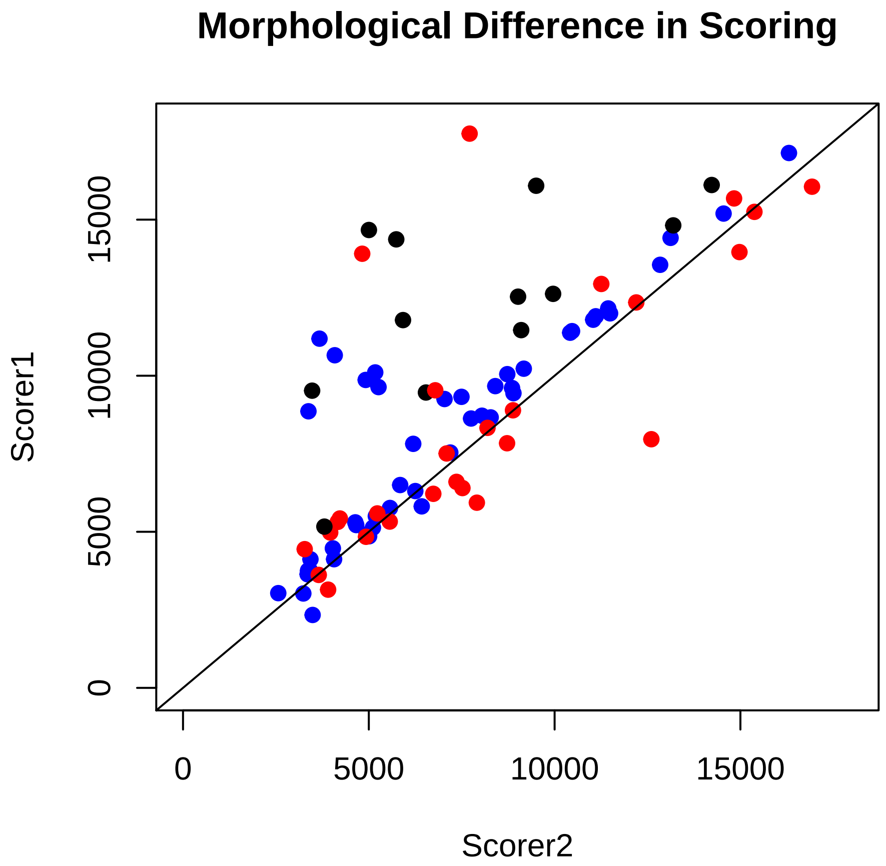


```
## correlation coefficients
cor(InterObs$O2_DAParea, InterObs$O1_DAParea, use="complete")

## [1] 0.8606

## correlation coefficients: HER2 cytoplasmic area
cor(InterObs$O2_HER2Area, InterObs$O1_HER2Area, use="complete")

## [1] 0.7892

cor(InterObs$O2_HER2Area[InterObs$HER2.prot==2],
    InterObs$O1_HER2Area[InterObs$HER2.prot==2], use="complete")

## [1] 0.8299
```

We notice in terms of morphology, the DAPI cells tend to have similar area between the two observers. There are a handful of cells which do not follow this trend: observation

of these cells shows cells which may be undergoing mitosis and is thus classified as a single cell by one Scorer and as 2 cells by the second Scorer. Overall, the consistency is 0.86.

The HER2 cytoplasmic area has most points on the main diagonal, but have the outlines of a number of cells fluctuating. It appears that most of these cells are HER2 negative and thus the membrane region is subject to interpretation in these cases. The correlation is 0.75 for all samples, and 0.79 after removing HER2 positive cases.

We can next look at the intensity of the samples in terms of ER and HER2

```
#
plot(InterObs$O2_HER2Int, InterObs$O1_HER2Int, pch = 19, xlim = c(0, 2250),
    ylim = c(0, 2250), xlab = "Scorer2", ylab = "Scorer1", main = "Intensity Difference i
    col = InterObs$HER2.prot)
points(InterObs$O2_ERInt, InterObs$O1_ERInt, col = "blue", pch = 19)
abline(0, 1)
legend("bottomright", c("HER2-", "HER2+", "ER"), col = c("black", "red", "blue"),
    lwd = 2)
```

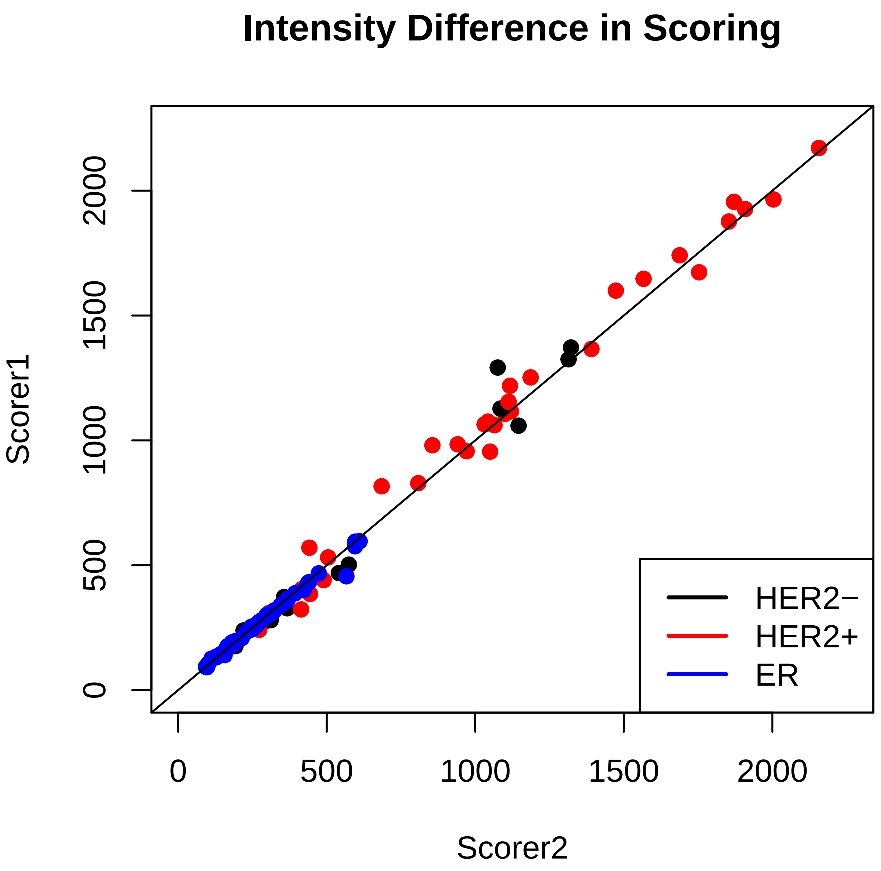

```r
# HER2 intensity correlation
cor(InterObs$O2_HER2Int, InterObs$O1_HER2Int, use = "complete")

## [1] 0.9933

# ER intensity correlation
cor(InterObs$O2_ERInt, InterObs$O1_ERInt, use = "complete")

## [1] 0.9935
```

We see that despite the differences in morphology, the measured intensities strongly correlate between the two scorers (r¿0.99 for both HER2 membrane intensity and ER nuclear intensity)

8 Session info: R-packages and their versions used for this analysis

```
sessionInfo()

## R version 3.0.2 (2013-09-25)
## Platform: x86_64-apple-darwin10.8.0 (64-bit)
##
## locale:
## [1] en_GB.UTF-8/en_GB.UTF-8/en_GB.UTF-8/C/en_GB.UTF-8/en_GB.UTF-8
##
## attached base packages:
## [1] stats     graphics  grDevices utils     datasets  methods   base
##
## other attached packages:
##  [1] latticeExtra_0.6-26 lattice_0.20-24     ggplot2_0.9.3.1
##  [4] e1071_1.6-3         cluster_1.14.4      scales_0.2.3
##  [7] plotrix_3.5-5       RColorBrewer_1.0-5  clinfun_1.0.5
## [10] MASS_7.3-29         knitr_1.5
##
## loaded via a namespace (and not attached):
##  [1] class_7.3-9        codetools_0.2-8    colorspace_1.2-4
##  [4] dichromat_2.0-0    digest_0.6.4       evaluate_0.5.1
##  [7] formatR_0.10       grid_3.0.2         gtable_0.1.2
## [10] highr_0.3          KernSmooth_2.23-10 labeling_0.2
## [13] munsell_0.4.2      mvtnorm_0.9-9997   plyr_1.8
## [16] proto_0.3-10       reshape2_1.2.2     stringr_0.6.2
## [19] tools_3.0.2
```
